# Expression of Hypoxia-Inducible Factor 1α (HIF-1α) and Genes of Related Pathways in Altered Gravity

**DOI:** 10.3390/ijms20020436

**Published:** 2019-01-20

**Authors:** Johannes Vogel, Cora Sandra Thiel, Svantje Tauber, Christian Stockmann, Max Gassmann, Oliver Ullrich

**Affiliations:** 1 Institute of Veterinary Physiology, Vetsuisse Faculty, University of Zurich, Winterthurerstrasse 260, CH-8057 Zurich, Switzerland; jvogel@vetphys.uzh.ch; 2 Institute of Anatomy, Faculty of Medicine, University of Zurich, Winterthurerstrasse 190, 8057 Zurich, Switzerland; cora.thiel@uzh.ch (C.S.T.); svantje.tauber@uzh.ch (S.T.); christian.stockmann@uzh.ch (C.S.); 3 Department of Machine Design, Engineering Design and Product Development, Institute of Mechanical Engineering, Otto-von-Guericke-University Magdeburg, Universitätsplatz 2, 39106 Magdeburg, Germany; 4 Zurich Center for Integrative Human Physiology (ZIHP), University of Zurich, Winterthurerstrasse 190, 8057 Zurich, Switzerland; 5 Space Life Sciences Laboratory (SLSL), Kennedy Space Center, 505 Odyssey Way, Exploration Park, FL 32953, USA

**Keywords:** gene expression, immune system, oxygen, microgravity

## Abstract

Immune system deterioration in space represents a major risk, which has to be mitigated for exploration-class missions into the solar system. Altered gravitational forces have been shown to regulate adaptation processes in cells of the immune system, which are important for appropriate risk management, monitoring and development of countermeasures. T lymphocytes and cells of the monocyte-macrophage system are highly migratory cell types that frequently encounter a wide range of oxygen tensions in human tissues and in hypoxic areas, even under homeostatic conditions. Hypoxia-inducible factor 1 and 2 (HIF’s) might have an important role in activation of T cells and cells of the monocyte-macrophages system. Thus, we investigated the regulation of HIF-dependent and, therefore, hypoxia-signaling systems in both cell types in altered gravity and performed transcript and protein analysis from parabolic flight and suborbital ballistic rocket experiments. We found that HIF-1α and HIF-1-dependent transcripts were differently regulated in altered gravity, whereas HIF-1α-dependent gene expression adapted after 5 min microgravity. Inter-platform comparisons identified PDK1 as highly responsive to gravitational changes in human U937 myelomonocytic cells and in Jurkat T cells. We suggest HIF-1 as a potential pharmacological target for counteracting immune system deterioration during space flight.

## 1. Introduction

The immune system deteriorates during long-term space flights, probably contributing to an increased susceptibility to infection, autoimmunity, and cancer during exploration-class missions [1,2,3,4,5]. Physiological systems strongly respond to altered gravitational forces and adapt to the new gravitational environment within hours until weeks [1]. Cellular and molecular processes are also severely affected in microgravity, including morphology, cell proliferation and differentiation, signal transduction and gene expression (reviewed in [2]) and understanding them is an essential condition for an integrated risk assessment for exploration-class space missions [3]. This is in line with the recommendation of the National Aeronautics and Space Administration (NASA) Inspector General report that potential immune system deterioration in space requires risk mitigation measures in preparation for exploration-class missions into the solar system [4] and of the U.S. National Academies of Science, Engineering and Medicine to investigate more carefully the reversibility of the changes that occur during and after flight [5]. Knowledge about molecular and cellular responses to altered gravity represents crucial information for medical monitoring, development of countermeasures and appropriate risk management of human health and performance-limiting factors during manned space exploration.

T lymphocytes are a highly migratory cell type of the adaptive immune system that frequently encounters a wide range of oxygen tensions in human tissues and in hypoxic areas even under homeostatic conditions [6]: T cells are present in a hypoxic environment in bone marrow [7], spleen [8], in the gastrointestinal tract [9] and following inflammation [10,11]. Effector T cells present at sites of inflammation or retained within non-lymphoid tissues have a high probability of prolonged exposure to a hypoxic environment [6], for example in inflammatory lesions during inflammatory bowel diseases [12] or rheumatoid arthritis [13]. It has been demonstrated that oxygen availability regulates T cell differentiation and function, a response orchestrated in large part by the hypoxia-inducible factor transcription factors [6].

Since mammals, including man, are crucially dependent on oxygen to fuel their highly active metabolism, any shortage of oxygen is an alarm situation for any mammalian cell. Consequently, evolution developed a rapid cellular sensor system that is able to change the gene-expression profile in hypoxic cells instantaneously [14]. The underlying sensor system for this acute response to hypoxia is based on heterodimeric proteins, called hypoxia-inducible factors (HIFs), of which the α-subunits are continuously produced and degraded in the presence of oxygen. During hypoxia, either HIF-1α for most rapid responses or HIF-2α for more delayed ones, both of them representing the master regulators of the cellular response to oxygen shortage, are stabilized and translocated to the nucleus to form HIF transcription complexes that bind the hypoxia response element (HRE) present in the promoter of oxygen-dependent regulated genes. Each HIF is a heterodimer composed of the constitutively expressed β subunit and an α subunit whose stability depends on the oxygen level [15]. During normoxia, HIF-1α and HIF-2α are hydroxylated by prolyl hydroxylases (PHDs), recognized by the von-Hippel–Lindau (VHL) factor, ubiquitinated and degraded by the proteasome. Under hypoxic conditions this mechanism is inactive and HIF-α proteins are not degraded and translocated to the nucleus to form a transcription complex with the HIFβ subunit [16] that induces transcription of genes mediating cellular adaptation to a low oxygen [15]. HIFs can also be stabilized and exert their activity under normoxic conditions in response to bacterial products, cytokines, inflammatory mediators and stress [17]. In the last decades a huge number of oxygen-dependent regulated genes has been identified underlining the importance of this fundamental biological process. Of note, regulation of oxygen-dependent genes occurs almost continuously somewhere in our body and is of significant importance in key physiological processes such as angiogenesis, regulation of red blood cell mass, wound healing or defense against infections [18,19]. Noteworthy, after stabilization of the HIF protein, the response to hypoxia requires rapid nuclear transfer of HIF that is dependent on the cytoskeleton to trigger the transcription of hypoxia-inducible genes [20] and gravitational changes have immediate impact on the cytoskeleton [21]. In addition to transcriptional regulation, gene expression can be controlled via retention or release of transcribed mRNA in response to various types of cellular stress [22,23,24] and this has also been demonstrated for HIF-dependent transcripts [24].

HIF-1α has been identified as a regulator of T cell responses [10,25], which is induced STAT3-dependent in naive CD4 T cells, even in the presence of oxygen [16] and promotes expression of several Th17-linked genes [26]. Thus, elevated HIF-1 levels, stabilized by either cytokine or T cell receptor (TCR) signaling or hypoxic conditions, should promote a strong and enduring Th17 response [16]. HIF-1 drives the expression of a number of genes necessary for the shift to a glycolysis dominated metabolism [27,28], resulting in metabolic reprogramming of T cells [29,30]. HIF-1-induced signaling pathways during metabolic reprogramming includes mTOR and PI3K/Akt [31]. Also, Notch signaling and antiapoptotic gene expression are under the control of HIF-1 [32]. Therefore, HIF-1α might have an important role in T cell activation and differentiation programs beyond its well-known role in coordinating hypoxic responses [6].

In the monocyte-macrophage-system, HIF promotes bactericidal activities and supports the innate immune functions [33]. A hypoxic microenvironment imposes a metabolic adaptation to macrophages, skewing their functions towards a mitogenic, pro-invasive and pro-angiogenic phenotype [33]. In damaged tissues, HIF-1α and HIF-2α are up-regulated in macrophages, where they orchestrate post-infarction remodeling [34].

Thus, the question arises, whether and how microgravity interferes with HIF-dependent and, therefore, hypoxia signaling systems in T cells and cells of the monocyte-macrophage system. Therefore, we employed three different cell lines, two for transcript analysis and one for quantifying cellular HIF-1α protein content in response to hypergravity (1.8 g to 13.5 g) as well as in microgravity (µg) and conducted experiments on board parabolic flights and during suborbital ballistic rocket missions. We found that the HIF-1α and hypoxia-inducible transcripts were differently regulated in altered gravity, whereas HIF-1α-dependent gene expression adapted after 5 min microgravity. Our data indicate that HIF-1α and subsequent signaling pathways are significantly regulated by altered gravity.

## 2. Results

### 2.1. Altered Gravity Regulates Hypoxia-Inducible Factor (HIF)-1α-Dependent Gene Expression in Human Jurkat T Cells and Myelomonocytic U937 Cells and HIF-1α Protein Content in MDA-MB468 cells

**Figure 1:**
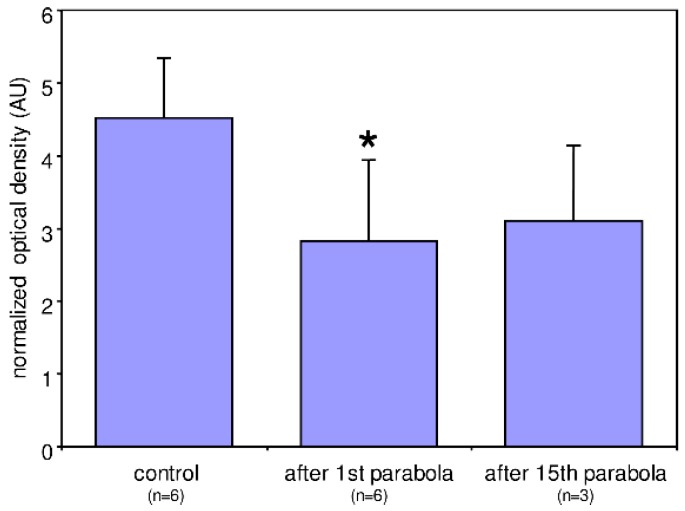
Average optical density of hypoxia-inducible factor (HIF)-1α-positive bands in Western blots of protein extracts from MDA-MB-468 cells normalized to total protein of the conditions as indicated. Compared to the control conditions (i.e., in 1 g in-flight condition before the first parabola) HIF-1α expression after the 1st parabola was significantly (* = *p* < 0.05) about 40% lower and stayed this low until the last (15th) parabola although there was a slight trend to a recovery of the HIF-1α expression (*p* = 0.075 vs. control).

HIF-1α is a rapid master transcriptional regulator of the adaptive response to hypoxia and continuously produced and degraded in the presence of oxygen. Thus, even slight imbalances between production and degradation will alter the cellular HIF-1α protein content and subsequently HIF-1α-dependent gene expression. We used MDA-MB-468 cells during the first Swiss Parabolic Flight Campaign to assess whether gravitational stress may change cellular HIF-1α protein content. MDA-MB-468 cells are a commonly used model in the study of cellular response to hypoxia [35]. Cells were harvested after being subjected to 20 s of hypergravity (1.8 g) followed by 20 s of microgravity during the first parabola of the first Swiss Parabolic Flight Campaign. Another set of cells was harvested at the end of the 15th parabola. Protein extracts were analyzed using semi-quantitative Western blotting [36]. Compared to the control conditions (i.e., in 1 g in-flight condition before the first parabola), cellular HIF-1α protein content after the first parabola was significantly about 40% lower and remained low until the last (15th) parabola (Figure 1).

Therefore, in a next step we used the data of four different microgravity campaigns to analyze hyper- and microgravity short term and midterm effects of gravitational alterations on HIF-1α-RNA expression and expression of numerous HIF1α-dependently regulated genes. Parabolic flights were used for the analysis of short-term altered gravity effects and suborbital rocket flights to detect time dependent dynamic adaptation processes within minutes (Table 1).

**Figure 2:**
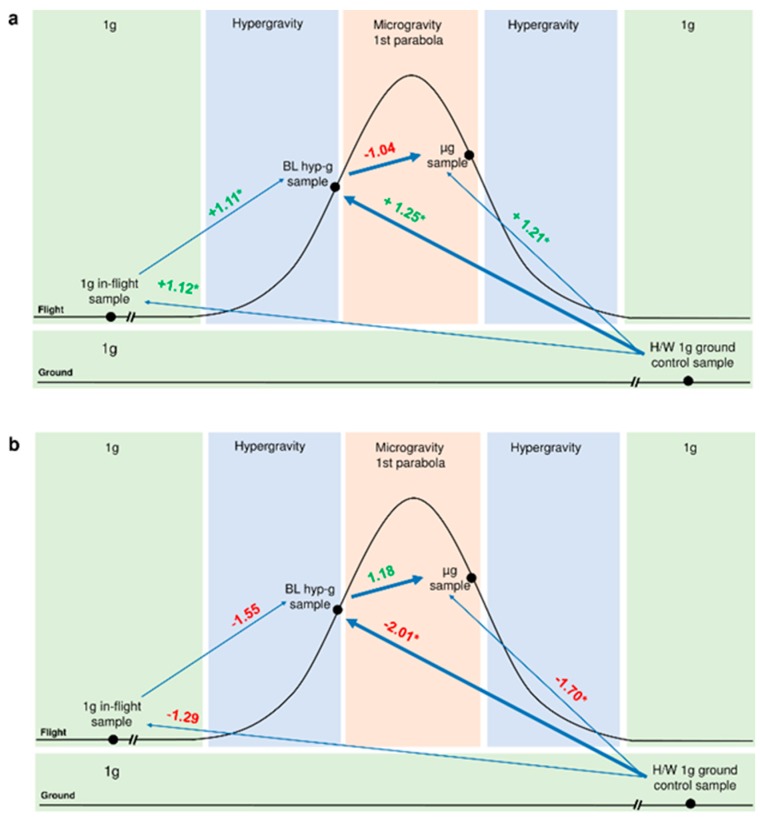
Differential gene expression of HIF-1α in (**a**) human Jurkat T cells and (**b**) U937 cells after 20 s altered gravity on a parabolic flight. The gene expression regulation is displayed for inter-phase comparisons as fold change numbers next to the blue arrows connecting the compared experimental conditions. Shown are the RNA expression values for the comparisons: 1 g in-flight versus hardware (H/W) 1 g ground control, baseline/hypergravity (BL hyp-g) versus 1 g in-flight, BL hyp-g versus H/W 1 g ground control, microgravity (µg) versus BL hyp-g and µg versus H/W 1 g ground control. For definition of the abbreviations of the conditions see also Table 1. (* *p*-value < 0.05, red = down-regulation, green = up-regulation).

**Figure 3:**
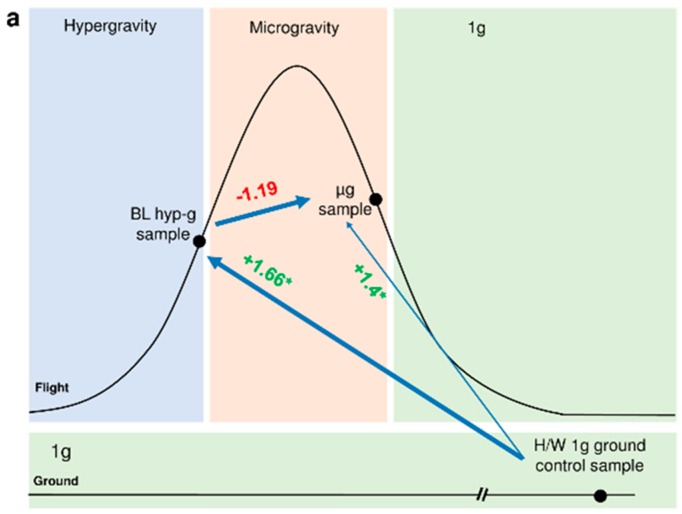

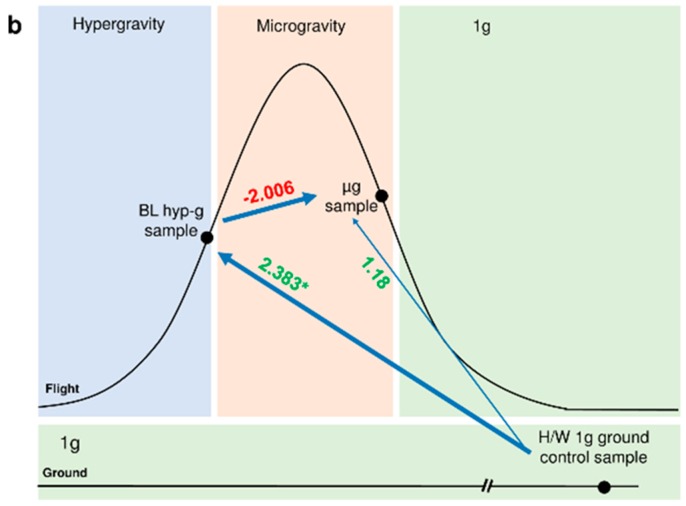
Differential gene expression of HIF-1α in (**a**) human Jurkat T cells and (**b**) U937 cells after 75 s and 300 s of altered gravity on a suborbital rocket mission. The gene expression regulation is displayed for inter-phase comparisons as fold change numbers next to the blue arrows connecting the compared experimental conditions. Shown are the RNA expression values for the comparisons: baseline/hypergravity (BL hyp-g) versus H/W 1 g ground control, microgravity (µg) versus BL hyp-g and µg versus H/W 1 g ground control. For definition of the abbreviations of the conditions see also Table 1. (* *p*-value < 0.05, red = down-regulation, green = up-regulation).

Experiments were performed with human Jurkat T cells and U937 cells to investigate general altered gravity induced effects on the transcriptome. As previously described [37,38,39], human Jurkat T cells as well as human U937 myelomonocytic cells were exposed to 20 s of hypergravity (1.8 g) and subsequently to 20 s of microgravity (µg) during the first parabola of a parabolic flight (19th and 23rd German Aerospace Center (DLR) Parabolic Flight Campaigns (PFC). Cell samples were lysed at the end of the respective flight phase. Corresponding 1 g in-flight control samples were taken five minutes before the first parabola, and 1g ground controls (GC) were taken on ground directly after the flight on board the aircraft under identical conditions except for the gravitational force. In Jurkat T cells and U937 cells, expression of HIF-1α mRNA was significantly affected when comparing hardware 1 g ground control samples with the hypergravity phase (Jurkat T cells: fold change (FC) = +1.25, *p* = 0.000003, U937: FC = −2.01, *p* = 0.020615). The subsequent microgravity phase showed only minimal, non-significant alterations of HIF1α expression (Figure 2). During the sounding rocket experiments (Technologische Experimente unter Schwerelosigkeit (TEXUS)) TEXUS-49 and TEXUS-51, hypergravity samples were acquired 75 s after lift-off, at the end of the hypergravity phase and after five minutes of microgravity. Ground control samples were performed in identical hardware units and under identical conditions except for the gravitational force. In case of the TEXUS-51 mission an on-board centrifuge allowed 1 g in-flight controls during the microgravity phase. Additionally, 1 g controls under standard cell culture conditions were carried out on the ground to monitor potential hardware effects on the experiment. In both suborbital ballistic rocket experiments we were able to identify a significant upregulation of HIF-1α expression after the 75 s hypergravity phase (Jurkat T cells: FC = +1.66, *p* = 0.000736, U937: FC = +2.383, *p* = 0.002885) and a tendency to recover after 5 min of microgravity (Figure 3). For the parabolic flight and suborbital rocket campaigns, a minimum of four RNA samples for each experiment group was isolated and processed for microarray analyses (see Materials and Methods).

### 2.2. Analysis of Hypoxia-Inducible Factor 1α Gene Expression

The differential gene expression of Hypoxia-inducible factor 1α (HIF-1α) was analyzed in detail for the different phases of a parabolic flight. Figure 2a shows the gene expression of HIF-1α measured in human Jurkat T cells. Interestingly, the force environment during the take-off and steady flight phase of the aircraft (<1.3 g, and vibrations of <±0.09 g) contributed already to a significant up-regulation of gene expression. The exposure of the cells to the first hypergravity phase increased this effect (Figure 2a). Exposure of the cells to the following microgravity phase, however, did not further affect the gene expression level. A similar trend could be observed for human myelomonocytic U937 cells. Again, the hypergravity generated during the take-off already influenced the HIF-1α gene expression and this effect was increased during the hypergravity phase of the first parabola (Figure 2b). However, the HIF-1α gene expression was downregulated in this cell type. As seen for the human Jurkat T cells, the following 20 s microgravity phase did not reverse or further influence this effect.

When we analyzed the midterm effects of altered gravity on the gene expression of human Jurkat T cells on a suborbital rocket flight, we also identified an up-regulation of HIF-1α after 75 s of hypergravity and a stable gene expression with a tendency to a counter-regulation in the subsequent microgravity phase (Figure 3a). In the human myelomonocytic U937 cells, we also identified an up-regulation of HIF-1α gene expression after 75 s of hypergravity with a rapid counter-regulation in the following microgravity phase (Figure 3b). The altered gravity-based transcriptome changes could be confirmed by measurements of HIF-1α protein expression levels in the breast cancer cell line MDA-MB-468 performed in an independent hardware on the First Swiss Parabolic Flight (Figure 1). In accordance with the gene expression data of the human myelomonocytic U937 cells we identified a down-regulation of the protein content (Figure 1), which suggests together with the aforementioned RNA-data that also HI-F1α-dependent gene expression might be altered in different gravitational environment.

### 2.3. Analysis of Hypoxia-Inducible Factor-Related Genes

This rapid reactivity of HIF-1α mRNA and protein to altered gravity encouraged us to extend the gene expression analysis to HIF-1α-regulated genes. In total, we selected 176 HIF-related genes (Supplementary Table S1). These are represented by 375 probe sets on the applied NimbleGen arrays based on the hg18 annotation, used for analysis of the U937 cells; 175 of the 176 genes (one gene was not present on the array) are represented by 184 transcript clusters (for simplicity also referred to as probe sets in this paper) on the Affymetrix GeneChip^®^ Human Transcriptome Array (Thermo Fisher Scientific, Waltham, MA, USA) used for analysis of the Jurkat T cells. For the respective gravity conditions, the differential gene expression was analyzed and expression changes between microgravity and hypergravity samples and the corresponding control samples were determined (Tables 2–5 and 7–10, Supplementary Tables S2–S5). During PFC and suborbital ballistic rocket missions, the first altered gravity condition is always represented by a hypergravity phase including other conditions like hardware effects on cell cultivation, temperature changes, and vibrations. This phase is followed by the microgravity phase. In order to avoid including prolonged or delayed hypergravity effects in the groups of microgravity-sensitive transcripts, probe sets that were expressed in the same direction as in the preceding hypergravity phase were excluded and the results are termed “controlled” versions. The probe sets were grouped into “no response” (no significant changes), “continuous response” (changes in the same direction compared to the initial response), “adaptation” (changes in the opposite direction compared to the initial response), and “late response” (no response after 20 s, but later significant response) according to duration and quality of the response to the respective gravity condition. The HIF signaling related and differentially regulated probe sets of the mentioned categories were further investigated to identify their time dependent response to the different altered gravity conditions.

### 2.4. Rapid Alterations of HIF-Related Genes after 20 s of Altered Gravity in Human Jurkat T cells

**Figure 4:**
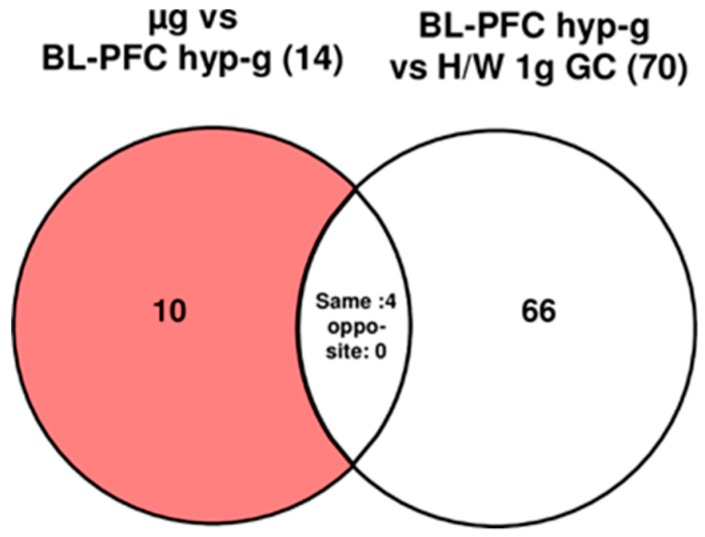
Strategy for the identification of microgravity- and hypergravity-sensitive probe sets in human Jurkat T cells during the 23rd DLR PFC. The different comparisons and resulting intersections for hypergravity and microgravity-sensitive probe sets are displayed as Venn-diagrams. In the circles numbers of probe sets are given that are differentially expressed with a *p*-value < 0.05. The red area shows the number of BL-controlled (BL-PFC hyp-g versus H/W 1 g GC) microgravity-sensitive transcripts: differentially expressed in the comparison µg versus BL-PFC hyp-g excluding those differentially expressed in the control comparison BL-PFC hyp-g versus H/W 1 g GC that are regulated in the same direction. For the abbreviations of the experiment group names see Table 1.

Already after take-off of the airplane and the associated hypergravity and vibration conditions, 65% of the HIF-related probe sets (114 out of 175) were significantly differentially expressed (comparison 1 g in flight (IF) versus hardware (H/W) 1 g ground control (GC)). In human Jurkat T cells 38 differentially regulated HIF-related probe sets showed a response within 20 s of hypergravity (hyp-g) during the first parabola (comparison of experiment groups baseline/hypergravity (BL-PFC hyp-g) versus 1 g IF, Table 2); 27 of these 38 probe sets, i.e., 71%, overlap with the 114 initially identified probe sets from the comparison 1 g IF versus H/W 1 g GC. After the subsequent first microgravity phase, 14 probe sets (microgravity (µg) versus BL-PFC hyp-g) were differentially expressed. When the number of hypergravity-sensitive probe sets was determined with respect to the H/W 1 g GC control group, 70 differentially expressed probe sets were identified (Figure 4, Table 2 and Table 3). After exclusion of the probe sets that were already altered in the control comparison (differentially expressed in the same direction of the comparisons µg versus BL-PFC hyp-g and BL-PFC hyp-g versus H/W 1 g GC), we revealed 10 microgravity-sensitive probe sets (µg versus BL-PFC hyp-g) (Figure 4 and Table 3). The analysis of probe sets double-sensitive for hyper- and microgravity (BL-PFC hyp-g versus H/W 1 g GC and μg versus BL-PFC hyp-g), revealed four probe sets being differentially regulated in the same direction and no probe set that was expressed in the opposite direction (Figure 4). The maximum and minimum expression fold change (FC) values for hypergravity-sensitive probe sets were +1.39 and –1.62, with average figures of +1.15 and −1.17, respectively. The maximum and minimum expression FC values for microgravity sensitive probe sets were +1.11 and −1.07, with average figures of +1.08 and −1.04, respectively (Table 3).

### 2.5. Strong Response of HIF-Related Genes in Human Jurkat T Cells After 75 s of Hypergravity but Not After 300 s of Microgravity

**Figure 5:**
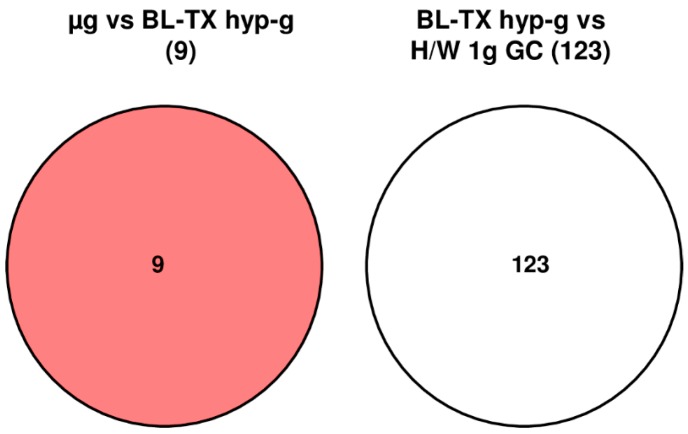
Strategy for the identification of hypergravity- and microgravity-sensitive probe sets from human Jurkat T cells during the TEXUS-51 sounding rocket flight. The different comparisons for hypergravity and microgravity-sensitive probe sets are displayed as Venn-diagrams. In the circles numbers of probe sets are given that are differentially expressed with a *p*-value < 0.05. The red area shows the number of microgravity-sensitive probe sets. There is no overlap between the identified probe sets for the comparisons µg versus BL-TX hyp-g and BL-TX hyp-g versus H/W 1 g GC. For the abbreviations of the experiment group names see Table 1.

During the launch phase of the suborbital ballistic rocket experiment, human Jurkat T cells responded rapidly within 75 s covering the hypergravity phase with a total number of 123 differentially regulated HIF-related probe sets (comparison baseline/hypergravity (BL-TX hyp-g) versus H/W 1 g GC, Table 4 and Table 5, Figure 5). After the subsequent 300 s microgravity phase, only 9 probe sets were differentially expressed (µg versus BL-TX hyp-g, Table 4 and Table 5, Figure 5), which is 13.6-fold lower than the number of differentially expressed probe sets in hypergravity. Interestingly and compared to the parabolic flight experiments, here none of the identified probe sets was hypergravity and microgravity double-sensitive (BL-TX hyp-g versus H/W 1 g GC and μg versus BL-TX hyp-g) (Figure 5). The expression fold change (FC) was in the range between +1.76 (up-regulation) and −1.35 (down-regulation), with average values of +1.26 and −1.14, respectively, for the hypergravity-sensitive probe sets and between +1.08 (up-regulation) and −1.15 (down-regulation) with average values of +1.05 and −1.10, respectively, for microgravity-sensitive probe sets (Table 5). Note that in these experiments with a much stronger and longer hypergravity phase, the number of hypergravity sensitive genes is far higher than in parabolic flight experiments (Table 3).

### 2.6. HIF-Related Genes Were Primarily Differentially Regulated in Hypergravity in Human Jurkat T Cells

**Figure 6:**
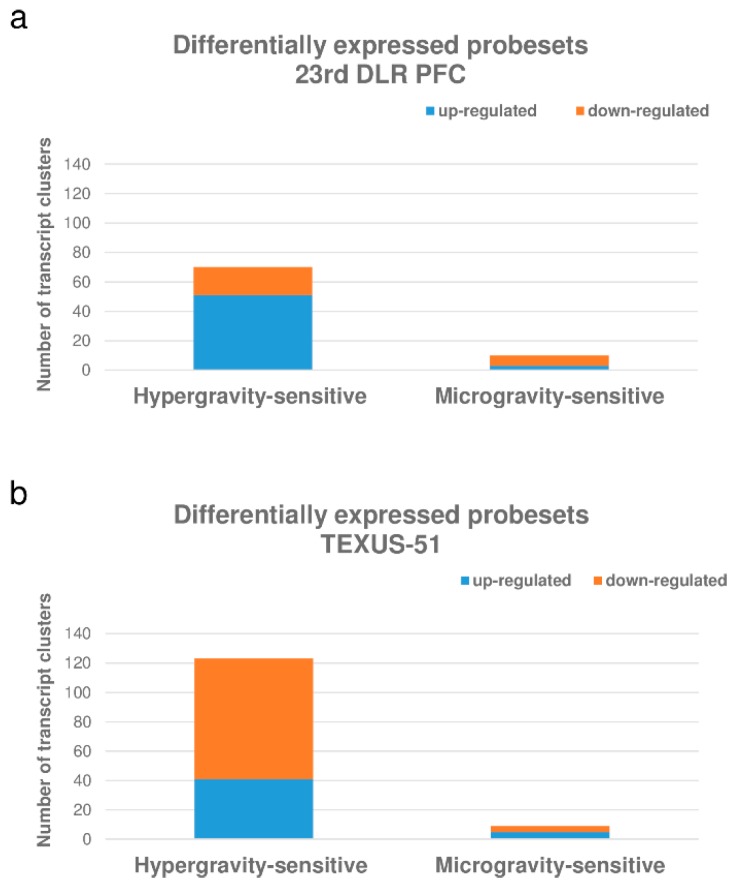
Distribution of differentially expressed probe sets representing HIF-related genes in human Jurkat T cells. (**a**) Number of hyper- and microgravity-sensitive probe sets (10^−2^–10^−3^ g/1.8 g) identified in the 23rd DLR Parabolic Flight Campaign, (**b**) Number of hyper- and microgravity sensitive probe sets (10^−4^ g/5–7 g) identified in the TEXUS-51 suborbital ballistic rocket campaign.

Figure 6 summarizes the number of differentially expressed probe sets in the parabolic flight and suborbital ballistic rocket experiments. Most of the differentially regulated probe sets were up-regulated after 20 s of hypergravity, whereas only a small fraction of the analyzed probe sets was differentially regulated at all after 20 s of microgravity (Figure 6a). Longer periods of hypergravity (75 s 5–7 g) resulted primarily in the down-regulation of probe sets while, after longer periods of microgravity (5 min 10^−4^ g), only a few probe sets remained differentially regulated (Figure 6b).

### 2.7. Dynamic Reaction and Adaptation Processes of Initially Differentially Altered HIF-Related Genes in Altered Gravity in Human Jurkat T Cells

**Figure 7:**
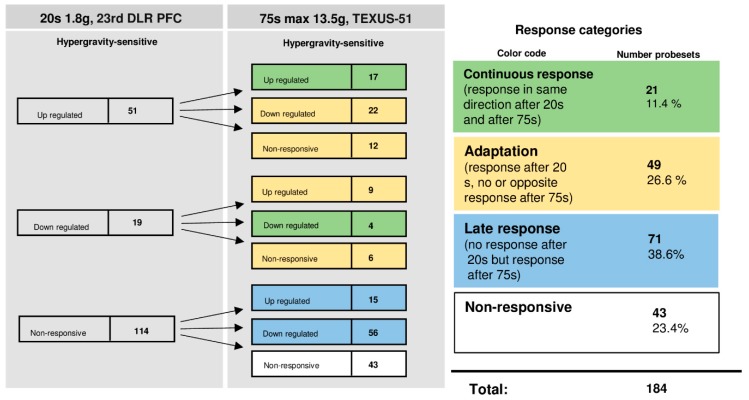
Time course of differential expression of 184 HIF-related probe sets in human Jurkat T cells after hypergravity exposure (list of probe sets in Supplementary Table S1). Human Jurkat T cells were exposed to 20 s and 75 s of hypergravity. The probe sets are grouped according to their regulation after the two exposure times. Continuous response means that the probe sets are either up-regulated or down-regulated at both time points. Adaptation is either the disappearance of the hypergravity-induced effect or regulation in the opposite direction after 75 s. Late response is a regulation that only appears after 75 s. Most of the probe sets show a late response. For the abbreviations of the experiment group names see Table 1.

**Figure 8:**
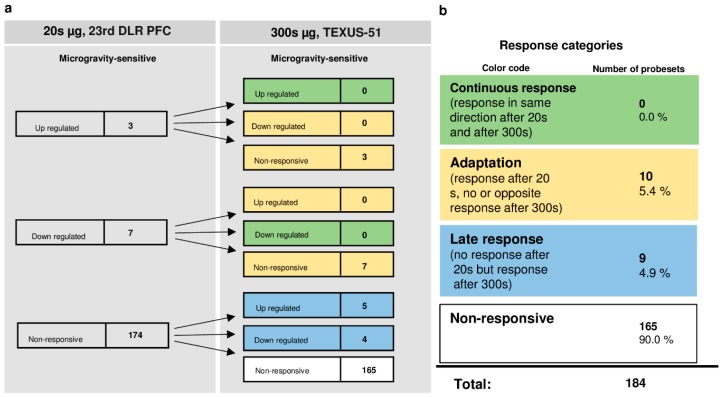
Time course of differential expression of 184 probe sets in human Jurkat T cells after microgravity exposure (list of probe sets in Supplementary Table S1). Human Jurkat T cells were exposed to 20 s and 300 s of microgravity. The probe sets are grouped according to their regulation after the two exposure times. Continuous response means that probe sets are either up-regulated or down-regulated at both time points. Adaptation is either the disappearance of the microgravity-induced effect or regulation in the opposite direction after 300 s. A late response is a regulation that only appears after 300 s. Most of the probe sets did not respond to microgravity at either time point. For the abbreviations of the experiment group names see Table 1.

The time course of differentially regulated probe sets in 1.8 g and 13.5 g of hypergravity is summarized in Figure 7, and that of microgravity is summarized in Figure 8. In hypergravity, a total number of 43 probe sets (23.4%) were not altered at all. Out of 70 initially altered probe sets, after 20 s in 1.8 g, 21 probe sets (11.4%) were altered in the same direction after 75 s in a maximum of 13.5 g (Table 6). These 21 probe sets, belonging to 21 genes, were differentially regulated in a continuous manner after 20 s and 75 s of hypergravity (Table 6). In contrast, 49 probe sets (26.6%) adapted. Furthermore, 71 probe sets (38.6%) were altered after 75 s in a maximum of 13.5 g, but not after 20 s of 1.8 g of hypergravity.

Interestingly, only a small number of HIF-related probe sets were regulated in the microgravity phase. A total number of 165 probe sets (90%) were not altered at all. Out of 10 initially altered probe sets after 20 s, no probe set (0%) was altered in the same direction after 300 s, whereas all 10 initially differentially expressed probe sets (5.4%) adapted. A further nine probe sets (4.9%) were altered after 300 s, but not after 20 s of microgravity. Thus, in our experiments, which have been conducted on two different experimental platforms (23rd DLR parabolic flight campaign and TEXUS-51 suborbital ballistic rocket campaign), we identified two pools of altered probe sets: a first one, which responded after seconds and adapted within 5 min at the latest, and a second one, which appeared not before 5 min of altered gravity.

### 2.8. Rapid Alterations of HIF-Related Genes After 20 s of Altered Gravity in Human U937 Cells

**Figure 9:**
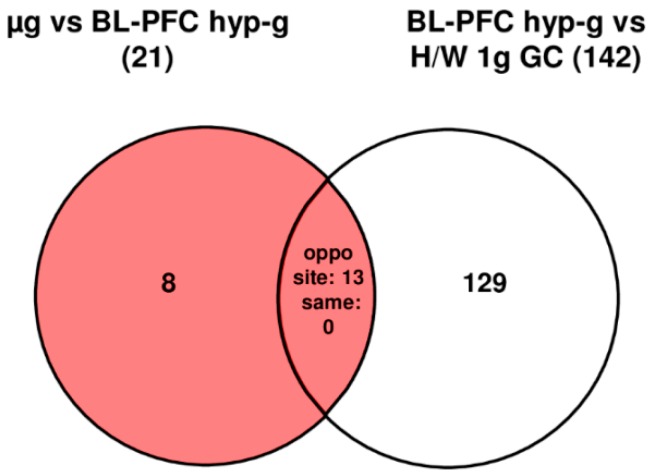
Strategy for the identification of hypergravity- and microgravity-sensitive probe sets in human U937 cells for the 19th DLR PFC. The different comparisons and resulting intersections for hypergravity and microgravity-sensitive probe sets are displayed as Venn-diagrams. In the circles numbers of probe sets are given that are differentially expressed with a *p*-value < 0.05. The red area shows the number of BL-controlled (BL-PFC hyp-g versus H/W 1 g GC) microgravity-sensitive transcripts: differentially expressed in the comparison µg versus BL-PFC hyp-g excluding those differentially expressed in the control comparison BL-PFC hyp-g versus H/W 1 g GC that are regulated in the same direction. For the abbreviations of the experiment group names see Table 1.

In human U937 cells we were also able to detect a cellular response at the transcriptome level to the hypergravity and vibration effects that is already present during the take-off of the parabolic flight aircraft; 45 out of 375 analyzed probe sets (12%) were significantly differentially expressed (comparison 1 g in flight (IF) versus hardware (H/W) 1 g ground control (GC)). In contrast to human Jurkat T cells, HIF-related probe sets in U937 cells responded even more sensitive to gravitational alterations. The hypergravity phase of the first parabola resulted in 104 differentially regulated probe sets in the comparison BL-PFC hyp-g versus 1 g IF, a 2.3-fold increase compared to the initial response (Table 7). Nine of the 45 initially responding probe sets, overlapped with the 104 regulated probe sets identified during the first hypergravity phase (data not shown). In the direct comparison BL-PFC hyp-g versus H/W 1 g GC 142 differentially expressed transcripts could be identified (Table 7 and Table 8, Figure 9). After the subsequent first microgravity phase, only 21 probe sets (µg versus BL-PFC hyp-g) were differentially expressed (Table 7 and Table 8, Figure 9). Note however, that in Jurkat T cells only nine probe sets were sensitive under the same condition. No probe set could be excluded based on the control comparison: differentially expressed in the same direction of the comparisons µg versus BL-PFC hyp-g and BL-PFC hyp-g versus H/W 1 g GC (Figure 9). In the investigation of hypergravity and microgravity double-sensitive probe sets (BL-PFC hyp-g versus H/W 1 g GC and µg versus BL-PFC hyp-g), 13 of the probe sets in the intersection were differentially expressed in the opposite direction, and none of the probe sets was differentially expressed in the same direction (Figure 9). The maximum and minimum expression FC values for probe sets sensitive to hypergravity were +3.60 and −6.46 with average figures of +2.00 and −2.00, respectively. The maximum and minimum expression FC for probe sets sensitive to microgravity were +1.82 and −2.09 with average figures of +1.33 and −1.46, respectively (Table 8). Altogether, compared to Jurkat T cells, U937 cells were in general more sensitive to gravitational alterations but similar as Jurkat cells also U937 cells were even more sensitive to hypergravity.

### 2.9. Strong Response of HIF-Related Genes in Human U937 Cells after 75 s of Hypergravity and After 300 s of Microgravity

**Figure 10:**
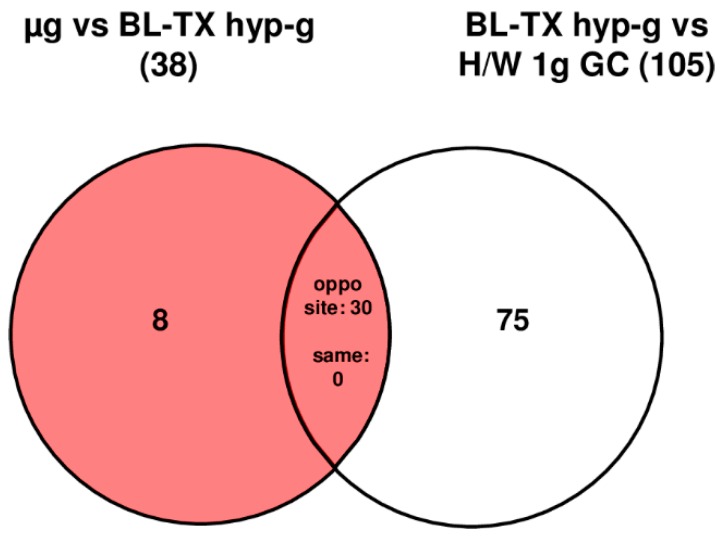
Strategy for the identification of hyper- and microgravity-sensitive probe sets in human U937 cells during the TEXUS-49 sounding rocket flight. The different comparisons and resulting intersections for hypergravity and microgravity-sensitive probe sets are displayed as Venn-diagrams. In the circles numbers of probe sets are given that are differentially expressed with a *p*-value < 0.05. The red area shows the number of BL-controlled (BL-PFC hyp-g versus H/W 1 g GC) microgravity-sensitive transcripts: differentially expressed in the comparison µg versus BL-TX hyp-g excluding those differentially expressed in the control comparison BL-TX hyp-g versus H/W 1 g GC that are regulated in the same direction. For the abbreviations of the experiment group names see Table 1.

During the suborbital ballistic rocket based investigation of human U937 cells, 105 HIF-related probe sets were differentially regulated after the launch phase (75 s hypergravity, comparison: BL-TX hyp-g versus H/W 1 g GC, Table 9 and Table 10, Figure 10). At the end of the following microgravity phase (300 s), 38 probe sets were differentially expressed (µg versus BL-TX hyp-g, Table 9 and Table 10, Figure 10), representing 36% of the differentially regulated probe sets in hypergravity. Interestingly, 30 of the identified probe sets were hypergravity and microgravity double-sensitive (BL-TX hyp-g versus H/W 1 g GC and µg versus BL-TX hyp-g) and were regulated in the opposite direction (Figure 10). The expression fold change (FC) was in the range between +4.69 (up-regulation) and −2.08 (down-regulation), with average values of +1.92 and −1.74, respectively, for the hypergravity-sensitive probe sets and between +2.48 (up-regulation) and −2.35 (down-regulation) with average values of +1.79 and −1.69, respectively, for microgravity-sensitive probe sets (Table 10). Analogous to Jurkat T cells, HIF-1α-dependent gene expression in U937 is more affected in hypergravity than in microgravity. However, in the latter cells the stronger and longer hypergravity phase did not much alter the number of hypergravity sensitive probe sets.

### 2.10. HIF-Related Probe Sets Were Primarily Differentially Regulated in Hypergravity in Human U937 T Cells

**Figure 11:**
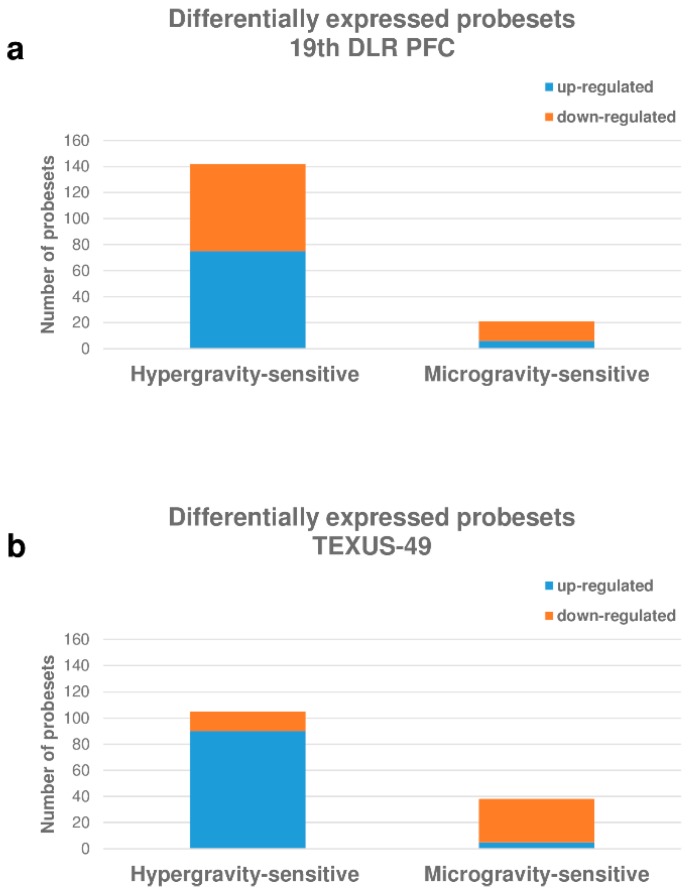
Distribution of differentially expressed probe sets representing HIF-related genes in human U937 cells. (**a**) Hyper- and microgravity-sensitive probe sets (10^−2^–10^−3^ g/1.8 g) identified in the 19th DLR Parabolic Flight Campaign, (**b**) hyper- and microgravity sensitive probe sets (10^−4^ g/5–7 g) identified in the TEXUS-49 suborbital ballistic rocket campaign.

Figure 11 summarizes the number of differentially expressed probe sets in the parabolic flight and suborbital ballistic rocket experiments. Similar fractions of the differentially expressed probe sets were up- and down-regulated after 20 s of hypergravity, while only a small portion of probe sets were differentially expressed after 20 s of microgravity with the majority of probe sets being down-regulated (Figure 11a). Longer periods of hypergravity (75 s 5–7 g) resulted primarily in the up-regulation of probe sets, while after longer periods of microgravity (5 min 10^−4^ g) only a small number of genes remained differentially expressed, mainly down-regulated (Figure 11b).

### 2.11. Dynamic Reaction and Adaptation Processes of Initially Differentially Altered HIF-Related Genes in Altered Gravity in Human U937 Cells

**Figure 12:**
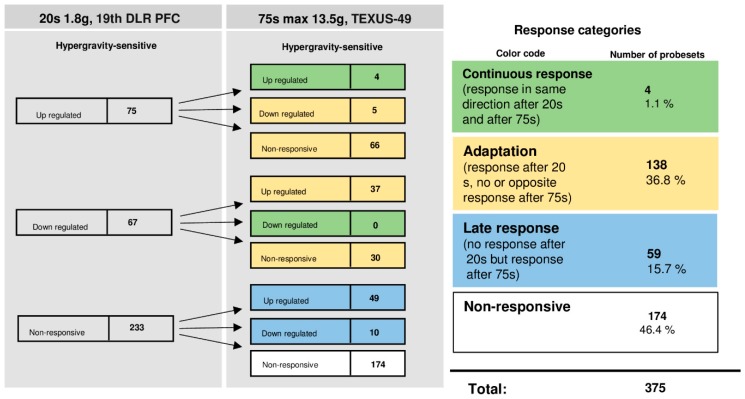
Time course of differential expression of 385 probeset IDs in human U937 cells after hypergravity exposure (list of probe sets in Supplementary Table S1). Human U937 cells were exposed to 20 s and 75 s of hypergravity. The probe sets are grouped according to their regulation after the two exposure times. Continuous response means that the probe sets are either up-regulated or down-regulated at both time points. Adaptation is either the disappearance of the hypergravity-induced effect or regulation in the opposite direction after 75 s. Late response is a regulation that only appears after 75 s. For the abbreviations of the experiment group names see Table 1.

**Figure 13:**
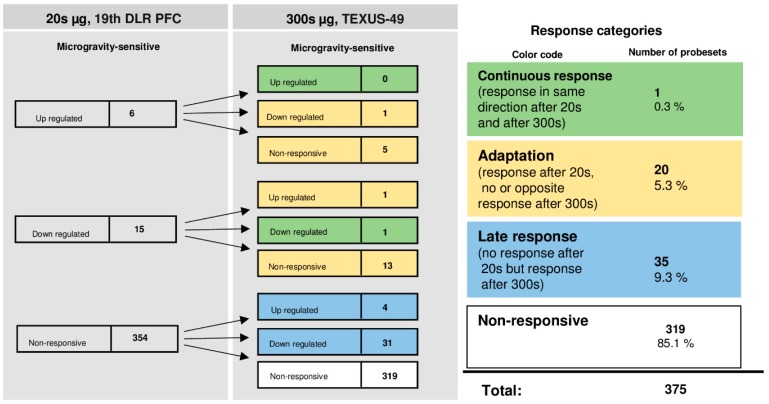
Time course of differential expression of 375 probe sets in human U937 cells after microgravity exposure (list of probe sets in Supplementary Table S1). Human U937 cells were exposed to 20 s and 300 s of microgravity. The probe sets are grouped according to their regulation after the two exposure times. Continuous response means that probe sets are either up-regulated or down-regulated at both time points. Adaption is either the disappearance of the microgravity-induced effect or regulation in the opposite direction after 300 s. A late response is a regulation that only appears after 300 s. Most of the probe sets did not respond to microgravity at either time point. For the abbreviations of the experiment group names see Table 1.

The time course of differentially regulated probe sets of U937 cells in 1.8 g and 13.5 g of hypergravity is summarized in Figure 12, and that of microgravity is summarized in Figure 13. In hypergravity, a total number of 174 probe sets (46.4%) were not altered at all. Out of 142 initially altered probe sets after 20 s in 1.8 g, only four probe sets (1.1%) were altered in the same direction after 75 s in a maximum of 13.5 g. These 4 probe sets, belonging to 3 genes, were differentially regulated in a continuous manner after 20 s and 75 s of hypergravity (Table 11), 38 probe sets (36.8%) adapted. 59 probe sets (15.7%) were altered after 75 s in a maximum of 13.5 g, but not after 20 s of 1.8 g of hypergravity. In contrast to the high HIF-related response of differential gene expression in hypergravity, there was only a minor cellular responsiveness after the microgravity phase. A total number of 319 probe sets (85.1%) were not altered at all. Out of 21 initially altered probe sets after 20 s, one transcript (0.3%) was altered in the same direction after 300 s. This gene is listed in Table 12. The remaining 20 initially differentially expressed probe sets (5.3%) adapted. A further 35 probe sets (9.3%) were altered after 300 s, but not after 20 s of microgravity. Thus, in our experiments performed with U937 cells, conducted in two different experiment platforms (19th DLR parabolic flight campaign andTEXUS-49 suborbital ballistic rocket campaign), we identified in analogy to human Jurkat T cells two pools of altered probe sets: a first one, which responded after seconds and adapted almost completely within 5 min, and a second one, which appeared after at least 5 min of altered gravity.

An inter-platform comparison between the parabolic flight and sounding rocket experiments and between different cell types identified pyruvate dehydrogenase kinase 1 (PDK1) as highly responsive to gravitational changes in human U937 myelomonocytic cells and in Jurkat T cells.

## 3. Discussion

**Figure 14:**
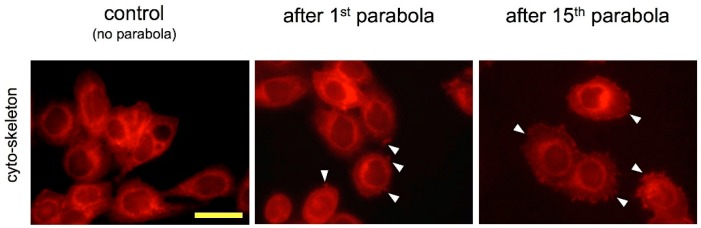
Cytoskeleton visualization in MDA-MB-468 cells of the first Swiss Parabolic Flight Campaign by using phalloidin staining. After the first parabola numerous cells showed single to few protrusions or lamellipodia. After the 15th parabola, almost all cells showed protrusions or lamellipodia allover the entire circumference of the cell border (arrow heads). Scale bar = 20 µm.

In our study we found that compared to normogravitational conditions, HIF-1α and hypoxia-inducible transcripts were differently regulated in early response to altered gravity, whereas HIF-1α-dependent gene expression adapted rapidly to an altered gravitational environment. In our previous studies, we detected profound alterations in the transcriptome after 20 s of microgravity or hypergravity and rapid adaptation responses after 5 min and assumed that gravitational forces are rapidly and constantly transduced into the nucleus [37,38,40]. The response of HIF-related genes to gravitational force changes is more frequent in hypergravity than in microgravity, which corresponds with the genome-wide effects of hypergravity compared to microgravity [37,38]. The question arises how the observed rapid changes in HIF-1α (mRNA and protein) and HIF-dependent gene expression (mRNA) in response to gravitational alterations are possible. Recent data have shown that RNAs are typically distributed inside cells not just by diffusion but are rather by an active, specific and well controlled transport leading to defined enrichment of selected mRNAs in distinct regions of the cell [41]. This is achieved by complex and highly dynamic RNA–protein interactions that start already in the nucleus directly after and even during transcription. Then after export of the mRNAs into the cytoplasm they are specifically protected from degradation by being complexed with another set of proteins or sometimes locally degraded to deplete certain areas of the cell from defined mRNAs [41]. More important is the fact that the well-controlled and specific intracellular transport is highly dependent on the cytoskeleton i.e., the actin network. mRNAs are building large particles with proteins including motor-proteins that ensure active movement on actin fibers to carry the RNAs to pre-defined regions in the cells [41]. Once the RNAs reached their destination they are unloaded and anchored to other specific proteins that aid the RNAs to get in contact with the translation machinery [42,43]. Of note, also proteins are transported actively using cytoskeleton-associated mechanisms back and forth through the cells [44]. This includes also transcription factors that may be synthetized remote in the cell and then transported back to the nucleus, a mechanism involved for transferring information from distant parts of the cell to the nucleus [41] i.e., in neurons from dendrites to the nucleus. Since all these transport events are significantly dependent on the cytoskeleton that is rearranged quite quickly in response to mechanical stress [45], we performed phalloidin staining for visualization of the cytoskeleton in MDA-MB-468 cells subjected to gravitational changes. Indeed, these cells showed alteration of the cell shape and actin fiber pattern already after a single hypergravity–microgravity–cycle (Figure 14). Of note, this effect was even more pronounced in cells subjected to 15 parabolic maneuvers. This observation of altered cytoskeleton might be accompanied by an altered intracellular RNA and protein transport and at least partly explaining why the expression of HIF-1α and its target genes which are so quickly altered in response to gravitational alterations. However, intense additional work is necessary to get a better insight as to the role of the cytoskeleton and intracellular RNA and protein transport in hyper- and / or microgravity.

Theoretical explanations for rapid transcript regulation require rapid force transduction into the nucleus, which is likewise theoretically possible and has been demonstrated for other mechanical forces than gravity [46]. It is possible that gravitational forces are rapidly and constantly transduced into the nucleus, acting as an omnipresent condition in the nuclear and chromatin structure and subsequently resulting in homeostasis of gene expression [37].

Hypoxic conditions during space missions are often caused by extravehicular activities (EVAs). A critical issue of spacesuits required for EVAs is the balance between suit air pressure and oxygen concentration in order to ensure an appropriate oxygen partial pressure (pO_2_ = air pressure × O_2_ concentration in the air) for the astronaut. Whereas high suit pressurization reduces mobility of the astronauts, increased oxygen concentrations that would allow lowering the air pressure increase the risk of flammability. Moreover, a markedly reduced suit pressure compared to the spacecraft’s atmosphere may induce decompression sickness (DCS) that per se might induce tissue hypoxia. To avoid DCS, pre-breathing of 100% oxygen is recommended to wash out nitrogen that is dissolved in the body fluids. The time necessary for pre-breathing pure oxygen in order to considerably reduce the risk of DCS depends on the desired suit pressure and duration for the planned EVA [47] as well as the atmospheric pressure the astronauts experienced in the spacecraft before EVAs [48]. Owing to this, the oxygen concentrations and atmospheric pressures inside the space suit need to be variable ranging from 34% to 100% oxygen and 56.5 to 29.6 kPa, respectively. Although this might avoid hypoxia during EVAs, astronauts may live for at least 12 h at a lowered cabin pressure from its nominal pressure of 101.2 kPa to 70.3 kPa prior to the EVA to reduce considerably pure oxygen pre-breath time, a compromise that puts the astronaut into mild hypoxia, later allowing a suit pressure that is low enough for an acceptable degree of mobility for the planned task during EVA. However, the combination of hypoxic exposure with microgravity as well as reduced atmospheric pressure aggravates the symptoms of hypoxia [49] such as vision impairment, edema of the visual nerve papilla and increased intracranial pressure. This symptom complex is called VIIP-Syndrome (visual impairment/intracranial pressure syndrome) and is considered to be a major risk of manned space flight. Moreover, these symptoms are often associated with headache, nausea, vomiting, sleep disturbance and poor physical performance. The latter combination of symptoms is also typical for acute mountain sickness (AMS) that occurs after rapid ascent to high altitude [50]. It appears that the combination of hypoxia (more prominent in AMS then in the VIIP-syndrome) with microgravity (only in VIIP) aggravates especially altered hemodynamics in the intracranial circulation [49] and disturbance of the cerebrospinal fluid system due to the loss of hydrostatic pressure gradients between the head and the rest of the body [51]. Nevertheless, although the detailed patho-physiology of VIIP and AMS is still not fully understood, a large body of evidence suggests that a dysregulated cellular response to hypoxia is involved [52]. Regarding the symptoms of VIIP and AMS, the oxygen-dependent regulated gene vascular endothelial growth factor (VEGF) is of central interest for the following reasons: first, it is already up-regulated by rather mild tissue hypoxia [53]. Second, initially after its discovery this signaling protein has been formerly termed “vascular permeability factor” because the first step of its action to stimulate the growth of new capillaries (angiogenesis) in hypoxic tissues is to weaken the mechanical interactions between endothelial cells [54,55]. Third, in particular in the brain and other neuronal tissues such as the retina and larger nerves such as the optic nerve this has unfavorable consequences as the disruption of cell-cell-interaction of brain endothelial cells disintegrates the blood brain barrier (BBB). Consequently, extravasation of blood plasma and water occurs leading to the clinical symptom of brain edema and increased intracranial pressure with subsequent impairment of brain function such as nausea or vomiting, vision changes and headache as seen in VIIP and AMS. Finally, HIF-signaling as well as VEGF expression, particularly in immune cells, can be induced independent of hypoxia, e.g., through inflammatory stimuli or growth factors [56,57]. Moreover, it is likely that transcription factors, which are involved in force transduction interact with the HIF pathway within a complex and multi-layered response to gravitational changes. In consequence, regarding the health of astronauts, it has to be taken in account that the function of the immune system is affected by HIF signaling and that this might also be aggravated by other environmental factors inherent to space flights [11,12,13,58,59,60].

In our experiments, we utilized the robust multichip average (RMA) algorithm combined with a quantile normalization of the microarray data, where the background noise is subtracted in order to avoid false positive results based on a low signal and high background. Furthermore, the large number and homogeneity of the applied samples resulted in a low variance within the sample groups and a high sensitivity of the applied method. Arbitrary definition of the cut-offs in gene expression analysis is usually made without underlying biological arguments [61,62,63,64] and, therefore, is not regarded as an adequate test procedure [65,66] because it does not include variance and an accompanying level of confidence [65,67,68]. Thus, we applied two sample *t*-test of the null hypothesis and used the *p*-value for the identification of significantly differentially regulated genes, which is frequently applied in microarray studies [69] and has many benefits in comparison to the fold change method. Despite the fact, that we did not select differentially regulated genes according to their fold change, maximum and minimum values were obtained for Jurkat T cells and U937 cells on parabolic flight and suborbital rocket experiments that were usually above/below +/−1.3 reaching fold change values up to +4.69 and −6.46. Interestingly, we detected that the pyruvate dehydrogenase kinase 1 (PDK1) was constantly and stably up-regulated in hypergravity, in different flight campaigns and in different cell types, underscoring a very high level of evidence. The pyruvate dehydrogenase kinase 1 (PDK1), crucially involved in the activation of T cells as well as macrophages, is one of the four family members of protein kinases that phosphorylate and inactivate pyruvate dehydrogenase (PDH) [70], thereby shunting pyruvate away from the TCA cycle toward glycolysis [71]. PDK1 expression results in the inhibition of pyruvate dehydrogenase a (PDHa) subunit through phosphorylation and is a direct target of HIF-1 [72]. Consistent with this finding, PDK1 is overexpressed in tumors in which HIF-1 is stabilized [73]. Also in our experiments, we could confirm that PDK1 up-regulation was accompanied by HIF-1α upregulation, both induced through an increased gravitational force. In line with this we found also a constant and stable up-regulation of SLC2A3 that is the glucose transporter 3 (GLUT3). Whereas lymphocytes express GLUT1 and GLUT3 in equal amounts, monocytes express markedly more GLUT3. Differentiation of monocytes into macrophages induced significant GLUT 3 and GLUT 5 protein expression [74]. Thus, induction of SLC2A3 could argue for a macrophageal activation in hypergravity. SERPINE1 or plasminogen activator inhibitor-1 (PAI-1) demonstrated a stable hypergravity-induced up-regulation. PAI-1 is the principal inhibitor of tissue and urokinase plasminogen activator (tPA, uPA), and hence an inhibitor of fibrinolysis. PAI-1 has pro-tumorigenic functions through stimulation of angiogenesis and inhibition of apoptosis as well as recruitment and polarization of macrophages to tumors [75]. On the other hand, IL1b was constantly and stable down-regulated in microgravity parallel to the down-regulation of HIF1a. HIF1a-dependent induction of the pro-inflammatory cytokine IL1b can even occur without hypoxia, e.g., by stimulation with lipopolysaccharides (LPS) that can lead to intracellular succinate accumulation [76]. Succinate itself inhibits prolyl-hydrogenases [77] that are crucial for continuous normoxic HIF1a degradation. Thus, increases of intracellular succinate per se or as a result of disturbance of the tricarboxylic acid cycle may induce HIF1a accumulation and subsequent hypoxia-inducible gene activation even in normoxia.

Cells were cultured under standard conditions (air with 5% CO_2_) before the experiments. Although the sealed hardware used for all experiments (custom-made parabolic flight and suborbital rocket mission hardware) did not allow for oxygen and CO_2_ measurements, we do not expect any significant differences in the gas concentrations between the experimental groups due to the very short intervals between the fixation time points: the interval between the fixation of the 1 g, hyper-g and µg group was less than 0.5% of the total time period in the sealed cell culture system for parabolic flight experiments, and less than 1% for suborbital ballistic rocket experiments. Since the experiments were not conducted in hypoxia, we assume that the observed differential gene expression was mainly hypoxia-independent. The regulation of HIF-1a by continuous degradation in the presence of oxygen and stabilization in hypoxia is well investigated. However, there is evidence that HIF-1a is specifically regulated independently of the intracellular oxygen concentration, mostly at the transcriptional, but also at the post-transcriptional (e.g., by microRNAs), as well as at the translational level. Many regulators of HIF-1a under normoxia have been identified, e.g., IL-18, IL-6, TNF-α, LPS, angiotensin II, thrombin, hepatocyte growth factor and glucose [78].

Hypoxia has long been shown to stimulate NF-κB-mediated signaling [79], and several pieces of evidence indicate that both HIF and NF-κB proteins are redox-sensitive crucial regulators of metabolic adaptation [15], regulated by the same oxygen sensors [33]. Transcriptional activation of HIF-1α by IKKβ-responsive NF-κB is a crucial precursor to posttranscriptional stabilization and accumulation of HIF-1α protein [80]. Previous studies have demonstrated that NF-κB signaling pathways are also regulated by altered gravity: Whereby inhibition of nuclear translocation of NF-κB has been observed as the predominant response to simulated microgravity, in either stimulated or non-stimulated Jurkat T cells [81], the Rel/NF-κB pathway in T cell activation was inhibited in long-term micogravity by real microgravity during an International Space Station (ISS) experiment [82]. In our study we detected a short-term regulation of HIF transcripts in the time frame of seconds to minutes [83], that is different from changes in HIF-1α-dependent gene expression that require several hours [15]. Therefore, it is tempting to speculate on the impact of long-term gravitational changes on HIF-dependent transcriptional response, which has not been investigated so far in microgravity. A recent study investigated brains of rats exposed to 28 days of hindlimb unloading / tail-suspension as simulation of microgravity and found HIF-1α, HIF-2α and several downstream targets upregulated as well as signs of increased oxidative stress [84]. The NF-κB pathway is known to play a role in many of the health risks associated with spaceflight and sufficient evidence has been accumulated to indicate that the NF-κB pathway is altered by either microgravity or space radiation [85]. We assume a synergistic relationship between HIF and NF-κB as regulator of immunity in altered gravity conditions. Because of the short half-life, HIF could represent an attractive pharmacological target for counteracting the immune system deterioration during space flights and, therefore, mitigating the risk for exploration class missions.

## 4. Materials and Methods

Material and methods have been described in our previous studies [37,38,40].

### 4.1. Cell Culture

The human cell lines U937 (myelomonocytic cells, ATCC CRL-1593.2, Manassas, VA, USA) and Jurkat (T cells, ATCC Clone E6-1, TIB-152, Manassas, VA, USA) were cultured under standard cell culture conditions as described previously [38], In summary, cells were kept in a standard cell culture incubator (5% CO_2_, 100% humidity, 37 °C), subcultured every second day and reseeded to a density of 0.2 × 10^6^ cells/mL. For quantification and assessment of cell viability cells were stained with trypan blue. Viability was between 97 and 100%. Population doubling time was between 24 and 28 h. MDA-MB-468 breast adenocarcinoma cells (ATCC HTB-13, Manassas, VA, USA) were cultured in Dulbecco’s modified Eagle’s medium (DMEM) containing high glucose levels (4.5 g/L) (Gibco/Life Technologies, Hessen, Germany) and supplemented with 10% Fetal Bovine Serum (10270-106, Gibco/Life Technologies, Hessen, Germany) and 1% penicillin/streptomycin (Gibco/Life Technologies, Hessen, Germany). Cells were cultured of a density of 60–70% and medium exchange was performed every 48 h. The viability of all cells in culture was frequently checked.

### 4.2. Parabolic Flight Experiment Platform

As already delineated [37,38,39,40,81,86,87], parabolic flights represent a suitable platform for the investigation of initial and primary effects of altered gravity in mammalian cells omitting interfering effects of secondary signaling cascades. During a parabola maneuver different gravitational conditions are experienced: 1 g, 1.8 g, and microgravity (μg, 10–2 g to 10–3 g). We designed a custom made parabolic flight hardware that allows cell culture experiments on board the Airbus A300 ZERO-G (F-BUAD) and the Airbus A310 ZERO-G (Reg F-WNOV) [15,27,28,29,30,31,32,33,34,35,36,37,38,39,40,81,84]. The experimental system is made of three units: (1) a storage rack for pre-experiment storage at 36.5 °C; (2) a cooling rack for post-experiment storage at 4 °C; and (3) a working rack for experiment execution and cell culture handling (Supplementary Figure S1). In total, up to 54 individual experiments can be performed during each flight including required control experiments during the 1 g phase before the first parabola. The fluid injection for cell activation, lysis or fixation works automatically and is pre-programmed. However, the exchange of the single-experiment containers was performed manually. This hardware was used to investigate altered gravity based gene expression changes in human U937 and Jurkat T cells during the 19th and 23rd DLR PFCs. Experiments were only conducted during the first parabola to ensure that the detected differential gene expression levels were a result of the effect of gravitational change rather than an accumulated long-term effect.

Regarding the MDA-MB-468 cells we developed smaller autarkic cell-containers (20 × 15 × 6 cm) that contained heating pads to keep the cells warm at 37 °C. Fife of these cell-containers were used at the first Swiss Parabolic Flight Campaign (Dubendorf Military Airport), each equipped with 6 IBIDI-slides (µ-Slide I Luer, IBIDI, Martinsried, Germany) to enable six simultaneous cell culture experiments for each desired condition (Supplementary Figure S2). Changing of the fluids inside the compartment with the cells (cell culture medium, fixing solution) was achieved with two levers allowing two fluid exchanges simultaneously in all six experiments. Two containers (one for immune cytochemistry, one for protein analysis) were used as control, e.g., the experiments were terminated by manual injection of fixing solutions just before the first parabola was initiated. The experiments of the next two containers (one for immune cytochemistry, one for protein analysis) were finished after the first parabola i.e., at the end of the first microgravity phase. The experiments in the remaining container (three slides for immune cytochemistry, three for protein analysis) were terminated after the 15th parabola.

### 4.3. Preparation and Execution of the Parabolic Flight Experiments

The preparation of U937 cells and Jurkat T cells for the parabolic flight experiments have been described previously [37,38,39,40]. Briefly, 1 × 10^7^ U937 cells or Jurkat T cells in 10 mL Medium were filled into 200 mL Nutrimix bags (B. Braun Melsungen, Melsungen, Germany). Cell samples were then transported to the pre-flight preparation laboratories at the NOVESPACE premises in Bordeaux, France, stored overnight at 36.5 °C, and directly used for the experiment the next morning. The pre-flight experiment preparation included the integration of the cells in the Nutrimix bag into a solid plastic container to avoid the risk of fluid spillage in the aircraft in case of leakage of the hardware.

In-flight, the cells were rapidly lysed at the desired time point (1 g IF samples 5 min before the first parabola, 1.8 g and microgravity samples during the first parabola) by fast injection of five volumes of RLT buffer (Qiagen, Hilden, Germany). 1 g ground controls were performed after landing inside the aircraft in the original experiment hardware. Total RNA was isolated from all experiment samples directly after the flight. A schematic overview of the experiment design is shown in Supplementary Figure S1. During the 19th DLR PFC, RNA from 28 U937 cell samples (6 × H/W 1 g GC, 8 × 1 g IF, 6 × BL-PFC hyp-g, 8 × µg, Table 1) was isolated and during the 23rd DLR PFC RNA from 24 Jurkat T cell samples (6 × 1 g ground controls, 6 × 1 g in-flight controls, 6 × 1.8 g and 6 × µg) were purified.

MDA-MB-468 cells were grown in the home laboratory already inside the IBIDI-slides. At the morning of the flight experiment the IBIDI-slides were mounted into pre-warmed (37 °C) cell containers equipped with heating pads and placed in welded plastic bags to prevent possible leakage of fluids in the aircraft. Then they were transported to the airfield in pre-warmed and isolated containers to keep them at 37 °C and mounted into the aircraft. About 40min before the first parabola the medium inside the IBIDI-slides was exchanged and two experimental sets (i.e., six each individual experiments in two containers) were terminated just before the start of the first parabola, two other experimental sets at the end of the first microgravity phase and the last experimental set after the 15th parabola by exchanging the cell culture medium with fixing solution (either 4% formalin solution or TRI-Reagent (R) (Fisher Scientific, Portsmouth, NH, USA)).

### 4.4. RNA Isolation after the Parabolic Flight

The RNA isolation has been described in detail in [37,38,39,40]. Briefly, after the landing of the airplane, the experiment hardware was disassembled, the lysed cell suspension was sheared and applied on an RNA maxi column (Qiagen, Hilden, Germany) with the help of a Qiavac 24 plus vacuum manifold (Qiagen, Hilden, Germany). RNA maxi columns were the centrifuged at 3220× *g* for three min at room temperature and washed with 15 mL of RW1 buffer and two times 10 mL of RPE buffer. Each washing step was followed by centrifugation at 3220× *g* for seven to ten min at room temperature. Total RNA was eluted with 600 µL of pre-warmed RNase-free water (Qiagen, Hilden, Germany) and four min centrifugation at 3220× *g* at room temperature. Extracted RNA was stored and transported on dry ice until RNA processing for microarray analysis.

### 4.5. Western Blotting and Cytoskeleton Visualisation of MDA-MB-468 Cells

Protein extracts of the cells were separated by sodium dodecyl sulfate polyacrylamide gel electrophoresis (SDS-PAGE) (10%) and after being transferred to a nitrocellulose membrane immuno-probed using an anti HIF-1α antibody (1:1000, Novus Biologicals, Centennial, CO, USA). The resulting signal was quantified using an image analyzer (MCID) as described [36] and normalized to the total protein concentration determined from the optical density of the Coomassie Blue stained nitrocellulose membranes as described [88]. Normalized optical densities of the HIF-1α bands were compared using a two-tailed *t*-test. Significant differences were considered at *p* < 0.05.

For phalloidin staining the cells were washed 3 times with PBS containing 0.1 M glycine and 0.1% Triton-X-100 (Sigma-Aldric, St. Louis, MO, USA). Then the cells were incubated in phalloidin solution (phalloidin-rhodamine conjugate, Abcam) for 60 min. Then the cells were rinsed with PBS and observed using a fluorescence microscope (Axio-Imager, Zeiss).

### 4.6. Technologische Experimente unter Schwerelosigkeit (TEXUS)-49 Suborbital Ballistic Rocket Experiment

TEXUS-49 was a suborbital ballistic rocket mission. The vehicle consisted of a two-stage VSB-30 rocket motor and the experiment payload. The rocket was launched on 29 March 2011 at 06:01 from the ESRANGE (European Space and Sounding Rocket Range) Space Center near Kiruna, Sweden. Details of the flight profile included: (1) altitude: 268 km, (2) total microgravity time: 378 s (10^−5^ g), (3) 6.3 g first-stage peak thrust acceleration, (4) 5.03 g mean thrust acceleration, (5) first-stage burnout at 12.3 s, (6) engine separation at 13.6 s, (7) 13.5 g second-stage peak thrust acceleration, (8) 7.3 g mean thrust acceleration, (9) burnout at 43.0 s, (10) yo-yo despin at 56.0s, and (11) engine separation at 59.0 s.

### 4.7. TEXUS-51 Suborbital Ballistic Rocket Experiment

TEXUS-51 was a suborbital ballistic rocket mission. The vehicle consisted of a two-stage VSB-30 rocket motor and the experiment payload. The rocket was launched on 23 April 2015 at 09:35 from the ESRANGE Space Center. Details of the flight profile included: (1) altitude: 258 km, (2) total microgravity time: 369 s (10^−5^ g), (3) 8.1 g first-stage peak thrust acceleration, (4) 5.1 g mean thrust acceleration, (5) first-stage burnout at 12.1 s, (6) engine separation at 13.4 s, (7) 12.6 g second-stage peak thrust acceleration, (8) 6.7 g mean thrust acceleration, (9) burnout at 43.2 s, (10) yo-yo despin at 56.0 s, and (11) engine separation at 59.0 s.

### 4.8. Procedures for the TEXUS Mission

The complete procedures for the TEXUS mission have been described previously in [37,38,39,40]. Briefly, one experimental unit consisted of three syringes. One syringe was filled with the cell suspension (U937 or Jurkat T cells), a second syringe was filled with cell culture medium and a third syringe was filled with the lysis solution (Trizol LS, Life Technologies, Germany). All three syringes were connected via a T-piece and contained small plugs at the outlet ports to avoid premature mixing of fluids. The three syringe-containing experiment units were integrated into temperature-controlled and vacuum-resistant containers (Supplementary Figure S1d). Syringes were activated during the experiment automatically at pre-defined time points via a pneumatic system. The experiment preparation started 7 h before launch and the experimental units were integrated between 1:15 h and 0:45 h before liftoff (late access port). During the experiment, the sample temperature was controlled and set to 36.5 °C ± 0.5 °C until lysis. After landing, recovery of the payload, and disassembly of the experiment units, purification of total RNA immediately started. The isolated RNA was stored and transported on dry ice and kept at −80 °C until processing for microarray analysis.

### 4.9. Experimental Preparation and Integration for TEXUS Experiments

Cells were cultivated on site under standard conditions using the ESRANGE laboratory facilities. The preparation of U937 cells and Jurkat T cells for the TEXUS experiments have been described previously [37,38,39,40]. Briefly, 3 mL plastic syringes were filled with 2.5 × 10^7^ U937 cells or Jurkat T cells shortly before the handover of the samples to the launch team. A second set of syringes was filled with 0.3 mL cell culture medium and a third set of syringes was filled with 1mL of Trizol LS (Life Technologies, Hessen, Germany). The three syringes representing one experimental unit were mounted on a sterilized plastic T-block with a connecting tubing system. Experiment units were stored at 36.5 °C ± 0.5 °C until integration or until execution of the ground controls. During the experiment sequence, first 0.3mL of cell culture medium was injected to the cells and then 1mL of Trizol LS for cell lysis according to the pre-programmed experiment protocol. Altogether, 18 samples were executed during the TEXUS-49 mission: 6 × H/W 1 g GC, 5 × BL-TX hyp-g, 7 × µg (see Table 1). During the TEXUS-51 mission, the in-flight hardware contained additionally and 1 g on-board centrifuge. Furthermore, 1 g cell culture controls were included on the ground. In total, 39 samples were processed for the TEXUS-51 mission: 7 × 1 g ground cell culture controls (CC), 7 × H/W 1 g GC, 9 × 1 g 1 g IF, and 7 × BL-TX hyp-g and 9 × µg.

### 4.10. RNA Isolation After TEXUS Landing

A detailed protocol is given elsewhere [37,38,39,40]. In summary, samples (1.8 mL) were sheared three times by passing them through a 20 G needle (B. Braun Melsungen, Melsungen, Germany), mixed with 0.2 mL chloroform and centrifuged. The upper phase was mixed with 4 mL RLT buffer and 3 mL ethanol and processed using a RNA Midi column (Qiagen, Hilden, Germany). After elution from the column, RNA was transported and stored at −80°C.

### 4.11. RNA Sample Processing and Microarray Data Analysis for U937 Cells

RNA was quality and quantity was assessed with a Nanodrop 1000 (Thermo Scientific). 260/280 nm ratios were between 1.9 and 2.1. An Agilent 2100 Bioanalyzer (Agilent Technologies, Santa Clara, CA, USA) was used to measure the RNA integrity number (RIN) and only RNA samples with values >8.7 were submitted to microarray analysis. For cy3-labeling the “Low RNA Input Linear Amplification Kit, PLUS, One-Color” (Agilent Technologies) was used, and for hybridization (17.5 h) the “Gene Expression Hybridization Kit” (Agilent Technologies, USA) was applied. Washing and scanning was done in the Micro Array Scanner G2505B (Agilent Technologies, USA), imaged files were processed with the NimbleScan Software 2.6 with using default setting of the Robust Multi-Array Analysis (RMA). Averages were made from the normalized microarray data from one experimental group, and ratios between two experimental groups were calculated with Excel 2013. In case the ratio is >1 it equals the fold change, if the ration is <1 the fold change is the negative reciprocal of the ratio. Fold changes were considered to represent a significantly differential expression if the *t*-test *p*-value was <0.05.

### 4.12. RNA Sample Processing and Microarray Data Analysis for Jurkat T Cells

RNA sample processing and microarray data analysis were described in detail elsewhere [37,38,39,40]. In summary the Affymetrix GeneChip^®^ Human Transcriptome Array 2.0 (Affymetrix United Kingdom Ltd., High Wycombe, UK) which contains 44,699 protein coding and 22,829 non-protein coding genes, was used. Only RNA which passed the quality criteria of 260/280 nm ratio between 1.97–2.04 and RNA integrity numbers (RIN) >8.2 was analyzed.

100 ng of total RNA was used as starting material for the preparation of fragmented and biotinylated DNA targets according to the standard Affymetrix WT PLUS Reagent Kit protocol (Affymetrix GeneChip^®^ WT PLUS Reagent Kit, 902,280). The generated DNA targets were hybridized (17 h, 45 °C) onto GeneChip Human Transcriptome Arrays 2.0 and chips were washed and stained according to a standard protocol (Affymetrix GeneChip Wash, Stain and Scan Kit, 900,720) in the Affymetrix Fluidics Station 450 and scanned with the Affymetrix 3000 7 G scanner. The data analysis was performed with the Affymetrix Expression Console and Transcriptome Analysis Console. We used the robust multi-array average (RMA) algorithm combined with a quantile normalization of the microarray data [89]. In this approach, the background noise is subtracted in order to avoid false positive results based on a low signal and high background. A one-way analysis of variance (ANOVA) was used the determine gene expression differences. The log 2 values of the fluorescent intensities for selected genes of interest were back calculated to linear values. The average values were then calculated and fold changes with a *p*-value (one-way ANOVA) of <0.05 were considered to represent a significant differential gene expression. The large number and homogeneity of the applied samples resulted in a low variance within the sample groups and a high sensitivity of the applied method.

### 4.13. Selection of Array Probe sets for Analysis of HIF-1 Signaling Pathway Related Genes

A list of 176 genes relevant for HIF-1 signaling was compiled comprising the 100 genes from Kyoto Encyclopedia of Genes and Genomes (KEGG) database entry hsa04066, “HIF-1 signaling pathway–Homo sapiens (human)” (https://www.genome.jp/dbget-bin/www_bget?pathway+hsa04066) and 76 genes which are known from the literature to also interact with HIF-1 signaling. All probe sets from the NimbleGen expression microarray and all Transcript IDs from the Affymetrix GeneChip^®^ Human Transcriptome Array (for simplicity also referred to as probe sets in this paper) matching in gene symbol and Entrez ID Number (current assignment) were selected. In case the gene symbol and Entrez ID within the annotation of the arrays were different from the current annotation, the probeset was only included if validated by the current annotation. All of the selected probe sets and transcript clusters are shown in Supplementary Table S1.

### 4.14. Intra-Platform and Inter-Platform Comparisons

To elucidate the sensitivity of probe sets to microgravity, intersections of the primary comparison and the respective control-comparison were built. All probe set which were differentially expressed in the same direction in the primary comparison and in the control comparison were not considered to be microgravity-sensitive. Intersections between the pools of gravisensitive probe sets of parabolic flight and of suborbital ballistic rocket flight were made to investigate gravisensitivity over time.

### 4.15. Data Availability

Microarray data sets are accessible under www.ncbi.nlm.nih.gov/projects/geo (Gene Expression Omnibus (GEO) repository), under accession no. GSE94256 for Jurkat T cell and under accession no. GSE101309 for U937 cells.

## Figures and Tables

**Table 1:** Experiment group description of the parabolic flight campaigns (PFC) (19th and 23rd German Aerospace Center (DLR)) and the sounding rocket campaigns (Technologische Experimente unter Schwerelosigkeit (TEXUS) TEXUS-49 and TEXUS-51). Maximum g-level and altered gravity times are given in brackets. BL = Baseline, hyp-g = hypergravity, µg = microgravity, IF = in flight, H/W= hardware, TX = TEXUS.

Experiment Group Description	Gravity Condition	19th DLR PFC 23rd DLR PFC	TEXUS-49 TEXUS-51
Cell culture control	1 g	n/a	CC (TEXUS-51)
Hardware 1 g ground control	1 g	H/W 1 g GC	H/W 1 g GC
1 g in-flight	1 g	1 g IF	1 g IF (TEXUS-51)
Baseline (BL)/Hypergravity [directly before µg phase]	hyp-g	BL-PFC hyp-g [1.8 g, 20 s]	BL-TX hyp-g [max. 13.5 g, 75 s]
Microgravity (µg)	µg	µg [20 s]	µg [300 s]

**Table 2:** Numbers of differentially expressed probe sets in response to altered gravitational conditions in Jurkat T cells during the 23rd DLR PFC. Direct primary comparisons between the sample groups are shown.

Primary Comparisons				
Gene Expression	1 g IF vs. H/W 1 g GC	BL-PFC hyp-g vs. 1 g IF	µg vs. BL-PFC hyp-g	BL-PFC hyp-g vs. H/W 1 g GC
up-regulated	85	19	3	51
down-regulated	29	19	11	19
total	114	38	14	70

**Table 3:** Definition and numbers of hypergravity- and microgravity-sensitive probe sets that are differentially expressed in response to hypergravity or microgravity in Jurkat T cells during parabolic flight.

Gene Expression	Hypergravity-Sensitive Probe Sets			Microgravity-Sensitive Probe Sets		
	Significantly differentially expressed in the primary comparison BL-PFC hyp-g vs. H/W 1 g GC			Significantly differentially expressed in the primary comparison µg vs. BL-PFC hyp-g NOT significantly expressed in the same direction in the primary comparisons µg vs. BL-PFC hyp-g and BL-PFC hyp-g vs. H/W 1 g GC		
	Number	average FC of BL-PFC hyp-g vs. 1 g IF	min/max FC of BL-PFC hyp-g vs. H/W 1 g GC	Number	average FC µg vs. BL-PFC hyp-g	min/max µg vs. BL-PFC hyp-g
up-regulated	51	1.15	1.39	3	1.08	1.11
down-regulated	19	−1.17	−1.62	7	−1.04	−1.07
total	70	-	-	10	-	-

**Table 4:** Numbers of differentially expressed probe sets in response to altered gravity conditions in Jurkat T cells during TEXUS-51 sounding rocket flight. Direct comparisons between the sample groups are shown.

Primary Comparisons						
Gene Expression	1 g IF vs. H/W 1 g GC	BL-TX hyp-g vs. 1 g IF	µg vs. BL-TX hyp-g	µg vs. 1 g IF	BL-TX hyp-g vs. H/W 1 g GC	H/W 1 g GC vs. CC
up-regulated	29	37	5	33	41	125
down-regulated	5	114	4	109	82	52
total	34	151	9	142	123	177

**Table 5:** Identification of hyper- and microgravity-sensitive probe sets representing genes involved in HIF-1 signaling pathway in response to altered gravity conditions during TEXUS-51 sounding rocket flight in Jurkat T cells.

Gene Expression	Hypergravity-Sensitive Probe Sets			Microgravity-Sensitive Probe Sets		
	Significantly differentially expressed in the primary comparison BL-TX hyp-g vs. H/W 1 g GC			Significantly differentially expressed in the primary comparison µg vs. BL-TX hyp-g NOT significantly expressed in the same direction in the primary comparisons µg vs. BL-TX hyp-g and BL-TX hyp-g vs. H/W 1 g GC		
	Number	average fold change (FC) of BL-TX hyp-g vs. H/W 1 g GC	min/max FC of BL-TX hyp-g vs. H/W 1 g GC	Number	average FC µg vs. BL-TX hyp-g	min/max µg vs. BL-TX hyp-g
up-regulated	41	1.26	1.76	5	1.05	1.08
down-regulated	82	−1.14	−1.35	4	−1.10	−1.15
total	123	-	-	9	-	-

**Table 6:** Differentially regulated probe sets in a continuous manner in 20 s and 75 s of hypergravity identified in human Jurkat T cells in the 23rd DLR parabolic flight and TEXUS-51 suborbital ballistic rocket campaigns; 21 probe sets, belonging to 21 genes were differentially regulated with respect to both altered gravity platforms. Plus: significantly up-regulated probe sets (*p*-value < 0.05), minus: significantly down-regulated probe sets (*p*-value < 0.05). FCs are ratios between the averages of linear expression values. If the ratio is <1, FC is calculated as the negative reciprocal of the ratio. For the abbreviations of the group names see Table 1.

Gene Name	Gene ID	Probe Set ID	Fold Change 23rd DLR PFC BL-PFC hyp-g vs. H/W 1 g GC	Fold Change TEXUS-51 BL-TX hyp-g vs. H/W 1 g GC
*SLC2A1*	6513	TC01002578.hg.1	−1.11	−1.03
*PIK3R3; RP11-322N21.2*	8503	TC01002616.hg.1	+1.05	+1.3
*ARNT*	405	TC01003212.hg.1	+1.15	+1.1
*PDK1*	5163	TC02001031.hg.1	+1.07	+1.17
*VHL*	7428	TC03000055.hg.1	+1.07	+1.17
*PIK3CA*	5290	TC03000951.hg.1	+1.18	+1.63
*CAMK2D*	817	TC04001486.hg.1	+1.11	+1.06
*MAPK14*	1432	TC06000523.hg.1	+1.08	+1.14
*IFNGR1*	3459	TC06002152.hg.1	11.13	+1.36
*NOS3; ATG9B*	4846	TC07001009.hg.1	−1.03	−1.12
*RPS6*	6194	TC09000938.hg.1	+1.13	+1.32
*VIM*	7431	TC10000126.hg.1	+1.19	+1.14
*CDKN1B*	1027	TC12000178.hg.1	+1.38	+1.06
*FLT1*	2321	TC13000517.hg.1	+1.12	+1.32
*HIF1A*	3091	TC14002197.hg.1	+1.25	+1.66
*MAP2K1*	5604	TC15000613.hg.1	+1.3	+1.07
*IGF1R*	3480	TC15000949.hg.1	+1.18	+1.08
*PRKCB*	5579	TC16000260.hg.1	+1.06	+1.12
*STAT3*	6774	TC17001531.hg.1	+1.33	+1.26
*AKT2*	208	TC19001532.hg.1	−1.08	−1.09
*PFKL*	5211	TC21000222.hg.1	−1.13	−1.07

**Table 7:** Numbers of differentially expressed probe sets in response to altered gravitational conditions in human U937 cells during parabolic flight. Direct comparisons between the sample groups are shown.

Primary Comparisons				
Gene Expression	1 g IF vs. H/W 1 g GC	BL-PFC hyp-g vs. 1 g IF	µg vs. BL-PFC hyp-g	BL-PFC hyp-g vs. H/W 1 g GC
up-regulated	15	61	6	75
down-regulated	30	43	15	67
total	45	104	21	142

**Table 8:** Definition and numbers of hypergravity- and microgravity-sensitive probe sets that are differentially expressed in response to hypergravity or microgravity in human U937 monocytic cells during parabolic flight.

Gene Expression	Hypergravity-Sensitive Probe Sets			Microgravity-Sensitive Probe Sets		
	Significantly differentially expressed in the primary comparison BL-PFC hyp-g vs. H/W 1 g GC			Significantly differentially expressed in the primary comparison µg vs. BL-PFC hyp-g NOT significantly expressed in the same direction in the primary comparisons µg vs. BL-PFC hyp-g and BL-PFC hyp-g vs. H/W 1 g GC		
	Number	average FC of BL-PFC hyp-g vs. H/W 1 g GC	min/max FC of BL- PFC hyp-g vs. H/W 1 g GC	Number	average FC µg vs. BL- PFC hyp-g	min/max µg vs. BL- PFC hyp-g
up-regulated	75	2.00	3.60	6	1.33	1.82
down-regulated	67	−2.00	−6.46	15	−1.46	−2.09
total	142	-	-	21	-	-

**Table 9:** Numbers of differentially expressed probe sets in response to altered gravity conditions in U937 cells during a suborbital rocket flight. Direct comparisons between the samples groups are shown.

Primary Comparisons			
Gene Expression	µg vs. BL-TX hyp-g	BL-TX hyp-g vs. H/W 1 g GC	µg vs. H/W 1 g GC
up-regulated	5	90	6
down-regulated	33	15	9
total	38	105	15

**Table 10:** Definition and numbers of hypergravity- and microgravity-sensitive probe sets that are differentially expressed in response to hypergravity or microgravity during TEXUS-49 sounding rocket flight in human U937 cells.

Gene Expression	Hypergravity-Sensitive Probe Sets			Microgravity-Sensitive Probe Sets		
.	Significantly differentially expressed in the primary comparison BL-TX hyp-g vs. H/W 1 g GC			Significantly differentially expressed in the primary comparison µg vs. BL-TX hyp-g NOT significantly expressed in the same direction in the primary comparisons µg vs. BL-TX hyp-g and BL-TX hyp-g vs. H/W 1 g GC		
	Number	average FC of BL-TX hyp-g vs. H/W 1 g GC	min/max FC of BL-TX hyp-g vs. H/W 1 g GC	Number	average FC µg vs. BL-TX hyp-g	min/max µg vs. BL-TX hyp-g
up-regulated	90	1.92	4.69	5	1.79	2.48
down-regulated	15	−1.74	−2.08	33	−1.69	−2.35
total	105	-	-	38	-	-

**Table 11:** Differentially regulated probe sets in a continuous manner in 20 s and 75 s of hypergravity identified in human U937 cells in the 19th DLR parabolic flight and TEXUS-49 suborbital ballistic rocket campaigns. Four probe sets, belonging to three genes were differentially regulated with respect to both altered gravity platforms. Plus: significantly up-regulated probe sets (*p*-value < 0.05). FCs are ratios between the averages of linear expression values. If the ratio is <1, FC is calculated as the negative reciprocal of the ratio. For the abbreviations of the group names see Table 1.

Gene Name	Gene ID	Probe Set ID	Fold Change 19th DLR PFC BL-PFC hyp-g vs. H/W 1 g GC	Fold Change TEXUS-49 BL-TX hyp-g vs. H/W 1 g GC
*SERPINE1*	5054	NM_000602	+2.16	+ 1.50
*PDK1*	5163	BC039158	+1.30	+1.48
*PDK1*	5163	NM_002610	+1.23	+1.62
*SLC2A3*	6515	NM_006931	+ 1.81	+1.50

**Table 12:** Differentially regulated probe set in a continuous manner in 20 s and 300 s of microgravity identified in human U937 cells in the 19th DLR parabolic flight and TEXUS-49 suborbital ballistic rocket campaigns. One probe set, belonging to one gene was differentially regulated with respect to both altered gravity platforms. Minus: significantly down-regulated probe set (*p*-value < 0.05). FCs are ratios between the averages of linear expression values. If the ratio is <1, FC is calculated as the negative reciprocal of the ratio. For the abbreviations of the group names see Table 1.

Gene Name	Gene ID	Probe Set ID	Fold Change 19th DLR PFC µg vs. BL-PFC hyp-g	Fold Change TEXUS-49 µg vs. BL-TX hyp-g
*IL1B*	3553	NM_000576	−1.65	−1.43

## Supplementary Materials

Supplementary materials can be found at http://www.mdpi.com/1422-0067/20/2/436/s1.

## Abbreviations

DLR	German Aerospace Center
ESRANGE	European Space and Sounding Rocket Range
FC	fold change
HIF	hypoxia inducible factor
RIN	RNA Integrity Number
TEXUS	Technologische Experimente unter Schwerelosigkeit
