# Supplementary material for: Expression of Hypoxia-Inducible Factor 1α (HIF-1α) and Genes of Related Pathways in Altered Gravity
Source: Int J Mol Sci. 2019 Jan 20;20(2):436. doi: 10.3390/ijms20020436 (PMC6358763; doi:10.3390/ijms20020436)
Supplement: Supplementary file 1 [file ijms-20-00436-s001.pdf]

*Supplementary Material*

# Expression of Hypoxia-Inducible Factor 1$\alpha$ (HIF-1$\alpha$) and Genes of Related Pathways in Altered Gravity

**Johannes Vogel [1,†], Cora Sandra Thiel [2,3,†], Svantje Tauber [2,3], Christian Stockmann [2], Max Gassmann [1,4,*] and Oliver Ullrich [2,3,4,5,*]**

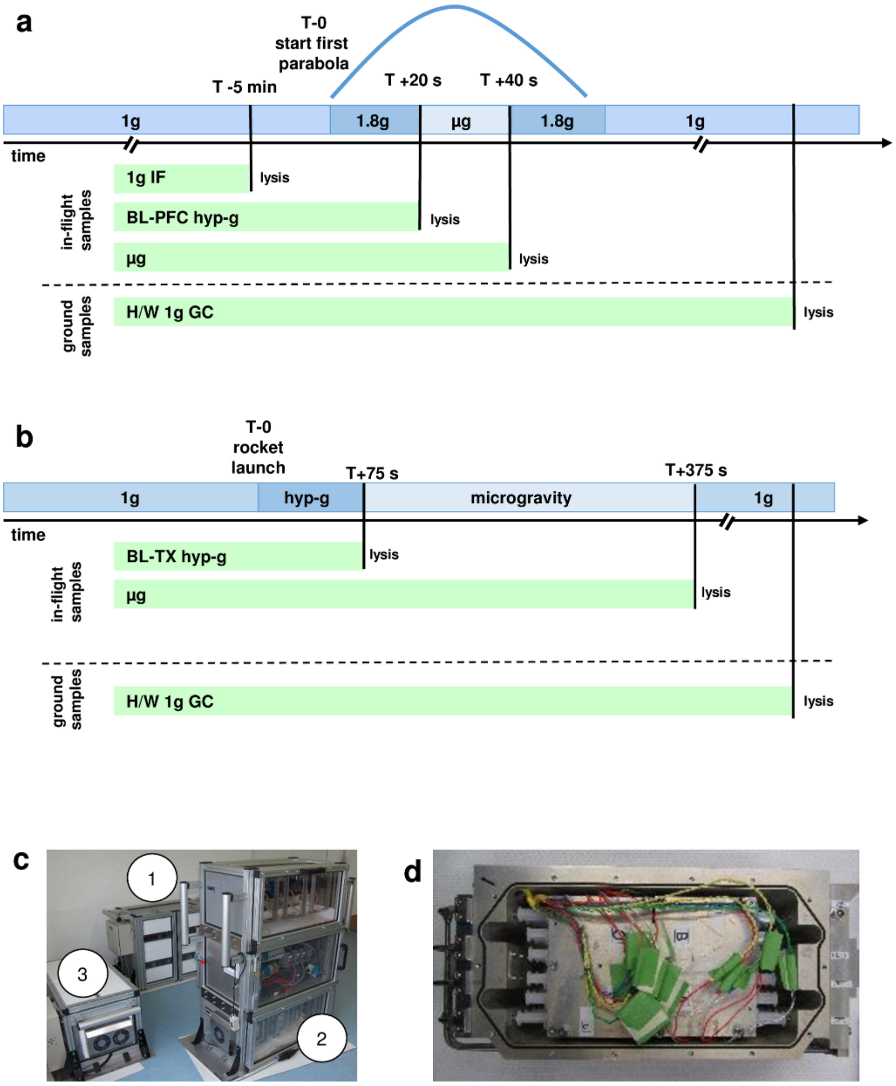


**Supplementary Figure S1.** Experiment design of the parabolic flight (19th and 23rd DLR PFC) and suborbital ballistic rocket (TEXUS-49 and TEXUS-51) experiments. **(a)** During the parabolic flight campaigns, U937 or Jurkat T cells were analyzed before and during the first parabola. 1g in-flight control (1g IF) samples were lysed 5 min before the first parabola, 1.8g hypergravity samples at the end of the first 1.8g phase, and microgravity samples at the end of the first microgravity phase. After the flight, 1g ground controls were performed in the experiment hardware inside the aircraft. **(b)** During the suborbital ballistic rocket missions, U937 and Jurkat T cells were lysed at time point T+75 s to monitor the hypergravity and vibration effects of the rocket launch. Microgravity samples and 1g in-flight reference centrifuge control samples (for the TEXUS-51 mission) were lysed after 415s post-launch. Additionally, hardware 1g ground controls were lysed post-flight approximately 15min after the rocket launch. **(c)** In-flight experiment system for parabolic flights on board the Airbus A300 / A310 ZERO-G. **(c1)** Experiment hardware structure which consists of an incubator rack to store the cell containers at 37°C before the experiment (1), an experiment rack, in which all technical aggregates are accommodated for the execution of the experiment and where the living cells are processed during

altered gravity (2), and a cooling rack to store all cell containers at 4°C after the injection of the lysis solution until landing (3). **(d)** In-flight experiment system for the suborbital ballistic rocket flight of the TEXUS-49 and TEXUS-51 payload. TEXUS consists of a VSB-30 engine (not shown) and of the payload structure. Sets of three sterile syringes were filled with cell suspension, medium, and lysis buffer connected by a T-piece with small plugs at the outlet ports to prevent premature contact of the fluids. The syringe systems are accommodated in tempered and vacuum-resistant containers at the static or centrifuge (TEXUS-51) position.

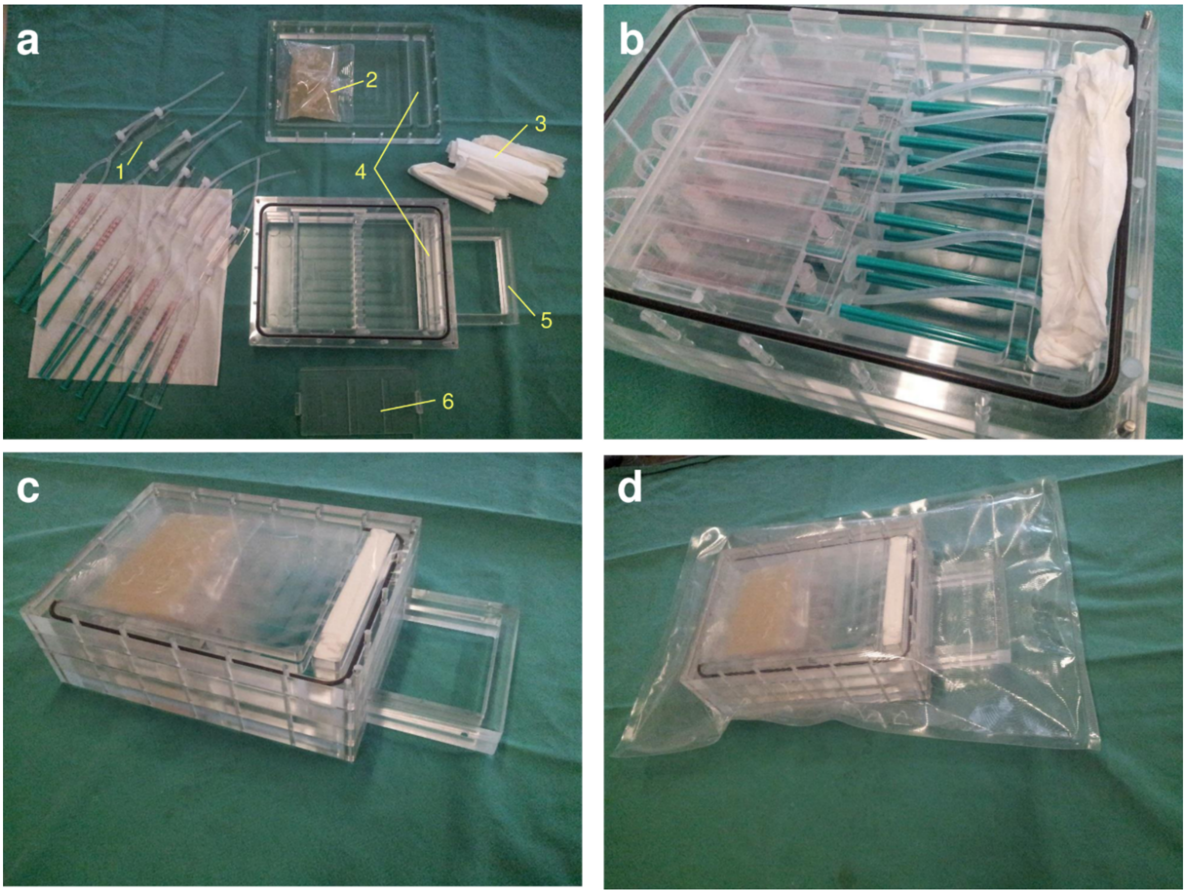


**Supplementary Figure S2.** Cell container designed and used for adherent cells used in the First Swiss Parabolic Flight campaign. (a) Container before packaging. IBIDI-slides containing the cells (1) were incubated for two days to ensure firm adherence of the cells. The inlet of the slides is connected to two syringes one containing cell culture medium (red) and the other fixing solution (colorless). Six sets of these syringe / IBIDI-slide assemblies can be mounted to the container equipped with two release handles (5) that operate six syringes of the same kind simultaneously. The handles have a locking mechanism that ensured that only one handle can be operated at the same time and that only the handle that operates the syringes containing the cell culture medium can be operated first. The outlet of the IBIDI-slides is connected with a tube that directs the waste fluids into a waste compartment (4) that is filled with soaking material (3). Attached to the cover of the container is a gel pad warmed to 37°C (2). (b) All six syringe / IBIDI-slide assemblies fitted into the container, secured in place with a slide holder (6 in (a)) and the outlet tubing guided to the waste compartment filled with the soaking material. (c) Container closed and sealed into a watertight plastic bag (d) as required for security reasons.

**Supplementary Table S1.** List of HIF-related genes. A list of 176 genes relevant for HIF1 signaling was compiled comprising the 100 genes from KEGG database entry hsa04066, "HIF1 signaling pathway - Homo sapiens (human)" (https://www.genome.jp/dbget-bin/www_bget?pathway+hsa04066) and 76 genes which are known from the literature to also interact with HIF1 signaling. These genes are represented by 375 probe sets on the applied NimbleGen arrays based on the hg18 annotation, used for analysis of the human U937 cells. 175 of the 176 genes (one gene was not present on the array) are represented by 184 transcript clusters on the Affymetrix GeneChip® Human Transcriptome Array (Thermo Fisher Scientific, Waltham, MA, USA) used for analysis of the human Jurkat T cells.

| Gene Symbol | Entrez Gene ID | Number of transcript clusters | Transcript Cluster IDs | Number of probe sets | Probe set IDs |
|---|---|---|---|---|---|
| *ADM* | 133 | 1 | TC11000182.hg.1 | 1 | NM_001124 |
| *ADORA2A* | 135 | 1 | TC22001457.hg.1 | 2 | BC013780, NM_000675 |
| *ADRA1B* | 147 | 1 | TC05000889.hg.1 | 1 | NM_000679 |
| *AKT1* | 207 | 1 | TC14001560.hg.1 | 3 | BC084538, BX647722, NM_001014431 |
| *AKT2* | 208 | 1 | TC19001532.hg.1 | 1 | NM_001626 |
| *ALDOA* | 226 | 1 | TC16000338.hg.1 | 3 | CR592372, NM_000034, NM_184041 |
| *ALDOC* | 230 | 1 | TC17001301.hg.1 | 2 | BC106925, NM_005165 |
| *ANGPT1* | 284 | 1 | TC08001521.hg.1 | 2 | BC029406, NM_001146 |
| *ANGPT2* | 285 | 1 | TC08000902.hg.1 | 3 | AF187858, AF218015, NM_001147 |
| *ARG1* | 383 | 1 | TC06000983.hg.1 | 2 | BC020653, NM_000045 |
| *ARNT* | 405 | 1 | TC01003212.hg.1 | 3 | AB209877, BC060838, NM_001668 |
| *BCL2* | 596 | 1 | TC18000554.hg.1 | 3 | BC027258, NM_000633, NM_000657 |
| *CXCR5* | 643 | 1 | TC11001073.hg.1 | 1 | NM_001716 |
| *CA9* | 768 | 1 | TC09000191.hg.1 | 1 | NM_001216 |
| *CAMK2A* | 815 | 1 | TC05001934.hg.1 | 3 | AF145710, AF145711, NM_015981 |
| *CAMK2B* | 816 | 1 | TC07001338.hg.1 | 6 | NM_001220, NM_172078, NM_172080, NM_172081, NM_172082, NM_172084 |
| *CAMK2D* | 817 | 1 | TC04001486.hg.1 | 4 | AB209288, NM_001221, NM_172115, NM_172127 |
| *CAMK2G* | 818 | 1 | TC10001420.hg.1 | 1 | NM_001222 |
| *CAMP* | 820 | 1 | TC03000276.hg.1 | 2 | BC055089, NM_004345 |
| *CD36* | 948 | 1 | TC07000509.hg.1 | 3 | BC008406, NM_000072, NM_001001548 |
| *CDKN1A* | 1026 | 1 | TC06000532.hg.1 | 2 | NM_000389, NM_078467 |
| *CDKN1B* | 1027 | 1 | TC12000178.hg.1 | 1 | NM_004064 |
| *CCR1* | 1230 | 1 | TC03001355.hg.1 | 2 | BC051306, NM_001295 |
| *CCR5* | 1234 | 1 | TC03000256.hg.1 | 2 | BC038398, NM_000579 |
| *CCR7* | 1236 | 1 | TC17001466.hg.1 | 1 | NM_001838 |
| *CREBBP* | 1387 | 1 | TC16000823.hg.1 | 2 | NM_004380, U85962 |
| *MAPK14* | 1432 | 1 | TC06000523.hg.1 | 3 | BC000092, NM_001315, NM_139013 |
| *CTLA4* | 1493 | 1 | TC02001201.hg.1 | 5 | BC069566, BC074842, BC074893, NM_001037631, NM_005214 |
| *CTSD* | 1509 | 1 | TC11003472.hg.1 | 1 | NM_001909 |
| *CYBB* | 1536 | 1 | TC0X000171.hg.1 | 2 | BC032720, NM_000397 |
| *DEFB1* | 1672 | 1 | TC08000906.hg.1 | 2 | BC033298, NM_005218 |

| | | | | | |
|---|---|---|---|---|---|
| *S1PR1* | 1901 | 1 | TC01000909.hg.1 | 1 | NM_001400 |
| *EDN1* | 1906 | 1 | TC06000087.hg.1 | 2 | CR605456, NM_001955 |
| *EGF* | 1950 | 1 | TC04000568.hg.1 | 2 | BC093731, NM_001963 |
| *EGFR* | 1956 | 1 | TC07000328.hg.1 | 6 | BC094761, K03193, NM_005228, NM_201282, NM_201283, NM_201284 |
| *EIF4E* | 1977 | 1 | TC04001403.hg.1 | 3 | BC012611, BC035166, NM_001968 |
| *EIF4EBP1* | 1978 | 1 | TC08000281.hg.1 | 2 | BC058073, NM_004095 |
| *ENG* | 2022 | 1 | TC09001609.hg.1 | 2 | BC014271, NM_000118 |
| *ENO1* | 2023 | 1 | TC01002175.hg.1 | 3 | BC050642, BC073991, NM_001428 |
| *ENO2* | 2026 | 1 | TC12000099.hg.1 | 1 | NM_001975 |
| *ENO3* | 2027 | 1 | TC17000058.hg.1 | 3 | BC017249, NM_001976, NM_053013 |
| *EP300* | 2033 | 1 | TC22000332.hg.1 | 1 | NM_001429 |
| *EPAS1* | 2034 | 1 | TC02000281.hg.1 | 1 | NM_001430 |
| *EPO* | 2056 | 1 | TC07000632.hg.1 | 2 | BC093628, NM_000799 |
| *ERBB2* | 2064 | 1 | TC17000480.hg.1 | 3 | AF177761, AK131568, NM_001005862 |
| *FLT1* | 2321 | 1 | TC13000517.hg.1 | 2 | BC039007, NM_002019 |
| *FN1* | 2335 | 1 | TC02002747.hg.1 | 5 | AB191261, BX538018, CR749317, NM_002026, NM_054034 |
| *MTOR* | 2475 | 1 | TC01002197.hg.1 | 1 | NM_004958 |
| *GAPDH* | 2597 | 1 | TC12000084.hg.1 | 3 | BC001601, BC009081, NM_002046 |
| *GPI* | 2821 | 1 | TC19000442.hg.1 | 2 | BC004982, NM_000175 |
| *MKNK2* | 2872 | 1 | TC19001019.hg.1 | 3 | AF237775, NM_017572, NM_199054 |
| *CXCL2* | 2920 | 1 | TC04001286.hg.1 | 2 | BC015753, NM_002089 |
| *HDAC2* | 3066 | 1 | TC06002035.hg.1 | 1 | NM_001527 |
| *HIF1A* | 3091 | 1 | TC14002197.hg.1 | 2 | NM_001530, NM_181054 |
| *HK1* | 3098 | 1 | TC10000419.hg.1 | 1 | NM_000188 |
| *HK2* | 3099 | 1 | TC02000466.hg.1 | 2 | BC021116, NM_000189 |
| *HK3* | 3101 | 1 | TC05002100.hg.1 | 1 | NM_002115 |
| *HMOX1* | 3162 | 1 | TC22000259.hg.1 | 2 | BC001491, NM_002133 |
| *ICAM1* | 3383 | 1 | TC19000174.hg.1 | 1 | NM_000201 |
| *IFNG* | 3458 | 1 | TC12001696.hg.1 | 1 | NM_000619 |
| *IFNGR1* | 3459 | 1 | TC06002152.hg.1 | 1 | NM_000416 |
| *IFNGR2* | 3460 | 1 | TC21000129.hg.1 | 1 | NM_005534 |
| *IGF1* | 3479 | 1 | TC12001890.hg.1 | 4 | M11568, M29644, M37484, NM_000618 |
| *IGF1R* | 3480 | 2 | TC15000949.hg.1, TC15000951.hg.1 | 1 | NM_000875 |
| *IGF2 (aka GRDF; IGF-II; PP9974; C11orf43)* | 3481 | 0 | - | 2 | BC053318, NM_000612 |
| *IGFBP1* | 3484 | 1 | TC07000294.hg.1 | 2 | NM_000596, NM_001013029 |
| *IGFBP2* | 3485 | 1 | TC02001281.hg.1 | 1 | NM_000597 |
| *IGFBP3* | 3486 | 1 | TC07001355.hg.1 | 2 | BC000013, NM_000598 |
| *IL1B* | 3553 | 1 | TC02002219.hg.1 | 1 | NM_000576 |
| *IL6* | 3569 | 1 | TC07000137.hg.1 | 1 | NM_000600 |

| | | | | | |
|---|---|---|---|---|---|
| *IL6R* | 3570 | 1 | TC01001276.hg.1 | 2 | BC089410, NM_000565 |
| *CXCL8* | 3576 | 1 | TC04000408.hg.1 | 1 | NM_000584 |
| *IL12A* | 3592 | 1 | TC03000870.hg.1 | 2 | BC104982, NM_000882 |
| *TNFRSF9* | 3604 | 1 | TC01002163.hg.1 | 1 | NM_001561 |
| *INS* | 3630 | 1 | TC11001274.hg.1 | 2 | BC005255, NM_000207 |
| *INSR* | 3643 | 1 | TC19001111.hg.1 | 2 | NM_000208, X02160 |
| *ITGAM* | 3684 | 1 | TC16000374.hg.1 | 3 | BC096346, J03925, NM_000632 |
| *ITGB2* | 3689 | 1 | TC21000538.hg.1 | 2 | AK097864, NM_000211 |
| *KDR* | 3791 | 1 | TC04001208.hg.1 | 1 | NM_002253 |
| *KRT14* | 3861 | 1 | TC17001513.hg.1 | 1 | NM_000526 |
| *KRT18* | 3875 | 1 | TC12000424.hg.1 | 4 | BC000180, BC004253, NM_000224, NM_199187 |
| *KRT19* | 3880 | 1 | TC17002906.hg.1 | 2 | BC007628, NM_002276 |
| *LAG3* | 3902 | 1 | TC12000091.hg.1 | 2 | BC052589, NM_002286 |
| *LDHA* | 3939 | 1 | TC11000240.hg.1 | 1 | NM_005566 |
| *LEP* | 3952 | 1 | TC07000768.hg.1 | 3 | BC069323, D49487, NM_000230 |
| *LRP1* | 4035 | 1 | TC12000521.hg.1 | 3 | BC045107, BC052593, NM_002332 |
| *LTBR* | 4055 | 1 | TC12000078.hg.1 | 1 | NM_002342 |
| *MMP2* | 4313 | 1 | TC16000454.hg.1 | 2 | BC002576, NM_004530 |
| *MMP9* | 4318 | 1 | TC20000363.hg.1 | 1 | NM_004994 |
| *MMP14* | 4323 | 1 | TC14000133.hg.1 | 2 | BC064803, NM_004995 |
| *NFKB1* | 4790 | 1 | TC04000526.hg.1 | 3 | BC051765, M58603, NM_003998 |
| *NOS2* | 4843 | 1 | TC17002894.hg.1 | 1 | NM_000625 |
| *NOS3* | 4846 | 1 | TC07001009.hg.1 | 2 | BC069465, NM_000603 |
| *NPPA* | 4878 | 1 | TC01002204.hg.1 | 2 | BC005893, NM_006172 |
| *SERPINE1* | 5054 | 1 | TC07000643.hg.1 | 2 | BC010860, NM_000602 |
| *PDGFB* | 5155 | 1 | TC22000802.hg.1 | 4 | BC029822, BC077725, NM_002608, X83705 |
| *PDHA1* | 5160 | 1 | TC0X000092.hg.1 | 2 | CR614489, NM_000284 |
| *PDHA2* | 5161 | 1 | TC04000504.hg.1 | 1 | NM_005390 |
| *PDHB* | 5162 | 1 | TC03001499.hg.1 | 1 | NM_000925 |
| *PDK1* | 5163 | 1 | TC02001031.hg.1 | 3 | BC039158, DQ234350, NM_002610 |
| *PFKFB3* | 5209 | 1 | TC10000053.hg.1 | 3 | AF056320, AK131307, NM_004566 |
| *PFKL* | 5211 | 1 | TC21000222.hg.1 | 2 | BX537446, NM_001002021 |
| *PGK1* | 5230 | 1 | TC0X000425.hg.1 | 3 | BC023234, BC103752, NM_000291 |
| *ABCB1* | 5243 | 1 | TC07001579.hg.1 | 2 | AF016535, NM_000927 |
| *PIK3CA* | 5290 | 1 | TC03000951.hg.1 | 1 | NM_006218 |
| *PIK3CB* | 5291 | 1 | TC03001824.hg.1 | 2 | CR749357, NM_006219 |
| *PIK3CD* | 5293 | 1 | TC01000118.hg.1 | 2 | NM_005026, U57843 |
| *PIK3R1* | 5295 | 1 | TC05000291.hg.1 | 3 | BC030815, BC094795, NM_181504 |
| *PIK3R2* | 5296 | 1 | TC19002628.hg.1 | 1 | NM_005027 |
| *PKM* | 5315 | 1 | TC15002776.hg.1 | 1 | NM_002654 |
| *PLAUR* | 5329 | 1 | TC19001593.hg.1 | 3 | NM_001005376, NM_001005377, NM_002659 |

| | | | | | |
|---|---|---|---|---|---|
| *PLCG1* | 5335 | 1 | TC20000303.hg.1 | 1 | NM_002660 |
| *PLCG2* | 5336 | 2 | TC16000642.hg.1, TC16000643.hg.1 | 2 | BC007565, NM_002661 |
| *PRKCA* | 5578 | 1 | TC17000783.hg.1 | 2 | BC109273, NM_002737 |
| *PRKCB* | 5579 | 1 | TC16000260.hg.1 | 3 | BC036472, NM_002738, NM_212535 |
| *PRKCG* | 5582 | 1 | TC19000866.hg.1 | 2 | BC047876, NM_002739 |
| *MAPK1* | 5594 | 1 | TC22000547.hg.1 | 4 | BC099905, NM_002745, NM_138957, Z11695 |
| *MAPK3* | 5595 | 1 | TC16001021.hg.1 | 3 | AY033607, NM_001040056, NM_002746 |
| *MAP2K1* | 5604 | 1 | TC15000613.hg.1 | 1 | NM_002755 |
| *MAP2K2* | 5605 | 1 | TC19001058.hg.1 | 1 | NM_030662 |
| *RELA* | 5970 | 1 | TC11001939.hg.1 | 4 | BC011603, BC014095, BC110830, NM_021975 |
| *RORC* | 6097 | 1 | TC01006373.hg.1 | 3 | BC031554, BC110571, NM_001001523 |
| *RPS6* | 6194 | 1 | TC09000938.hg.1 | 1 | NM_001010 |
| *RPS6KB1* | 6198 | 1 | TC17000729.hg.1 | 3 | BC036033, BC053365, NM_003161 |
| *RPS6KB2* | 6199 | 1 | TC11000691.hg.1 | 3 | BC000094, BC006106, NM_001007071 |
| *CCL2* | 6347 | 1 | TC17000383.hg.1 | 1 | NM_002982 |
| *CXCL6* | 6372 | 1 | TC04000409.hg.1 | 1 | NM_002993 |
| *SELL* | 6402 | 1 | TC01003500.hg.1 | 3 | AJ246000, BC020758, NM_000655 |
| *SLC2A1* | 6513 | 1 | TC01002578.hg.1 | 1 | NM_006516 |
| *SLC2A3* | 6515 | 1 | TC12001170.hg.1 | 2 | BC039196, NM_006931 |
| *SLC11A1* | 6556 | 1 | TC02001300.hg.1 | 3 | BC041787, NM_000578, NM_001032220 |
| *STAT3* | 6774 | 1 | TC17001531.hg.1 | 4 | BC000627, BC014482, NM_003150, NM_213662 |
| *STAT4* | 6775 | 1 | TC02002625.hg.1 | 1 | NM_003151 |
| *ELOC* | 6921 | 1 | TC08001332.hg.1 | 4 | BC093065, BC100028, BC100283, NM_005648 |
| *ELOB* | 6923 | 1 | TC16000791.hg.1 | 4 | BC013306, BC065000, NM_007108, NM_207013 |
| *TCF7* | 6932 | 1 | TC05000657.hg.1 | 3 | BC048769, NM_003202, NM_201633 |
| *TEK* | 7010 | 1 | TC09000121.hg.1 | 1 | NM_000459 |
| *TF* | 7018 | 1 | TC03000723.hg.1 | 2 | AK126941, NM_001063 |
| *TFF3* | 7033 | 1 | TC21000484.hg.1 | 2 | BC017859, NM_003226 |
| *TFRC* | 7037 | 1 | TC03002155.hg.1 | 2 | BC001188, NM_003234 |
| *TGFA* | 7039 | 1 | TC02001963.hg.1 | 4 | AF149096, AF149098, BC005308, NM_003236 |
| *TGFB1* | 7040 | 1 | TC19001553.hg.1 | 2 | BC000125, NM_000660 |
| *TGFB3* | 7043 | 1 | TC14001326.hg.1 | 2 | BC018503, NM_003239 |
| *THBS1* | 7057 | 1 | TC15000270.hg.1 | 1 | NM_003246 |
| *TIMP1* | 7076 | 1 | TC0X000238.hg.1 | 2 | BC000866, NM_003254 |
| *TLR4* | 7099 | 1 | TC09000601.hg.1 | 3 | NM_003266, NM_138554, NM_138557 |
| *TNF* | 7124 | 8 | TC06000371.hg.1,<br>TC6_apd_hap1000036.hg.1,<br>TC6_cox_hap2000067.hg.1,<br>TC6_dbb_hap3000058.hg.1,<br>TC6_mann_hap4000059.hg.1, | 1 | NM_000594 |

| | | | | | |
|---|---|---|---|---|---|
| | | | TC6_mcf_hap5000053.hg.1, TC6_qbl_hap6000058.hg.1, TC6_ssto_hap7000054.hg.1 | | |
| *TP* | 7167 | 1 | TC12000096.hg.1 | 4 | BC007086, BC017917, BC070129, NM_000365 |
| *TNFRSF4* | 7293 | 1 | TC01002086.hg.1 | 1 | NM_003327 |
| *VEGFA* | 7422 | 1 | TC06000608.hg.1 | 5 | AF323587, AY263145, M27281, NM_001025366, S85192 |
| *VHL* | 7428 | 1 | TC03000055.hg.1 | 3 | BC058831, L15409, NM_000551 |
| *VIM* | 7431 | 1 | TC10000126.hg.1 | 4 | AK093924, BC030573, BC066956, NM_003380 |
| *CXCR4* | 7852 | 1 | TC02002378.hg.1 | 1 | NM_001008540 |
| *EOMES* | 8320 | 1 | TC03001257.hg.1 | 1 | NM_005442 |
| *CUL2* | 8453 | 1 | TC10001173.hg.1 | 1 | NM_003591 |
| *PIK3R3* | 8503 | 1 | TC01002616.hg.1 | 2 | AF028785, NM_003629 |
| *MKNK1* | 8569 | 1 | TC01006353.hg.1 | 4 | AB000409, AK096423, NM_003684, NM_198973 |
| *TNFRSF18* | 8784 | 1 | TC01002085.hg.1 | 2 | NM_004195, NM_148901 |
| *NRP1* | 8829 | 1 | TC10001166.hg.1 | 5 | AF268691, AF280547, NM_001024628, NM_001024629, NM_003873 |
| *EIF4E2* | 9470 | 1 | TC02001404.hg.1 | 1 | NM_004846 |
| *RBX1* | 9978 | 1 | TC22000327.hg.1 (Entrez ID 63929) | 1 | NM_014248 |
| *AKT3* | 10000 | 1 | TC01004040.hg.1 | 2 | NM_005465, NM_181690 |
| *TBX21* | 30009 | 1 | TC17000613.hg.1 | 1 | NM_013351 |
| *AK3* | 50808 | 1 | TC09000884.hg.1 | 2 | BC013771, NM_016282 |
| *FOXP3* | 50943 | 1 | TC0X001031.hg.1 | 1 | NM_014009 |
| *ANGPT4* | 51378 | 1 | TC20000546.hg.1 | 3 | BC111976, BC111978, NM_015985 |
| *EGLN1* | 54583 | 1 | TC01003968.hg.1 | 2 | BC005369, NM_022051 |
| *HIF1AN* | 55662 | 1 | TC10000724.hg.1 | 3 | AK025680, BC007719, NM_017902 |
| *HAMP* | 57817 | 1 | TC19000464.hg.1 | 1 | NM_021175 |
| *HKDC1* | 80201 | 1 | TC10000418.hg.1 | 3 | AK026414, BC110504, NM_025130 |
| *PROK1* | 84432 | 1 | TC01000966.hg.1 | 1 | NM_032414 |
| *RETNLB* | 84666 | 1 | TC03001633.hg.1 | 2 | BC069318, NM_032579 |
| *EGLN2* | 112398 | 1 | TC19002634.hg.1 | 2 | NM_017555, NM_053046 |
| *EGLN3* | 112399 | 1 | TC14001022.hg.1 | 2 | AK123350, NM_022073 |
| *EIF4E1B* | 253314 | 1 | TC05001004.hg.1 | 3 | XM_171094, XM_927854, XM_932348 |
| *ENO4* | 387712 | 1 | TC10000848.hg.1 | 1 | XM_370577 |

**Supplementary Table S2.** Expression fold changes of 175 of the 176 HIF-related genes (one gene was not present on the microarray).The 175 genes are represented by 184 transcript cluster IDs analyzed in human Jurkat T cells exposed to different gravitational conditions during parabolic flight (23rd DLR PFC). The analysis was made with the Affymetrix GeneChip® Human Transcriptome Array (Thermo Fisher Scientific, Waltham, MA, USA).

| | | | | 1g IF vs H/W 1g GC | | BL-PFC hyp-g vs H/W | | BL-PFC hyp-g vs 1g IF | | µg vs H/W 1g GC | | µg vs 1g IF | | µg vs BL-PFC hyp-g | |
|---|---|---|---|---|---|---|---|---|---|---|---|---|---|---|---|
| **Gene Symbol** | **Entrez ID** | **Transcript Cluster ID** | **Description** | **Fold Change** | **p-value** | **Fold Change** | **p-value** | **Fold Change** | **p-value** | **Fold Change** | **p-value** | **Fold Change** | **p-value** | **Fold Change** | **p-value** |
| *ADM* | 133 | TC11000182.hg.1 | adrenomedullin | 1.2 | 0.0002 | 1.17 | 0.0011 | -1.03 | 0.2901 | 1.12 | 0.0003 | -1.07 | 0.0300 | -1.04 | 0.3310 |
| *ADORA2A* | 135 | TC22001457.hg.1 | adenosine A2a receptor | 1.1 | 0.0049 | 1.02 | 0.2254 | -1.07 | 0.0028 | 1.03 | 0.1775 | -1.07 | 0.0236 | 1.01 | 0.6247 |
| *ADRA1B* | 147 | TC05000889.hg.1 | adrenoceptor alpha 1B; adrenergic, alpha-1B-, receptor | 1.17 | 0.0021 | 1.12 | 0.0000 | -1.05 | 0.2537 | 1.12 | 0.0006 | -1.05 | 0.3225 | 1 | 0.9371 |
| *AKT1* | 207 | TC14001560.hg.1 | v-akt murine thymoma viral oncogene homolog 1 | 1.01 | 0.4663 | 1.03 | 0.3247 | 1.02 | 0.8387 | 1.05 | 0.0372 | 1.03 | 0.4179 | 1.01 | 0.5618 |
| *AKT2* | 208 | TC19001532.hg.1 | v-akt murine thymoma viral oncogene homolog 2 | -1.07 | 0.0043 | -1.08 | 0.0028 | -1.02 | 0.2969 | -1.07 | 0.0035 | -1.01 | 0.7081 | 1.01 | 0.4725 |
| *ALDOA* | 226 | TC16000338.hg.1 | aldolase A, fructose-bisphosphate | -1.03 | 0.6823 | -1 | 0.1359 | 1.02 | 0.0951 | 1.01 | 0.4750 | 1.03 | 0.3075 | 1.01 | 0.3608 |
| *ALDOC* | 230 | TC17001301.hg.1 | aldolase C, fructose-bisphosphate | 1.07 | 0.2721 | 1.06 | 0.1082 | -1 | 0.9758 | 1.01 | 0.8740 | -1.06 | 0.1994 | -1.06 | 0.0464 |
| *ANGPT1* | 284 | TC08001521.hg.1 | angiopoietin 1 | 1.01 | 0.3598 | 1.02 | 0.5641 | 1.01 | 0.8532 | 1.01 | 0.1305 | 1.01 | 0.9456 | -1 | 0.7788 |
| *ANGPT2* | 285 | TC08000902.hg.1 | angiopoietin 2 | -1.01 | 0.7178 | -1.01 | 0.5720 | 1 | 0.8365 | -1.01 | 0.7029 | 1 | 0.9681 | 1 | 0.8840 |
| *ARG1* | 383 | TC06000983.hg.1 | arginase 1; arginase, liver | 1.06 | 0.0129 | 1 | 0.7601 | -1.05 | 0.0352 | 1.01 | 0.5436 | -1.04 | 0.0555 | 1.01 | 0.7812 |
| *ARNT* | 405 | TC01003212.hg.1 | aryl hydrocarbon receptor nuclear translocator | 1.12 | 0.0035 | 1.15 | 0.0007 | 1.03 | 0.2120 | 1.16 | 0.0004 | 1.03 | 0.1182 | 1.01 | 0.7522 |
| *BCL2* | 596 | TC18000554.hg.1 | B-cell CLL/lymphoma 2 | -1.02 | 0.1129 | 1.06 | 0.1522 | 1.08 | 0.0259 | 1.04 | 0.6140 | 1.06 | 0.0603 | -1.02 | 0.2571 |
| *CXCR5* | 643 | TC11001073.hg.1 | chemokine (C-X-C motif) receptor 5 | 1.01 | 0.3770 | 1.02 | 0.4904 | 1 | 0.8263 | 1.02 | 0.1909 | 1.01 | 0.7806 | 1 | 0.5901 |
| *CA9* | 768 | TC09000191.hg.1 | carbonic anhydrase IX | 1.09 | 0.0287 | 1.07 | 0.1624 | -1.02 | 0.1416 | 1.06 | 0.0857 | -1.03 | 0.1925 | -1.01 | 0.6410 |
| *CAMK2A* | 815 | TC05001934.hg.1 | calcium/calmodulin-dependent protein kinase II alpha | 1.1 | 0.0041 | 1.06 | 0.0294 | -1.04 | 0.1218 | 1.07 | 0.0352 | -1.03 | 0.3586 | 1.01 | 0.6634 |
| *CAMK2B* | 816 | TC07001338.hg.1 | calcium/calmodulin-dependent protein kinase II beta | 1.12 | 0.0008 | 1.06 | 0.0320 | -1.05 | 0.0081 | 1.06 | 0.0335 | -1.06 | 0.0274 | -1.01 | 0.7907 |
| *CAMK2D* | 817 | TC04001486.hg.1 | calcium/calmodulin-dependent protein kinase II delta | 1.04 | 0.4922 | 1.11 | 0.0014 | 1.07 | 0.0018 | 1.11 | 0.0019 | 1.07 | 0.0027 | 1 | 0.8087 |
| *CAMK2G* | 818 | TC10001420.hg.1 | calcium/calmodulin-dependent protein kinase II gamma | 1.21 | 0.0000 | 1.25 | 0.0000 | 1.04 | 0.2606 | 1.32 | 0.0000 | 1.09 | 0.0111 | 1.05 | 0.1702 |

| | | | | | | | | | | | | | | | |
|---|---|---|---|---|---|---|---|---|---|---|---|---|---|---|---|
| *CAMP* | 820 | TC03000276.hg.1 | cathelicidin antimicrobial peptide | 1.16 | 0.1007 | 1.09 | 0.3065 | -1.07 | 0.2872 | 1.14 | 0.0852 | -1.02 | 0.9141 | 1.05 | 0.2370 |
| *CD36* | 948 | TC07000509.hg.1 | CD36 molecule (thrombospondin receptor) | 1.03 | 0.3345 | 1.01 | 0.4982 | -1.02 | 0.5298 | -1.01 | 0.5907 | -1.04 | 0.0649 | -1.02 | 0.0168 |
| *CDKN1A* | 1026 | TC06000532.hg.1 | cyclin-dependent kinase inhibitor 1A (p21, Cip1) | 1.23 | 0.0005 | 1.13 | 0.0080 | -1.09 | 0.1116 | 1.12 | 0.0113 | -1.1 | 0.0873 | -1.01 | 0.8698 |
| *CDKN1B* | 1027 | TC12000178.hg.1 | cyclin-dependent kinase inhibitor 1B (p27, Kip1) | 1.31 | 0.0002 | 1.38 | 0.0001 | 1.05 | 0.1619 | 1.32 | 0.0001 | 1.01 | 0.6501 | -1.05 | 0.2833 |
| *CCR1* | 1230 | TC03001355.hg.1 | chemokine (C-C motif) receptor 1 | 1.08 | 0.4411 | 1.01 | 0.8819 | -1.06 | 0.6054 | 1.03 | 0.4602 | -1.05 | 0.9668 | 1.02 | 0.6036 |
| *CCR5* | 1234 | TC03000256.hg.1 | chemokine (C-C motif) receptor 5 (gene/pseudogene); chemokine (C-C motif) receptor 5 | 1.03 | 0.4591 | -1.03 | 0.8664 | -1.05 | 0.5887 | 1 | 0.9656 | -1.02 | 0.5438 | 1.03 | 0.8649 |
| *CCR7* | 1236 | TC17001466.hg.1 | chemokine (C-C motif) receptor 7 | 1.38 | 0.0006 | 1.38 | 0.0032 | -1 | 0.4596 | 1.41 | 0.0007 | 1.02 | 0.9003 | 1.02 | 0.5166 |
| *CREBBP* | 1387 | TC16000823.hg.1 | CREB binding protein | -1.11 | 0.1203 | 1.06 | 0.9619 | 1.19 | 0.1057 | -1 | 0.9670 | 1.11 | 0.0749 | -1.07 | 0.9902 |
| *MAPK14* | 1432 | TC06000523.hg.1 | mitogen-activated protein kinase 14 | -1.01 | 0.5032 | 1.08 | 0.0036 | 1.1 | 0.0033 | 1.04 | 0.0167 | 1.06 | 0.0127 | -1.04 | 0.1836 |
| *CTLA4* | 1493 | TC02001201.hg.1 | cytotoxic T-lymphocyte-associated protein 4 | 1.11 | 0.0121 | 1.01 | 0.2702 | -1.1 | 0.1182 | 1.02 | 0.1587 | -1.09 | 0.0432 | 1 | 0.8190 |
| *CTSD* | 1509 | TC11003472.hg.1 | cathepsin D | 1.05 | 0.3127 | 1.09 | 0.0135 | 1.03 | 0.3450 | 1.05 | 0.0252 | -1 | 0.5980 | -1.03 | 0.5142 |
| *CYBB* | 1536 | TC0X000171.hg.1 | cytochrome b-245, beta polypeptide | 1.05 | 0.1040 | -1.01 | 0.5846 | -1.06 | 0.1996 | -1.07 | 0.5204 | -1.13 | 0.0823 | -1.06 | 0.3244 |
| *DEFB1* | 1672 | TC08000906.hg.1 | defensin, beta 1 | 1.17 | 0.0688 | -1 | 0.5819 | -1.18 | 0.0053 | 1.11 | 0.1351 | -1.06 | 0.5592 | 1.11 | 0.0103 |
| *S1PR1* | 1901 | TC01000909.hg.1 | sphingosine-1-phosphate receptor 1 | -1.2 | 0.0010 | -1.24 | 0.0006 | -1.04 | 0.5051 | -1.21 | 0.0010 | -1.01 | 0.7430 | 1.03 | 0.7403 |
| *EDN1* | 1906 | TC06000087.hg.1 | endothelin 1 | 1.05 | 0.1739 | -1 | 0.8277 | -1.05 | 0.3263 | 1.01 | 0.5196 | -1.04 | 0.3609 | 1.01 | 0.7974 |
| *EGF* | 1950 | TC04000568.hg.1 | epidermal growth factor; epidermal growth factor (beta-urogastrone) | 1.09 | 0.0046 | 1.04 | 0.0153 | -1.05 | 0.2145 | 1.04 | 0.0049 | -1.05 | 0.0532 | -1 | 0.3980 |
| *EGFR* | 1956 | TC07000328.hg.1 | epidermal growth factor receptor; epidermal growth factor receptor (erythroblastic leukemia viral (v-erb-b) oncogene homolog, avian) | 1.06 | 0.0038 | 1.03 | 0.0941 | -1.03 | 0.1489 | 1.05 | 0.0214 | -1.01 | 0.3686 | 1.02 | 0.5089 |
| *EIF4E* | 1977 | TC04001403.hg.1 | eukaryotic translation initiation factor 4E | -1.16 | 0.0001 | -1.09 | 0.0011 | 1.07 | 0.0502 | -1.16 | 0.0000 | 1 | 0.8036 | -1.07 | 0.0204 |

| | | | | | | | | | | | | | | | |
|---|---|---|---|---|---|---|---|---|---|---|---|---|---|---|---|
| *EIF4EBP1* | 1978 | TC08000281.hg.1 | eukaryotic translation initiation factor 4E binding protein 1 | 1.03 | 0.3728 | 1 | 0.6361 | -1.03 | 0.1614 | 1.01 | 0.6902 | -1.03 | 0.1632 | 1 | 0.9059 |
| *ENG* | 2022 | TC09001609.hg.1 | endoglin | 1.06 | 0.0972 | 1.04 | 0.3511 | -1.02 | 0.3191 | 1.04 | 0.1063 | -1.01 | 0.8719 | 1 | 0.3684 |
| *ENO1* | 2023 | TC01002175.hg.1 | enolase 1, (alpha) | -1.06 | 0.0896 | -1.04 | 0.1933 | 1.03 | 0.3663 | -1.11 | 0.0070 | -1.04 | 0.3018 | -1.07 | 0.0208 |
| *ENO2* | 2026 | TC12000099.hg.1 | enolase 2 (gamma, neuronal) | 1.04 | 0.0642 | 1 | 0.8574 | -1.04 | 0.1947 | 1.04 | 0.8659 | 1.01 | 0.2346 | 1.04 | 0.9923 |
| *ENO3* | 2027 | TC17000058.hg.1 | enolase 3 (beta, muscle) | -1.02 | 0.3498 | -1.05 | 0.1518 | -1.03 | 0.3711 | -1.01 | 0.3228 | 1.01 | 0.8981 | 1.04 | 0.4337 |
| *EP300; MIR1281* | 2033 | TC22000332.hg.1 | E1A binding protein p300; microRNA 1281 | -1.1 | 0.1506 | 1.04 | 0.7909 | 1.15 | 0.1051 | 1.05 | 0.4435 | 1.16 | 0.0477 | 1.01 | 0.6150 |
| *EPAS1* | 2034 | TC02000281.hg.1 | endothelial PAS domain protein 1 | 1.39 | 0.0009 | 1.25 | 0.0032 | -1.11 | 0.3535 | 1.39 | 0.0000 | 1 | 0.8181 | 1.12 | 0.1672 |
| *EPO* | 2056 | TC07000632.hg.1 | erythropoietin | 1.1 | 0.0078 | 1.04 | 0.0928 | -1.06 | 0.3066 | 1.08 | 0.0115 | -1.01 | 0.8315 | 1.04 | 0.3997 |
| *ERBB2; MIR4728* | 2064 | TC17000480.hg.1 | v-erb-b2 avian erythroblastic leukemia viral oncogene homolog 2; microRNA 4728; v-erb-b2 erythroblastic leukemia viral oncogene homolog 2, neuro/glioblastoma derived oncogene homolog (avian) | 1.06 | 0.0111 | 1.01 | 0.1791 | -1.04 | 0.1231 | 1.02 | 0.0313 | -1.03 | 0.1071 | 1.01 | 0.7278 |
| *FLT1* | 2321 | TC13000517.hg.1 | fms-related tyrosine kinase 1; fms-related tyrosine kinase 1 (vascular endothelial growth factor/vascular permeability factor receptor) | 1.03 | 0.1129 | 1.12 | 0.0008 | 1.08 | 0.0024 | 1.14 | 0.0068 | 1.1 | 0.0246 | 1.02 | 0.9834 |
| *FN1* | 2335 | TC02002747.hg.1 | fibronectin 1 | 1.04 | 0.0997 | 1.03 | 0.0661 | -1.01 | 0.7894 | 1.03 | 0.1362 | -1.01 | 0.7737 | 1 | 0.9437 |
| *MTOR* | 2475 | TC01002197.hg.1 | mechanistic target of rapamycin (serine/threonine kinase) | -1.2 | 0.0000 | -1.18 | 0.0000 | 1.01 | 0.9830 | -1.18 | 0.0000 | 1.02 | 0.3989 | 1 | 0.5158 |
| *GAPDH* | 2597 | TC12000084.hg.1 | glyceraldehyde-3-phosphate dehydrogenase | 1.03 | 0.2594 | 1.01 | 0.3041 | -1.02 | 0.6786 | -1.01 | 0.5646 | -1.04 | 0.0798 | -1.02 | 0.0482 |
| *GPI* | 2821 | TC19000442.hg.1 | glucose-6-phosphate isomerase | -1.12 | 0.0044 | -1.04 | 0.2414 | 1.08 | 0.0260 | -1.08 | 0.0048 | 1.04 | 0.2837 | -1.03 | 0.0472 |
| *MKNK2* | 2872 | TC19001019.hg.1 | MAP kinase interacting serine/threonine kinase 2 | 1.07 | 0.2851 | 1.12 | 0.2053 | 1.05 | 0.6501 | 1.17 | 0.0152 | 1.09 | 0.0973 | 1.04 | 0.4576 |
| *CXCL2* | 2920 | TC04001286.hg.1 | chemokine (C-X-C motif) ligand 2 | 1.14 | 0.0015 | 1.02 | 0.1332 | -1.12 | 0.0254 | 1.06 | 0.3146 | -1.08 | 0.0512 | 1.03 | 0.9136 |
| *HDAC2* | 3066 | TC06002035.hg.1 | histone deacetylase 2 | -1.09 | 0.0009 | -1.05 | 0.0543 | 1.04 | 0.0702 | -1.1 | 0.0047 | -1 | 0.4139 | -1.05 | 0.2617 |
| *HIF1A* | 3091 | TC14002197.hg.1 | hypoxia inducible factor 1, alpha subunit (basic helix- | 1.12 | 0.0011 | 1.25 | 0.0000 | 1.11 | 0.0016 | 1.21 | 0.0000 | 1.07 | 0.0063 | -1.04 | 0.2370 |

| | | | | | | | | | | | | | |
|---|---|---|---|---|---|---|---|---|---|---|---|---|---|
| | | | loop-helix transcription factor) | | | | | | | | | | |
| *HK1* | 3098 | TC10000419.hg.1 | hexokinase 1 | 1.14 | 0.0019 | 1.14 | 0.0023 | 1 | 0.8576 | 1.13 | 0.0027 | -1 | 0.7820 | -1.01 | 0.6665 |
| *HK2* | 3099 | TC02000466.hg.1 | hexokinase 2 | -1.24 | 0.0011 | -1.16 | 0.0075 | 1.07 | 0.0446 | -1.23 | 0.0008 | 1.01 | 0.6689 | -1.05 | 0.0348 |
| *HK3* | 3101 | TC05002100.hg.1 | hexokinase 3 (white cell) | 1.14 | 0.0010 | 1.07 | 0.1561 | -1.07 | 0.0439 | 1.03 | 0.2688 | -1.1 | 0.0015 | -1.03 | 0.4974 |
| *HMOX1* | 3162 | TC22000259.hg.1 | heme oxygenase (decycling) 1 | 1.12 | 0.0033 | 1.03 | 0.1454 | -1.09 | 0.0175 | 1.03 | 0.1676 | -1.09 | 0.0475 | -1 | 0.8646 |
| *ICAM1* | 3383 | TC19000174.hg.1 | intercellular adhesion molecule 1 | 1.09 | 0.0229 | 1.04 | 0.2392 | -1.04 | 0.1743 | 1.05 | 0.6169 | -1.03 | 0.1221 | 1.01 | 0.6565 |
| *IFNG* | 3458 | TC12001696.hg.1 | interferon, gamma | 1.06 | 0.2721 | -1.01 | 0.7131 | -1.08 | 0.2213 | -1.01 | 0.7969 | -1.07 | 0.2831 | 1.01 | 0.9944 |
| *IFNGR1* | 3459 | TC06002152.hg.1 | interferon gamma receptor 1 | 1.09 | 0.0365 | 1.13 | 0.0145 | 1.04 | 0.5012 | 1.13 | 0.0052 | 1.04 | 0.2236 | -1 | 0.6146 |
| *IFNGR2* | 3460 | TC21000129.hg.1 | interferon gamma receptor 2 (interferon gamma transducer 1) | -1.14 | 0.0050 | -1.06 | 0.0558 | 1.07 | 0.1089 | -1.08 | 0.0071 | 1.06 | 0.5372 | -1.01 | 0.2233 |
| *IGF1* | 3479 | TC12001890.hg.1 | insulin-like growth factor 1 (somatomedin C) | 1.07 | 0.0077 | 1.04 | 0.0558 | -1.02 | 0.3323 | 1.05 | 0.0013 | -1.02 | 0.2491 | 1 | 0.8863 |
| *IGF1R* | 3480 | TC15000949.hg.1 | insulin-like growth factor 1 receptor | 1.15 | 0.0032 | 1.18 | 0.0007 | 1.03 | 0.3974 | 1.19 | 0.0003 | 1.03 | 0.0984 | 1.01 | 0.3233 |
| *IGF1R* | 3480 | TC15000951.hg.1 | insulin-like growth factor 1 receptor | 1.4 | 0.0052 | 1.27 | 0.0013 | -1.11 | 0.4209 | 1.33 | 0.0016 | -1.05 | 0.6223 | 1.05 | 0.6340 |
| *IGFBP1* | 3484 | TC07000294.hg.1 | insulin-like growth factor binding protein 1 | 1.06 | 0.0602 | -1.01 | 0.4156 | -1.07 | 0.0115 | 1.02 | 0.4541 | -1.04 | 0.3511 | 1.03 | 0.1548 |
| *IGFBP2* | 3485 | TC02001281.hg.1 | insulin-like growth factor binding protein 2, 36kDa | 1.06 | 0.4016 | 1.05 | 0.2353 | -1.01 | 0.9097 | 1.08 | 0.0035 | 1.02 | 0.2438 | 1.03 | 0.1914 |
| *IGFBP3* | 3486 | TC07001355.hg.1 | insulin-like growth factor binding protein 3 | 1.09 | 0.0106 | 1.05 | 0.2310 | -1.04 | 0.2409 | 1.05 | 0.0137 | -1.04 | 0.3189 | 1 | 0.5720 |
| *IL1B* | 3553 | TC02002219.hg.1 | interleukin 1, beta | 1.1 | 0.0002 | 1.05 | 0.0920 | -1.05 | 0.0964 | 1.06 | 0.0022 | -1.04 | 0.0115 | 1.01 | 0.6589 |
| *IL6* | 3569 | TC07000137.hg.1 | interleukin 6; interleukin 6 (interferon, beta 2) | -1 | 0.2700 | 1.02 | 0.3954 | 1.02 | 0.7366 | 1.08 | 0.0074 | 1.08 | 0.3754 | 1.05 | 0.1585 |
| *IL6R* | 3570 | TC01001276.hg.1 | interleukin 6 receptor | 1.09 | 0.0007 | 1.02 | 0.2065 | -1.07 | 0.0457 | 1.03 | 0.0912 | -1.06 | 0.0145 | 1.01 | 0.9914 |
| *CXCL8; IL8* | 3576 | TC04000408.hg.1 | chemokine (C-X-C motif) ligand 8; interleukin 8 | 1.07 | 0.2568 | 1.01 | 0.5232 | -1.06 | 0.9258 | -1.02 | 0.7988 | -1.09 | 0.1834 | -1.03 | 0.4352 |
| *IL12A* | 3592 | TC03000870.hg.1 | interleukin 12A; interleukin 12A (natural killer cell stimulatory factor 1, cytotoxic lymphocyte maturation factor 1, p35) | 1.08 | 0.0262 | 1.03 | 0.2637 | -1.05 | 0.0927 | 1.03 | 0.2416 | -1.05 | 0.1680 | -1 | 0.8668 |
| *TNFRSF9* | 3604 | TC01002163.hg.1 | tumor necrosis factor receptor superfamily, member 9 | 1.05 | 0.1626 | 1.04 | 0.2233 | -1.01 | 0.8221 | 1 | 0.7379 | -1.05 | 0.1663 | -1.04 | 0.2420 |

| | | | | | | | | | | | | | | | |
|---|---|---|---|---|---|---|---|---|---|---|---|---|---|---|---|
| *INS; IGF2; INS-IGF2; AC132217.4* | 3630 | TC11001274.hg.1 | insulin; insulin-like growth factor 2 (somatomedin A); INS-IGF2 readthrough; novel transcript, 3'_overlapping_ncRNA IGF2 | 1.09 | 0.0184 | 1.05 | 0.1426 | -1.04 | 0.2095 | 1.05 | 0.1157 | -1.04 | 0.2980 | -1 | 0.8581 |
| *INSR* | 3643 | TC19001111.hg.1 | insulin receptor | 1.16 | 0.0016 | 1.23 | 0.0000 | 1.06 | 0.2563 | 1.22 | 0.0000 | 1.05 | 0.1759 | -1.01 | 0.7062 |
| *ITGAM* | 3684 | TC16000374.hg.1 | integrin, alpha M (complement component 3 receptor 3 subunit) | 1.1 | 0.0081 | 1.05 | 0.1856 | -1.05 | 0.0473 | 1.04 | 0.2960 | -1.06 | 0.0317 | -1.01 | 0.7481 |
| *ITGB2* | 3689 | TC21000538.hg.1 | integrin, beta 2 (complement component 3 receptor 3 and 4 subunit) | -1.01 | 0.7248 | -1.01 | 0.2640 | -1 | 0.5403 | -1.03 | 0.1089 | -1.02 | 0.3187 | -1.02 | 0.6612 |
| *KDR* | 3791 | TC04001208.hg.1 | kinase insert domain receptor (a type III receptor tyrosine kinase) | 1.08 | 0.0060 | 1.02 | 0.2407 | -1.06 | 0.0589 | 1.05 | 0.0338 | -1.03 | 0.1183 | 1.03 | 0.3723 |
| *KRT14* | 3861 | TC17001513.hg.1 | keratin 14 | 1.07 | 0.0279 | 1.01 | 0.5850 | -1.06 | 0.1903 | 1.02 | 0.3483 | -1.05 | 0.1364 | 1.01 | 0.8990 |
| *KRT18* | 3875 | TC12000424.hg.1 | keratin 18 | 1.06 | 0.0080 | -1 | 0.3660 | -1.06 | 0.0315 | 1.06 | 0.0104 | -1.01 | 0.6824 | 1.06 | 0.0464 |
| *KRT19* | 3880 | TC17002906.hg.1 | keratin 19 | 1.07 | 0.0367 | 1.05 | 0.5071 | -1.03 | 0.0526 | 1.04 | 0.5279 | -1.03 | 0.2263 | -1.01 | 0.9016 |
| *LAG3* | 3902 | TC12000091.hg.1 | lymphocyte-activation gene 3 | 1.1 | 0.1856 | 1.02 | 0.8450 | -1.08 | 0.0120 | 1.04 | 0.8764 | -1.06 | 0.1550 | 1.01 | 0.6493 |
| *LDHA* | 3939 | TC11000240.hg.1 | lactate dehydrogenase A | -1.11 | 0.0369 | -1.08 | 0.0632 | 1.02 | 0.5939 | -1.11 | 0.0064 | -1.01 | 0.0996 | -1.03 | 0.0359 |
| *LEP* | 3952 | TC07000768.hg.1 | leptin | 1.09 | 0.0766 | 1.02 | 0.6256 | -1.07 | 0.0181 | 1.09 | 0.0490 | -1 | 0.4357 | 1.07 | 0.0253 |
| *LRP1* | 4035 | TC12000521.hg.1 | low density lipoprotein receptor-related protein 1 | 1.12 | 0.0058 | 1.05 | 0.1830 | -1.06 | 0.1141 | 1.07 | 0.0345 | -1.04 | 0.1843 | 1.02 | 0.6014 |
| *LTBR* | 4055 | TC12000078.hg.1 | lymphotoxin beta receptor (TNFR superfamily, member 3) | 1.11 | 0.0133 | 1.06 | 0.0942 | -1.04 | 0.0772 | 1.07 | 0.0240 | -1.03 | 0.3662 | 1.01 | 0.2074 |
| *MMP2* | 4313 | TC16000454.hg.1 | matrix metallopeptidase 2 (gelatinase A, 72kDa gelatinase, 72kDa type IV collagenase) | 1.1 | 0.0048 | 1.02 | 0.1417 | -1.08 | 0.0866 | 1.05 | 0.0414 | -1.05 | 0.3592 | 1.02 | 0.4423 |
| *MMP9* | 4318 | TC20000363.hg.1 | matrix metallopeptidase 9 (gelatinase B, 92kDa gelatinase, 92kDa type IV collagenase) | 1.15 | 0.0031 | 1.07 | 0.0231 | -1.07 | 0.1740 | 1.09 | 0.0074 | -1.05 | 0.1724 | 1.02 | 0.8470 |
| *MMP14* | 4323 | TC14000133.hg.1 | matrix metallopeptidase 14 (membrane-inserted) | 1.22 | 0.0029 | 1.25 | 0.0035 | 1.03 | 0.8593 | 1.36 | 0.0004 | 1.11 | 0.0725 | 1.08 | 0.1333 |
| *NFKB1* | 4790 | TC04000526.hg.1 | nuclear factor of kappa light polypeptide gene enhancer in B-cells 1 | -1.03 | 0.0496 | -1 | 0.9974 | 1.03 | 0.1628 | -1.04 | 0.2007 | -1.01 | 0.9529 | -1.04 | 0.2887 |

| | | | | | | | | | | | | | | | |
|---|---|---|---|---|---|---|---|---|---|---|---|---|---|---|---|
| *NOS2* | 4843 | TC17002894.hg.1 | nitric oxide synthase 2, inducible | 1.1 | 0.0057 | 1.06 | 0.0100 | -1.03 | 0.2059 | 1.05 | 0.0073 | -1.04 | 0.1989 | -1.01 | 0.9835 |
| *NOS3; ATG9B* | 4846 | TC07001009.hg.1 | nitric oxide synthase 3 (endothelial cell); ATG9 autophagy related 9 homolog B (S. cerevisiae) | 1 | 0.6362 | -1.03 | 0.0272 | -1.03 | 0.0751 | -1.04 | 0.0329 | -1.04 | 0.1164 | -1.01 | 0.4720 |
| *NPPA* | 4878 | TC01002204.hg.1 | natriuretic peptide A; natriuretic peptide precursor A | 1.06 | 0.1827 | -1.02 | 0.6859 | -1.08 | 0.3027 | 1.01 | 0.5639 | -1.04 | 0.1992 | 1.03 | 0.9075 |
| *SERPINE1* | 5054 | TC07000643.hg.1 | serpin peptidase inhibitor, clade E (nexin, plasminogen activator inhibitor type 1), member 1 | 1.17 | 0.0040 | 1.13 | 0.0242 | -1.04 | 0.1251 | 1.14 | 0.0073 | -1.03 | 0.4521 | 1.01 | 0.3308 |
| *PDGFB* | 5155 | TC22000802.hg.1 | platelet-derived growth factor beta polypeptide; platelet-derived growth factor beta polypeptide (simian sarcoma viral (v-sis) oncogene homolog) | 1.09 | 0.0040 | 1.04 | 0.0715 | -1.05 | 0.0704 | 1.04 | 0.1126 | -1.05 | 0.1947 | -1 | 0.8404 |
| *PDHA1* | 5160 | TC0X000092.hg.1 | pyruvate dehydrogenase (lipoamide) alpha 1 | -1.23 | 0.0025 | -1.21 | 0.0007 | 1.02 | 0.6613 | -1.25 | 0.0002 | -1.02 | 0.4954 | -1.04 | 0.0470 |
| *PDHA2* | 5161 | TC04000504.hg.1 | pyruvate dehydrogenase (lipoamide) alpha 2 | 1.03 | 0.0781 | -1.01 | 0.4545 | -1.04 | 0.3579 | -1 | 0.2900 | -1.03 | 0.2898 | 1.01 | 0.9110 |
| *PDHB* | 5162 | TC03001499.hg.1 | pyruvate dehydrogenase (lipoamide) beta | -1.15 | 0.0757 | -1.11 | 0.1940 | 1.03 | 0.4763 | -1.18 | 0.0066 | -1.02 | 0.3977 | -1.06 | 0.0744 |
| *PDK1* | 5163 | TC02001031.hg.1 | pyruvate dehydrogenase kinase, isozyme 1 | 1.06 | 0.0029 | 1.07 | 0.0014 | 1.01 | 0.3570 | 1.07 | 0.0007 | 1.01 | 0.4445 | -1 | 0.6384 |
| *PFKFB3* | 5209 | TC10000053.hg.1 | 6-phosphofructo-2-kinase/fructose-2,6-biphosphatase 3 | 1.07 | 0.0522 | 1.07 | 0.0401 | -1 | 0.6722 | 1.08 | 0.0075 | 1.01 | 0.4115 | 1.01 | 0.7941 |
| *PFKL* | 5211 | TC21000222.hg.1 | phosphofructokinase, liver | -1.07 | 0.0509 | -1.13 | 0.0002 | -1.06 | 0.0061 | -1.12 | 0.0006 | -1.05 | 0.0247 | 1.01 | 0.4674 |
| *PGK1; LOC100652805; LOC100653302* | 5230 | TC0X000425.hg.1 | phosphoglycerate kinase 1; uncharacterized LOC100652805; uncharacterized LOC100653302 | -1.1 | 0.0377 | -1.06 | 0.3276 | 1.04 | 0.0990 | -1.12 | 0.0222 | -1.02 | 0.9552 | -1.06 | 0.0438 |
| *ABCB1* | 5243 | TC07001579.hg.1 | ATP-binding cassette, sub-family B (MDR/TAP), member 1 | 1.06 | 0.0281 | 1.04 | 0.3061 | -1.02 | 0.3168 | 1.01 | 0.5181 | -1.04 | 0.0278 | -1.02 | 0.4819 |
| *PIK3CA* | 5290 | TC03000951.hg.1 | phosphatidylinositol-4,5-bisphosphate 3-kinase, catalytic subunit alpha; | 1.08 | 0.0144 | 1.18 | 0.0000 | 1.09 | 0.0007 | 1.2 | 0.0002 | 1.11 | 0.0085 | 1.02 | 0.9973 |

| | | | | | | | | | | | | | | |
|---|---|---|---|---|---|---|---|---|---|---|---|---|---|---|
| | | | phosphoinositide-3-kinase, catalytic, alpha polypeptide | | | | | | | | | | | |
| *PIK3CB* | 5291 | TC03001824.hg.1 | phosphatidylinositol-4,5-bisphosphate 3-kinase, catalytic subunit beta; phosphoinositide-3-kinase, catalytic, beta polypeptide | -1.12 | 0.0014 | -1.03 | 0.0504 | 1.09 | 0.0124 | -1.02 | 0.1180 | 1.11 | 0.0397 | 1.02 | 0.9331 |
| *PIK3CD* | 5293 | TC01000118.hg.1 | phosphatidylinositol-4,5-bisphosphate 3-kinase, catalytic subunit delta; phosphoinositide-3-kinase, catalytic, delta polypeptide | 1.06 | 0.0037 | 1.03 | 0.0935 | -1.03 | 0.2896 | 1.04 | 0.0306 | -1.01 | 0.1673 | 1.02 | 0.9668 |
| *PIK3R1* | 5295 | TC05000291.hg.1 | phosphoinositide-3-kinase, regulatory subunit 1 (alpha) | -1.16 | 0.0003 | 1.05 | 0.1319 | 1.21 | 0.0003 | 1.01 | 0.8662 | 1.17 | 0.0131 | -1.03 | 0.3982 |
| *PIK3R2* | 5296 | TC19002628.hg.1 | phosphoinositide-3-kinase, regulatory subunit 2 (beta) | 1.1 | 0.0528 | 1.07 | 0.1774 | -1.02 | 0.4858 | 1.12 | 0.0020 | 1.02 | 0.4725 | 1.04 | 0.1096 |
| *PKM* | 5315 | TC15002776.hg.1 | pyruvate kinase, muscle | 1.05 | 0.0269 | 1.08 | 0.0070 | 1.04 | 0.3197 | 1.07 | 0.0002 | 1.02 | 0.2409 | -1.02 | 0.8313 |
| *PLAUR* | 5329 | TC19001593.hg.1 | plasminogen activator, urokinase receptor | 1.1 | 0.0016 | 1.16 | 0.0016 | 1.05 | 0.3185 | 1.12 | 0.0008 | 1.01 | 0.8915 | -1.04 | 0.2478 |
| *PLCG1* | 5335 | TC20000303.hg.1 | phospholipase C, gamma 1 | 1.07 | 0.0246 | 1.14 | 0.0010 | 1.06 | 0.1005 | 1.15 | 0.0001 | 1.07 | 0.0128 | 1.01 | 0.4432 |
| *PLCG2* | 5336 | TC16000642.hg.1 | phospholipase C, gamma 2 (phosphatidylinositol-specific) | 1.07 | 0.2072 | 1.05 | 0.1756 | -1.02 | 0.9361 | 1.04 | 0.0477 | -1.03 | 0.9141 | -1 | 0.9978 |
| *PLCG2* | 5336 | TC16000643.hg.1 | phospholipase C, gamma 2 (phosphatidylinositol-specific) | -1.15 | 0.0047 | -1.2 | 0.0001 | -1.05 | 0.1367 | -1.19 | 0.0005 | -1.04 | 0.2429 | 1.01 | 0.9192 |
| *PRKCA* | 5578 | TC17000783.hg.1 | protein kinase C, alpha | -1.15 | 0.0035 | -1.1 | 0.0268 | 1.05 | 0.2808 | -1.05 | 0.0970 | 1.1 | 0.0180 | 1.05 | 0.1872 |
| *PRKCB* | 5579 | TC16000260.hg.1 | protein kinase C, beta | 1.11 | 0.0001 | 1.06 | 0.0090 | -1.05 | 0.0643 | 1.1 | 0.0008 | -1.01 | 0.4773 | 1.04 | 0.2536 |
| *PRKCG* | 5582 | TC19000866.hg.1 | protein kinase C, gamma | 1.19 | 0.0007 | 1.13 | 0.0403 | -1.05 | 0.0651 | 1.13 | 0.0043 | -1.05 | 0.1109 | -1 | 0.4667 |
| *MAPK1* | 5594 | TC22000547.hg.1 | mitogen-activated protein kinase 1 | -1.03 | 0.8452 | 1.03 | 0.1608 | 1.05 | 0.1010 | 1 | 0.9763 | 1.03 | 0.7915 | -1.03 | 0.0537 |
| *MAPK3* | 5595 | TC16001021.hg.1 | mitogen-activated protein kinase 3 | 1.08 | 0.0258 | 1.05 | 0.2006 | -1.03 | 0.3105 | 1.08 | 0.0170 | -1.01 | 0.8641 | 1.03 | 0.3112 |
| *MAP2K1* | 5604 | TC15000613.hg.1 | mitogen-activated protein kinase kinase 1 | 1.23 | 0.0000 | 1.3 | 0.0000 | 1.06 | 0.0454 | 1.29 | 0.0000 | 1.05 | 0.0395 | -1.01 | 0.8193 |
| *MAP2K2* | 5605 | TC19001058.hg.1 | mitogen-activated protein kinase kinase 2 | 1.07 | 0.0747 | 1.08 | 0.1089 | 1.01 | 0.9078 | 1.11 | 0.0061 | 1.04 | 0.3812 | 1.03 | 0.3464 |
| *RELA* | 5970 | TC11001939.hg.1 | v-rel avian reticuloendotheliosis viral oncogene homolog A; v-rel reticuloendotheliosis viral | -1.07 | 0.0494 | -1.07 | 0.0590 | 1 | 0.8477 | -1.07 | 0.0333 | 1.01 | 0.8109 | 1 | 0.6803 |

| | | | | | | | | | | | | | | | |
|---|---|---|---|---|---|---|---|---|---|---|---|---|---|---|---|
| | | | oncogene homolog A (avian) | | | | | | | | | | | | |
| *RORC* | 6097 | TC01006373.hg.1 | RAR-related orphan receptor C | 1.11 | 0.0004 | 1.11 | 0.0000 | 1.01 | 0.4874 | 1.13 | 0.0000 | 1.02 | 0.2115 | 1.01 | 0.5797 |
| *RPS6* | 6194 | TC09000938.hg.1 | ribosomal protein S6 | 1.05 | 0.1394 | 1.13 | 0.0264 | 1.08 | 0.1996 | 1.05 | 0.2094 | -1 | 0.3904 | -1.08 | 0.0526 |
| *RPS6KB1* | 6198 | TC17000729.hg.1 | ribosomal protein S6 kinase, 70kDa, polypeptide 1 | -1.2 | 0.0000 | -1.15 | 0.0000 | 1.04 | 0.0892 | -1.18 | 0.0000 | 1.02 | 0.4990 | -1.02 | 0.1274 |
| *RPS6KB2* | 6199 | TC11000691.hg.1 | ribosomal protein S6 kinase, 70kDa, polypeptide 2 | 1.07 | 0.1789 | 1.03 | 0.7790 | -1.04 | 0.3016 | 1.06 | 0.2883 | -1.01 | 0.8856 | 1.03 | 0.4279 |
| *CCL2* | 6347 | TC17000383.hg.1 | chemokine (C-C motif) ligand 2 | 1.01 | 0.6685 | 1.08 | 0.0734 | 1.07 | 0.1662 | 1.04 | 0.6008 | 1.03 | 0.8954 | -1.04 | 0.2712 |
| *CXCL6* | 6372 | TC04000409.hg.1 | chemokine (C-X-C motif) ligand 6; chemokine (C-X-C motif) ligand 6 (granulocyte chemotactic protein 2) | 1.09 | 0.0071 | 1.04 | 0.1880 | -1.06 | 0.0464 | 1.05 | 0.0748 | -1.04 | 0.1511 | 1.02 | 0.4960 |
| *SELL* | 6402 | TC01003500.hg.1 | selectin L | -1.44 | 0.0001 | -1.62 | 0.0000 | -1.13 | 0.0528 | -1.72 | 0.0000 | -1.2 | 0.0114 | -1.07 | 0.3195 |
| *SLC2A1* | 6513 | TC01002578.hg.1 | solute carrier family 2 (facilitated glucose transporter), member 1 | -1.16 | 0.0011 | -1.11 | 0.0100 | 1.05 | 0.0231 | -1.17 | 0.0009 | -1.01 | 0.5102 | -1.06 | 0.0126 |
| *SLC2A3* | 6515 | TC12001170.hg.1 | solute carrier family 2 (facilitated glucose transporter), member 3 | 1.24 | 0.0012 | 1.38 | 0.0000 | 1.11 | 0.0216 | 1.34 | 0.0000 | 1.08 | 0.1944 | -1.03 | 0.1175 |
| *SLC11A1* | 6556 | TC02001300.hg.1 | solute carrier family 11 (proton-coupled divalent metal ion transporter), member 1; solute carrier family 11 (proton-coupled divalent metal ion transporters), member 1 | 1.08 | 0.0014 | 1.04 | 0.0301 | -1.04 | 0.0340 | 1.05 | 0.0583 | -1.03 | 0.0791 | 1 | 0.8528 |
| *STAT3* | 6774 | TC17001531.hg.1 | signal transducer and activator of transcription 3 (acute-phase response factor) | 1.25 | 0.0000 | 1.33 | 0.0000 | 1.06 | 0.0488 | 1.31 | 0.0000 | 1.05 | 0.0510 | -1.01 | 0.5966 |
| *STAT4* | 6775 | TC02002625.hg.1 | signal transducer and activator of transcription 4 | 1.18 | 0.0000 | 1.15 | 0.0008 | -1.03 | 0.3524 | 1.19 | 0.0002 | 1.01 | 0.5882 | 1.04 | 0.2525 |
| *TCEB1* | 6921 | TC08001332.hg.1 | transcription elongation factor B (SIII), polypeptide 1 (15kDa, elongin C) | -1.01 | 0.9366 | 1.04 | 0.0981 | 1.04 | 0.0332 | -1.01 | 0.8946 | -1.01 | 0.9297 | -1.05 | 0.0558 |
| *TCEB2* | 6923 | TC16000791.hg.1 | transcription elongation factor B (SIII), polypeptide 2 (18kDa, elongin B) | 1.11 | 0.0081 | 1.08 | 0.0116 | -1.04 | 0.6138 | 1.07 | 0.0457 | -1.04 | 0.7267 | -1 | 0.9741 |

| | | | | | | | | | | | | | | | |
|---|---|---|---|---|---|---|---|---|---|---|---|---|---|---|---|
| *TCF7* | 6932 | TC05000657.hg.1 | transcription factor 7 (T-cell specific, HMG-box) | 1.19 | 0.0428 | 1.3 | 0.0740 | 1.09 | 0.9707 | 1.27 | 0.0023 | 1.07 | 0.1713 | -1.02 | 0.2620 |
| *TEK* | 7010 | TC09000121.hg.1 | TEK tyrosine kinase, endothelial | 1.05 | 0.0110 | -1 | 0.3337 | -1.05 | 0.2763 | 1.01 | 0.5918 | -1.05 | 0.0798 | 1.01 | 0.6379 |
| *TF; TFP1* | 7018 | TC03000723.hg.1 | transferrin; transferrin pseudogene 1 | 1.11 | 0.0035 | 1.03 | 0.1072 | -1.08 | 0.0597 | 1.03 | 0.1755 | -1.08 | 0.0276 | 1.01 | 0.7031 |
| *TFF3* | 7033 | TC21000484.hg.1 | trefoil factor 3 (intestinal) | 1.04 | 0.1176 | 1.01 | 0.5098 | -1.03 | 0.6036 | -1.04 | 0.5879 | -1.08 | 0.1253 | -1.05 | 0.3648 |
| *TFRC* | 7037 | TC03002155.hg.1 | transferrin receptor; transferrin receptor (p90, CD71) | -1.15 | 0.0578 | -1.07 | 0.1338 | 1.08 | 0.3651 | -1.17 | 0.0129 | -1.02 | 0.7328 | -1.09 | 0.1279 |
| *TGFA* | 7039 | TC02001963.hg.1 | transforming growth factor, alpha | 1.09 | 0.0005 | 1.05 | 0.0331 | -1.04 | 0.0535 | 1.07 | 0.0044 | -1.02 | 0.1597 | 1.02 | 0.4619 |
| *TGFB1* | 7040 | TC19001553.hg.1 | transforming growth factor, beta 1 | 1.23 | 0.0360 | 1.39 | 0.0172 | 1.13 | 0.7692 | 1.36 | 0.0001 | 1.1 | 0.2233 | -1.03 | 0.3741 |
| *TGFB3* | 7043 | TC14001326.hg.1 | transforming growth factor, beta 3 | 1.04 | 0.1534 | 1 | 0.7887 | -1.04 | 0.2633 | 1.01 | 0.9130 | -1.04 | 0.2043 | 1 | 0.8804 |
| *THBS1* | 7057 | TC15000270.hg.1 | thrombospondin 1 | 1.07 | 0.0164 | 1.04 | 0.3193 | -1.03 | 0.1516 | 1.03 | 0.0891 | -1.03 | 0.1258 | -1.01 | 0.6711 |
| *TIMP1* | 7076 | TC0X000238.hg.1 | TIMP metallopeptidase inhibitor 1 | 1.19 | 0.0009 | 1.14 | 0.0048 | -1.04 | 0.1679 | 1.11 | 0.0241 | -1.07 | 0.0412 | -1.02 | 0.3417 |
| *TLR4* | 7099 | TC09000601.hg.1 | toll-like receptor 4 | 1.1 | 0.0024 | 1.01 | 0.9914 | -1.09 | 0.0128 | 1.01 | 0.9840 | -1.09 | 0.0127 | -1 | 0.9807 |
| *TNF* | 7124 | TC06000371.hg.1 | tumor necrosis factor; tumor necrosis factor (TNF superfamily, member 2) | 1.14 | 0.0634 | 1.12 | 0.1120 | -1.01 | 0.5901 | 1.15 | 0.0421 | 1.01 | 0.7316 | 1.02 | 0.7579 |
| *TNF* | 7124 | TC6_apd_hap 1000036.hg.1 | tumor necrosis factor; tumor necrosis factor (TNF superfamily, member 2) | 1.11 | 0.1761 | 1.11 | 0.1906 | 1 | 0.7617 | 1.18 | 0.0733 | 1.06 | 0.8036 | 1.06 | 0.4959 |
| *TNF* | 7124 | TC6_cox_hap 2000067.hg.1 | tumor necrosis factor; tumor necrosis factor (TNF superfamily, member 2) | 1.14 | 0.0634 | 1.12 | 0.1120 | -1.01 | 0.5901 | 1.15 | 0.0421 | 1.01 | 0.7316 | 1.02 | 0.7579 |
| *TNF* | 7124 | TC6_dbb_hap 3000058.hg.1 | tumor necrosis factor; tumor necrosis factor (TNF superfamily, member 2) | 1.14 | 0.0634 | 1.12 | 0.1120 | -1.01 | 0.5901 | 1.15 | 0.0421 | 1.01 | 0.7316 | 1.02 | 0.7579 |
| *TNF* | 7124 | TC6_mann_hap 4000059.hg.1 | tumor necrosis factor; tumor necrosis factor (TNF superfamily, member 2) | 1.14 | 0.0634 | 1.12 | 0.1120 | -1.01 | 0.5901 | 1.15 | 0.0421 | 1.01 | 0.7316 | 1.02 | 0.7579 |
| *TNF* | 7124 | TC6_mcf_hap 5000053.hg.1 | tumor necrosis factor; tumor necrosis factor (TNF superfamily, member 2) | 1.14 | 0.0634 | 1.12 | 0.1120 | -1.01 | 0.5901 | 1.15 | 0.0421 | 1.01 | 0.7316 | 1.02 | 0.7579 |
| *TNF* | 7124 | TC6_qbl_hap 6000058.hg.1 | tumor necrosis factor; tumor necrosis factor (TNF superfamily, member 2) | 1.14 | 0.0634 | 1.12 | 0.1120 | -1.01 | 0.5901 | 1.15 | 0.0421 | 1.01 | 0.7316 | 1.02 | 0.7579 |

| | | | | | | | | | | | | | | | |
|---|---|---|---|---|---|---|---|---|---|---|---|---|---|---|---|
| *TNF* | 7124 | TC6_ssto_hap 7000054.hg.1 | tumor necrosis factor; tumor necrosis factor (TNF superfamily, member 2) | 1.14 | 0.0634 | 1.12 | 0.1120 | -1.01 | 0.5901 | 1.15 | 0.0421 | 1.01 | 0.7316 | 1.02 | 0.7579 |
| *TPI1* | 7167 | TC12000096.hg.1 | triosephosphate isomerase 1 | -1.12 | 0.0057 | -1.06 | 0.0630 | 1.05 | 0.1838 | -1.1 | 0.0044 | 1.02 | 0.6600 | -1.03 | 0.2358 |
| *TNFRSF4* | 7293 | TC01002086.hg.1 | tumor necrosis factor receptor superfamily, member 4 | 1.11 | 0.0026 | 1.05 | 0.1001 | -1.06 | 0.0539 | 1.03 | 0.1431 | -1.08 | 0.1731 | -1.02 | 0.8417 |
| *VEGFA* | 7422 | TC06000608.hg.1 | vascular endothelial growth factor A | -1.08 | 0.0014 | -1.11 | 0.0008 | -1.03 | 0.3687 | -1.11 | 0.0004 | -1.02 | 0.2852 | 1 | 0.9026 |
| *VHL* | 7428 | TC03000055.hg.1 | von Hippel-Lindau tumor suppressor, E3 ubiquitin protein ligase; von Hippel-Lindau tumor suppressor | 1.01 | 0.1718 | 1.07 | 0.0006 | 1.06 | 0.0109 | 1.04 | 0.0052 | 1.03 | 0.0709 | -1.02 | 0.4321 |
| *VIM* | 7431 | TC10000126.hg.1 | vimentin | 1.16 | 0.0009 | 1.19 | 0.0017 | 1.03 | 0.7901 | 1.21 | 0.0000 | 1.04 | 0.5095 | 1.02 | 0.7929 |
| *CXCR4* | 7852 | TC02002378.hg.1 | chemokine (C-X-C motif) receptor 4 | -1.47 | 0.0000 | -1.5 | 0.0000 | -1.02 | 0.6746 | -1.49 | 0.0000 | -1.01 | 0.6819 | 1.01 | 0.9757 |
| *EOMES* | 8320 | TC03001257.hg.1 | eomesodermin; eomesodermin homolog (Xenopus laevis) | -1.06 | 0.0160 | -1.12 | 0.0016 | -1.06 | 0.3446 | -1.11 | 0.0056 | -1.05 | 0.4051 | 1.01 | 0.9311 |
| *CUL2* | 8453 | TC10001173.hg.1 | cullin 2 | -1.21 | 0.0001 | -1.05 | 0.0766 | 1.15 | 0.0006 | -1.09 | 0.0312 | 1.11 | 0.0178 | -1.04 | 0.3220 |
| *PIK3R3; RP11-322N21.2* | 8503 | TC01002616.hg.1 | phosphoinositide-3-kinase, regulatory subunit 3 (gamma); novel transcript | -1.08 | 0.0013 | 1.05 | 0.0176 | 1.13 | 0.0001 | -1.01 | 0.4865 | 1.07 | 0.0031 | -1.05 | 0.1705 |
| *MKNK1* | 8569 | TC01006353.hg.1 | MAP kinase interacting serine/threonine kinase 1 | 1.14 | 0.0001 | 1.09 | 0.0436 | -1.04 | 0.2896 | 1.18 | 0.0011 | 1.04 | 0.6019 | 1.08 | 0.2173 |
| *TNFRSF18* | 8784 | TC01002085.hg.1 | tumor necrosis factor receptor superfamily, member 18 | 1.12 | 0.0651 | 1.1 | 0.3785 | -1.02 | 0.2438 | 1.07 | 0.4362 | -1.04 | 0.2179 | -1.03 | 0.9184 |
| *NRP1* | 8829 | TC10001166.hg.1 | neuropilin 1 | 1.15 | 0.0000 | 1.13 | 0.0001 | -1.01 | 0.8616 | 1.15 | 0.0000 | -1 | 0.7753 | 1.01 | 0.9693 |
| *EIF4E2* | 9470 | TC02001404.hg.1 | eukaryotic translation initiation factor 4E family member 2 | -1.15 | 0.0014 | -1.14 | 0.0019 | 1.01 | 0.5509 | -1.14 | 0.0008 | 1 | 0.7654 | -1 | 0.3142 |
| *AKT3* | 10000 | TC01004040.hg.1 | v-akt murine thymoma viral oncogene homolog 3; v-akt murine thymoma viral oncogene homolog 3 (protein kinase B, gamma) | 1.02 | 0.6228 | 1.05 | 0.1930 | 1.03 | 0.6188 | 1.09 | 0.0036 | 1.07 | 0.1564 | 1.03 | 0.2417 |
| *TBX21* | 30009 | TC17000613.hg.1 | T-box 21 | 1.12 | 0.0018 | 1.05 | 0.0116 | -1.07 | 0.1492 | 1.09 | 0.0082 | -1.03 | 0.2405 | 1.03 | 0.7596 |
| *AK3* | 50808 | TC09000884.hg.1 | adenylate kinase 3 | -1.07 | 0.0574 | -1.06 | 0.0258 | 1 | 0.9841 | -1.04 | 0.0692 | 1.02 | 0.6241 | 1.02 | 0.5516 |
| *FOXP3* | 50943 | TC0X001031.hg.1 | forkhead box P3 | 1.09 | 0.0262 | 1.06 | 0.0397 | -1.03 | 0.4270 | 1.05 | 0.0670 | -1.03 | 0.5815 | -1 | 0.8851 |
| *ANGPT4* | 51378 | TC20000546.hg.1 | angiopoietin 4 | 1.1 | 0.0240 | 1.06 | 0.1083 | -1.04 | 0.1252 | 1.05 | 0.1434 | -1.05 | 0.2681 | -1.01 | 0.9846 |

| | | | | | | | | | | | | | | | |
|---|---|---|---|---|---|---|---|---|---|---|---|---|---|---|---|
| *EGLN1* | 54583 | TC01003968.hg.1 | egl-9 family hypoxia-inducible factor 1; egl nine homolog 1 (C. elegans) | -1.24 | 0.0003 | -1.1 | 0.0053 | 1.13 | 0.0083 | -1.09 | 0.0222 | 1.14 | 0.1000 | 1.01 | 0.4745 |
| *HIF1AN* | 55662 | TC10000724.hg.1 | hypoxia inducible factor 1, alpha subunit inhibitor | -1.03 | 0.2697 | 1.01 | 0.3951 | 1.05 | 0.0685 | -1 | 0.9558 | 1.03 | 0.2101 | -1.02 | 0.1830 |
| *HAMP* | 57817 | TC19000464.hg.1 | hepcidin antimicrobial peptide | 1.17 | 0.0006 | 1.04 | 0.4512 | -1.13 | 0.0024 | 1.06 | 0.1961 | -1.11 | 0.0240 | 1.02 | 0.5047 |
| *XPNPEP3; RBX1* | 63929 | TC22000327.hg.1 | X-prolyl aminopeptidase (aminopeptidase P) 3, putative; ring-box 1, E3 ubiquitin protein ligase; ring-box 1 | -1.07 | 0.0273 | -1.03 | 0.3686 | 1.04 | 0.0769 | -1.08 | 0.0219 | -1 | 0.4719 | -1.04 | 0.0814 |
| *HKDC1* | 80201 | TC10000418.hg.1 | hexokinase domain containing 1 | 1.04 | 0.3694 | -1.01 | 0.6689 | -1.05 | 0.7304 | -1.01 | 0.4528 | -1.05 | 0.0548 | 1 | 0.2675 |
| *PROK1* | 84432 | TC01000966.hg.1 | prokineticin 1 | 1.08 | 0.1628 | 1.08 | 0.1714 | 1 | 0.9413 | 1.08 | 0.1089 | 1 | 0.6233 | -1 | 0.5755 |
| *RETNLB* | 84666 | TC03001633.hg.1 | resistin like beta | 1.1 | 0.0291 | 1.05 | 0.0648 | -1.04 | 0.3855 | 1.07 | 0.0442 | -1.03 | 0.5277 | 1.01 | 0.7358 |
| *EGLN2* | 112398 | TC19002634.hg.1 | egl-9 family hypoxia-inducible factor 2 | 1 | 0.9012 | -1.02 | 0.7454 | -1.02 | 0.6541 | -1.01 | 0.9535 | -1.01 | 0.8172 | 1.01 | 0.7497 |
| *EGLN3* | 112399 | TC14001022.hg.1 | egl-9 family hypoxia-inducible factor 3; egl nine homolog 3 (C. elegans) | 1.08 | 0.0023 | 1.06 | 0.1415 | -1.02 | 0.5786 | 1.08 | 0.0073 | 1.01 | 0.5343 | 1.02 | 0.3651 |
| *EIF4E1B* | 253314 | TC05001004.hg.1 | eukaryotic translation initiation factor 4E family member 1B | 1.1 | 0.0049 | 1.05 | 0.0626 | -1.04 | 0.0476 | 1.08 | 0.0402 | -1.02 | 0.2044 | 1.02 | 0.5248 |
| *ENO4* | 387712 | TC10000848.hg.1 | enolase family member 4 | 1.08 | 0.0014 | 1.04 | 0.2461 | -1.04 | 0.0633 | 1.04 | 0.0627 | -1.04 | 0.0638 | 1 | 0.6384 |

**Supplementary Table S3.** Expression fold changes of 175 of the 176 HIF-related genes (one gene was not present on the microarray).The 175 genes are represented by 184 transcript cluster IDs analyzed in human Jurkat T cells exposed to different gravitational conditions during a suborbital ballistic rocket flight (TEXUS-51). The analysis was made with the Affymetrix GeneChip® Human Transcriptome Array (Thermo Fisher Scientific, Waltham, MA, USA).

| | | | | **H/W 1g GC vs CC** | | **BL-TX hyp-g vs H/W 1g GC** | | **1g IF vs BL-TX hyp-g** | | **BL-TX hyp-g vs 1gIF** | | **µg vs 1g IF** | | **1g IF vs H/W 1g GC** | | **µg vs.BL-TX hyp-g** | | **ug vs H/W 1g GC** | |
|---|---|---|---|---|---|---|---|---|---|---|---|---|---|---|---|---|---|---|---|
| **Gene Symbol** | **Entrez ID** | **Transcript Cluster ID** | **Description** | **Fold Change** | **p-value** | **Fold Change** | **p-value** | **Fold Change** | **p-value** | **Fold Change** | **p-value** | **Fold Change** | **p-value** | **Fold Change** | **p-value** | **Fold Change** | **p-value** | **Fold Change** | **p-value** |
| *ADM* | 133 | TC11000182.hg.1 | adrenomedullin | 1.44 | 0.0000 | -1.12 | 0.0274 | 1.18 | 0.0006 | -1.18 | 0.0006 | -1.24 | 0.0003 | 1.05 | 0.2867 | -1.05 | 0.6792 | -1.18 | 0.0140 |
| *ADORA2A* | 135 | TC22001457.hg.1 | adenosine A2a receptor | 1.28 | 0.0000 | -1.17 | 0.0003 | 1.12 | 0.0010 | -1.12 | 0.0010 | -1.1 | 0.0076 | -1.04 | 0.3776 | 1.02 | 0.5504 | -1.14 | 0.0023 |
| *ADRA1B* | 147 | TC05000889.hg.1 | adrenoceptor alpha 1B; adrenergic, alpha-1B-, receptor | 1.44 | 0.0000 | -1.1 | 0.0509 | 1.12 | 0.0121 | -1.12 | 0.0121 | -1.2 | 0.0007 | 1.02 | 0.8496 | -1.08 | 0.1503 | -1.18 | 0.0051 |
| *AKT1* | 207 | TC14001560.hg.1 | v-akt murine thymoma viral oncogene homolog 1 | 1.19 | 0.0000 | 1.08 | 0.0624 | 1.09 | 0.0141 | -1.09 | 0.0141 | -1.07 | 0.0083 | 1.18 | 0.0000 | 1.03 | 0.2167 | 1.11 | 0.0000 |
| *AKT2* | 208 | TC19001532.hg.1 | v-akt murine thymoma viral oncogene homolog 2 | 1.11 | 0.0001 | -1.09 | 0.0008 | 1.07 | 0.0017 | -1.07 | 0.0017 | -1.04 | 0.0135 | -1.02 | 0.9553 | 1.02 | 0.1226 | -1.06 | 0.0058 |
| *ALDOA* | 226 | TC16000338.hg.1 | aldolase A, fructose-bisphosphate | 1.11 | 0.0001 | -1.02 | 0.1710 | 1.06 | 0.0067 | -1.06 | 0.0067 | -1.06 | 0.0445 | 1.04 | 0.0314 | 1 | 0.1606 | -1.02 | 0.8587 |
| *ALDOC* | 230 | TC17001301.hg.1 | aldolase C, fructose-bisphosphate | -1.78 | 0.0000 | -1.05 | 0.1519 | 1.05 | 0.0906 | -1.05 | 0.0906 | 1.04 | 0.9896 | -1 | 0.6856 | 1.09 | 0.0558 | 1.04 | 0.5625 |
| *ANGPT1* | 284 | TC08001521.hg.1 | angiopoietin 1 | 1.14 | 0.0001 | -1.04 | 0.0972 | 1.01 | 0.0738 | -1.01 | 0.0738 | -1.01 | 0.0199 | -1.03 | 0.8689 | -1 | 0.8876 | -1.04 | 0.0320 |
| *ANGPT2* | 285 | TC08000902.hg.1 | angiopoietin 2 | 1.19 | 0.0000 | -1.07 | 0.0038 | 1.08 | 0.0037 | -1.08 | 0.0037 | -1.06 | 0.0091 | 1.01 | 0.5519 | 1.02 | 0.6719 | -1.06 | 0.0225 |
| *ARG1* | 383 | TC06000983.hg.1 | arginase 1; arginase, liver | 1.21 | 0.0001 | -1.07 | 0.0854 | 1.05 | 0.0203 | -1.05 | 0.0203 | -1.05 | 0.0047 | -1.03 | 0.8130 | 1 | 0.7983 | -1.07 | 0.0361 |
| *ARNT* | 405 | TC01003212.hg.1 | aryl hydrocarbon receptor nuclear translocator | -1.33 | 0.0000 | 1.1 | 0.0271 | -1.05 | 0.1282 | 1.05 | 0.1282 | 1.05 | 0.0184 | 1.05 | 0.1302 | -1 | 0.6735 | 1.1 | 0.0036 |
| *BCL2* | 596 | TC18000554.hg.1 | B-cell CLL/lymphoma 2 | -1.66 | 0.0000 | 1.33 | 0.0012 | -1.14 | 0.0018 | 1.14 | 0.0018 | 1.11 | 0.0201 | 1.17 | 0.0301 | -1.03 | 0.6849 | 1.29 | 0.0030 |
| *CXCR5* | 643 | TC11001073.hg.1 | chemokine (C-X-C motif) receptor 5 | 1.37 | 0.0011 | -1.17 | 0.1156 | 1.14 | 0.0116 | -1.14 | 0.0116 | -1.2 | 0.0073 | -1.03 | 0.9068 | -1.05 | 0.5834 | -1.23 | 0.0605 |
| *CA9* | 768 | TC09000191.hg.1 | carbonic anhydrase IX | 1.29 | 0.0002 | -1.17 | 0.0121 | 1.21 | 0.0004 | -1.21 | 0.0004 | -1.25 | 0.0001 | 1.03 | 0.6504 | -1.03 | 0.4887 | -1.21 | 0.0037 |
| *CAMK2A* | 815 | TC05001934.hg.1 | calcium/calmodulin-dependent protein kinase II alpha | 1.29 | 0.0000 | -1.12 | 0.0054 | 1.09 | 0.0002 | -1.09 | 0.0002 | -1.09 | 0.0025 | -1.04 | 0.6777 | -1.01 | 0.6866 | -1.13 | 0.0089 |
| *CAMK2B* | 816 | TC07001338.hg.1 | calcium/calmodulin-dependent protein kinase II beta | 1.34 | 0.0000 | -1.13 | 0.0048 | 1.12 | 0.0000 | -1.12 | 0.0000 | -1.13 | 0.0001 | -1 | 0.9979 | -1 | 0.8750 | -1.13 | 0.0040 |
| *CAMK2D* | 817 | TC04001486.hg.1 | calcium/calmodulin-dependent protein kinase II delta | -1.23 | 0.0000 | 1.06 | 0.0024 | -1.04 | 0.0723 | 1.04 | 0.0723 | 1.03 | 0.2759 | 1.02 | 0.3168 | -1.01 | 0.2969 | 1.05 | 0.0200 |

| | | | | | | | | | | | | | | | | | |
|---|---|---|---|---|---|---|---|---|---|---|---|---|---|---|---|---|---|
| *CAMK2G* | 818 | TC10001420.hg.1 | calcium/calmodulin-dependent protein kinase II gamma | 1.11 | 0.0005 | -1.04 | 0.0608 | -1.02 | 0.2873 | 1.02 | 0.2873 | 1.01 | 0.4377 | -1.06 | 0.0079 | -1.01 | 0.6402 | -1.05 | 0.0157 |
| *CAMP* | 820 | TC03000276.hg.1 | cathelicidin antimicrobial peptide | 1.25 | 0.0001 | -1.13 | 0.0117 | 1.14 | 0.0020 | -1.14 | 0.0020 | -1.14 | 0.0079 | 1 | 0.6077 | -1 | 0.8511 | -1.13 | 0.0389 |
| *CD36* | 948 | TC07000509.hg.1 | CD36 molecule (thrombospondin receptor) | 1.12 | 0.0000 | -1.04 | 0.0742 | 1.04 | 0.0664 | -1.04 | 0.0664 | -1.05 | 0.0441 | 1.01 | 0.8330 | -1 | 0.5733 | -1.04 | 0.0556 |
| *CDKN1A* | 1026 | TC06000532.hg.1 | cyclin-dependent kinase inhibitor 1A (p21, Cip1) | 1.31 | 0.0001 | -1.12 | 0.0515 | 1.14 | 0.0003 | -1.14 | 0.0003 | -1.13 | 0.0002 | 1.02 | 0.4898 | 1.01 | 0.7629 | -1.11 | 0.0298 |
| *CDKN1B* | 1027 | TC12000178.hg.1 | cyclin-dependent kinase inhibitor 1B (p27, Kip1) | -1.05 | 0.0071 | 1.06 | 0.0119 | -1.06 | 0.0334 | 1.06 | 0.0334 | 1.05 | 0.0906 | -1.01 | 0.9637 | -1.01 | 0.7870 | 1.04 | 0.0856 |
| *CCR1* | 1230 | TC03001355.hg.1 | chemokine (C-C motif) receptor 1 | 1.08 | 0.0154 | -1.01 | 0.8140 | 1.07 | 0.4070 | -1.07 | 0.4070 | -1.08 | 0.3369 | 1.06 | 0.3489 | -1.01 | 0.8635 | -1.02 | 0.9466 |
| *CCR5* | 1234 | TC03000256.hg.1 | chemokine (C-C motif) receptor 5 (gene/pseudogene); chemokine (C-C motif) receptor 5 | 1.22 | 0.0001 | -1.09 | 0.0220 | 1.11 | 0.0232 | -1.11 | 0.0232 | -1.09 | 0.0915 | 1.02 | 0.8741 | 1.02 | 0.5482 | -1.07 | 0.0886 |
| *CCR7* | 1236 | TC17001466.hg.1 | chemokine (C-C motif) receptor 7 | 1.14 | 0.0116 | -1.02 | 0.8843 | 1.07 | 0.1872 | -1.07 | 0.1872 | -1.22 | 0.0012 | 1.05 | 0.1809 | -1.15 | 0.0197 | -1.17 | 0.0044 |
| *CREBBP* | 1387 | TC16000823.hg.1 | CREB binding protein | -1.43 | 0.0000 | 1.44 | 0.0000 | -1.17 | 0.0010 | 1.17 | 0.0010 | 1.18 | 0.0008 | 1.23 | 0.0001 | 1.01 | 0.9219 | 1.45 | 0.0000 |
| *MAPK14* | 1432 | TC06000523.hg.1 | mitogen-activated protein kinase 14 | -1.81 | 0.0000 | 1.14 | 0.0029 | -1.15 | 0.0044 | 1.15 | 0.0044 | 1.12 | 0.0171 | -1.01 | 0.4962 | -1.03 | 0.6196 | 1.11 | 0.0093 |
| *CTLA4* | 1493 | TC02001201.hg.1 | cytotoxic T-lymphocyte-associated protein 4 | 1.26 | 0.0000 | -1.1 | 0.0199 | 1.11 | 0.0064 | -1.11 | 0.0064 | -1.11 | 0.0072 | 1.01 | 0.6480 | -1 | 0.6788 | -1.1 | 0.0258 |
| *CTSD* | 1509 | TC11003472.hg.1 | cathepsin D | 1.27 | 0.0000 | -1.11 | 0.0011 | 1.1 | 0.0158 | -1.1 | 0.0158 | -1.17 | 0.0452 | -1.01 | 0.9363 | -1.07 | 0.8782 | -1.19 | 0.0338 |
| *CYBB* | 1536 | TC0X000171.hg.1 | cytochrome b-245, beta polypeptide | 1.15 | 0.0006 | -1.04 | 0.5299 | 1.04 | 0.0444 | -1.04 | 0.0444 | -1.04 | 0.0054 | 1 | 0.3502 | 1 | 0.6708 | -1.04 | 0.3013 |
| *DEFB1* | 1672 | TC08000906.hg.1 | defensin, beta 1 | 1.34 | 0.0001 | -1.05 | 0.1599 | 1.1 | 0.0236 | -1.1 | 0.0236 | -1.14 | 0.0098 | 1.04 | 0.2247 | -1.04 | 0.9912 | -1.1 | 0.1020 |
| *S1PR1* | 1901 | TC01000909.hg.1 | sphingosine-1-phosphate receptor 1 | 1.03 | 0.2497 | 1.06 | 0.0096 | -1.12 | 0.0001 | 1.12 | 0.0001 | 1.09 | 0.0007 | -1.06 | 0.0580 | -1.03 | 0.2001 | 1.03 | 0.0933 |
| *EDN1* | 1906 | TC06000087.hg.1 | endothelin 1 | 1.16 | 0.0008 | -1.01 | 0.3083 | 1.13 | 0.0023 | -1.13 | 0.0023 | -1.11 | 0.0035 | 1.12 | 0.0689 | 1.01 | 0.6917 | 1.01 | 0.4461 |
| *EGF* | 1950 | TC04000568.hg.1 | epidermal growth factor; epidermal growth factor (beta-urogastrone) | 1.21 | 0.0000 | -1.05 | 0.0521 | 1.05 | 0.0062 | -1.05 | 0.0062 | -1.06 | 0.0015 | 1.01 | 0.4393 | -1.01 | 0.9587 | -1.05 | 0.0217 |
| *EGFR* | 1956 | TC07000328.hg.1 | epidermal growth factor receptor; epidermal growth factor receptor (erythroblastic leukemia viral (v-erb-b) oncogene homolog, avian) | 1.28 | 0.0000 | -1.1 | 0.0104 | 1.11 | 0.0016 | -1.11 | 0.0016 | -1.12 | 0.0014 | 1.02 | 0.8719 | -1.01 | 0.8103 | -1.1 | 0.0070 |
| *EIF4E* | 1977 | TC04001403.hg.1 | eukaryotic translation initiation factor 4E | -1.49 | 0.0000 | 1.22 | 0.0019 | -1.19 | 0.0018 | 1.19 | 0.0018 | 1.13 | 0.0547 | 1.02 | 0.5959 | -1.05 | 0.3596 | 1.16 | 0.0418 |

| | | | | | | | | | | | | | | | | | | | |
|---|---|---|---|---|---|---|---|---|---|---|---|---|---|---|---|---|---|---|---|
| *EIF4EBP1* | 1978 | TC08000281.hg.1 | eukaryotic translation initiation factor 4E binding protein 1 | 1.42 | 0.0000 | -1.07 | 0.1420 | 1.18 | 0.0001 | -1.18 | 0.0001 | -1.17 | 0.0000 | 1.1 | 0.0117 | 1.01 | 0.9521 | -1.07 | 0.1118 |
| *ENG* | 2022 | TC09001609.hg.1 | endoglin | 1.3 | 0.0001 | -1.13 | 0.0242 | 1.16 | 0.0001 | -1.16 | 0.0001 | -1.21 | 0.0000 | 1.02 | 0.5388 | -1.04 | 0.1320 | -1.18 | 0.0032 |
| *ENO1* | 2023 | TC01002175.hg.1 | enolase 1, (alpha) | -1.22 | 0.0000 | 1.07 | 0.1896 | -1.05 | 0.6694 | 1.05 | 0.6694 | 1.06 | 0.0211 | 1.03 | 0.3432 | 1.01 | 0.0858 | 1.08 | 0.0002 |
| *ENO2* | 2026 | TC12000099.hg.1 | enolase 2 (gamma, neuronal) | -1.09 | 0.0019 | -1.05 | 0.0738 | 1.02 | 0.0966 | -1.02 | 0.0966 | 1.01 | 0.5888 | -1.03 | 0.9731 | 1.03 | 0.0794 | -1.02 | 0.4443 |
| *ENO3* | 2027 | TC17000058.hg.1 | enolase 3 (beta, muscle) | 1.15 | 0.0000 | -1.12 | 0.0000 | 1.1 | 0.0009 | -1.1 | 0.0009 | -1.07 | 0.0038 | -1.02 | 0.3847 | 1.03 | 0.3039 | -1.09 | 0.0002 |
| *EP300; MIR1281* | 2033 | TC22000332.hg.1 | E1A binding protein p300; microRNA 1281 | -1.63 | 0.0000 | 1.56 | 0.0001 | -1.34 | 0.0002 | 1.34 | 0.0002 | 1.42 | 0.0011 | 1.17 | 0.0545 | 1.06 | 0.9983 | 1.65 | 0.0004 |
| *EPAS1* | 2034 | TC02000281.hg.1 | endothelial PAS domain protein 1 | 1.28 | 0.0000 | -1.11 | 0.0008 | 1.1 | 0.0000 | -1.1 | 0.0000 | -1.11 | 0.0000 | -1.01 | 0.4173 | -1.01 | 0.9149 | -1.12 | 0.0004 |
| *EPO* | 2056 | TC07000632.hg.1 | erythropoietin | 1.42 | 0.0000 | -1.12 | 0.0655 | 1.11 | 0.0092 | -1.11 | 0.0092 | -1.22 | 0.0065 | -1.01 | 0.9958 | -1.11 | 0.3481 | -1.24 | 0.0271 |
| *ERBB2; MIR4728* | 2064 | TC17000480.hg.1 | v-erb-b2 avian erythroblastic leukemia viral oncogene homolog 2; microRNA 4728; v-erb-b2 erythroblastic leukemia viral oncogene homolog 2, neuro/glioblastoma derived oncogene homolog (avian) | 1.28 | 0.0000 | -1.15 | 0.0016 | 1.17 | 0.0002 | -1.17 | 0.0002 | -1.17 | 0.0006 | 1.01 | 0.8404 | 1 | 0.8773 | -1.15 | 0.0038 |
| *FLT1* | 2321 | TC13000517.hg.1 | fms-related tyrosine kinase 1; fms-related tyrosine kinase 1 (vascular endothelial growth factor/vascular permeability factor receptor) | -1.67 | 0.0000 | 1.32 | 0.0000 | -1.2 | 0.0003 | 1.2 | 0.0003 | 1.13 | 0.0289 | 1.11 | 0.0023 | -1.06 | 0.3894 | 1.25 | 0.0005 |
| *FN1* | 2335 | TC02002747.hg.1 | fibronectin 1 | 1.22 | 0.0000 | -1.07 | 0.0128 | 1.08 | 0.0005 | -1.08 | 0.0005 | -1.09 | 0.0013 | 1.01 | 0.9530 | -1 | 0.9925 | -1.08 | 0.0135 |
| *MTOR* | 2475 | TC01002197.hg.1 | mechanistic target of rapamycin (serine/threonine kinase) | -1.22 | 0.0001 | 1.06 | 0.2996 | -1.08 | 0.0448 | 1.08 | 0.0448 | 1.1 | 0.0046 | -1.02 | 0.6494 | 1.03 | 0.2711 | 1.09 | 0.0617 |
| *GAPDH* | 2597 | TC12000084.hg.1 | glyceraldehyde-3-phosphate dehydrogenase | 1.02 | 0.2816 | -1.02 | 0.0892 | -1.03 | 0.7456 | 1.03 | 0.7456 | 1.04 | 0.1354 | -1.05 | 0.1880 | 1.01 | 0.0639 | -1.01 | 0.8717 |
| *GPI* | 2821 | TC19000442.hg.1 | glucose-6-phosphate isomerase | -1.13 | 0.0016 | -1.03 | 0.1377 | 1.03 | 0.0873 | -1.03 | 0.0873 | -1.01 | 0.4773 | 1 | 0.4425 | 1.02 | 0.1465 | -1.01 | 0.9241 |
| *MKNK2* | 2872 | TC19001019.hg.1 | MAP kinase interacting serine/threonine kinase 2 | 1.2 | 0.0000 | 1.01 | 0.8171 | 1.09 | 0.0110 | -1.09 | 0.0110 | -1.08 | 0.0026 | 1.1 | 0.0009 | 1.01 | 0.8116 | 1.02 | 0.4849 |
| *CXCL2* | 2920 | TC04001286.hg.1 | chemokine (C-X-C motif) ligand 2 | 1.27 | 0.0001 | -1.12 | 0.0153 | 1.1 | 0.0012 | -1.1 | 0.0012 | -1.13 | 0.0002 | -1.02 | 0.9801 | -1.03 | 0.2381 | -1.16 | 0.0033 |
| *HDAC2* | 3066 | TC06002035.hg.1 | histone deacetylase 2 | -2.04 | 0.0000 | 1.41 | 0.0003 | -1.26 | 0.0092 | 1.26 | 0.0092 | 1.11 | 0.0973 | 1.12 | 0.1679 | -1.14 | 0.5062 | 1.23 | 0.0125 |
| *HIF1A* | 3091 | TC14002197.hg.1 | hypoxia inducible factor 1, alpha subunit (basic helix- | -2.38 | 0.0000 | 1.66 | 0.0007 | -1.55 | 0.0009 | 1.55 | 0.0009 | 1.3 | 0.0362 | 1.07 | 0.2663 | -1.19 | 0.3220 | 1.4 | 0.0124 |

| | | | | | | | | | | | | | | | | | | | |
|---|---|---|---|---|---|---|---|---|---|---|---|---|---|---|---|---|---|---|---|
| | | | loop-helix transcription factor) | | | | | | | | | | | | | | | | |
| *HK1* | 3098 | TC10000419.hg.1 | hexokinase 1 | -1.26 | 0.0000 | 1.08 | 0.1621 | -1.01 | 0.3033 | 1.01 | 0.3033 | 1.03 | 0.3802 | 1.07 | 0.0006 | 1.02 | 0.1318 | 1.1 | 0.0002 |
| *HK2* | 3099 | TC02000466.hg.1 | hexokinase 2 | 1.09 | 0.0048 | -1.06 | 0.0728 | -1.18 | 0.0001 | 1.18 | 0.0001 | 1.28 | 0.0001 | -1.25 | 0.0000 | 1.09 | 0.4785 | 1.03 | 0.3288 |
| *HK3* | 3101 | TC05002100.hg.1 | hexokinase 3 (white cell) | 1.42 | 0.0000 | -1.2 | 0.0017 | 1.18 | 0.0001 | -1.18 | 0.0001 | -1.14 | 0.0002 | -1.02 | 0.6490 | 1.03 | 0.7576 | -1.16 | 0.0029 |
| *HMOX1* | 3162 | TC22000259.hg.1 | heme oxygenase (decycling) 1 | 1.23 | 0.0001 | -1.12 | 0.0089 | 1.18 | 0.0001 | -1.18 | 0.0001 | -1.24 | 0.0000 | 1.06 | 0.5567 | -1.05 | 0.0857 | -1.17 | 0.0009 |
| *ICAM1* | 3383 | TC19000174.hg.1 | intercellular adhesion molecule 1 | 1.43 | 0.0000 | -1.14 | 0.0075 | 1.14 | 0.0010 | -1.14 | 0.0010 | -1.18 | 0.0015 | 1 | 0.6940 | -1.03 | 0.9306 | -1.17 | 0.0103 |
| *IFNG* | 3458 | TC12001696.hg.1 | interferon, gamma | 1.14 | 0.0001 | 1.01 | 0.4260 | 1.04 | 0.4784 | -1.04 | 0.4784 | -1.07 | 0.3323 | 1.05 | 0.2564 | -1.03 | 0.6723 | -1.02 | 0.9166 |
| *IFNGR1* | 3459 | TC06002152.hg.1 | interferon gamma receptor 1 | -1.61 | 0.0000 | 1.36 | 0.0000 | -1.15 | 0.0232 | 1.15 | 0.0232 | 1.07 | 0.1987 | 1.18 | 0.0003 | -1.07 | 0.5233 | 1.26 | 0.0015 |
| *IFNGR2* | 3460 | TC21000129.hg.1 | interferon gamma receptor 2 (interferon gamma transducer 1) | -1.07 | 0.0044 | 1.01 | 0.7075 | 1.02 | 0.1189 | -1.02 | 0.1189 | -1.01 | 0.7557 | 1.03 | 0.1733 | 1.01 | 0.2502 | 1.02 | 0.3598 |
| *IGF1* | 3479 | TC12001890.hg.1 | insulin-like growth factor 1 (somatomedin C) | 1.18 | 0.0000 | -1.05 | 0.0590 | 1.08 | 0.0214 | -1.08 | 0.0214 | -1.04 | 0.0777 | 1.02 | 0.3720 | 1.03 | 0.1506 | -1.02 | 0.3308 |
| *IGF1R* | 3480 | TC15000949.hg.1 | insulin-like growth factor 1 receptor | -1.05 | 0.0039 | 1.08 | 0.0237 | -1.05 | 0.3860 | 1.05 | 0.3860 | 1.07 | 0.0078 | 1.03 | 0.0605 | 1.02 | 0.1299 | 1.1 | 0.0001 |
| *IGF1R* | 3480 | TC15000951.hg.1 | insulin-like growth factor 1 receptor | -1.03 | 0.1524 | -1.08 | 0.1386 | 1.14 | 0.0150 | -1.14 | 0.0150 | -1.1 | 0.1067 | 1.05 | 0.1625 | 1.03 | 0.3011 | -1.04 | 0.7725 |
| *IGFBP1* | 3484 | TC07000294.hg.1 | insulin-like growth factor binding protein 1 | 1.33 | 0.0000 | -1.22 | 0.0006 | 1.16 | 0.0028 | -1.16 | 0.0028 | -1.04 | 0.1031 | -1.05 | 0.4007 | 1.11 | 0.1286 | -1.09 | 0.0270 |
| *IGFBP2* | 3485 | TC02001281.hg.1 | insulin-like growth factor binding protein 2, 36kDa | 1.08 | 0.0012 | 1 | 0.8647 | 1.11 | 0.0013 | -1.11 | 0.0013 | -1.11 | 0.0000 | 1.12 | 0.0000 | 1 | 0.9946 | 1 | 0.7720 |
| *IGFBP3* | 3486 | TC07001355.hg.1 | insulin-like growth factor binding protein 3 | 1.34 | 0.0000 | -1.13 | 0.0113 | 1.15 | 0.0000 | -1.15 | 0.0000 | -1.19 | 0.0000 | 1.01 | 0.7689 | -1.04 | 0.3040 | -1.18 | 0.0030 |
| *IL1B* | 3553 | TC02002219.hg.1 | interleukin 1, beta | 1.26 | 0.0000 | -1.1 | 0.0043 | 1.07 | 0.0013 | -1.07 | 0.0013 | -1.06 | 0.0017 | -1.02 | 0.4661 | 1.01 | 0.6974 | -1.08 | 0.0038 |
| *IL6* | 3569 | TC07000137.hg.1 | interleukin 6; interleukin 6 (interferon, beta 2) | 1.19 | 0.0000 | -1.04 | 0.0202 | 1.08 | 0.0010 | -1.08 | 0.0010 | -1.1 | 0.0002 | 1.04 | 0.0585 | -1.02 | 0.4600 | -1.06 | 0.0076 |
| *IL6R* | 3570 | TC01001276.hg.1 | interleukin 6 receptor | 1.39 | 0.0000 | -1.13 | 0.0192 | 1.18 | 0.0000 | -1.18 | 0.0000 | -1.23 | 0.0001 | 1.05 | 0.4765 | -1.04 | 0.6779 | -1.17 | 0.0147 |
| *CXCL8; IL8* | 3576 | TC04000408.hg.1 | chemokine (C-X-C motif) ligand 8; interleukin 8 | 1.17 | 0.0001 | -1.05 | 0.4581 | 1.05 | 0.3924 | -1.05 | 0.3924 | -1.11 | 0.0044 | 1 | 0.9693 | -1.06 | 0.1255 | -1.11 | 0.0092 |
| *IL12A* | 3592 | TC03000870.hg.1 | interleukin 12A; interleukin 12A (natural killer cell stimulatory factor 1, cytotoxic lymphocyte maturation factor 1, p35) | 1.26 | 0.0000 | -1.05 | 0.1455 | 1.12 | 0.0027 | -1.12 | 0.0027 | -1.15 | 0.0010 | 1.07 | 0.0436 | -1.02 | 0.8589 | -1.07 | 0.0973 |
| *TNFRSF9* | 3604 | TC01002163.hg.1 | tumor necrosis factor receptor superfamily, member 9 | 1.2 | 0.0001 | -1.11 | 0.0060 | 1.14 | 0.0001 | -1.14 | 0.0001 | -1.12 | 0.0007 | 1.03 | 0.4978 | 1.02 | 0.3804 | -1.09 | 0.0208 |

| | | | | | | | | | | | | | | | | | |
|---|---|---|---|---|---|---|---|---|---|---|---|---|---|---|---|---|---|
| *INS; IGF2; INS-IGF2; AC132217.4* | 3630 | TC11001274.hg.1 | insulin; insulin-like growth factor 2 (somatomedin A); INS-IGF2 readthrough; novel transcript, 3'_overlapping_ncRNA IGF2 | 1.35 | 0.0000 | -1.14 | 0.0050 | 1.16 | 0.0000 | -1.16 | 0.0000 | -1.15 | 0.0004 | 1.01 | 0.7546 | 1 | 0.7645 | -1.14 | 0.0079 |
| *INSR* | 3643 | TC19001111.hg.1 | insulin receptor | 1.24 | 0.0000 | -1.09 | 0.0036 | -1.04 | 0.1528 | 1.04 | 0.1528 | 1.05 | 0.0382 | -1.14 | 0.0001 | 1 | 0.4553 | -1.09 | 0.0145 |
| *ITGAM* | 3684 | TC16000374.hg.1 | integrin, alpha M (complement component 3 receptor 3 subunit) | 1.28 | 0.0000 | -1.11 | 0.0043 | 1.13 | 0.0001 | -1.13 | 0.0001 | -1.15 | 0.0005 | 1.02 | 0.6855 | -1.02 | 0.9317 | -1.13 | 0.0066 |
| *ITGB2* | 3689 | TC21000538.hg.1 | integrin, beta 2 (complement component 3 receptor 3 and 4 subunit) | 1.27 | 0.0000 | -1.13 | 0.0000 | 1.09 | 0.0002 | -1.09 | 0.0002 | -1.06 | 0.0130 | -1.04 | 0.0122 | 1.03 | 0.3117 | -1.1 | 0.0002 |
| *KDR* | 3791 | TC04001208.hg.1 | kinase insert domain receptor (a type III receptor tyrosine kinase) | 1.24 | 0.0000 | -1.09 | 0.0091 | 1.08 | 0.0000 | -1.08 | 0.0000 | -1.07 | 0.0001 | -1.01 | 0.8950 | 1.01 | 0.5854 | -1.08 | 0.0138 |
| *KRT14* | 3861 | TC17001513.hg.1 | keratin 14 | 1.23 | 0.0007 | -1.09 | 0.1528 | 1.11 | 0.0048 | -1.11 | 0.0048 | -1.13 | 0.0001 | 1.02 | 0.7106 | -1.02 | 0.0501 | -1.11 | 0.0119 |
| *KRT18* | 3875 | TC12000424.hg.1 | keratin 18 | 1.19 | 0.0011 | -1.13 | 0.0122 | 1.14 | 0.0086 | -1.14 | 0.0086 | -1.11 | 0.0189 | 1.01 | 0.5785 | 1.03 | 0.4416 | -1.1 | 0.0176 |
| *KRT19* | 3880 | TC17002906.hg.1 | keratin 19 | 1.34 | 0.0000 | -1.12 | 0.0124 | 1.11 | 0.0002 | -1.11 | 0.0002 | -1.11 | 0.0029 | -1 | 0.9142 | -1 | 0.8112 | -1.12 | 0.0194 |
| *LAG3* | 3902 | TC12000091.hg.1 | lymphocyte-activation gene 3 | 1.24 | 0.0009 | -1.11 | 0.0529 | 1.14 | 0.0013 | -1.14 | 0.0013 | -1.16 | 0.0012 | 1.03 | 0.6702 | -1.01 | 0.7919 | -1.12 | 0.0337 |
| *LDHA* | 3939 | TC11000240.hg.1 | lactate dehydrogenase A | -2.29 | 0.0000 | 1.4 | 0.0017 | -1.23 | 0.0116 | 1.23 | 0.0116 | 1.16 | 0.1248 | 1.14 | 0.2404 | -1.06 | 0.4053 | 1.32 | 0.0222 |
| *LEP* | 3952 | TC07000768.hg.1 | leptin | 1.23 | 0.0000 | -1.05 | 0.0335 | 1.16 | 0.0004 | -1.16 | 0.0004 | -1.22 | 0.0006 | 1.11 | 0.0944 | -1.04 | 0.5312 | -1.09 | 0.0386 |
| *LRP1* | 4035 | TC12000521.hg.1 | low density lipoprotein receptor-related protein 1 | 1.33 | 0.0000 | -1.14 | 0.0008 | 1.15 | 0.0014 | -1.15 | 0.0014 | -1.14 | 0.0067 | 1.01 | 0.3000 | 1 | 0.5558 | -1.14 | 0.0024 |
| *LTBR* | 4055 | TC12000078.hg.1 | lymphotoxin beta receptor (TNFR superfamily, member 3) | 1.46 | 0.0000 | -1.14 | 0.0095 | 1.22 | 0.0000 | -1.22 | 0.0000 | -1.28 | 0.0000 | 1.07 | 0.1139 | -1.05 | 0.4630 | -1.2 | 0.0031 |
| *MMP2* | 4313 | TC16000454.hg.1 | matrix metallopeptidase 2 (gelatinase A, 72kDa gelatinase, 72kDa type IV collagenase) | 1.35 | 0.0000 | -1.13 | 0.0133 | 1.17 | 0.0003 | -1.17 | 0.0003 | -1.17 | 0.0009 | 1.03 | 0.4497 | -1 | 0.8804 | -1.14 | 0.0219 |
| *MMP9* | 4318 | TC20000363.hg.1 | matrix metallopeptidase 9 (gelatinase B, 92kDa gelatinase, 92kDa type IV collagenase) | 1.56 | 0.0000 | -1.24 | 0.0022 | 1.19 | 0.0024 | -1.19 | 0.0024 | -1.17 | 0.0004 | -1.04 | 0.3218 | 1.02 | 0.8780 | -1.21 | 0.0004 |
| *MMP14* | 4323 | TC14000133.hg.1 | matrix metallopeptidase 14 (membrane-inserted) | 1.3 | 0.0000 | -1.12 | 0.0017 | 1.13 | 0.0011 | -1.13 | 0.0011 | -1.1 | 0.0089 | 1.01 | 0.4538 | 1.03 | 0.3576 | -1.08 | 0.0061 |
| *NFKB1* | 4790 | TC04000526.hg.1 | nuclear factor of kappa light polypeptide gene enhancer in B-cells 1 | -1.42 | 0.0000 | 1.22 | 0.0001 | -1.18 | 0.0014 | 1.18 | 0.0014 | 1.24 | 0.0033 | 1.03 | 0.0829 | 1.05 | 0.6939 | 1.28 | 0.0006 |

| | | | | | | | | | | | | | | | | | |
|---|---|---|---|---|---|---|---|---|---|---|---|---|---|---|---|---|---|
| *NOS2* | 4843 | TC17002894.hg.1 | nitric oxide synthase 2, inducible | 1.27 | 0.0000 | -1.11 | 0.0009 | 1.15 | 0.0000 | -1.15 | 0.0000 | -1.17 | 0.0000 | 1.03 | 0.2850 | -1.02 | 0.6319 | -1.13 | 0.0011 |
| *NOS3; ATG9B* | 4846 | TC07001009.hg.1 | nitric oxide synthase 3 (endothelial cell); ATG9 autophagy related 9 homolog B (S. cerevisiae) | 1.29 | 0.0000 | -1.12 | 0.0034 | 1.19 | 0.0001 | -1.19 | 0.0001 | -1.17 | 0.0001 | 1.06 | 0.1204 | 1.01 | 0.8313 | -1.1 | 0.0074 |
| *NPPA* | 4878 | TC01002204.hg.1 | natriuretic peptide A; natriuretic peptide precursor A | 1.41 | 0.0000 | -1.16 | 0.0050 | 1.13 | 0.0349 | -1.13 | 0.0349 | -1.15 | 0.0208 | -1.03 | 0.7555 | -1.02 | 0.7101 | -1.18 | 0.0074 |
| *SERPINE1* | 5054 | TC07000643.hg.1 | serpin peptidase inhibitor, clade E (nexin, plasminogen activator inhibitor type 1), member 1 | 1.26 | 0.0000 | -1.09 | 0.0105 | 1.11 | 0.0007 | -1.11 | 0.0007 | -1.11 | 0.0023 | 1.02 | 0.6033 | 1 | 0.5559 | -1.09 | 0.0248 |
| *PDGFB* | 5155 | TC22000802.hg.1 | platelet-derived growth factor beta polypeptide; platelet-derived growth factor beta polypeptide (simian sarcoma viral (v-sis) oncogene homolog) | 1.44 | 0.0000 | -1.15 | 0.0152 | 1.16 | 0.0005 | -1.16 | 0.0005 | -1.17 | 0.0005 | 1.01 | 0.7158 | -1.01 | 0.7321 | -1.16 | 0.0099 |
| *PDHA1* | 5160 | TC0X000092.hg.1 | pyruvate dehydrogenase (lipoamide) alpha 1 | -1.33 | 0.0000 | 1.11 | 0.0087 | -1.2 | 0.0002 | 1.2 | 0.0002 | 1.28 | 0.0008 | -1.08 | 0.3955 | 1.06 | 0.6887 | 1.19 | 0.0152 |
| *PDHA2* | 5161 | TC04000504.hg.1 | pyruvate dehydrogenase (lipoamide) alpha 2 | 1.31 | 0.0000 | -1.14 | 0.0289 | 1.13 | 0.0466 | -1.13 | 0.0466 | -1.07 | 0.4385 | -1.01 | 0.9717 | 1.06 | 0.2236 | -1.07 | 0.4100 |
| *PDHB* | 5162 | TC03001499.hg.1 | pyruvate dehydrogenase (lipoamide) beta | -1.34 | 0.0000 | 1.02 | 0.2627 | -1.21 | 0.0000 | 1.21 | 0.0000 | 1.21 | 0.0002 | -1.19 | 0.0015 | -1 | 0.8984 | 1.02 | 0.4173 |
| *PDK1* | 5163 | TC02001031.hg.1 | pyruvate dehydrogenase kinase, isozyme 1 | -2.3 | 0.0000 | 1.17 | 0.0010 | -1.09 | 0.0284 | 1.09 | 0.0284 | 1.06 | 0.2242 | 1.07 | 0.1210 | -1.03 | 0.4599 | 1.14 | 0.0189 |
| *PFKFB3* | 5209 | TC10000053.hg.1 | 6-phosphofructo-2-kinase/fructose-2,6-biphosphatase 3 | 1.04 | 0.0418 | -1.07 | 0.0043 | 1.03 | 0.0613 | -1.03 | 0.0613 | -1.05 | 0.0118 | -1.04 | 0.2920 | -1.02 | 0.4917 | -1.09 | 0.0009 |
| *PFKL* | 5211 | TC21000222.hg.1 | phosphofructokinase, liver | 1.08 | 0.0011 | -1.07 | 0.0072 | 1.12 | 0.0002 | -1.12 | 0.0002 | -1.08 | 0.0051 | 1.05 | 0.0464 | 1.03 | 0.2326 | -1.03 | 0.1565 |
| *PGK1; LOC100652805; LOC100653302* | 5230 | TC0X000425.hg.1 | phosphoglycerate kinase 1; uncharacterized LOC100652805; uncharacterized LOC100653302 | -1.42 | 0.0000 | 1.13 | 0.0442 | -1.12 | 0.0418 | 1.12 | 0.0418 | 1.12 | 0.0152 | 1.01 | 0.9018 | -1 | 0.6581 | 1.13 | 0.0187 |
| *ABCB1* | 5243 | TC07001579.hg.1 | ATP-binding cassette, sub-family B (MDR/TAP), member 1 | 1.16 | 0.0000 | -1.05 | 0.0196 | 1.08 | 0.0005 | -1.08 | 0.0005 | -1.08 | 0.0007 | 1.03 | 0.0762 | 1 | 0.9580 | -1.05 | 0.0400 |
| *PIK3CA* | 5290 | TC03000951.hg.1 | phosphatidylinositol-4,5-bisphosphate 3-kinase, catalytic subunit alpha; phosphoinositide-3-kinase, catalytic, alpha polypeptide | -3.15 | 0.0000 | 1.63 | 0.0013 | -1.38 | 0.0057 | 1.38 | 0.0057 | 1.29 | 0.0606 | 1.18 | 0.1645 | -1.07 | 0.4944 | 1.53 | 0.0127 |
| *PIK3CB* | 5291 | TC03001824.hg.1 | phosphatidylinositol-4,5-bisphosphate 3-kinase, catalytic subunit beta; | -2.54 | 0.0000 | 1.36 | 0.0000 | -1.22 | 0.0045 | 1.22 | 0.0045 | 1.22 | 0.0206 | 1.11 | 0.0102 | -1 | 0.9172 | 1.36 | 0.0004 |

| | | | | | | | | | | | | | | | | | | | |
|---|---|---|---|---|---|---|---|---|---|---|---|---|---|---|---|---|---|---|---|
| | | | phosphoinositide-3-kinase, catalytic, beta polypeptide | | | | | | | | | | | | | | | | |
| *PIK3CD* | 5293 | TC01000118.hg.1 | phosphatidylinositol-4,5-bisphosphate 3-kinase, catalytic subunit delta; phosphoinositide-3-kinase, catalytic, delta polypeptide | 1.23 | 0.0000 | -1.11 | 0.0004 | 1.11 | 0.0011 | -1.11 | 0.0011 | -1.09 | 0.0036 | -1 | 0.7489 | 1.02 | 0.3083 | -1.09 | 0.0013 |
| *PIK3R1* | 5295 | TC05000291.hg.1 | phosphoinositide-3-kinase, regulatory subunit 1 (alpha) | -2.16 | 0.0000 | 1.21 | 0.0316 | -1.37 | 0.0003 | 1.37 | 0.0003 | 1.26 | 0.0042 | -1.13 | 0.1598 | -1.09 | 0.3888 | 1.11 | 0.1557 |
| *PIK3R2* | 5296 | TC19002628.hg.1 | phosphoinositide-3-kinase, regulatory subunit 2 (beta) | 1.4 | 0.0000 | -1.04 | 0.1658 | 1.13 | 0.0000 | -1.13 | 0.0000 | -1.09 | 0.0009 | 1.08 | 0.0008 | 1.04 | 0.3689 | -1.01 | 0.7028 |
| *PKM* | 5315 | TC15002776.hg.1 | pyruvate kinase, muscle | -1.05 | 0.0266 | 1.03 | 0.8982 | -1.02 | 0.6682 | 1.02 | 0.6682 | 1.03 | 0.5352 | 1 | 0.7094 | 1 | 0.2349 | 1.03 | 0.1247 |
| *PLAUR* | 5329 | TC19001593.hg.1 | plasminogen activator, urokinase receptor | 1.14 | 0.0001 | -1.15 | 0.0009 | 1.17 | 0.0004 | -1.17 | 0.0004 | -1.1 | 0.0005 | 1.02 | 0.8695 | 1.06 | 0.1846 | -1.08 | 0.0009 |
| *PLCG1* | 5335 | TC20000303.hg.1 | phospholipase C, gamma 1 | 1.07 | 0.0254 | -1.04 | 0.1283 | -1.01 | 0.3752 | 1.01 | 0.3752 | 1.03 | 0.1672 | -1.05 | 0.3505 | 1.02 | 0.0366 | -1.02 | 0.8886 |
| *PLCG2* | 5336 | TC16000642.hg.1 | phospholipase C, gamma 2 (phosphatidylinositol-specific) | 1.15 | 0.2507 | -1.2 | 0.0248 | 1.15 | 0.0451 | -1.15 | 0.0451 | -1.07 | 0.2073 | -1.05 | 0.8229 | 1.07 | 0.4281 | -1.12 | 0.1514 |
| *PLCG2* | 5336 | TC16000643.hg.1 | phospholipase C, gamma 2 (phosphatidylinositol-specific) | -1.16 | 0.0001 | -1.05 | 0.2256 | -1 | 0.8491 | 1 | 0.8491 | 1.05 | 0.0007 | -1.05 | 0.1393 | 1.05 | 0.0023 | 1 | 0.1912 |
| *PRKCA* | 5578 | TC17000783.hg.1 | protein kinase C, alpha | -1.65 | 0.0000 | 1.41 | 0.0001 | -1.24 | 0.0017 | 1.24 | 0.0017 | 1.27 | 0.0035 | 1.14 | 0.0111 | 1.02 | 0.9409 | 1.45 | 0.0002 |
| *PRKCB* | 5579 | TC16000260.hg.1 | protein kinase C, beta | -1.25 | 0.0000 | 1.12 | 0.0097 | -1.19 | 0.0005 | 1.19 | 0.0005 | 1.29 | 0.0018 | -1.07 | 0.6120 | 1.09 | 0.8331 | 1.22 | 0.0157 |
| *PRKCG* | 5582 | TC19000866.hg.1 | protein kinase C, gamma | 1.4 | 0.0000 | -1.15 | 0.0207 | 1.16 | 0.0005 | -1.16 | 0.0005 | -1.2 | 0.0002 | 1.01 | 0.6794 | -1.03 | 0.3801 | -1.19 | 0.0065 |
| *MAPK1* | 5594 | TC22000547.hg.1 | mitogen-activated protein kinase 1 | -1.46 | 0.0000 | 1.37 | 0.0001 | -1.22 | 0.0001 | 1.22 | 0.0001 | 1.23 | 0.0007 | 1.12 | 0.0697 | 1.01 | 0.9334 | 1.38 | 0.0002 |
| *MAPK3* | 5595 | TC16001021.hg.1 | mitogen-activated protein kinase 3 | 1.14 | 0.0000 | -1.1 | 0.0006 | 1.09 | 0.0006 | -1.09 | 0.0006 | -1.06 | 0.0150 | -1.01 | 0.7176 | 1.03 | 0.1334 | -1.07 | 0.0102 |
| *MAP2K1* | 5604 | TC15000613.hg.1 | mitogen-activated protein kinase kinase 1 | 1.09 | 0.0329 | 1.07 | 0.0198 | -1.07 | 0.0004 | 1.07 | 0.0004 | 1.06 | 0.0191 | -1 | 0.9236 | -1.01 | 0.7388 | 1.06 | 0.0804 |
| *MAP2K2* | 5605 | TC19001058.hg.1 | mitogen-activated protein kinase kinase 2 | 1.33 | 0.0000 | -1.05 | 0.1598 | 1.12 | 0.0048 | -1.12 | 0.0048 | -1.08 | 0.0141 | 1.07 | 0.0144 | 1.04 | 0.3195 | -1.01 | 0.6387 |
| *RELA* | 5970 | TC11001939.hg.1 | v-rel avian reticuloendotheliosis viral oncogene homolog A; v-rel reticuloendotheliosis viral oncogene homolog A (avian) | 1.28 | 0.0000 | -1.1 | 0.0023 | 1.07 | 0.0146 | -1.07 | 0.0146 | -1.1 | 0.0182 | -1.02 | 0.7875 | -1.02 | 0.8876 | -1.12 | 0.0159 |
| *RORC* | 6097 | TC01006373.hg.1 | RAR-related orphan receptor C | 1.07 | 0.0263 | -1.11 | 0.0123 | 1.13 | 0.0002 | -1.13 | 0.0002 | -1.12 | 0.0155 | 1.02 | 0.5397 | 1.01 | 0.5280 | -1.1 | 0.0992 |
| *RPS6* | 6194 | TC09000938.hg.1 | ribosomal protein S6 | -1.52 | 0.0000 | 1.32 | 0.0003 | -1.21 | 0.0057 | 1.21 | 0.0057 | 1.31 | 0.0022 | 1.09 | 0.0253 | 1.08 | 0.5444 | 1.43 | 0.0001 |

| | | | | | | | | | | | | | | | | | | |
|---|---|---|---|---|---|---|---|---|---|---|---|---|---|---|---|---|---|---|
| *RPS6KB1* | 6198 | TC17000729.hg.1 | ribosomal protein S6 kinase, 70kDa, polypeptide 1 | -1.95 | 0.0000 | 1.26 | 0.0012 | -1.3 | 0.0005 | 1.3 | 0.0005 | 1.23 | 0.0076 | -1.03 | 0.9085 | -1.05 | 0.5062 | 1.2 | 0.0139 |
| *RPS6KB2* | 6199 | TC11000691.hg.1 | ribosomal protein S6 kinase, 70kDa, polypeptide 2 | 1.28 | 0.0000 | -1.05 | 0.0790 | 1.14 | 0.0009 | -1.14 | 0.0009 | -1.09 | 0.0237 | 1.09 | 0.0031 | 1.05 | 0.0676 | 1 | 0.7795 |
| *CCL2* | 6347 | TC17000383.hg.1 | chemokine (C-C motif) ligand 2 | 1.2 | 0.0094 | 1.04 | 0.2735 | 1.07 | 0.2604 | -1.07 | 0.2604 | -1.21 | 0.0019 | 1.11 | 0.0397 | -1.13 | 0.0277 | -1.09 | 0.2591 |
| *CXCL6* | 6372 | TC04000409.hg.1 | chemokine (C-X-C motif) ligand 6; chemokine (C-X-C motif) ligand 6 (granulocyte chemotactic protein 2) | 1.27 | 0.0000 | -1.08 | 0.0296 | 1.13 | 0.0016 | -1.13 | 0.0016 | -1.2 | 0.0030 | 1.04 | 0.2960 | -1.07 | 0.6925 | -1.16 | 0.0408 |
| *SELL* | 6402 | TC01003500.hg.1 | selectin L | -2.58 | 0.0000 | 1.76 | 0.0003 | -1.15 | 0.1692 | 1.15 | 0.1692 | 1.12 | 0.2468 | 1.54 | 0.0003 | -1.03 | 0.6940 | 1.72 | 0.0001 |
| *SLC2A1* | 6513 | TC01002578.hg.1 | solute carrier family 2 (facilitated glucose transporter), member 1 | 1.15 | 0.0000 | -1.03 | 0.0283 | 1.09 | 0.0028 | -1.09 | 0.0028 | -1.05 | 0.0448 | 1.06 | 0.1585 | 1.04 | 0.0663 | 1 | 0.5335 |
| *SLC2A3* | 6515 | TC12001170.hg.1 | solute carrier family 2 (facilitated glucose transporter), member 3 | -1.28 | 0.0000 | 1.08 | 0.0563 | -1.04 | 0.1307 | 1.04 | 0.1307 | 1.05 | 0.0058 | 1.04 | 0.3307 | 1.01 | 0.2222 | 1.09 | 0.0045 |
| *SLC11A1* | 6556 | TC02001300.hg.1 | solute carrier family 11 (proton-coupled divalent metal ion transporter), member 1; solute carrier family 11 (proton-coupled divalent metal ion transporters), member 1 | 1.23 | 0.0000 | -1.11 | 0.0005 | 1.1 | 0.0000 | -1.1 | 0.0000 | -1.14 | 0.0000 | -1.01 | 0.6679 | -1.03 | 0.4942 | -1.15 | 0.0003 |
| *STAT3* | 6774 | TC17001531.hg.1 | signal transducer and activator of transcription 3 (acute-phase response factor) | -1.38 | 0.0000 | 1.26 | 0.0008 | -1.08 | 0.3405 | 1.08 | 0.3405 | 1.08 | 0.0899 | 1.16 | 0.0004 | -1.01 | 0.4956 | 1.25 | 0.0002 |
| *STAT4* | 6775 | TC02002625.hg.1 | signal transducer and activator of transcription 4 | 1.11 | 0.0000 | -1.04 | 0.0021 | 1.05 | 0.0076 | -1.05 | 0.0076 | -1.04 | 0.0074 | 1 | 0.9470 | 1 | 0.5876 | -1.04 | 0.0024 |
| *TCEB1* | 6921 | TC08001332.hg.1 | transcription elongation factor B (SIII), polypeptide 1 (15kDa, elongin C) | -1.31 | 0.0000 | 1.08 | 0.0061 | -1.09 | 0.0010 | 1.09 | 0.0010 | 1.05 | 0.0682 | -1.01 | 0.9001 | -1.04 | 0.2810 | 1.04 | 0.1361 |
| *TCEB2* | 6923 | TC16000791.hg.1 | transcription elongation factor B (SIII), polypeptide 2 (18kDa, elongin B) | 1.56 | 0.0000 | 1 | 0.2559 | 1.09 | 0.0020 | -1.09 | 0.0020 | -1.04 | 0.0784 | 1.1 | 0.0164 | 1.05 | 0.0275 | 1.06 | 0.2558 |
| *TCF7* | 6932 | TC05000657.hg.1 | transcription factor 7 (T-cell specific, HMG-box) | -1.09 | 0.0132 | 1 | 0.3095 | -1.01 | 0.8134 | 1.01 | 0.8134 | -1.02 | 0.2694 | -1.01 | 0.4197 | -1.03 | 0.3867 | -1.03 | 0.0479 |
| *TEK* | 7010 | TC09000121.hg.1 | TEK tyrosine kinase, endothelial | 1.2 | 0.0000 | -1.05 | 0.0095 | 1.06 | 0.0036 | -1.06 | 0.0036 | -1.06 | 0.0714 | 1.01 | 0.9712 | -1.01 | 0.4436 | -1.06 | 0.1079 |
| *TF; TFP1* | 7018 | TC03000723.hg.1 | transferrin; transferrin pseudogene 1 | 1.25 | 0.0000 | -1.11 | 0.0088 | 1.11 | 0.0003 | -1.11 | 0.0003 | -1.11 | 0.0027 | 1.01 | 0.8895 | 1 | 0.4917 | -1.11 | 0.0241 |
| *TFF3* | 7033 | TC21000484.hg.1 | trefoil factor 3 (intestinal) | 1.37 | 0.0000 | -1.12 | 0.0640 | 1.19 | 0.0004 | -1.19 | 0.0004 | -1.18 | 0.0005 | 1.06 | 0.0212 | 1.01 | 0.9521 | -1.11 | 0.0901 |

| | | | | | | | | | | | | | | | | | | | |
|---|---|---|---|---|---|---|---|---|---|---|---|---|---|---|---|---|---|---|---|
| *TFRC* | 7037 | TC03002155.hg.1 | transferrin receptor; transferrin receptor (p90, CD71) | -2.56 | 0.0000 | 1.63 | 0.0002 | -1.45 | 0.0006 | 1.45 | 0.0006 | 1.3 | 0.0380 | 1.12 | 0.1888 | -1.11 | 0.3455 | 1.46 | 0.0086 |
| *TGFA* | 7039 | TC02001963.hg.1 | transforming growth factor, alpha | 1.26 | 0.0000 | -1.12 | 0.0116 | 1.15 | 0.0000 | -1.15 | 0.0000 | -1.13 | 0.0016 | 1.03 | 0.3849 | 1.02 | 0.4868 | -1.1 | 0.0590 |
| *TGFB1* | 7040 | TC19001553.hg.1 | transforming growth factor, beta 1 | 1.21 | 0.0002 | -1.08 | 0.0489 | 1.09 | 0.0342 | -1.09 | 0.0342 | -1.05 | 0.1641 | 1.01 | 0.6491 | 1.04 | 0.3471 | -1.04 | 0.2857 |
| *TGFB3* | 7043 | TC14001326.hg.1 | transforming growth factor, beta 3 | 1.21 | 0.0002 | -1.08 | 0.0349 | 1.06 | 0.0131 | -1.06 | 0.0131 | -1.05 | 0.0255 | -1.02 | 0.7473 | 1.01 | 0.6684 | -1.07 | 0.0706 |
| *THBS1* | 7057 | TC15000270.hg.1 | thrombospondin 1 | 1.26 | 0.0000 | -1.09 | 0.0066 | 1.09 | 0.0008 | -1.09 | 0.0008 | -1.15 | 0.0013 | 1 | 0.8717 | -1.05 | 0.8298 | -1.15 | 0.0061 |
| *TIMP1* | 7076 | TC0X000238.hg.1 | TIMP metallopeptidase inhibitor 1 | 1.37 | 0.0000 | -1.14 | 0.0117 | 1.2 | 0.0007 | -1.2 | 0.0007 | -1.21 | 0.0001 | 1.05 | 0.2107 | -1.01 | 0.6459 | -1.16 | 0.0036 |
| *TLR4* | 7099 | TC09000601.hg.1 | toll-like receptor 4 | 1.24 | 0.0000 | -1.07 | 0.0559 | 1.14 | 0.0009 | -1.14 | 0.0009 | -1.12 | 0.0010 | 1.06 | 0.1658 | 1.01 | 0.9640 | -1.05 | 0.0631 |
| *TNF* | 7124 | TC06000371.hg.1 | tumor necrosis factor; tumor necrosis factor (TNF superfamily, member 2) | 1.73 | 0.0000 | -1.35 | 0.0018 | 1.27 | 0.0001 | -1.27 | 0.0001 | -1.25 | 0.0000 | -1.06 | 0.7019 | 1.02 | 0.5330 | -1.33 | 0.0009 |
| *TNF* | 7124 | TC6_apd_hap1000036.hg.1 | tumor necrosis factor; tumor necrosis factor (TNF superfamily, member 2) | 1.76 | 0.0000 | -1.35 | 0.0026 | 1.31 | 0.0000 | -1.31 | 0.0000 | -1.32 | 0.0001 | -1.03 | 0.6111 | -1.01 | 0.8450 | -1.36 | 0.0044 |
| *TNF* | 7124 | TC6_cox_hap2000067.hg.1 | tumor necrosis factor; tumor necrosis factor (TNF superfamily, member 2) | 1.73 | 0.0000 | -1.35 | 0.0018 | 1.27 | 0.0001 | -1.27 | 0.0001 | -1.25 | 0.0000 | -1.06 | 0.7019 | 1.02 | 0.5330 | -1.33 | 0.0009 |
| *TNF* | 7124 | TC6_dbb_hap3000058.hg.1 | tumor necrosis factor; tumor necrosis factor (TNF superfamily, member 2) | 1.73 | 0.0000 | -1.35 | 0.0018 | 1.27 | 0.0001 | -1.27 | 0.0001 | -1.25 | 0.0000 | -1.06 | 0.7019 | 1.02 | 0.5330 | -1.33 | 0.0009 |
| *TNF* | 7124 | TC6_mann_hap4 000059.hg.1 | tumor necrosis factor; tumor necrosis factor (TNF superfamily, member 2) | 1.73 | 0.0000 | -1.35 | 0.0018 | 1.27 | 0.0001 | -1.27 | 0.0001 | -1.25 | 0.0000 | -1.06 | 0.7019 | 1.02 | 0.5330 | -1.33 | 0.0009 |
| *TNF* | 7124 | TC6_mcf_hap5000053.hg.1 | tumor necrosis factor; tumor necrosis factor (TNF superfamily, member 2) | 1.73 | 0.0000 | -1.35 | 0.0018 | 1.27 | 0.0001 | -1.27 | 0.0001 | -1.25 | 0.0000 | -1.06 | 0.7019 | 1.02 | 0.5330 | -1.33 | 0.0009 |
| *TNF* | 7124 | TC6_qbl_hap6000058.hg.1 | tumor necrosis factor; tumor necrosis factor (TNF superfamily, member 2) | 1.73 | 0.0000 | -1.35 | 0.0018 | 1.27 | 0.0001 | -1.27 | 0.0001 | -1.25 | 0.0000 | -1.06 | 0.7019 | 1.02 | 0.5330 | -1.33 | 0.0009 |
| *TNF* | 7124 | TC6_ssto_hap7000054.hg.1 | tumor necrosis factor; tumor necrosis factor (TNF superfamily, member 2) | 1.73 | 0.0000 | -1.35 | 0.0018 | 1.27 | 0.0001 | -1.27 | 0.0001 | -1.25 | 0.0000 | -1.06 | 0.7019 | 1.02 | 0.5330 | -1.33 | 0.0009 |
| *TPI1* | 7167 | TC12000096.hg.1 | triosephosphate isomerase 1 | 1.2 | 0.0000 | 1.03 | 0.3676 | 1.04 | 0.0662 | -1.04 | 0.0662 | 1.01 | 0.5112 | 1.07 | 0.0014 | 1.05 | 0.0151 | 1.08 | 0.0000 |
| *TNFRSF4* | 7293 | TC01002086.hg.1 | tumor necrosis factor receptor superfamily, member 4 | 1.36 | 0.0000 | -1.15 | 0.0029 | 1.18 | 0.0000 | -1.18 | 0.0000 | -1.16 | 0.0003 | 1.03 | 0.5189 | 1.02 | 0.7561 | -1.13 | 0.0094 |
| *VEGFA* | 7422 | TC06000608.hg.1 | vascular endothelial growth factor A | 1.03 | 0.0884 | -1.05 | 0.1235 | 1.13 | 0.0000 | -1.13 | 0.0000 | -1.11 | 0.0009 | 1.08 | 0.0236 | 1.02 | 0.6759 | -1.03 | 0.3078 |
| *VHL* | 7428 | TC03000055.hg.1 | von Hippel-Lindau tumor suppressor, E3 ubiquitin | -1.22 | 0.0000 | 1.12 | 0.0001 | -1.06 | 0.0165 | 1.06 | 0.0165 | 1.05 | 0.1168 | 1.05 | 0.1921 | -1.01 | 0.5285 | 1.1 | 0.0088 |

| | | | | | | | | | | | | | | | | | | | |
|---|---|---|---|---|---|---|---|---|---|---|---|---|---|---|---|---|---|---|---|
| | | | protein ligase; von Hippel-Lindau tumor suppressor | | | | | | | | | | | | | | | | |
| *VIM* | 7431 | TC10000126.hg.1 | vimentin | -1.51 | 0.0000 | 1.14 | 0.0047 | -1.09 | 0.0468 | 1.09 | 0.0468 | 1.05 | 0.1922 | 1.05 | 0.3702 | -1.04 | 0.2792 | 1.1 | 0.0262 |
| *CXCR4* | 7852 | TC02002378.hg.1 | chemokine (C-X-C motif) receptor 4 | -1.22 | 0.0001 | 1.18 | 0.0003 | -1.11 | 0.0019 | 1.11 | 0.0019 | 1.12 | 0.0011 | 1.06 | 0.0828 | 1 | 0.6486 | 1.18 | 0.0001 |
| *EOMES* | 8320 | TC03001257.hg.1 | eomesodermin; eomesodermin homolog (Xenopus laevis) | 1.09 | 0.0012 | -1.02 | 0.2570 | 1.09 | 0.0008 | -1.09 | 0.0008 | -1.08 | 0.0000 | 1.07 | 0.0133 | 1.01 | 0.7663 | -1.01 | 0.2230 |
| *CUL2* | 8453 | TC10001173.hg.1 | cullin 2 | -2.14 | 0.0000 | 1.26 | 0.0075 | -1.27 | 0.0011 | 1.27 | 0.0011 | 1.26 | 0.0165 | -1.01 | 0.8643 | -1.01 | 0.7330 | 1.25 | 0.0382 |
| *PIK3R3; RP11-322N21.2* | 8503 | TC01002616.hg.1 | phosphoinositide-3-kinase, regulatory subunit 3 (gamma); novel transcript | -2.14 | 0.0000 | 1.3 | 0.0000 | -1.16 | 0.0177 | 1.16 | 0.0177 | 1.13 | 0.0718 | 1.12 | 0.0978 | -1.03 | 0.6731 | 1.26 | 0.0018 |
| *MKNK1* | 8569 | TC01006353.hg.1 | MAP kinase interacting serine/threonine kinase 1 | -1.01 | 0.7755 | 1.02 | 0.3534 | 1.03 | 0.0170 | -1.03 | 0.0170 | -1.01 | 0.7298 | 1.06 | 0.0047 | 1.02 | 0.0555 | 1.04 | 0.0149 |
| *TNFRSF18* | 8784 | TC01002085.hg.1 | tumor necrosis factor receptor superfamily, member 18 | 1.28 | 0.0016 | -1.16 | 0.0209 | 1.15 | 0.0000 | -1.15 | 0.0000 | -1.15 | 0.0003 | -1.01 | 0.8419 | -1.01 | 0.7849 | -1.16 | 0.0147 |
| *NRP1* | 8829 | TC10001166.hg.1 | neuropilin 1 | 1.27 | 0.0000 | -1.07 | 0.0401 | 1.11 | 0.0011 | -1.11 | 0.0011 | -1.11 | 0.0005 | 1.03 | 0.5675 | 1 | 0.8226 | -1.07 | 0.0211 |
| *EIF4E2* | 9470 | TC02001404.hg.1 | eukaryotic translation initiation factor 4E family member 2 | 1 | 0.6744 | 1.11 | 0.0009 | 1.02 | 0.1767 | -1.02 | 0.1767 | 1.01 | 0.7412 | 1.14 | 0.0000 | 1.04 | 0.1067 | 1.15 | 0.0000 |
| *AKT3* | 10000 | TC01004040.hg.1 | v-akt murine thymoma viral oncogene homolog 3; v-akt murine thymoma viral oncogene homolog 3 (protein kinase B, gamma) | -1.4 | 0.0000 | 1.06 | 0.0012 | 1 | 0.9269 | -1 | 0.9269 | 1 | 0.9697 | 1.07 | 0.0079 | 1 | 0.9731 | 1.07 | 0.0226 |
| *TBX21* | 30009 | TC17000613.hg.1 | T-box 21 | 1.5 | 0.0000 | -1.15 | 0.0339 | 1.16 | 0.0002 | -1.16 | 0.0002 | -1.17 | 0.0002 | 1.01 | 0.4620 | -1.01 | 0.6881 | -1.16 | 0.0354 |
| *AK3* | 50808 | TC09000884.hg.1 | adenylate kinase 3 | -1.12 | 0.0003 | -1.01 | 0.1391 | -1.05 | 0.1641 | 1.05 | 0.1641 | 1.06 | 0.3283 | -1.06 | 0.5604 | 1 | 0.6987 | -1 | 0.2137 |
| *FOXP3* | 50943 | TC0X001031.hg.1 | forkhead box P3 | 1.34 | 0.0000 | -1.14 | 0.0044 | 1.19 | 0.0000 | -1.19 | 0.0000 | -1.2 | 0.0002 | 1.04 | 0.3106 | -1.01 | 0.7378 | -1.15 | 0.0176 |
| *ANGPT4* | 51378 | TC20000546.hg.1 | angiopoietin 4 | 1.21 | 0.0037 | -1.08 | 0.2102 | 1.17 | 0.0003 | -1.17 | 0.0003 | -1.2 | 0.0000 | 1.08 | 0.0746 | -1.03 | 0.4849 | -1.11 | 0.0764 |
| *EGLN1* | 54583 | TC01003968.hg.1 | egl-9 family hypoxia-inducible factor 1; egl nine homolog 1 (C. elegans) | -1.76 | 0.0000 | 1.27 | 0.0001 | -1.26 | 0.0009 | 1.26 | 0.0009 | 1.26 | 0.0043 | 1.01 | 0.9069 | -1 | 0.8381 | 1.26 | 0.0027 |
| *HIF1AN* | 55662 | TC10000724.hg.1 | hypoxia inducible factor 1, alpha subunit inhibitor | -1.18 | 0.0007 | 1.09 | 0.1383 | -1.07 | 0.1442 | 1.07 | 0.1442 | 1.16 | 0.0001 | 1.01 | 0.7623 | 1.08 | 0.0154 | 1.17 | 0.0004 |
| *HAMP* | 57817 | TC19000464.hg.1 | hepcidin antimicrobial peptide | 1.31 | 0.0002 | -1.06 | 0.1684 | 1.09 | 0.0500 | -1.09 | 0.0500 | -1.2 | 0.0005 | 1.03 | 0.6614 | -1.1 | 0.0369 | -1.17 | 0.0038 |
| *XPNPEP3; RBX1* | 63929 | TC22000327.hg.1 | X-prolyl aminopeptidase (aminopeptidase P) 3, putative; ring-box 1, E3 | -1.28 | 0.0000 | 1.11 | 0.0126 | -1.1 | 0.0081 | 1.1 | 0.0081 | 1.1 | 0.0285 | 1 | 0.6536 | 1 | 0.9469 | 1.11 | 0.0303 |

| | | | | | | | | | | | | | | | | | | | |
|---|---|---|---|---|---|---|---|---|---|---|---|---|---|---|---|---|---|---|---|
| | | | ubiquitin protein ligase; ring-box 1 | | | | | | | | | | | | | | | | |
| *HKDC1* | 80201 | TC10000418.hg.1 | hexokinase domain containing 1 | 1.27 | 0.0000 | -1.11 | 0.0024 | 1.11 | 0.0002 | -1.11 | 0.0002 | -1.1 | 0.0006 | -1 | 0.8566 | 1 | 0.9474 | -1.1 | 0.0038 |
| *PROK1* | 84432 | TC01000966.hg.1 | prokineticin 1 | 1.18 | 0.0003 | -1.07 | 0.1067 | 1.1 | 0.0154 | -1.1 | 0.0154 | -1.16 | 0.0001 | 1.03 | 0.6481 | -1.05 | 0.0082 | -1.13 | 0.0015 |
| *RETNLB* | 84666 | TC03001633.hg.1 | resistin like beta | 1.16 | 0.0000 | -1.03 | 0.2053 | 1.08 | 0.0216 | -1.08 | 0.0216 | -1.08 | 0.0083 | 1.05 | 0.1511 | 1 | 0.8914 | -1.03 | 0.1254 |
| *EGLN2* | 112398 | TC19002634.hg.1 | egl-9 family hypoxia-inducible factor 2 | 1.1 | 0.0002 | -1.02 | 0.1183 | 1.08 | 0.0121 | -1.08 | 0.0121 | -1.05 | 0.1822 | 1.06 | 0.4550 | 1.02 | 0.2401 | 1 | 0.6268 |
| *EGLN3* | 112399 | TC14001022.hg.1 | egl-9 family hypoxia-inducible factor 3; egl nine homolog 3 (C. elegans) | 1.09 | 0.0242 | -1.05 | 0.2165 | 1.04 | 0.0348 | -1.04 | 0.0348 | -1.03 | 0.2862 | -1 | 0.5082 | 1.01 | 0.2583 | -1.04 | 0.7854 |
| *EIF4E1B* | 253314 | TC05001004.hg.1 | eukaryotic translation initiation factor 4E family member 1B | 1.39 | 0.0000 | -1.19 | 0.0058 | 1.26 | 0.0007 | -1.26 | 0.0007 | -1.22 | 0.0008 | 1.06 | 0.9984 | 1.04 | 0.8332 | -1.15 | 0.0056 |
| *ENO4* | 387712 | TC10000848.hg.1 | enolase family member 4 | 1.21 | 0.0001 | -1.11 | 0.0556 | 1.13 | 0.0038 | -1.13 | 0.0038 | -1.13 | 0.0204 | 1.02 | 0.7158 | 1 | 0.7988 | -1.1 | 0.1128 |

**Supplementary Table S4.** Expression fold changes of 176 HIF-related genes represented by 375 probe set IDs analyzed in human U937 cells exposed to different gravitational conditions during parabolic flight (19th DLR PFC). The analysis was made with the NimbleGen expression microarray based on the hg18 annotation.

| | | | | 1g IF vs HW 1g GC | | BL-PFC hyp-g vs 1g IF | | µg vs BL-PFC hyp-g | | µg vs 1g IF | | BL-PFC hyp-g vs HW 1g GC | | µg vs HW 1g GC | |
|---|---|---|---|---|---|---|---|---|---|---|---|---|---|---|---|
| **Gene Symbol** | **Entrez Gene ID** | **Probe set ID** | **Description** | **Fold Change** | **p-value** | **Fold Change** | **p-value** | **Fold Change** | **p-value** | **Fold Change** | **p-value** | **Fold Change** | **p-value** | **Fold Change** | **p-value** |
| *ADM* | 133 | NM_001124 | adrenomedullin | 1.01 | 0.9409 | 1.23 | 0.3740 | -1.12 | 0.6665 | 1.09 | 0.7036 | 1.24 | 0.3316 | 1.11 | 0.6692 |
| *ADORA2A* | 135 | BC013780 | adenosine A2a receptor | -1.21 | 0.3323 | -1.20 | 0.3624 | 1.20 | 0.5133 | 1.00 | 0.9915 | -1.45 | 0.1532 | -1.21 | 0.4587 |
| *ADORA2A* | 135 | NM_000675 | adenosine A2a receptor | 1.00 | 0.9801 | 1.14 | 0.2984 | -1.03 | 0.8496 | 1.11 | 0.4027 | 1.14 | 0.4110 | 1.11 | 0.4987 |
| *ADRA1B* | 147 | NM_000679 | adrenergic, alpha-1B-, receptor | 1.13 | 0.5589 | 1.73 | 0.0579 | -1.70 | 0.0626 | 1.01 | 0.9420 | 1.96 | 0.0510 | 1.15 | 0.5132 |
| *AKT1* | 207 | BC084538 | v-akt murine thymoma viral oncogene homolog 1 | -1.01 | 0.9151 | -1.25 | 0.1093 | 1.17 | 0.3563 | -1.06 | 0.5676 | -1.26 | 0.2555 | -1.08 | 0.6396 |
| *AKT1* | 207 | BX647722 | v-akt murine thymoma viral oncogene homolog 1 | 1.34 | 0.0177 | -1.27 | 0.0851 | 1.55 | 0.0043 | 1.22 | 0.0853 | 1.06 | 0.5971 | 1.64 | 0.0007 |
| *AKT1* | 207 | NM_001014431 | v-akt murine thymoma viral oncogene homolog 1 | 1.10 | 0.2272 | -1.27 | 0.0155 | 1.20 | 0.0585 | -1.06 | 0.1661 | -1.15 | 0.3048 | 1.04 | 0.6174 |
| *AKT2* | 208 | NM_001626 | v-akt murine thymoma viral oncogene homolog 2 | -1.04 | 0.8233 | -1.81 | 0.0637 | 1.27 | 0.5192 | -1.43 | 0.0686 | -1.89 | 0.1060 | -1.49 | 0.1082 |
| *ALDOA* | 226 | CR592372 | aldolase A, fructose-bisphosphate | -1.05 | 0.4004 | 1.03 | 0.6182 | -1.01 | 0.8904 | 1.02 | 0.6601 | -1.02 | 0.8011 | -1.03 | 0.6504 |
| *ALDOA* | 226 | NM_000034 | aldolase A, fructose-bisphosphate | -1.06 | 0.1865 | 1.04 | 0.3970 | -1.00 | 0.9408 | 1.03 | 0.5574 | -1.02 | 0.6233 | -1.02 | 0.6774 |
| *ALDOA* | 226 | NM_184041 | aldolase A, fructose-bisphosphate | -1.02 | 0.7502 | -1.01 | 0.8483 | 1.01 | 0.8701 | 1.00 | 0.9915 | -1.03 | 0.6717 | -1.02 | 0.8065 |
| *ALDOC* | 230 | BC106925 | aldolase C, fructose-bisphosphate | 1.95 | 0.0002 | -1.27 | 0.1561 | 1.13 | 0.5368 | -1.12 | 0.3756 | 1.54 | 0.0980 | 1.75 | 0.0107 |
| *ALDOC* | 230 | NM_005165 | aldolase C, fructose-bisphosphate | 1.39 | 0.0304 | -1.14 | 0.4131 | 1.03 | 0.8539 | -1.11 | 0.4140 | 1.22 | 0.3167 | 1.25 | 0.1277 |
| *ANGPT1* | 284 | BC029406 | angiopoietin 1 | -1.09 | 0.3194 | 1.04 | 0.0516 | -1.02 | 0.4889 | 1.02 | 0.3691 | -1.04 | 0.6669 | -1.07 | 0.4650 |
| *ANGPT1* | 284 | NM_001146 | angiopoietin 1 | -1.04 | 0.2616 | 1.03 | 0.2645 | -1.02 | 0.5536 | 1.01 | 0.7303 | -1.01 | 0.7873 | -1.03 | 0.4492 |
| *ANGPT2* | 285 | AF187858 | angiopoietin 2 | -1.01 | 0.8564 | 1.24 | 0.0270 | -1.16 | 0.1058 | 1.07 | 0.2674 | 1.22 | 0.0711 | 1.06 | 0.4407 |
| *ANGPT2* | 285 | AF218015 | angiopoietin 2 | -1.01 | 0.6916 | 1.13 | 0.0222 | -1.06 | 0.2378 | 1.06 | 0.0767 | 1.12 | 0.0482 | 1.05 | 0.1361 |
| *ANGPT2* | 285 | NM_001147 | angiopoietin 2 | -1.02 | 0.5353 | 1.13 | 0.0254 | -1.14 | 0.0274 | -1.01 | 0.8845 | 1.11 | 0.0603 | -1.03 | 0.4943 |
| *ARG1* | 383 | BC020653 | arginase, liver | -1.27 | 0.1391 | 1.00 | 0.9832 | -1.01 | 0.9127 | -1.00 | 0.9667 | -1.27 | 0.1615 | -1.28 | 0.0974 |
| *ARG1* | 383 | NM_000045 | arginase, liver | -1.13 | 0.0401 | 1.09 | 0.0252 | -1.12 | 0.0107 | -1.03 | 0.4637 | -1.04 | 0.5046 | -1.17 | 0.0204 |

| | | | | | | | | | | | | | | | |
|---|---|---|---|---|---|---|---|---|---|---|---|---|---|---|---|
| *ARNT* | 405 | AB209877 | aryl hydrocarbon receptor nuclear translocator | 1.11 | 0.2563 | -1.42 | 0.0217 | 1.23 | 0.2072 | -1.15 | 0.1341 | -1.28 | 0.1597 | -1.04 | 0.7269 |
| *ARNT* | 405 | BC060838 | aryl hydrocarbon receptor nuclear translocator | -1.39 | 0.1168 | -1.27 | 0.2909 | 1.13 | 0.6821 | -1.12 | 0.5956 | -1.76 | 0.0501 | -1.56 | 0.0896 |
| *ARNT* | 405 | NM_001668 | aryl hydrocarbon receptor nuclear translocator | 1.05 | 0.7394 | -1.43 | 0.0769 | 1.08 | 0.7037 | -1.33 | 0.1425 | -1.36 | 0.0601 | -1.26 | 0.1941 |
| *BCL2* | 596 | BC027258 | B-cell CLL/lymphoma 2 | -1.48 | 0.1130 | -1.69 | 0.0877 | 1.20 | 0.2581 | -1.40 | 0.1689 | -2.50 | 0.0028 | -2.08 | 0.0028 |
| *BCL2* | 596 | NM_000633 | B-cell CLL/lymphoma 2 | -2.31 | 0.0156 | -2.23 | 0.0906 | 1.22 | 0.4035 | -1.83 | 0.1132 | -5.16 | 0.0014 | -4.23 | 0.0005 |
| *BCL2* | 596 | NM_000657 | B-cell CLL/lymphoma 2 | 1.17 | 0.3200 | 1.33 | 0.0578 | -1.17 | 0.2635 | 1.13 | 0.4215 | 1.56 | 0.0045 | 1.33 | 0.0907 |
| *BLR1* | 643 | NM_001716 | Burkitt lymphoma receptor 1, GTP binding protein (chemokine (C-X-C motif) receptor 5) | 1.52 | 0.1012 | 1.70 | 0.0579 | -1.35 | 0.2550 | 1.26 | 0.2735 | 2.60 | 0.0194 | 1.92 | 0.0318 |
| *CA9* | 768 | NM_001216 | carbonic anhydrase IX | 1.08 | 0.5647 | 1.85 | 0.0214 | -1.53 | 0.0975 | 1.21 | 0.2113 | 2.00 | 0.0360 | 1.31 | 0.1705 |
| *CAMK2A* | 815 | AF145710 | calcium/calmodulin-dependent protein kinase (CaM kinase) II alpha | 1.30 | 0.2728 | 1.30 | 0.2290 | -1.01 | 0.9468 | 1.28 | 0.2432 | 1.70 | 0.0605 | 1.67 | 0.0628 |
| *CAMK2A* | 815 | AF145711 | calcium/calmodulin-dependent protein kinase (CaM kinase) II alpha | 1.33 | 0.2031 | 1.44 | 0.1267 | -1.17 | 0.5103 | 1.23 | 0.3265 | 1.91 | 0.0437 | 1.63 | 0.0778 |
| *CAMK2A* | 815 | NM_015981 | calcium/calmodulin-dependent protein kinase (CaM kinase) II alpha | -1.02 | 0.7554 | 1.20 | 0.0142 | -1.16 | 0.0158 | 1.03 | 0.6808 | 1.17 | 0.0336 | 1.00 | 0.9626 |
| *CAMK2B* | 816 | NM_001220 | calcium/calmodulin-dependent protein kinase (CaM kinase) II beta | 1.12 | 0.6263 | 1.93 | 0.0262 | -1.37 | 0.2790 | 1.41 | 0.1680 | 2.17 | 0.0483 | 1.58 | 0.1750 |
| *CAMK2B* | 816 | NM_172078 | calcium/calmodulin-dependent protein kinase (CaM kinase) II beta | 1.16 | 0.5120 | 1.90 | 0.0117 | -1.44 | 0.1009 | 1.32 | 0.1535 | 2.19 | 0.0121 | 1.53 | 0.0721 |

| | | | | | | | | | | | | | | | |
|---|---|---|---|---|---|---|---|---|---|---|---|---|---|---|---|
| *CAMK2B* | 816 | NM_172080 | calcium/calmodulin-dependent protein kinase (CaM kinase) II beta | 1.10 | 0.7217 | 2.12 | 0.0308 | -1.71 | 0.0942 | 1.24 | 0.3941 | 2.34 | 0.0414 | 1.37 | 0.2842 |
| *CAMK2B* | 816 | NM_172081 | calcium/calmodulin-dependent protein kinase (CaM kinase) II beta | 1.04 | 0.8516 | 1.99 | 0.0051 | -1.53 | 0.0393 | 1.30 | 0.1536 | 2.07 | 0.0102 | 1.36 | 0.1455 |
| *CAMK2B* | 816 | NM_172082 | calcium/calmodulin-dependent protein kinase (CaM kinase) II beta | -1.07 | 0.6950 | 1.99 | 0.0028 | -1.39 | 0.1168 | 1.43 | 0.0654 | 1.86 | 0.0166 | 1.34 | 0.1915 |
| *CAMK2B* | 816 | NM_172084 | calcium/calmodulin-dependent protein kinase (CaM kinase) II beta | 1.18 | 0.4627 | 1.81 | 0.0560 | -1.31 | 0.3583 | 1.38 | 0.1381 | 2.14 | 0.0648 | 1.64 | 0.1017 |
| *CAMK2D* | 817 | NM_172115 | calcium/calmodulin-dependent protein kinase (CaM kinase) II delta | -1.07 | 0.7436 | -2.09 | 0.0075 | 1.38 | 0.2177 | -1.52 | 0.0341 | -2.23 | 0.0197 | -1.62 | 0.0500 |
| *CAMK2D* | 817 | AB209288 | calcium/calmodulin-dependent protein kinase (CaM kinase) II delta | -1.17 | 0.1613 | -1.06 | 0.4230 | -1.06 | 0.0963 | -1.12 | 0.0838 | -1.24 | 0.0795 | -1.31 | 0.0165 |
| *CAMK2D* | 817 | NM_001221 | calcium/calmodulin-dependent protein kinase (CaM kinase) II delta | -1.49 | 0.1749 | -2.04 | 0.0736 | 1.23 | 0.3845 | -1.65 | 0.1226 | -3.05 | 0.0043 | -2.47 | 0.0046 |
| *CAMK2D* | 817 | NM_172127 | calcium/calmodulin-dependent protein kinase (CaM kinase) II delta | -1.36 | 0.3856 | -2.55 | 0.0797 | 1.35 | 0.1934 | -1.88 | 0.1143 | -3.46 | 0.0084 | -2.56 | 0.0082 |
| *CAMK2G* | 818 | NM_001222 | calcium/calmodulin-dependent protein kinase (CaM kinase) II gamma | -1.05 | 0.4747 | -1.32 | 0.0009 | 1.17 | 0.0047 | -1.13 | 0.0097 | -1.39 | 0.0026 | -1.19 | 0.0094 |
| *CAMP* | 820 | BC055089 | cathelicidin antimicrobial peptide | -1.38 | 0.1018 | 1.25 | 0.0675 | -1.32 | 0.0137 | -1.06 | 0.5241 | -1.10 | 0.6200 | -1.46 | 0.0539 |
| *CAMP* | 820 | NM_004345 | cathelicidin antimicrobial peptide | 1.21 | 0.5203 | 1.11 | 0.6768 | 1.28 | 0.2995 | 1.42 | 0.1627 | 1.34 | 0.2541 | 1.71 | 0.0642 |

| | | | | | | | | | | | | | | | |
|---|---|---|---|---|---|---|---|---|---|---|---|---|---|---|---|
| *CD36* | 948 | BC008406 | CD36 molecule (thrombospondin receptor) | -1.10 | 0.3529 | -1.23 | 0.1058 | 1.16 | 0.3055 | -1.06 | 0.5856 | -1.36 | 0.0549 | -1.17 | 0.2201 |
| *CD36* | 948 | NM_000072 | CD36 molecule (thrombospondin receptor) | -1.17 | 0.2902 | -1.54 | 0.0430 | 1.30 | 0.2779 | -1.18 | 0.3266 | -1.80 | 0.0168 | -1.38 | 0.0890 |
| *CD36* | 948 | NM_001001548 | CD36 molecule (thrombospondin receptor) | 1.08 | 0.2608 | -1.35 | 0.0089 | 1.22 | 0.1559 | -1.10 | 0.2734 | -1.24 | 0.0910 | -1.02 | 0.8693 |
| *CDKN1A* | 1026 | NM_000389 | cyclin-dependent kinase inhibitor 1A (p21, Cip1) | 1.08 | 0.6638 | 1.44 | 0.0838 | -1.21 | 0.4215 | 1.19 | 0.3315 | 1.55 | 0.1162 | 1.29 | 0.2996 |
| *CDKN1A* | 1026 | NM_078467 | cyclin-dependent kinase inhibitor 1A (p21, Cip1) | 1.04 | 0.8425 | 1.48 | 0.0538 | -1.23 | 0.3787 | 1.20 | 0.3583 | 1.54 | 0.1127 | 1.25 | 0.4004 |
| *CDKN1B* | 1027 | NM_004064 | cyclin-dependent kinase inhibitor 1B (p27, Kip1) | -1.83 | 0.0712 | -3.53 | 0.0522 | 1.33 | 0.4473 | -2.66 | 0.0466 | -6.46 | 0.0008 | -4.86 | 0.0002 |
| *CCR1* | 1230 | BC051306 | chemokine (C-C motif) receptor 1 | -2.56 | 0.0098 | 1.13 | 0.6029 | -1.07 | 0.8160 | 1.06 | 0.8058 | -2.26 | 0.0438 | -2.42 | 0.0169 |
| *CCR1* | 1230 | NM_001295 | chemokine (C-C motif) receptor 1 | -1.85 | 0.0034 | 1.10 | 0.5467 | -1.07 | 0.7477 | 1.03 | 0.8567 | -1.68 | 0.0357 | -1.80 | 0.0114 |
| *CCR5* | 1234 | BC038398 | chemokine (C-C motif) receptor 5 | 1.47 | 0.2190 | 1.90 | 0.1060 | -1.56 | 0.2516 | 1.22 | 0.4895 | 2.80 | 0.0630 | 1.80 | 0.1358 |
| *CCR5* | 1234 | NM_000579 | chemokine (C-C motif) receptor 5 | 1.58 | 0.1852 | 2.26 | 0.0648 | -1.71 | 0.1916 | 1.32 | 0.3439 | 3.56 | 0.0477 | 2.09 | 0.0929 |
| *CCR7* | 1236 | NM_001838 | chemokine (C-C motif) receptor 7 | 1.32 | 0.3311 | 1.88 | 0.0374 | -1.72 | 0.0510 | 1.10 | 0.6205 | 2.48 | 0.0263 | 1.45 | 0.1354 |
| *CREBBP* | 1387 | NM_004380 | CREB binding protein (Rubinstein-Taybi syndrome) | -2.36 | 0.0282 | -1.78 | 0.1712 | 1.03 | 0.8914 | -1.73 | 0.1308 | -4.21 | 0.0082 | -4.09 | 0.0026 |
| *CREBBP* | 1387 | U85962 | CREB binding protein (Rubinstein-Taybi syndrome) | -1.40 | 0.2561 | -1.41 | 0.4022 | -1.00 | 0.9959 | -1.41 | 0.3159 | -1.97 | 0.0674 | -1.97 | 0.0290 |
| *MAPK14* | 1432 | BC000092 | mitogen-activated protein kinase 14 | -1.07 | 0.3785 | -1.43 | 0.0008 | 1.20 | 0.1658 | -1.20 | 0.0320 | -1.53 | 0.0040 | -1.28 | 0.0324 |
| *MAPK14* | 1432 | NM_001315 | mitogen-activated protein kinase 14 | -1.16 | 0.1219 | -1.11 | 0.3444 | 1.04 | 0.7182 | -1.07 | 0.5068 | -1.29 | 0.0324 | -1.24 | 0.0383 |
| *MAPK14* | 1432 | NM_139013 | mitogen-activated protein kinase 14 | -1.12 | 0.2742 | -1.68 | 0.0042 | 1.42 | 0.0644 | -1.18 | 0.1029 | -1.88 | 0.0055 | -1.32 | 0.0358 |

| | | | | | | | | | | | | | | | |
|---|---|---|---|---|---|---|---|---|---|---|---|---|---|---|---|
| *CTLA4* | 1493 | BC069566 | cytotoxic T-lymphocyte-associated protein 4 | -1.04 | 0.6862 | 1.35 | 0.0167 | -1.18 | 0.0397 | 1.14 | 0.1240 | 1.29 | 0.0199 | 1.09 | 0.1034 |
| *CTLA4* | 1493 | BC074842 | cytotoxic T-lymphocyte-associated protein 4 | 1.08 | 0.7404 | 1.53 | 0.1268 | -1.20 | 0.4551 | 1.27 | 0.2081 | 1.65 | 0.1405 | 1.37 | 0.1737 |
| *CTLA4* | 1493 | BC074893 | cytotoxic T-lymphocyte-associated protein 4 | 1.05 | 0.5381 | 1.57 | 0.0088 | -1.39 | 0.0622 | 1.13 | 0.2932 | 1.64 | 0.0151 | 1.18 | 0.2161 |
| *CTLA4* | 1493 | NM_001037631 | cytotoxic T-lymphocyte-associated protein 4 | 1.30 | 0.3718 | 1.59 | 0.1322 | -1.35 | 0.2564 | 1.17 | 0.3565 | 2.07 | 0.0890 | 1.53 | 0.0978 |
| *CTLA4* | 1493 | NM_005214 | cytotoxic T-lymphocyte-associated protein 4 | 1.32 | 0.2652 | 1.35 | 0.2779 | -1.38 | 0.2407 | -1.02 | 0.9326 | 1.78 | 0.0930 | 1.29 | 0.2643 |
| *CTSD* | 1509 | NM_001909 | cathepsin D (lysosomal aspartyl peptidase) | 1.06 | 0.3334 | -1.07 | 0.2629 | 1.08 | 0.1915 | 1.01 | 0.8594 | -1.01 | 0.8724 | 1.06 | 0.2454 |
| *CYBB* | 1536 | BC032720 | cytochrome b-245, beta polypeptide (chronic granulomatous disease) | -1.00 | 0.9815 | -1.82 | 0.0044 | 1.37 | 0.1290 | -1.33 | 0.0337 | -1.82 | 0.0166 | -1.34 | 0.0755 |
| *CYBB* | 1536 | NM_000397 | cytochrome b-245, beta polypeptide (chronic granulomatous disease) | 1.01 | 0.9481 | -1.81 | 0.0039 | 1.36 | 0.1587 | -1.33 | 0.0569 | -1.79 | 0.0115 | -1.32 | 0.1024 |
| *DEFB1* | 1672 | BC033298 | defensin, beta 1 | -1.10 | 0.6707 | -1.18 | 0.3807 | 1.05 | 0.7184 | -1.12 | 0.5358 | -1.30 | 0.1961 | -1.23 | 0.2966 |
| *DEFB1* | 1672 | NM_005218 | defensin, beta 1 | 1.04 | 0.8350 | 1.93 | 0.0048 | -1.64 | 0.0273 | 1.18 | 0.2694 | 2.00 | 0.0181 | 1.22 | 0.3376 |
| *EDG1* | 1901 | NM_001400 | endothelial differentiation, sphingolipid G-protein-coupled receptor, 1 | 1.45 | 0.2599 | 1.52 | 0.1829 | -1.19 | 0.5994 | 1.28 | 0.3522 | 2.20 | 0.0969 | 1.85 | 0.1274 |
| *EDN1* | 1906 | CR605456 | endothelin 1 | 1.05 | 0.3114 | 1.13 | 0.0900 | -1.08 | 0.3286 | 1.05 | 0.3705 | 1.19 | 0.0584 | 1.10 | 0.1399 |
| *EDN1* | 1906 | NM_001955 | endothelin 1 | -1.07 | 0.3919 | 1.02 | 0.7668 | -1.02 | 0.7959 | 1.00 | 0.9943 | -1.04 | 0.5383 | -1.07 | 0.4448 |
| *EGF* | 1950 | BC093731 | epidermal growth factor (beta-urogastrone) | 1.14 | 0.2912 | 1.05 | 0.6618 | 1.02 | 0.8801 | 1.08 | 0.5626 | 1.20 | 0.1625 | 1.22 | 0.1662 |
| *EGF* | 1950 | NM_001963 | epidermal growth factor (beta-urogastrone) | 1.05 | 0.5322 | 1.01 | 0.8358 | -1.03 | 0.4217 | -1.01 | 0.8277 | 1.06 | 0.0504 | 1.03 | 0.3869 |
| *EGFR* | 1956 | BC094761 | epidermal growth factor receptor (erythroblastic leukemia viral (v-erb-b) oncogene hom | 1.11 | 0.5427 | 1.45 | 0.0598 | -1.13 | 0.5544 | 1.29 | 0.1746 | 1.61 | 0.0469 | 1.43 | 0.1116 |

| | | | | | | | | | | | | | | | |
|---|---|---|---|---|---|---|---|---|---|---|---|---|---|---|---|
| *EGFR* | 1956 | K03193 | epidermal growth factor receptor (erythroblastic leukemia viral (v-erb-b) oncogene hom | 1.06 | 0.1343 | 1.14 | 0.0286 | -1.08 | 0.2774 | 1.06 | 0.2824 | 1.21 | 0.0174 | 1.11 | 0.0941 |
| *EGFR* | 1956 | NM_005228 | epidermal growth factor receptor (erythroblastic leukemia viral (v-erb-b) oncogene hom | -1.13 | 0.3647 | 1.11 | 0.3581 | -1.09 | 0.3190 | 1.01 | 0.8892 | -1.02 | 0.8922 | -1.11 | 0.3509 |
| *EGFR* | 1956 | NM_201282 | epidermal growth factor receptor (erythroblastic leukemia viral (v-erb-b) oncogene hom | -1.08 | 0.3561 | 1.34 | 0.0037 | -1.12 | 0.3312 | 1.20 | 0.0958 | 1.25 | 0.0292 | 1.12 | 0.3419 |
| *EGFR* | 1956 | NM_201283 | epidermal growth factor receptor (erythroblastic leukemia viral (v-erb-b) oncogene hom | 1.44 | 0.2674 | 1.73 | 0.0295 | -1.53 | 0.0799 | 1.13 | 0.5780 | 2.49 | 0.0178 | 1.62 | 0.1510 |
| *EGFR* | 1956 | NM_201284 | epidermal growth factor receptor (erythroblastic leukemia viral (v-erb-b) oncogene hom | 1.11 | 0.5697 | -1.02 | 0.8874 | 1.02 | 0.8701 | 1.01 | 0.9590 | 1.09 | 0.6609 | 1.12 | 0.5846 |
| *EIF4E* | 1977 | BC012611 | eukaryotic translation initiation factor 4E | -1.00 | 0.9849 | 1.03 | 0.8557 | -1.09 | 0.6823 | -1.05 | 0.7668 | 1.03 | 0.8738 | -1.05 | 0.7638 |
| *EIF4E* | 1977 | BC035166 | eukaryotic translation initiation factor 4E | 1.34 | 0.3613 | 1.05 | 0.8771 | -1.18 | 0.4154 | -1.12 | 0.6704 | 1.41 | 0.0911 | 1.19 | 0.3120 |
| *EIF4E* | 1977 | NM_001968 | eukaryotic translation initiation factor 4E | 1.07 | 0.5726 | -1.01 | 0.9600 | -1.10 | 0.5876 | -1.10 | 0.4417 | 1.06 | 0.7162 | -1.03 | 0.8495 |
| *EIF4EBP1* | 1978 | BC058073 | eukaryotic translation initiation factor 4E binding protein 1 | -1.02 | 0.6951 | -1.04 | 0.3557 | 1.05 | 0.1967 | 1.01 | 0.8635 | -1.06 | 0.2557 | -1.01 | 0.7451 |
| *EIF4EBP1* | 1978 | NM_004095 | eukaryotic translation initiation factor 4E binding protein 1 | 1.02 | 0.7394 | -1.03 | 0.4968 | 1.02 | 0.6644 | -1.01 | 0.7546 | -1.01 | 0.8939 | 1.01 | 0.8935 |
| *ENG* | 2022 | BC014271 | endoglin (Osler-Rendu-Weber syndrome 1) | -1.03 | 0.7002 | -1.31 | 0.0139 | 1.16 | 0.2131 | -1.13 | 0.0384 | -1.35 | 0.0528 | -1.16 | 0.1187 |

| | | | | | | | | | | | | | |
|---|---|---|---|---|---|---|---|---|---|---|---|---|---|
| *ENG* | 2022 | NM_000118 | endoglin (Osler-Rendu-Weber syndrome 1) | -1.05 | 0.5565 | -1.26 | 0.0297 | 1.17 | 0.1850 | -1.08 | 0.2513 | -1.33 | 0.0627 | -1.14 | 0.2146 |
| *ENO1* | 2023 | BC050642 | enolase 1, (alpha) | -1.00 | 0.9537 | -1.10 | 0.2549 | 1.10 | 0.1951 | 1.01 | 0.8844 | -1.10 | 0.3184 | 1.00 | 0.9529 |
| *ENO1* | 2023 | BC073991 | enolase 1, (alpha) | 1.02 | 0.7657 | -1.07 | 0.3733 | 1.06 | 0.4620 | -1.01 | 0.8114 | -1.05 | 0.5396 | 1.00 | 0.9361 |
| *ENO1* | 2023 | NM_001428 | enolase 1, (alpha) | -1.01 | 0.9078 | -1.08 | 0.3456 | 1.04 | 0.5580 | -1.03 | 0.5262 | -1.09 | 0.3853 | -1.04 | 0.5169 |
| *ENO2* | 2026 | NM_001975 | enolase 2 (gamma, neuronal) | 1.13 | 0.3610 | -1.08 | 0.4857 | 1.06 | 0.6507 | -1.02 | 0.8707 | 1.05 | 0.6609 | 1.11 | 0.4809 |
| *ENO3* | 2027 | BC017249 | enolase 3 (beta, muscle) | -1.22 | 0.1968 | -1.21 | 0.2379 | 1.05 | 0.7618 | -1.15 | 0.3770 | -1.48 | 0.0177 | -1.41 | 0.0396 |
| *ENO3* | 2027 | NM_001976 | enolase 3 (beta, muscle) | -1.20 | 0.2708 | -1.28 | 0.1563 | 1.03 | 0.8546 | -1.24 | 0.2516 | -1.54 | 0.0120 | -1.49 | 0.0373 |
| *ENO3* | 2027 | NM_053013 | enolase 3 (beta, muscle) | -1.22 | 0.2250 | -1.24 | 0.1781 | 1.02 | 0.8835 | -1.22 | 0.2520 | -1.52 | 0.0209 | -1.48 | 0.0349 |
| *EP300* | 2033 | NM_001429 | E1A binding protein p300 | -1.14 | 0.0992 | -1.04 | 0.7700 | 1.02 | 0.9111 | -1.02 | 0.8336 | -1.19 | 0.2764 | -1.17 | 0.1953 |
| *EPAS1* | 2034 | NM_001430 | endothelial PAS domain protein 1 | -1.18 | 0.3113 | 1.11 | 0.4721 | -1.01 | 0.9563 | 1.10 | 0.6165 | -1.06 | 0.7439 | -1.08 | 0.7383 |
| *EPO* | 2056 | BC093628 | erythropoietin | 1.16 | 0.6579 | 1.24 | 0.3969 | -1.09 | 0.7264 | 1.14 | 0.5348 | 1.44 | 0.3272 | 1.32 | 0.3731 |
| *EPO* | 2056 | NM_000799 | erythropoietin | 1.02 | 0.8937 | 1.67 | 0.0030 | -1.33 | 0.0748 | 1.25 | 0.1308 | 1.71 | 0.0133 | 1.28 | 0.1944 |
| *ERBB2* | 2064 | AF177761 | v-erb-b2 erythroblastic leukemia viral oncogene homolog 2, neuro/glioblastoma derived | 1.31 | 0.3908 | 1.82 | 0.0315 | -1.39 | 0.1632 | 1.31 | 0.2200 | 2.38 | 0.0212 | 1.72 | 0.0692 |
| *ERBB2* | 2064 | AK131568 | v-erb-b2 erythroblastic leukemia viral oncogene homolog 2, neuro/glioblastoma derived | 1.07 | 0.7316 | 1.36 | 0.1967 | 1.05 | 0.8223 | 1.43 | 0.0630 | 1.46 | 0.1720 | 1.53 | 0.0532 |
| *ERBB2* | 2064 | NM_001005862 | v-erb-b2 erythroblastic leukemia viral oncogene homolog 2, neuro/glioblastoma derived | -1.27 | 0.0396 | 1.18 | 0.2721 | -1.20 | 0.2502 | -1.02 | 0.8699 | -1.07 | 0.6339 | -1.29 | 0.0429 |
| *FLT1* | 2321 | BC039007 | fms-related tyrosine kinase 1 (vascular endothelial growth factor/vascular permeabilit | 1.05 | 0.6351 | 1.14 | 0.3071 | -1.31 | 0.0835 | -1.14 | 0.1990 | 1.20 | 0.2578 | -1.09 | 0.5120 |
| *FLT1* | 2321 | NM_002019 | fms-related tyrosine kinase 1 (vascular | 1.15 | 0.4813 | 2.21 | 0.0251 | -1.69 | 0.1040 | 1.30 | 0.2317 | 2.53 | 0.0336 | 1.49 | 0.1337 |

| | | | | | | | | | | | | | | | |
|---|---|---|---|---|---|---|---|---|---|---|---|---|---|---|---|
| | | | endothelial growth factor/vascular permeabilit | | | | | | | | | | | | |
| *FN1* | 2335 | AB191261 | fibronectin 1 | -1.16 | 0.1226 | -1.06 | 0.1542 | 1.04 | 0.4682 | -1.02 | 0.6288 | -1.23 | 0.0819 | -1.18 | 0.0978 |
| *FN1* | 2335 | BX538018 | fibronectin 1 | -1.08 | 0.4138 | -1.10 | 0.2151 | -1.01 | 0.8930 | -1.11 | 0.1724 | -1.19 | 0.0677 | -1.20 | 0.0478 |
| *FN1* | 2335 | CR749317 | fibronectin 1 | -1.14 | 0.0687 | -1.04 | 0.4158 | -1.00 | 0.9760 | -1.04 | 0.4125 | -1.19 | 0.0307 | -1.19 | 0.0213 |
| *FN1* | 2335 | NM_002026 | fibronectin 1 | -1.16 | 0.2740 | -1.20 | 0.1243 | 1.07 | 0.4425 | -1.13 | 0.2736 | -1.39 | 0.0202 | -1.30 | 0.0367 |
| *FN1* | 2335 | NM_054034 | fibronectin 1 | 1.02 | 0.8394 | -1.26 | 0.0821 | 1.02 | 0.7382 | -1.23 | 0.0501 | -1.23 | 0.0555 | -1.20 | 0.0199 |
| *FRAP1* | 2475 | NM_004958 | FK506 binding protein 12-rapamycin associated protein 1 | -1.37 | 0.0005 | -1.26 | 0.0691 | 1.09 | 0.4506 | -1.16 | 0.0987 | -1.74 | 0.0001 | -1.59 | 0.0000 |
| *GAPDH* | 2597 | BC001601 | glyceraldehyde-3-phosphate dehydrogenase | 1.04 | 0.4602 | -1.13 | 0.1002 | 1.09 | 0.2572 | -1.03 | 0.5363 | -1.08 | 0.3696 | 1.01 | 0.8728 |
| *GAPDH* | 2597 | BC009081 | glyceraldehyde-3-phosphate dehydrogenase | 1.01 | 0.8921 | -1.07 | 0.4897 | 1.10 | 0.3183 | 1.03 | 0.6795 | -1.06 | 0.6160 | 1.04 | 0.6181 |
| *GAPDH* | 2597 | NM_002046 | glyceraldehyde-3-phosphate dehydrogenase | 1.00 | 0.9962 | -1.10 | 0.1673 | 1.04 | 0.4709 | -1.05 | 0.1961 | -1.10 | 0.3360 | -1.05 | 0.4367 |
| *GPI* | 2821 | BC004982 | glucose phosphate isomerase | -1.00 | 0.9270 | -1.13 | 0.0641 | 1.08 | 0.2458 | -1.04 | 0.2232 | -1.13 | 0.1220 | -1.05 | 0.3118 |
| *GPI* | 2821 | NM_000175 | glucose phosphate isomerase | 1.00 | 0.9316 | -1.08 | 0.3015 | 1.05 | 0.5181 | -1.02 | 0.5507 | -1.07 | 0.4662 | -1.02 | 0.7502 |
| *MKNK2* | 2872 | AF237775 | MAP kinase interacting serine/threonine kinase 2 | -1.07 | 0.6112 | -1.21 | 0.3929 | 1.10 | 0.7181 | -1.11 | 0.5080 | -1.30 | 0.3047 | -1.19 | 0.3196 |
| *MKNK2* | 2872 | NM_017572 | MAP kinase interacting serine/threonine kinase 2 | -1.01 | 0.9320 | -1.19 | 0.4504 | 1.06 | 0.8385 | -1.13 | 0.4794 | -1.20 | 0.4920 | -1.14 | 0.5056 |
| *MKNK2* | 2872 | NM_199054 | MAP kinase interacting serine/threonine kinase 2 | 1.21 | 0.2247 | 1.11 | 0.4760 | 1.04 | 0.8260 | 1.15 | 0.3371 | 1.35 | 0.1343 | 1.40 | 0.0926 |
| *CXCL2* | 2920 | BC015753 | chemokine (C-X-C motif) ligand 2 | -1.42 | 0.0062 | 1.22 | 0.2159 | 1.00 | 0.9858 | 1.22 | 0.0168 | -1.17 | 0.3601 | -1.17 | 0.1260 |
| *CXCL2* | 2920 | NM_002089 | chemokine (C-X-C motif) ligand 2 | -1.61 | 0.1039 | 1.04 | 0.5563 | 1.15 | 0.4935 | 1.19 | 0.3159 | -1.55 | 0.1909 | -1.35 | 0.3232 |
| *HDAC2* | 3066 | NM_001527 | histone deacetylase 2 | -1.04 | 0.4793 | -1.13 | 0.0678 | 1.00 | 0.9841 | -1.13 | 0.1101 | -1.17 | 0.0712 | -1.17 | 0.0916 |

| | | | | | | | | | | | | | |
|---|---|---|---|---|---|---|---|---|---|---|---|---|---|
| *HIF1A* | 3091 | NM_001530 | hypoxia-inducible factor 1, alpha subunit (basic helix-loop-helix transcription factor | -1.30 | 0.1998 | -1.46 | 0.1736 | 1.11 | 0.7265 | -1.31 | 0.2362 | -1.89 | 0.0312 | -1.70 | 0.0289 |
| *HIF1A* | 3091 | NM_181054 | hypoxia-inducible factor 1, alpha subunit (basic helix-loop-helix transcription factor | -1.29 | 0.1543 | -1.65 | 0.0577 | 1.25 | 0.4870 | -1.32 | 0.2216 | -2.13 | 0.0100 | -1.70 | 0.0304 |
| *HK1* | 3098 | NM_000188 | hexokinase 1 | 1.01 | 0.9066 | -1.29 | 0.0685 | 1.17 | 0.2506 | -1.10 | 0.1720 | -1.27 | 0.2137 | -1.08 | 0.4831 |
| *HK2* | 3099 | BC021116 | hexokinase 2 | 1.10 | 0.5024 | -1.34 | 0.1323 | 1.09 | 0.6791 | -1.23 | 0.1325 | -1.22 | 0.3986 | -1.12 | 0.4991 |
| *HK2* | 3099 | NM_000189 | hexokinase 2 | -1.01 | 0.9574 | 1.08 | 0.7163 | -1.17 | 0.5231 | -1.09 | 0.6516 | 1.07 | 0.7385 | -1.10 | 0.6247 |
| *HK3* | 3101 | NM_002115 | hexokinase 3 (white cell) | -1.66 | 0.0932 | -1.30 | 0.3320 | 1.46 | 0.1870 | 1.12 | 0.5774 | -2.15 | 0.0615 | -1.48 | 0.1829 |
| *HMOX1* | 3162 | BC001491 | heme oxygenase (decycling) 1 | 1.62 | 0.2382 | 1.88 | 0.1080 | -1.25 | 0.5986 | 1.51 | 0.3582 | 3.04 | 0.0309 | 2.44 | 0.1387 |
| *HMOX1* | 3162 | NM_002133 | heme oxygenase (decycling) 1 | 1.72 | 0.1989 | 1.63 | 0.2241 | -1.17 | 0.7234 | 1.39 | 0.4458 | 2.80 | 0.0567 | 2.39 | 0.1471 |
| *ICAM1* | 3383 | NM_000201 | intercellular adhesion molecule 1 (CD54), human rhinovirus receptor | -1.01 | 0.9167 | 1.02 | 0.8572 | -1.02 | 0.8683 | -1.00 | 0.9936 | 1.01 | 0.9223 | -1.01 | 0.9214 |
| *IFNG* | 3458 | NM_000619 | interferon, gamma | -1.02 | 0.3928 | 1.10 | 0.0168 | -1.06 | 0.3785 | 1.05 | 0.3783 | 1.08 | 0.0752 | 1.02 | 0.6952 |
| *IFNGR1* | 3459 | NM_000416 | interferon gamma receptor 1 | -1.10 | 0.3157 | -1.29 | 0.0799 | 1.11 | 0.5250 | -1.16 | 0.2472 | -1.42 | 0.0157 | -1.27 | 0.0595 |
| *IFNGR2* | 3460 | NM_005534 | interferon gamma receptor 2 (interferon gamma transducer 1) | 1.07 | 0.1222 | -1.45 | 0.0001 | 1.21 | 0.0383 | -1.20 | 0.0011 | -1.35 | 0.0033 | -1.12 | 0.0637 |
| *IGF1* | 3479 | M11568 | insulin-like growth factor 1 (somatomedin C) | 1.25 | 0.4999 | 1.56 | 0.1435 | -1.51 | 0.0909 | 1.03 | 0.8790 | 1.94 | 0.0659 | 1.29 | 0.2402 |
| *IGF1* | 3479 | M29644 | insulin-like growth factor 1 (somatomedin C) | 1.51 | 0.2119 | 1.54 | 0.1651 | -1.21 | 0.4995 | 1.27 | 0.3537 | 2.33 | 0.0421 | 1.92 | 0.0517 |
| *IGF1* | 3479 | M37484 | insulin-like growth factor 1 (somatomedin C) | 1.34 | 0.1834 | 1.35 | 0.1641 | -1.21 | 0.3474 | 1.11 | 0.5906 | 1.81 | 0.0171 | 1.49 | 0.0728 |
| *IGF1* | 3479 | NM_000618 | insulin-like growth factor 1 (somatomedin C) | 1.08 | 0.2169 | 1.07 | 0.4663 | -1.07 | 0.4265 | -1.00 | 0.9949 | 1.15 | 0.1531 | 1.08 | 0.1115 |
| *IGF1R* | 3480 | NM_000875 | insulin-like growth factor 1 receptor | -1.03 | 0.8367 | -1.32 | 0.0737 | 1.06 | 0.6965 | -1.25 | 0.0186 | -1.36 | 0.1722 | -1.29 | 0.1081 |

| | | | | | | | | | | | | | |
|---|---|---|---|---|---|---|---|---|---|---|---|---|---|
| *IGF2* | 3481 | BC053318 | insulin-like growth factor 2 (somatomedin A) | -1.02 | 0.8596 | 1.57 | 0.0030 | -1.27 | 0.1310 | 1.23 | 0.1761 | 1.54 | 0.0041 | 1.21 | 0.2289 |
| *IGF2* | 3481 | NM_000612 | insulin-like growth factor 2 (somatomedin A) | -1.01 | 0.9064 | 1.34 | 0.0150 | -1.34 | 0.0109 | 1.00 | 0.9823 | 1.33 | 0.0165 | -1.01 | 0.9093 |
| *IGFBP1* | 3484 | NM_000596 | insulin-like growth factor binding protein 1 | -1.04 | 0.4088 | 1.07 | 0.1980 | 1.10 | 0.5202 | 1.17 | 0.2317 | 1.02 | 0.7294 | 1.13 | 0.4405 |
| *IGFBP1* | 3484 | NM_001013029 | insulin-like growth factor binding protein 1 | -1.05 | 0.4091 | 1.32 | 0.0039 | -1.18 | 0.0543 | 1.12 | 0.0808 | 1.26 | 0.0187 | 1.07 | 0.2956 |
| *IGFBP2* | 3485 | NM_000597 | insulin-like growth factor binding protein 2, 36kDa | 1.11 | 0.1761 | -1.24 | 0.0256 | 1.09 | 0.3720 | -1.14 | 0.0435 | -1.12 | 0.3392 | -1.03 | 0.7447 |
| *IGFBP3* | 3486 | BC000013 | insulin-like growth factor binding protein 3 | 1.28 | 0.1258 | 1.88 | 0.0423 | -1.65 | 0.0800 | 1.14 | 0.3686 | 2.41 | 0.0296 | 1.46 | 0.0192 |
| *IGFBP3* | 3486 | NM_000598 | insulin-like growth factor binding protein 3 | 1.08 | 0.5559 | 1.91 | 0.0235 | -1.54 | 0.1012 | 1.24 | 0.1307 | 2.07 | 0.0385 | 1.35 | 0.1044 |
| *IL1B* | 3553 | NM_000576 | interleukin 1, beta | -1.47 | 0.0340 | 1.71 | 0.0025 | -1.65 | 0.0156 | 1.04 | 0.8106 | 1.17 | 0.4234 | -1.41 | 0.1058 |
| *IL6* | 3569 | NM_000600 | interleukin 6 (interferon, beta 2) | 1.20 | 0.1997 | 1.11 | 0.4291 | -1.14 | 0.1285 | -1.03 | 0.7629 | 1.33 | 0.0310 | 1.16 | 0.0926 |
| *IL6R* | 3570 | BC089410 | interleukin 6 receptor | -1.04 | 0.7238 | -1.22 | 0.1467 | 1.07 | 0.6209 | -1.13 | 0.0707 | -1.27 | 0.2497 | -1.18 | 0.2225 |
| *IL6R* | 3570 | NM_000565 | interleukin 6 receptor | -1.16 | 0.4073 | -1.17 | 0.4418 | 1.03 | 0.9092 | -1.14 | 0.3791 | -1.36 | 0.2833 | -1.32 | 0.2040 |
| *IL8* | 3576 | NM_000584 | interleukin 8 | -1.31 | 0.6278 | 1.09 | 0.8906 | 1.02 | 0.9741 | 1.11 | 0.8617 | -1.21 | 0.7450 | -1.18 | 0.7699 |
| *IL12A* | 3592 | BC104982 | interleukin 12A (natural killer cell stimulatory factor 1, cytotoxic lymphocyte matura | -1.86 | 0.0295 | -1.15 | 0.0283 | 1.13 | 0.0082 | -1.02 | 0.6808 | -2.14 | 0.0342 | -1.89 | 0.0258 |
| *IL12A* | 3592 | NM_000882 | interleukin 12A (natural killer cell stimulatory factor 1, cytotoxic lymphocyte matura | -1.07 | 0.5346 | -1.14 | 0.1306 | 1.03 | 0.3199 | -1.10 | 0.1510 | -1.22 | 0.0667 | -1.18 | 0.0536 |
| *TNFRSF9* | 3604 | NM_001561 | tumor necrosis factor receptor superfamily, member 9 | -1.28 | 0.2675 | 1.04 | 0.8227 | -1.05 | 0.7749 | -1.01 | 0.9744 | -1.23 | 0.3704 | -1.29 | 0.2295 |
| *INS* | 3630 | BC005255 | insulin | -1.02 | 0.9071 | -1.26 | 0.1074 | 1.03 | 0.7980 | -1.22 | 0.1427 | -1.28 | 0.0600 | -1.24 | 0.1058 |
| *INS* | 3630 | NM_000207 | insulin | -1.05 | 0.5453 | -1.10 | 0.3398 | 1.03 | 0.8561 | -1.07 | 0.5732 | -1.16 | 0.2330 | -1.12 | 0.4092 |
| *INSR* | 3643 | NM_000208 | insulin receptor | 1.09 | 0.5218 | -1.33 | 0.0661 | 1.08 | 0.6259 | -1.22 | 0.0405 | -1.22 | 0.3734 | -1.12 | 0.4531 |
| *INSR* | 3643 | X02160 | insulin receptor | 1.04 | 0.8001 | 1.01 | 0.9287 | -1.02 | 0.9176 | -1.00 | 0.9714 | 1.05 | 0.7985 | 1.03 | 0.8525 |
| *ITGAM* | 3684 | BC096346 | integrin, alpha M (complement | -1.25 | 0.1456 | -1.06 | 0.6643 | -1.07 | 0.6501 | -1.13 | 0.2067 | -1.32 | 0.1788 | -1.41 | 0.0496 |

| | | | | | | | | | | | | | | | |
|---|---|---|---|---|---|---|---|---|---|---|---|---|---|---|---|
| | | | component 3 receptor 3 subunit) | | | | | | | | | | | | |
| *ITGAM* | 3684 | J03925 | integrin, alpha M (complement component 3 receptor 3 subunit) | -1.59 | 0.0734 | 1.04 | 0.8388 | -1.16 | 0.3964 | -1.12 | 0.5156 | -1.54 | 0.1297 | -1.79 | 0.0353 |
| *ITGAM* | 3684 | NM_000632 | integrin, alpha M (complement component 3 receptor 3 subunit) | -1.45 | 0.0646 | 1.06 | 0.6914 | -1.15 | 0.4029 | -1.08 | 0.6075 | -1.37 | 0.1455 | -1.57 | 0.0398 |
| *ITGB2* | 3689 | AK097864 | integrin, beta 2 (complement component 3 receptor 3 and 4 subunit) | 1.41 | 0.0886 | 1.04 | 0.8540 | 1.09 | 0.5800 | 1.12 | 0.4324 | 1.46 | 0.0560 | 1.58 | 0.0034 |
| *ITGB2* | 3689 | NM_000211 | integrin, beta 2 (complement component 3 receptor 3 and 4 subunit) | -1.16 | 0.0988 | -1.02 | 0.8606 | 1.01 | 0.9248 | -1.01 | 0.9324 | -1.18 | 0.1957 | -1.17 | 0.1335 |
| *KDR* | 3791 | NM_002253 | kinase insert domain receptor (a type III receptor tyrosine kinase) | 1.01 | 0.8927 | 1.22 | 0.0352 | -1.14 | 0.1401 | 1.07 | 0.3204 | 1.23 | 0.0356 | 1.08 | 0.2551 |
| *KRT14* | 3861 | NM_000526 | keratin 14 (epidermolysis bullosa simplex, Dowling-Meara, Koebner) | 1.13 | 0.7442 | 3.03 | 0.0235 | -1.88 | 0.1203 | 1.61 | 0.1294 | 3.41 | 0.0442 | 1.82 | 0.1454 |
| *KRT18* | 3875 | BC000180 | keratin 18 | 1.31 | 0.5193 | 2.62 | 0.0237 | -1.47 | 0.2670 | 1.78 | 0.0990 | 3.43 | 0.0290 | 2.33 | 0.0696 |
| *KRT18* | 3875 | BC004253 | keratin 18 | 1.33 | 0.5309 | 2.70 | 0.0162 | -1.46 | 0.2560 | 1.85 | 0.0937 | 3.60 | 0.0183 | 2.47 | 0.0612 |
| *KRT18* | 3875 | NM_000224 | keratin 18 | 1.20 | 0.6709 | 2.67 | 0.0137 | -1.41 | 0.2806 | 1.90 | 0.0631 | 3.20 | 0.0241 | 2.27 | 0.0684 |
| *KRT18* | 3875 | NM_199187 | keratin 18 | 1.32 | 0.5517 | 2.49 | 0.0176 | -1.47 | 0.2138 | 1.69 | 0.1136 | 3.28 | 0.0209 | 2.23 | 0.0739 |
| *KRT19* | 3880 | BC007628 | keratin 19 | 1.05 | 0.8101 | 2.35 | 0.0042 | -1.59 | 0.0766 | 1.47 | 0.0753 | 2.46 | 0.0129 | 1.55 | 0.1119 |
| *KRT19* | 3880 | NM_002276 | keratin 19 | 1.22 | 0.2805 | 1.61 | 0.0871 | -1.32 | 0.2714 | 1.22 | 0.2279 | 1.97 | 0.0601 | 1.49 | 0.0546 |
| *LAG3* | 3902 | BC052589 | lymphocyte-activation gene 3 | 1.05 | 0.7834 | 2.84 | 0.0015 | -2.10 | 0.0092 | 1.35 | 0.0879 | 2.97 | 0.0051 | 1.42 | 0.1103 |
| *LAG3* | 3902 | NM_002286 | lymphocyte-activation gene 3 | 1.18 | 0.6541 | 2.11 | 0.0919 | -1.51 | 0.3031 | 1.40 | 0.2970 | 2.49 | 0.1041 | 1.65 | 0.2191 |
| *LDHA* | 3939 | NM_005566 | lactate dehydrogenase A | -1.02 | 0.7968 | 1.17 | 0.1071 | -1.07 | 0.4783 | 1.09 | 0.2442 | 1.14 | 0.2606 | 1.07 | 0.4852 |
| *LEP* | 3952 | BC069323 | leptin (obesity homolog, mouse) | 1.62 | 0.0952 | 1.32 | 0.2848 | -1.17 | 0.5704 | 1.12 | 0.6722 | 2.13 | 0.0212 | 1.81 | 0.0925 |
| *LEP* | 3952 | D49487 | leptin (obesity homolog, mouse) | 1.43 | 0.1845 | 1.20 | 0.3952 | -1.25 | 0.1872 | -1.04 | 0.8195 | 1.72 | 0.0538 | 1.38 | 0.1067 |

| | | | | | | | | | | | | | | | |
|---|---|---|---|---|---|---|---|---|---|---|---|---|---|---|---|
| *LEP* | 3952 | NM_000230 | leptin (obesity homolog, mouse) | 1.37 | 0.1789 | 1.11 | 0.5912 | -1.01 | 0.9507 | 1.10 | 0.6399 | 1.52 | 0.0399 | 1.50 | 0.0742 |
| *LRP1* | 4035 | BC045107 | low density lipoprotein-related protein 1 (alpha-2-macroglobulin receptor) | 1.23 | 0.2679 | 1.46 | 0.0506 | -1.33 | 0.0957 | 1.10 | 0.5264 | 1.79 | 0.0133 | 1.35 | 0.0709 |
| *LRP1* | 4035 | BC052593 | low density lipoprotein-related protein 1 (alpha-2-macroglobulin receptor) | 1.04 | 0.7628 | 1.74 | 0.0016 | -1.37 | 0.0233 | 1.27 | 0.0615 | 1.81 | 0.0025 | 1.32 | 0.0393 |
| *LRP1* | 4035 | NM_002332 | low density lipoprotein-related protein 1 (alpha-2-macroglobulin receptor) | 1.25 | 0.4377 | 1.62 | 0.0773 | -1.25 | 0.4084 | 1.30 | 0.3325 | 2.02 | 0.0318 | 1.62 | 0.1362 |
| *LTBR* | 4055 | NM_002342 | lymphotoxin beta receptor (TNFR superfamily, member 3) | -1.08 | 0.3008 | -1.01 | 0.9004 | 1.07 | 0.5726 | 1.06 | 0.5372 | -1.09 | 0.4129 | -1.02 | 0.8598 |
| *MMP2* | 4313 | BC002576 | matrix metallopeptidase 2 (gelatinase A, 72kDa gelatinase, 72kDa type IV collagenase) | 1.13 | 0.1169 | -1.27 | 0.0176 | 1.09 | 0.4097 | -1.16 | 0.0086 | -1.12 | 0.4178 | -1.03 | 0.7783 |
| *MMP2* | 4313 | NM_004530 | matrix metallopeptidase 2 (gelatinase A, 72kDa gelatinase, 72kDa type IV collagenase) | 1.10 | 0.2164 | -1.17 | 0.1301 | 1.01 | 0.9527 | -1.16 | 0.0252 | -1.07 | 0.6152 | -1.06 | 0.4886 |
| *MMP9* | 4318 | NM_004994 | matrix metallopeptidase 9 (gelatinase B, 92kDa gelatinase, 92kDa type IV collagenase) | -2.09 | 0.0704 | 1.30 | 0.1666 | -1.23 | 0.4146 | 1.05 | 0.8391 | -1.61 | 0.2396 | -1.98 | 0.1029 |
| *MMP14* | 4323 | BC064803 | matrix metallopeptidase 14 (membrane-inserted) | 1.34 | 0.0681 | -1.23 | 0.1127 | 1.20 | 0.3280 | -1.02 | 0.8999 | 1.09 | 0.5666 | 1.31 | 0.2074 |
| *MMP14* | 4323 | NM_004995 | matrix metallopeptidase 14 (membrane-inserted) | 1.08 | 0.7391 | -1.06 | 0.7962 | 1.14 | 0.4647 | 1.08 | 0.6909 | 1.02 | 0.9088 | 1.17 | 0.4362 |

| | | | | | | | | | | | | | | | |
|---|---|---|---|---|---|---|---|---|---|---|---|---|---|---|---|
| *NFKB1* | 4790 | NM_003998 | nuclear factor of kappa light polypeptide gene enhancer in B-cells 1 (p105) | -1.05 | 0.3676 | -1.08 | 0.3803 | -1.06 | 0.5810 | -1.14 | 0.0188 | -1.14 | 0.2274 | -1.20 | 0.0068 |
| *NFKB1* | 4790 | BC051765 | nuclear factor of kappa light polypeptide gene enhancer in B-cells 1 (p105) | -1.04 | 0.5444 | -1.07 | 0.3971 | -1.03 | 0.7772 | -1.10 | 0.0865 | -1.11 | 0.2975 | -1.14 | 0.0662 |
| *NFKB1* | 4790 | M58603 | nuclear factor of kappa light polypeptide gene enhancer in B-cells 1 (p105) | -1.08 | 0.1449 | -1.02 | 0.7934 | -1.07 | 0.5229 | -1.09 | 0.1275 | -1.10 | 0.3457 | -1.18 | 0.0308 |
| *NOS2A* | 4843 | NM_000625 | nitric oxide synthase 2A (inducible, hepatocytes) | 1.09 | 0.2998 | 1.57 | 0.0136 | -1.29 | 0.1385 | 1.21 | 0.0530 | 1.71 | 0.0216 | 1.32 | 0.0419 |
| *NOS3* | 4846 | BC069465 | nitric oxide synthase 3 (endothelial cell) | -1.80 | 0.0258 | 1.15 | 0.4134 | -1.14 | 0.4898 | 1.01 | 0.9337 | -1.56 | 0.1076 | -1.77 | 0.0316 |
| *NOS3* | 4846 | NM_000603 | nitric oxide synthase 3 (endothelial cell) | -1.76 | 0.0138 | 1.37 | 0.1463 | -1.36 | 0.1585 | 1.00 | 0.9845 | -1.29 | 0.2945 | -1.75 | 0.0156 |
| *NPPA* | 4878 | BC005893 | natriuretic peptide precursor A | -1.14 | 0.4085 | 1.49 | 0.0108 | -1.29 | 0.0651 | 1.15 | 0.2211 | 1.31 | 0.1247 | 1.02 | 0.9104 |
| *NPPA* | 4878 | NM_006172 | natriuretic peptide precursor A | -1.13 | 0.4634 | 1.36 | 0.0591 | -1.20 | 0.2455 | 1.13 | 0.3494 | 1.21 | 0.3241 | 1.01 | 0.9731 |
| *SERPINE1* | 5054 | BC010860 | serpin peptidase inhibitor, clade E (nexin, plasminogen activator inhibitor type 1), m | 1.24 | 0.5610 | 1.73 | 0.2666 | -1.48 | 0.4268 | 1.17 | 0.7015 | 2.14 | 0.1973 | 1.44 | 0.4141 |
| *SERPINE1* | 5054 | NM_000602 | serpin peptidase inhibitor, clade E (nexin, plasminogen activator inhibitor type 1), m | 1.11 | 0.6285 | 1.95 | 0.0335 | -1.36 | 0.3206 | 1.44 | 0.1827 | 2.16 | 0.0419 | 1.59 | 0.1536 |
| *PDGFB* | 5155 | BC029822 | platelet-derived growth factor beta polypeptide (simian sarcoma viral (v-sis) oncogene | 1.11 | 0.6341 | 1.06 | 0.8083 | -1.01 | 0.9636 | 1.05 | 0.8335 | 1.18 | 0.5063 | 1.16 | 0.5224 |
| *PDGFB* | 5155 | BC077725 | platelet-derived growth factor beta polypeptide (simian | 1.26 | 0.4449 | 1.78 | 0.0344 | -1.31 | 0.2749 | 1.36 | 0.2015 | 2.24 | 0.0263 | 1.71 | 0.0924 |

| | | | | | | | | | | | | | | | |
|---|---|---|---|---|---|---|---|---|---|---|---|---|---|---|---|
| | | | sarcoma viral (v-sis) oncogene | | | | | | | | | | | | |
| *PDGFB* | 5155 | NM_002608 | platelet-derived growth factor beta polypeptide (simian sarcoma viral (v-sis) oncogene | -1.01 | 0.9342 | 1.27 | 0.1393 | 1.04 | 0.8263 | 1.33 | 0.1338 | 1.25 | 0.2179 | 1.31 | 0.2096 |
| *PDGFB* | 5155 | X83705 | platelet-derived growth factor beta polypeptide (simian sarcoma viral (v-sis) oncogene | -1.12 | 0.4831 | 1.31 | 0.0760 | -1.15 | 0.3281 | 1.14 | 0.3410 | 1.17 | 0.3626 | 1.02 | 0.9169 |
| *PDHA1* | 5160 | CR614489 | pyruvate dehydrogenase (lipoamide) alpha 1 | -1.04 | 0.1698 | -1.10 | 0.0229 | 1.04 | 0.2885 | -1.05 | 0.0171 | -1.14 | 0.0200 | -1.10 | 0.0115 |
| *PDHA1* | 5160 | NM_000284 | pyruvate dehydrogenase (lipoamide) alpha 1 | -1.13 | 0.0021 | -1.09 | 0.1604 | -1.01 | 0.9155 | -1.10 | 0.0147 | -1.24 | 0.0118 | -1.25 | 0.0003 |
| *PDHA2* | 5161 | NM_005390 | pyruvate dehydrogenase (lipoamide) alpha 2 | 1.30 | 0.2600 | 1.36 | 0.2876 | -1.15 | 0.5971 | 1.18 | 0.3115 | 1.77 | 0.1535 | 1.53 | 0.0679 |
| *PDHB* | 5162 | NM_000925 | pyruvate dehydrogenase (lipoamide) beta | -1.30 | 0.0045 | -1.07 | 0.6919 | -1.02 | 0.9079 | -1.09 | 0.4256 | -1.39 | 0.0490 | -1.41 | 0.0015 |
| *PDK1* | 5163 | BC039158 | pyruvate dehydrogenase kinase, isozyme 1 | 1.18 | 0.0169 | 1.10 | 0.1436 | -1.04 | 0.5403 | 1.06 | 0.2746 | 1.30 | 0.0140 | 1.25 | 0.0081 |
| *PDK1* | 5163 | DQ234350 | pyruvate dehydrogenase kinase, isozyme 1 | 1.20 | 0.0001 | -1.13 | 0.0061 | 1.16 | 0.0177 | 1.02 | 0.6090 | 1.06 | 0.1529 | 1.23 | 0.0016 |
| *PDK1* | 5163 | NM_002610 | pyruvate dehydrogenase kinase, isozyme 1 | 1.17 | 0.0115 | 1.05 | 0.2752 | -1.01 | 0.8538 | 1.04 | 0.3332 | 1.23 | 0.0044 | 1.22 | 0.0030 |
| *PFKFB3* | 5209 | AF056320 | 6-phosphofructo-2-kinase/fructose-2,6-biphosphatase 3 | -1.03 | 0.7359 | 1.12 | 0.1721 | -1.13 | 0.1810 | -1.01 | 0.9411 | 1.09 | 0.0511 | -1.03 | 0.6957 |
| *PFKFB3* | 5209 | AK131307 | 6-phosphofructo-2-kinase/fructose-2,6-biphosphatase 3 | -1.01 | 0.8819 | 1.09 | 0.2787 | -1.10 | 0.2390 | -1.02 | 0.8695 | 1.08 | 0.0657 | -1.03 | 0.7445 |
| *PFKFB3* | 5209 | NM_004566 | 6-phosphofructo-2-kinase/fructose-2,6-biphosphatase 3 | -1.11 | 0.3814 | -1.02 | 0.8519 | -1.11 | 0.5644 | -1.14 | 0.4574 | -1.13 | 0.1907 | -1.26 | 0.1726 |

| | | | | | | | | | | | | | | | |
|---|---|---|---|---|---|---|---|---|---|---|---|---|---|---|---|
| *PFKL* | 5211 | NM_001002021 | phosphofructokinase, liver | -1.17 | 0.2103 | -1.45 | 0.0209 | 1.19 | 0.2768 | -1.23 | 0.0197 | -1.70 | 0.0202 | -1.43 | 0.0139 |
| *PFKL* | 5211 | BX537446 | phosphofructokinase, liver | -1.13 | 0.2922 | -1.27 | 0.0986 | 1.15 | 0.3973 | -1.11 | 0.1995 | -1.44 | 0.0887 | -1.25 | 0.1075 |
| *PGK1* | 5230 | BC023234 | phosphoglycerate kinase 1 | -1.09 | 0.2597 | -1.03 | 0.6972 | -1.00 | 0.9725 | -1.03 | 0.6277 | -1.11 | 0.2756 | -1.12 | 0.2009 |
| *PGK1* | 5230 | BC103752 | phosphoglycerate kinase 1 | -1.01 | 0.9430 | -1.06 | 0.3568 | 1.06 | 0.4575 | -1.01 | 0.9204 | -1.07 | 0.4757 | -1.01 | 0.8888 |
| *PGK1* | 5230 | NM_000291 | phosphoglycerate kinase 1 | -1.01 | 0.8469 | 1.04 | 0.6356 | -1.05 | 0.5840 | -1.01 | 0.9218 | 1.02 | 0.7869 | -1.02 | 0.7801 |
| *ABCB1* | 5243 | AF016535 | ATP-binding cassette, sub-family B (MDR/TAP), member 1 | -1.03 | 0.8293 | 1.43 | 0.0671 | -1.21 | 0.2876 | 1.18 | 0.1936 | 1.39 | 0.1387 | 1.14 | 0.3428 |
| *ABCB1* | 5243 | NM_000927 | ATP-binding cassette, sub-family B (MDR/TAP), member 1 | 1.14 | 0.2093 | 1.47 | 0.1314 | -1.39 | 0.2177 | 1.06 | 0.6930 | 1.68 | 0.1071 | 1.21 | 0.2759 |
| *PIK3CA* | 5290 | NM_006218 | phosphoinositide-3-kinase, catalytic, alpha polypeptide | -1.13 | 0.5602 | -1.95 | 0.0401 | 1.29 | 0.4552 | -1.52 | 0.0839 | -2.20 | 0.0187 | -1.71 | 0.0294 |
| *PIK3CB* | 5291 | NM_006219 | phosphoinositide-3-kinase, catalytic, beta polypeptide | -1.32 | 0.0711 | -1.77 | 0.0337 | 1.14 | 0.5993 | -1.55 | 0.0304 | -2.34 | 0.0011 | -2.05 | 0.0002 |
| *PIK3CB* | 5291 | CR749357 | phosphoinositide-3-kinase, catalytic, beta polypeptide | -1.26 | 0.3051 | -1.33 | 0.2100 | -1.05 | 0.7482 | -1.39 | 0.0894 | -1.67 | 0.0508 | -1.75 | 0.0143 |
| *PIK3CD* | 5293 | U57843 | phosphoinositide-3-kinase, catalytic, delta polypeptide | -1.18 | 0.1240 | -1.20 | 0.0561 | 1.02 | 0.8076 | -1.18 | 0.0214 | -1.42 | 0.0220 | -1.39 | 0.0070 |
| *PIK3CD* | 5293 | NM_005026 | phosphoinositide-3-kinase, catalytic, delta polypeptide | -1.23 | 0.0190 | -1.11 | 0.3709 | -1.03 | 0.8302 | -1.15 | 0.1724 | -1.37 | 0.0282 | -1.41 | 0.0055 |
| *PIK3R1* | 5295 | BC030815 | phosphoinositide-3-kinase, regulatory subunit 1 (p85 alpha) | 1.04 | 0.7875 | -1.37 | 0.0832 | 1.08 | 0.7251 | -1.27 | 0.0321 | -1.32 | 0.2924 | -1.22 | 0.2671 |
| *PIK3R1* | 5295 | BC094795 | phosphoinositide-3-kinase, regulatory subunit 1 (p85 alpha) | -1.19 | 0.5348 | -2.43 | 0.0247 | 1.30 | 0.4025 | -1.86 | 0.0270 | -2.88 | 0.0223 | -2.22 | 0.0172 |
| *PIK3R1* | 5295 | NM_181504 | phosphoinositide-3-kinase, regulatory subunit 1 (p85 alpha) | -1.03 | 0.7601 | -1.29 | 0.0955 | -1.02 | 0.8974 | -1.33 | 0.0144 | -1.34 | 0.1479 | -1.37 | 0.0411 |

| | | | | | | | | | | | | | | | |
|---|---|---|---|---|---|---|---|---|---|---|---|---|---|---|---|
| *PIK3R2* | 5296 | NM_005027 | phosphoinositide-3-kinase, regulatory subunit 2 (p85 beta) | 1.04 | 0.7462 | -1.17 | 0.2886 | 1.08 | 0.6384 | -1.08 | 0.4875 | -1.13 | 0.5222 | -1.04 | 0.7807 |
| *PKM2* | 5315 | NM_002654 | pyruvate kinase, muscle | -1.11 | 0.2198 | -1.01 | 0.8437 | 1.07 | 0.3655 | 1.05 | 0.3084 | -1.12 | 0.2881 | -1.05 | 0.5508 |
| *PLAUR* | 5329 | NM_001005376 | plasminogen activator, urokinase receptor | 1.32 | 0.0246 | 1.08 | 0.5642 | -1.10 | 0.5761 | -1.01 | 0.9172 | 1.43 | 0.0199 | 1.30 | 0.1120 |
| *PLAUR* | 5329 | NM_001005377 | plasminogen activator, urokinase receptor | 1.27 | 0.0708 | 1.10 | 0.5345 | -1.05 | 0.7819 | 1.04 | 0.7891 | 1.39 | 0.0457 | 1.32 | 0.1263 |
| *PLAUR* | 5329 | NM_002659 | plasminogen activator, urokinase receptor | 1.33 | 0.0197 | 1.11 | 0.4107 | -1.10 | 0.5448 | 1.01 | 0.9386 | 1.48 | 0.0104 | 1.34 | 0.0754 |
| *PLCG1* | 5335 | NM_002660 | phospholipase C, gamma 1 | 1.07 | 0.7177 | -1.34 | 0.1136 | 1.22 | 0.3036 | -1.10 | 0.5369 | -1.25 | 0.3475 | -1.03 | 0.8920 |
| *PLCG2* | 5336 | BC007565 | phospholipase C, gamma 2 (phosphatidylinositol-specific) | -1.35 | 0.0012 | -1.15 | 0.1064 | 1.07 | 0.4657 | -1.08 | 0.2908 | -1.55 | 0.0007 | -1.45 | 0.0004 |
| *PLCG2* | 5336 | NM_002661 | phospholipase C, gamma 2 (phosphatidylinositol-specific) | -1.38 | 0.0003 | -1.21 | 0.0428 | 1.10 | 0.2882 | -1.10 | 0.1664 | -1.67 | 0.0002 | -1.51 | 0.0000 |
| *PRKCA* | 5578 | BC109273 | protein kinase C, alpha | -1.06 | 0.2834 | 1.37 | 0.0636 | -1.32 | 0.1118 | 1.04 | 0.6029 | 1.28 | 0.1889 | -1.03 | 0.7641 |
| *PRKCA* | 5578 | NM_002737 | protein kinase C, alpha | 1.04 | 0.6970 | 1.30 | 0.0672 | -1.27 | 0.1145 | 1.02 | 0.8016 | 1.35 | 0.0895 | 1.06 | 0.6010 |
| *PRKCB1* | 5579 | NM_212535 | protein kinase C, beta 1 | -1.27 | 0.1790 | -1.10 | 0.4862 | -1.17 | 0.2398 | -1.29 | 0.0167 | -1.40 | 0.1377 | -1.63 | 0.0131 |
| *PRKCB1* | 5579 | BC036472 | protein kinase C, beta 1 | -1.25 | 0.0176 | -1.09 | 0.4101 | -1.06 | 0.6182 | -1.15 | 0.0961 | -1.36 | 0.0191 | -1.44 | 0.0014 |
| *PRKCB1* | 5579 | NM_002738 | protein kinase C, beta 1 | -1.13 | 0.4720 | -1.44 | 0.1200 | 1.14 | 0.4164 | -1.26 | 0.2198 | -1.63 | 0.0037 | -1.43 | 0.0097 |
| *PRKCG* | 5582 | BC047876 | protein kinase C, gamma | 1.24 | 0.3085 | 2.85 | 0.0023 | -1.80 | 0.0354 | 1.58 | 0.0372 | 3.55 | 0.0047 | 1.97 | 0.0237 |
| *PRKCG* | 5582 | NM_002739 | protein kinase C, gamma | 1.29 | 0.3230 | 2.61 | 0.0113 | -1.71 | 0.0882 | 1.52 | 0.0523 | 3.37 | 0.0181 | 1.97 | 0.0361 |
| *MAPK1* | 5594 | NM_138957 | mitogen-activated protein kinase 1 | -1.29 | 0.1468 | -1.96 | 0.0213 | 1.33 | 0.2324 | -1.48 | 0.0420 | -2.54 | 0.0018 | -1.91 | 0.0008 |
| *MAPK1* | 5594 | Z11695 | mitogen-activated protein kinase 1 | 1.01 | 0.8892 | -1.51 | 0.0136 | 1.21 | 0.2249 | -1.25 | 0.0339 | -1.49 | 0.0299 | -1.23 | 0.0609 |
| *MAPK1* | 5594 | BC099905 | mitogen-activated protein kinase 1 | -1.11 | 0.3164 | 1.04 | 0.7443 | -1.04 | 0.7803 | 1.00 | 0.9892 | -1.07 | 0.6277 | -1.11 | 0.3969 |
| *MAPK1* | 5594 | NM_002745 | mitogen-activated protein kinase 1 | -1.18 | 0.5167 | -1.36 | 0.2472 | -1.11 | 0.5086 | -1.52 | 0.1136 | -1.60 | 0.0441 | -1.79 | 0.0167 |

| | | | | | | | | | | | | | | | |
|---|---|---|---|---|---|---|---|---|---|---|---|---|---|---|---|
| *MAPK3* | 5595 | NM_002746 | mitogen-activated protein kinase 3 | 1.22 | 0.0470 | -1.30 | 0.0378 | 1.08 | 0.5471 | -1.21 | 0.0221 | -1.06 | 0.6788 | 1.01 | 0.9022 |
| *MAPK3* | 5595 | AY033607 | mitogen-activated protein kinase 3 | 1.24 | 0.3419 | -1.50 | 0.1438 | 1.27 | 0.2736 | -1.18 | 0.3465 | -1.21 | 0.5064 | 1.05 | 0.7644 |
| *MAPK3* | 5595 | NM_001040056 | mitogen-activated protein kinase 3 | 1.14 | 0.1930 | -1.04 | 0.6958 | -1.01 | 0.9635 | -1.05 | 0.6717 | 1.09 | 0.5807 | 1.08 | 0.6207 |
| *MAP2K1* | 5604 | NM_002755 | mitogen-activated protein kinase kinase 1 | 1.07 | 0.4820 | -1.18 | 0.1248 | 1.12 | 0.3382 | -1.06 | 0.5563 | -1.10 | 0.3942 | 1.01 | 0.9117 |
| *MAP2K2* | 5605 | NM_030662 | mitogen-activated protein kinase kinase 2 | -1.04 | 0.6285 | -1.15 | 0.1033 | 1.05 | 0.6084 | -1.10 | 0.1944 | -1.20 | 0.1326 | -1.14 | 0.1795 |
| *RELA* | 5970 | BC011603 | v-rel reticuloendotheliosis viral oncogene homolog A, nuclear factor of kappa light po | -1.23 | 0.2221 | -1.34 | 0.2404 | 1.19 | 0.5996 | -1.13 | 0.5025 | -1.65 | 0.1167 | -1.39 | 0.1654 |
| *RELA* | 5970 | BC014095 | v-rel reticuloendotheliosis viral oncogene homolog A, nuclear factor of kappa light po | -1.14 | 0.2904 | -1.01 | 0.9761 | 1.05 | 0.8354 | 1.04 | 0.7886 | -1.15 | 0.5190 | -1.10 | 0.6299 |
| *RELA* | 5970 | BC110830 | v-rel reticuloendotheliosis viral oncogene homolog A, nuclear factor of kappa light po | -1.19 | 0.2177 | 1.12 | 0.6486 | -1.06 | 0.8461 | 1.06 | 0.7625 | -1.06 | 0.8095 | -1.13 | 0.5547 |
| *RELA* | 5970 | NM_021975 | v-rel reticuloendotheliosis viral oncogene homolog A, nuclear factor of kappa light po | -1.20 | 0.2255 | -1.37 | 0.1749 | 1.21 | 0.5245 | -1.13 | 0.4920 | -1.64 | 0.0956 | -1.35 | 0.1747 |
| *RORC* | 6097 | BC031554 | RAR-related orphan receptor C | 1.10 | 0.4074 | 1.35 | 0.0366 | -1.23 | 0.1716 | 1.10 | 0.4291 | 1.48 | 0.0250 | 1.20 | 0.1896 |
| *RORC* | 6097 | BC110571 | RAR-related orphan receptor C | 1.19 | 0.2082 | -1.02 | 0.9056 | -1.19 | 0.1634 | -1.21 | 0.1846 | 1.17 | 0.1075 | -1.01 | 0.8923 |
| *RORC* | 6097 | NM_001001523 | RAR-related orphan receptor C | -1.05 | 0.6217 | 1.66 | 0.0215 | -1.43 | 0.0882 | 1.16 | 0.1996 | 1.58 | 0.0651 | 1.10 | 0.4526 |
| *RPS6* | 6194 | NM_001010 | ribosomal protein S6 | 1.10 | 0.4975 | 1.04 | 0.6804 | -1.05 | 0.4940 | -1.01 | 0.8708 | 1.14 | 0.3867 | 1.08 | 0.4978 |
| *RPS6KB1* | 6198 | BC053365 | ribosomal protein S6 kinase, 70kDa, polypeptide 1 | -1.04 | 0.6098 | -1.33 | 0.0132 | 1.13 | 0.3161 | -1.18 | 0.0474 | -1.38 | 0.0200 | -1.22 | 0.0442 |

| | | | | | | | | | | | | | | | |
|---|---|---|---|---|---|---|---|---|---|---|---|---|---|---|---|
| *RPS6KB1* | 6198 | BC036033 | ribosomal protein S6 kinase, 70kDa, polypeptide 1 | -1.14 | 0.2782 | -1.27 | 0.1491 | 1.08 | 0.7264 | -1.18 | 0.2898 | -1.45 | 0.0485 | -1.34 | 0.0878 |
| *RPS6KB1* | 6198 | NM_003161 | ribosomal protein S6 kinase, 70kDa, polypeptide 1 | 1.07 | 0.4739 | -1.18 | 0.1045 | 1.06 | 0.4982 | -1.11 | 0.1884 | -1.10 | 0.4474 | -1.03 | 0.7457 |
| *RPS6KB2* | 6199 | BC000094 | ribosomal protein S6 kinase, 70kDa, polypeptide 2 | -1.03 | 0.7897 | -1.40 | 0.0303 | 1.25 | 0.2340 | -1.12 | 0.2991 | -1.44 | 0.0844 | -1.15 | 0.3472 |
| *RPS6KB2* | 6199 | BC006106 | ribosomal protein S6 kinase, 70kDa, polypeptide 2 | -1.02 | 0.8793 | -1.41 | 0.0316 | 1.29 | 0.1553 | -1.10 | 0.3956 | -1.44 | 0.0888 | -1.12 | 0.4530 |
| *RPS6KB2* | 6199 | NM_001007071 | ribosomal protein S6 kinase, 70kDa, polypeptide 2 | -1.04 | 0.6708 | -1.31 | 0.0356 | 1.20 | 0.2652 | -1.09 | 0.4028 | -1.36 | 0.0880 | -1.14 | 0.3680 |
| *CCL2* | 6347 | NM_002982 | chemokine (C-C motif) ligand 2 | 1.03 | 0.9288 | -1.04 | 0.8530 | -1.04 | 0.8177 | -1.08 | 0.5854 | -1.01 | 0.9757 | -1.05 | 0.8635 |
| *CXCL6* | 6372 | NM_002993 | chemokine (C-X-C motif) ligand 6 (granulocyte chemotactic protein 2) | -1.19 | 0.1518 | -1.03 | 0.4858 | 1.02 | 0.7451 | -1.01 | 0.7947 | -1.22 | 0.1650 | -1.20 | 0.1409 |
| *SELL* | 6402 | BC020758 | selectin L (lymphocyte adhesion molecule 1) | -1.40 | 0.0408 | -1.42 | 0.0376 | -1.06 | 0.6168 | -1.51 | 0.0097 | -1.99 | 0.0015 | -2.10 | 0.0002 |
| *SELL* | 6402 | NM_000655 | selectin L (lymphocyte adhesion molecule 1) | -1.41 | 0.0424 | -1.25 | 0.1981 | -1.18 | 0.3446 | -1.48 | 0.0051 | -1.77 | 0.0190 | -2.08 | 0.0007 |
| *SELL* | 6402 | AJ246000 | selectin L (lymphocyte adhesion molecule 1) | -1.40 | 0.1010 | -1.53 | 0.0708 | 1.07 | 0.6534 | -1.43 | 0.0708 | -2.15 | 0.0040 | -2.00 | 0.0018 |
| *SLC2A1* | 6513 | NM_006516 | solute carrier family 2 (facilitated glucose transporter), member 1 | -1.25 | 0.4182 | 1.06 | 0.8667 | 1.06 | 0.8764 | 1.12 | 0.7007 | -1.18 | 0.6359 | -1.12 | 0.7185 |
| *SLC2A3* | 6515 | BC039196 | solute carrier family 2 (facilitated glucose transporter), member 3 | 1.52 | 0.0925 | 1.16 | 0.5984 | -1.16 | 0.6221 | -1.00 | 0.9878 | 1.75 | 0.0988 | 1.51 | 0.1622 |
| *SLC2A3* | 6515 | NM_006931 | solute carrier family 2 (facilitated glucose transporter), member 3 | 1.51 | 0.0382 | 1.20 | 0.3444 | -1.15 | 0.4989 | 1.04 | 0.8142 | 1.81 | 0.0151 | 1.57 | 0.0471 |
| *SLC11A1* | 6556 | BC041787 | solute carrier family 11 (proton-coupled divalent metal ion transporters), member 1 | 1.17 | 0.3635 | 1.45 | 0.0261 | -1.21 | 0.2053 | 1.19 | 0.1381 | 1.69 | 0.0297 | 1.39 | 0.0715 |

| | | | | | | | | | | | | | | | |
|---|---|---|---|---|---|---|---|---|---|---|---|---|---|---|---|
| *SLC11A1* | 6556 | NM_000578 | solute carrier family 11 (proton-coupled divalent metal ion transporters), member 1 | -1.02 | 0.9322 | 1.47 | 0.0550 | -1.12 | 0.5454 | 1.31 | 0.1981 | 1.44 | 0.0614 | 1.29 | 0.2440 |
| *SLC11A1* | 6556 | NM_001032220 | solute carrier family 11 (proton-coupled divalent metal ion transporters), member 1 | 1.08 | 0.6329 | 1.68 | 0.0181 | -1.43 | 0.1090 | 1.17 | 0.4166 | 1.81 | 0.0184 | 1.26 | 0.2834 |
| *STAT3* | 6774 | BC000627 | signal transducer and activator of transcription 3 (acute-phase response factor) | 1.05 | 0.5343 | -1.23 | 0.0222 | 1.08 | 0.3917 | -1.14 | 0.0144 | -1.17 | 0.1898 | -1.09 | 0.2838 |
| *STAT3* | 6774 | BC014482 | signal transducer and activator of transcription 3 (acute-phase response factor) | 1.04 | 0.6164 | -1.23 | 0.0410 | 1.07 | 0.4954 | -1.15 | 0.0048 | -1.18 | 0.2510 | -1.10 | 0.2725 |
| *STAT3* | 6774 | NM_003150 | signal transducer and activator of transcription 3 (acute-phase response factor) | -1.23 | 0.0619 | -1.10 | 0.5460 | 1.04 | 0.8836 | -1.06 | 0.7333 | -1.36 | 0.0751 | -1.31 | 0.1538 |
| *STAT3* | 6774 | NM_213662 | signal transducer and activator of transcription 3 (acute-phase response factor) | -1.17 | 0.1186 | -1.08 | 0.6467 | 1.03 | 0.9016 | -1.05 | 0.7717 | -1.26 | 0.1638 | -1.23 | 0.2210 |
| *STAT4* | 6775 | NM_003151 | signal transducer and activator of transcription 4 | 1.25 | 0.2699 | 1.31 | 0.1903 | -1.14 | 0.5169 | 1.14 | 0.4755 | 1.63 | 0.0422 | 1.43 | 0.1074 |
| *TCEB1* | 6921 | BC093065 | transcription elongation factor B (SIII), polypeptide 1 (15kDa, elongin C) | 1.17 | 0.2684 | 1.23 | 0.1793 | -1.25 | 0.1818 | -1.01 | 0.9415 | 1.44 | 0.0801 | 1.16 | 0.3297 |
| *TCEB1* | 6921 | BC100028 | transcription elongation factor B (SIII), polypeptide 1 (15kDa, elongin C) | 1.18 | 0.2361 | 1.22 | 0.2191 | -1.23 | 0.2193 | -1.01 | 0.9274 | 1.44 | 0.0991 | 1.17 | 0.3062 |
| *TCEB1* | 6921 | BC100283 | transcription elongation factor B (SIII), polypeptide 1 (15kDa, elongin C) | 1.19 | 0.2944 | 1.20 | 0.2226 | -1.19 | 0.2421 | 1.00 | 0.9733 | 1.43 | 0.1050 | 1.19 | 0.2964 |

| | | | | | | | | | | | | | | | |
|---|---|---|---|---|---|---|---|---|---|---|---|---|---|---|---|
| *TCEB1* | 6921 | NM_005648 | transcription elongation factor B (SIII), polypeptide 1 (15kDa, elongin C) | 1.13 | 0.3874 | 1.22 | 0.2225 | -1.24 | 0.2002 | -1.02 | 0.8867 | 1.38 | 0.1359 | 1.11 | 0.4657 |
| *TCEB2* | 6923 | BC013306 | transcription elongation factor B (SIII), polypeptide 2 (18kDa, elongin B) | 1.29 | 0.0072 | 1.02 | 0.7270 | 1.05 | 0.5546 | 1.08 | 0.3929 | 1.32 | 0.0013 | 1.39 | 0.0080 |
| *TCEB2* | 6923 | BC065000 | transcription elongation factor B (SIII), polypeptide 2 (18kDa, elongin B) | 1.18 | 0.0071 | 1.06 | 0.0577 | 1.07 | 0.3479 | 1.14 | 0.0493 | 1.26 | 0.0026 | 1.34 | 0.0042 |
| *TCEB2* | 6923 | NM_007108 | transcription elongation factor B (SIII), polypeptide 2 (18kDa, elongin B) | 1.26 | 0.0202 | 1.01 | 0.9040 | 1.05 | 0.5424 | 1.07 | 0.3976 | 1.27 | 0.0396 | 1.34 | 0.0074 |
| *TCEB2* | 6923 | NM_207013 | transcription elongation factor B (SIII), polypeptide 2 (18kDa, elongin B) | 1.24 | 0.0053 | 1.04 | 0.4812 | 1.02 | 0.7156 | 1.07 | 0.3054 | 1.29 | 0.0012 | 1.32 | 0.0015 |
| *TCF7* | 6932 | BC048769 | transcription factor 7 (T-cell specific, HMG-box) | -1.60 | 0.0425 | -1.03 | 0.7835 | -1.15 | 0.2247 | -1.18 | 0.1969 | -1.65 | 0.0582 | -1.89 | 0.0153 |
| *TCF7* | 6932 | NM_003202 | transcription factor 7 (T-cell specific, HMG-box) | -1.78 | 0.0402 | -1.01 | 0.9373 | -1.08 | 0.4520 | -1.09 | 0.3726 | -1.80 | 0.0731 | -1.94 | 0.0242 |
| *TCF7* | 6932 | NM_201633 | transcription factor 7 (T-cell specific, HMG-box) | -1.38 | 0.0280 | 1.13 | 0.3383 | -1.08 | 0.5257 | 1.05 | 0.6426 | -1.23 | 0.1865 | -1.32 | 0.0425 |
| *TEK* | 7010 | NM_000459 | TEK tyrosine kinase, endothelial (venous malformations, multiple cutaneous and mucosal | -1.00 | 0.8218 | 1.03 | 0.1282 | -1.02 | 0.3696 | 1.01 | 0.4431 | 1.03 | 0.2337 | 1.01 | 0.6240 |
| *TF* | 7018 | AK126941 | transferrin | -1.09 | 0.3212 | 1.34 | 0.0344 | -1.31 | 0.0917 | 1.03 | 0.7943 | 1.23 | 0.1675 | -1.06 | 0.6411 |
| *TF* | 7018 | NM_001063 | transferrin | -1.29 | 0.0081 | 1.31 | 0.0014 | -1.13 | 0.1872 | 1.16 | 0.0961 | 1.02 | 0.8413 | -1.11 | 0.3051 |
| *TFF3* | 7033 | BC017859 | trefoil factor 3 (intestinal) | 1.10 | 0.5641 | 1.63 | 0.0197 | -1.58 | 0.0178 | 1.03 | 0.8037 | 1.79 | 0.0226 | 1.13 | 0.3606 |
| *TFF3* | 7033 | NM_003226 | trefoil factor 3 (intestinal) | 1.38 | 0.3933 | 1.94 | 0.0762 | -1.57 | 0.1942 | 1.24 | 0.4414 | 2.68 | 0.0574 | 1.71 | 0.1676 |
| *TFRC* | 7037 | BC001188 | transferrin receptor (p90, CD71) | -1.01 | 0.9167 | -1.07 | 0.6556 | 1.09 | 0.5953 | 1.02 | 0.8389 | -1.08 | 0.6754 | 1.01 | 0.9447 |

| | | | | | | | | | | | | | | | |
|---|---|---|---|---|---|---|---|---|---|---|---|---|---|---|---|
| *TFRC* | 7037 | NM_003234 | transferrin receptor (p90, CD71) | -1.01 | 0.9313 | -1.01 | 0.9667 | 1.06 | 0.6978 | 1.05 | 0.6039 | -1.02 | 0.9267 | 1.04 | 0.7472 |
| *TGFA* | 7039 | AF149096 | transforming growth factor, alpha | -1.11 | 0.1982 | 1.13 | 0.2575 | 1.03 | 0.8562 | 1.16 | 0.2114 | 1.02 | 0.8515 | 1.05 | 0.7082 |
| *TGFA* | 7039 | AF149098 | transforming growth factor, alpha | 1.07 | 0.6478 | -1.05 | 0.7819 | 1.06 | 0.6693 | 1.01 | 0.9272 | 1.03 | 0.8011 | 1.09 | 0.5086 |
| *TGFA* | 7039 | BC005308 | transforming growth factor, alpha | 1.44 | 0.1466 | 1.04 | 0.8798 | -1.04 | 0.8943 | 1.01 | 0.9779 | 1.50 | 0.1615 | 1.45 | 0.1164 |
| *TGFA* | 7039 | NM_003236 | transforming growth factor, alpha | -1.48 | 0.0539 | -1.19 | 0.0387 | 1.16 | 0.1755 | -1.03 | 0.7820 | -1.76 | 0.0299 | -1.52 | 0.0504 |
| *TGFB1* | 7040 | BC000125 | transforming growth factor, beta 1 (Camurati-Engelmann disease) | 1.15 | 0.0681 | -1.11 | 0.1247 | 1.01 | 0.8995 | -1.10 | 0.0984 | 1.03 | 0.7474 | 1.04 | 0.6164 |
| *TGFB1* | 7040 | NM_000660 | transforming growth factor, beta 1 (Camurati-Engelmann disease) | 1.14 | 0.1458 | -1.06 | 0.4397 | -1.00 | 0.9907 | -1.06 | 0.3477 | 1.08 | 0.5522 | 1.08 | 0.4853 |
| *TGFB3* | 7043 | BC018503 | transforming growth factor, beta 3 | -1.05 | 0.7632 | -1.30 | 0.1560 | 1.19 | 0.3473 | -1.10 | 0.5393 | -1.36 | 0.1019 | -1.15 | 0.3688 |
| *TGFB3* | 7043 | NM_003239 | transforming growth factor, beta 3 | -1.10 | 0.4583 | -1.13 | 0.3748 | 1.06 | 0.6436 | -1.07 | 0.5302 | -1.25 | 0.1657 | -1.18 | 0.1763 |
| *THBS1* | 7057 | NM_003246 | thrombospondin 1 | 1.18 | 0.4354 | 1.26 | 0.3504 | -1.16 | 0.5580 | 1.08 | 0.7334 | 1.49 | 0.1502 | 1.28 | 0.3277 |
| *TIMP1* | 7076 | BC000866 | TIMP metallopeptidase inhibitor 1 | 1.04 | 0.3643 | -1.05 | 0.5139 | -1.01 | 0.9288 | -1.06 | 0.3740 | -1.01 | 0.9436 | -1.01 | 0.8417 |
| *TIMP1* | 7076 | NM_003254 | TIMP metallopeptidase inhibitor 1 | -1.12 | 0.2806 | 1.12 | 0.3531 | -1.12 | 0.4304 | 1.00 | 0.9933 | 1.01 | 0.9640 | -1.12 | 0.3909 |
| *TLR4* | 7099 | NM_003266 | toll-like receptor 4 | -1.40 | 0.1133 | -1.37 | 0.2438 | 1.08 | 0.8374 | -1.27 | 0.3022 | -1.92 | 0.0627 | -1.78 | 0.0508 |
| *TLR4* | 7099 | NM_138554 | toll-like receptor 4 | -1.53 | 0.0438 | -1.01 | 0.9676 | 1.04 | 0.8800 | 1.03 | 0.8756 | -1.54 | 0.1078 | -1.48 | 0.1289 |
| *TLR4* | 7099 | NM_138557 | toll-like receptor 4 | -1.47 | 0.1281 | -1.65 | 0.1311 | 1.19 | 0.6689 | -1.39 | 0.1986 | -2.42 | 0.0335 | -2.04 | 0.0275 |
| *TNF* | 7124 | NM_000594 | tumor necrosis factor (TNF superfamily, member 2) | -1.39 | 0.0927 | 2.02 | 0.0041 | -1.35 | 0.1571 | 1.50 | 0.0238 | 1.45 | 0.1431 | 1.08 | 0.7213 |
| *TPI1* | 7167 | BC007086 | triosephosphate isomerase 1 | 1.13 | 0.0881 | -1.08 | 0.3668 | 1.12 | 0.1285 | 1.04 | 0.4611 | 1.05 | 0.5907 | 1.18 | 0.0133 |
| *TPI1* | 7167 | BC017917 | triosephosphate isomerase 1 | 1.07 | 0.1658 | -1.04 | 0.2786 | 1.02 | 0.6390 | -1.03 | 0.3475 | 1.03 | 0.6784 | 1.04 | 0.3615 |
| *TPI1* | 7167 | BC070129 | triosephosphate isomerase 1 | 1.14 | 0.0059 | -1.06 | 0.3390 | 1.11 | 0.0856 | 1.05 | 0.2818 | 1.08 | 0.2091 | 1.19 | 0.0005 |

| | | | | | | | | | | | | | | | |
|---|---|---|---|---|---|---|---|---|---|---|---|---|---|---|---|
| *TPI1* | 7167 | NM_000365 | triosephosphate isomerase 1 | 1.12 | 0.1749 | -1.08 | 0.3470 | 1.10 | 0.1741 | 1.02 | 0.5720 | 1.04 | 0.7381 | 1.15 | 0.0742 |
| *TNFRSF4* | 7293 | NM_003327 | tumor necrosis factor receptor superfamily, member 4 | -1.04 | 0.7167 | 1.33 | 0.1033 | -1.31 | 0.1163 | 1.02 | 0.8971 | 1.28 | 0.1477 | -1.03 | 0.8206 |
| *VEGF* | 7422 | AF323587 | vascular endothelial growth factor | -1.28 | 0.1189 | -1.31 | 0.1007 | 1.25 | 0.1182 | -1.05 | 0.6658 | -1.68 | 0.0164 | -1.35 | 0.0494 |
| *VEGF* | 7422 | AY263145 | vascular endothelial growth factor | -1.19 | 0.2406 | -1.16 | 0.3582 | -1.04 | 0.8296 | -1.20 | 0.2157 | -1.38 | 0.0696 | -1.42 | 0.0293 |
| *VEGF* | 7422 | M27281 | vascular endothelial growth factor | -1.19 | 0.1407 | -1.32 | 0.0646 | 1.10 | 0.4265 | -1.20 | 0.0908 | -1.57 | 0.0058 | -1.43 | 0.0023 |
| *VEGF* | 7422 | NM_001025366 | vascular endothelial growth factor | -1.30 | 0.0228 | -1.18 | 0.2998 | 1.10 | 0.5388 | -1.07 | 0.5904 | -1.53 | 0.0068 | -1.39 | 0.0066 |
| *VEGF* | 7422 | S85192 | vascular endothelial growth factor | -1.61 | 0.0261 | -1.27 | 0.2821 | 1.06 | 0.7973 | -1.20 | 0.3327 | -2.06 | 0.0118 | -1.94 | 0.0058 |
| *VHL* | 7428 | BC058831 | von Hippel-Lindau tumor suppressor | 1.25 | 0.1730 | -2.20 | 0.0006 | 1.38 | 0.1817 | -1.60 | 0.0008 | -1.76 | 0.0854 | -1.27 | 0.2543 |
| *VHL* | 7428 | L15409 | von Hippel-Lindau tumor suppressor | 1.20 | 0.3220 | -2.01 | 0.0026 | 1.36 | 0.1780 | -1.48 | 0.0091 | -1.68 | 0.0958 | -1.24 | 0.3076 |
| *VHL* | 7428 | NM_000551 | von Hippel-Lindau tumor suppressor | 1.22 | 0.2405 | -2.14 | 0.0012 | 1.42 | 0.1661 | -1.50 | 0.0059 | -1.75 | 0.0879 | -1.23 | 0.3378 |
| *VIM* | 7431 | AK093924 | vimentin | 1.32 | 0.3528 | -2.16 | 0.0217 | 1.83 | 0.0241 | -1.18 | 0.4457 | -1.64 | 0.1919 | 1.12 | 0.6776 |
| *VIM* | 7431 | BC030573 | vimentin | -1.16 | 0.3067 | -1.01 | 0.9256 | 1.00 | 0.9880 | -1.01 | 0.9344 | -1.18 | 0.4016 | -1.18 | 0.3497 |
| *VIM* | 7431 | BC066956 | vimentin | -1.14 | 0.2726 | -1.03 | 0.8114 | 1.06 | 0.6770 | 1.03 | 0.7520 | -1.18 | 0.3478 | -1.11 | 0.4815 |
| *VIM* | 7431 | NM_003380 | vimentin | -1.17 | 0.2335 | 1.01 | 0.9167 | 1.03 | 0.8531 | 1.05 | 0.6928 | -1.16 | 0.4342 | -1.12 | 0.4342 |
| *CXCR4* | 7852 | NM_001008540 | chemokine (C-X-C motif) receptor 4 | -1.04 | 0.5741 | -1.16 | 0.0405 | 1.01 | 0.8359 | -1.14 | 0.0069 | -1.20 | 0.0688 | -1.19 | 0.0238 |
| *EOMES* | 8320 | NM_005442 | eomesodermin homolog (Xenopus laevis) | 1.07 | 0.5955 | 1.44 | 0.0387 | -1.17 | 0.3554 | 1.23 | 0.1300 | 1.54 | 0.0455 | 1.31 | 0.1002 |
| *CUL2* | 8453 | NM_003591 | cullin 2 | -1.19 | 0.2100 | -1.01 | 0.9608 | -1.12 | 0.6717 | -1.13 | 0.5494 | -1.20 | 0.3554 | -1.35 | 0.1635 |
| *PIK3R3* | 8503 | AF028785 | phosphoinositide-3-kinase, regulatory subunit 3 (p55, gamma) | -1.05 | 0.7872 | -2.32 | 0.0020 | 1.61 | 0.1406 | -1.44 | 0.0757 | -2.44 | 0.0104 | -1.51 | 0.1073 |
| *PIK3R3* | 8503 | NM_003629 | phosphoinositide-3-kinase, regulatory subunit 3 (p55, gamma) | -1.40 | 0.1120 | -1.48 | 0.0836 | 1.14 | 0.5446 | -1.29 | 0.2284 | -2.07 | 0.0084 | -1.81 | 0.0160 |
| *MKNK1* | 8569 | AB000409 | MAP kinase interacting serine/threonine kinase 1 | -1.29 | 0.0812 | -1.28 | 0.0858 | 1.20 | 0.3692 | -1.07 | 0.6374 | -1.65 | 0.0240 | -1.37 | 0.0928 |

| | | | | | | | | | | | | | | | |
|---|---|---|---|---|---|---|---|---|---|---|---|---|---|---|---|
| *MKNK1* | 8569 | AK096423 | MAP kinase interacting serine/threonine kinase 1 | -1.29 | 0.0575 | -1.12 | 0.3533 | 1.06 | 0.7378 | -1.06 | 0.6537 | -1.44 | 0.0428 | -1.36 | 0.0668 |
| *MKNK1* | 8569 | NM_003684 | MAP kinase interacting serine/threonine kinase 1 | -1.31 | 0.0158 | -1.19 | 0.0538 | 1.17 | 0.2481 | -1.01 | 0.9021 | -1.56 | 0.0084 | -1.33 | 0.0465 |
| *MKNK1* | 8569 | NM_198973 | MAP kinase interacting serine/threonine kinase 1 | -1.27 | 0.1621 | -1.26 | 0.1603 | 1.19 | 0.4556 | -1.06 | 0.7150 | -1.59 | 0.0676 | -1.34 | 0.1765 |
| *TNFRSF18* | 8784 | NM_004195 | tumor necrosis factor receptor superfamily, member 18 | 1.05 | 0.6897 | 1.25 | 0.1875 | -1.05 | 0.7936 | 1.19 | 0.1857 | 1.31 | 0.1779 | 1.25 | 0.1540 |
| *TNFRSF18* | 8784 | NM_148901 | tumor necrosis factor receptor superfamily, member 18 | 1.07 | 0.6372 | 1.27 | 0.1815 | -1.11 | 0.5241 | 1.15 | 0.3761 | 1.37 | 0.0699 | 1.23 | 0.1450 |
| *NRP1* | 8829 | AF268691 | neuropilin 1 | 1.26 | 0.0635 | 1.02 | 0.8353 | -1.01 | 0.9321 | 1.01 | 0.8390 | 1.28 | 0.1010 | 1.27 | 0.0343 |
| *NRP1* | 8829 | AF280547 | neuropilin 1 | 1.15 | 0.2729 | 1.00 | 0.9764 | -1.01 | 0.9710 | -1.00 | 0.9925 | 1.16 | 0.2252 | 1.15 | 0.3234 |
| *NRP1* | 8829 | NM_001024628 | neuropilin 1 | 1.15 | 0.1049 | -1.23 | 0.0499 | 1.15 | 0.2722 | -1.06 | 0.5070 | -1.07 | 0.6040 | 1.08 | 0.4958 |
| *NRP1* | 8829 | NM_001024629 | neuropilin 1 | 1.11 | 0.2355 | -1.04 | 0.5877 | -1.05 | 0.6028 | -1.10 | 0.2590 | 1.06 | 0.5658 | 1.01 | 0.9556 |
| *NRP1* | 8829 | NM_003873 | neuropilin 1 | -1.38 | 0.1748 | -1.20 | 0.3816 | -1.01 | 0.9716 | -1.22 | 0.3607 | -1.66 | 0.0883 | -1.68 | 0.0642 |
| *EIF4E2* | 9470 | NM_004846 | eukaryotic translation initiation factor 4E member 2 | -1.14 | 0.2337 | -1.03 | 0.8561 | -1.10 | 0.5135 | -1.13 | 0.2998 | -1.17 | 0.2700 | -1.29 | 0.0350 |
| *RBX1* | 9978 | NM_014248 | ring-box 1 | 1.00 | 0.9823 | 1.02 | 0.8656 | -1.03 | 0.7854 | -1.01 | 0.9505 | 1.03 | 0.7622 | -1.01 | 0.9595 |
| *AKT3* | 10000 | NM_005465 | v-akt murine thymoma viral oncogene homolog 3 (protein kinase B, gamma) | -1.35 | 0.3184 | -2.44 | 0.0532 | 1.30 | 0.3727 | -1.88 | 0.0781 | -3.29 | 0.0043 | -2.53 | 0.0043 |
| *AKT3* | 10000 | NM_181690 | v-akt murine thymoma viral oncogene homolog 3 (protein kinase B, gamma) | -1.34 | 0.3842 | -3.41 | 0.0502 | 1.54 | 0.2416 | -2.22 | 0.0745 | -4.58 | 0.0024 | -2.98 | 0.0025 |
| *TBX21* | 30009 | NM_013351 | T-box 21 | 1.20 | 0.4451 | 1.73 | 0.0406 | -1.19 | 0.4679 | 1.46 | 0.0715 | 2.07 | 0.0365 | 1.75 | 0.0411 |
| *AK3* | 50808 | BC013771 | adenylate kinase 3 | -1.12 | 0.5736 | -1.23 | 0.3281 | 1.11 | 0.6827 | -1.11 | 0.5416 | -1.38 | 0.2560 | -1.24 | 0.3416 |
| *AK3* | 50808 | NM_016282 | adenylate kinase 3 | -1.58 | 0.1324 | -1.15 | 0.5905 | -1.12 | 0.6036 | -1.29 | 0.2884 | -1.82 | 0.0882 | -2.04 | 0.0281 |
| *FOXP3* | 50943 | NM_014009 | forkhead box P3 | 1.26 | 0.2764 | 1.19 | 0.4358 | -1.19 | 0.4381 | -1.00 | 0.9851 | 1.49 | 0.1319 | 1.25 | 0.3028 |
| *ANGPT4* | 51378 | BC111976 | angiopoietin 4 | -1.46 | 0.0231 | 1.15 | 0.1447 | -1.17 | 0.0990 | -1.02 | 0.7804 | -1.27 | 0.1862 | -1.48 | 0.0185 |
| *ANGPT4* | 51378 | BC111978 | angiopoietin 4 | -1.68 | 0.0196 | 1.74 | 0.0144 | -1.71 | 0.0238 | 1.02 | 0.8567 | 1.03 | 0.8910 | -1.65 | 0.0320 |
| *ANGPT4* | 51378 | NM_015985 | angiopoietin 4 | -1.29 | 0.0997 | 1.78 | 0.0053 | -1.41 | 0.0653 | 1.26 | 0.1369 | 1.38 | 0.1099 | -1.03 | 0.8694 |

| | | | | | | | | | | | | | | | |
|---|---|---|---|---|---|---|---|---|---|---|---|---|---|---|---|
| *EGLN1* | 54583 | BC005369 | egl nine homolog 1 (C. elegans) | -1.16 | 0.2072 | -1.15 | 0.3232 | -1.01 | 0.9740 | -1.16 | 0.4034 | -1.33 | 0.0534 | -1.34 | 0.1163 |
| *EGLN1* | 54583 | NM_022051 | egl nine homolog 1 (C. elegans) | -1.40 | 0.2053 | -1.55 | 0.2403 | 1.30 | 0.5797 | -1.19 | 0.6176 | -2.17 | 0.0473 | -1.66 | 0.1442 |
| *HIF1AN* | 55662 | AK025680 | hypoxia-inducible factor 1, alpha subunit inhibitor | -1.11 | 0.4216 | -1.41 | 0.0401 | 1.10 | 0.6574 | -1.28 | 0.1075 | -1.56 | 0.0403 | -1.41 | 0.0699 |
| *HIF1AN* | 55662 | BC007719 | hypoxia-inducible factor 1, alpha subunit inhibitor | -1.31 | 0.1362 | -1.44 | 0.0515 | 1.17 | 0.5028 | -1.24 | 0.1690 | -1.89 | 0.0268 | -1.62 | 0.0358 |
| *HIF1AN* | 55662 | NM_017902 | hypoxia-inducible factor 1, alpha subunit inhibitor | -1.09 | 0.1042 | -1.11 | 0.1189 | 1.02 | 0.8137 | -1.09 | 0.1679 | -1.21 | 0.0398 | -1.18 | 0.0389 |
| *HAMP* | 57817 | NM_021175 | hepcidin antimicrobial peptide | 1.29 | 0.3593 | 1.91 | 0.0159 | -1.30 | 0.2782 | 1.48 | 0.1003 | 2.46 | 0.0126 | 1.90 | 0.0434 |
| *HKDC1* | 80201 | AK026414 | hexokinase domain containing 1 | -1.02 | 0.8197 | 1.37 | 0.0670 | -1.29 | 0.1403 | 1.06 | 0.5519 | 1.34 | 0.1332 | 1.04 | 0.7325 |
| *HKDC1* | 80201 | BC110504 | hexokinase domain containing 1 | -1.07 | 0.5432 | 1.53 | 0.0055 | -1.40 | 0.0285 | 1.09 | 0.4846 | 1.43 | 0.0242 | 1.02 | 0.8876 |
| *HKDC1* | 80201 | NM_025130 | hexokinase domain containing 1 | -1.01 | 0.9644 | 1.42 | 0.0226 | -1.24 | 0.0951 | 1.15 | 0.3057 | 1.41 | 0.0318 | 1.14 | 0.3453 |
| *PROK1* | 84432 | NM_032414 | prokineticin 1 | 1.26 | 0.4256 | 1.21 | 0.4218 | 1.06 | 0.8340 | 1.28 | 0.3445 | 1.52 | 0.1639 | 1.61 | 0.1574 |
| *RETNLB* | 84666 | BC069318 | resistin like beta | 1.19 | 0.4838 | 2.13 | 0.0111 | -1.57 | 0.0907 | 1.36 | 0.1409 | 2.54 | 0.0191 | 1.61 | 0.1016 |
| *RETNLB* | 84666 | NM_032579 | resistin like beta | 1.28 | 0.3437 | 1.37 | 0.1248 | -1.03 | 0.8886 | 1.33 | 0.2405 | 1.75 | 0.0449 | 1.69 | 0.1018 |
| *EGLN2* | 112398 | NM_017555 | egl nine homolog 2 (C. elegans) | -1.20 | 0.1551 | -1.28 | 0.0974 | 1.22 | 0.2817 | -1.04 | 0.7143 | -1.53 | 0.0414 | -1.25 | 0.1645 |
| *EGLN2* | 112398 | NM_053046 | egl nine homolog 2 (C. elegans) | -1.11 | 0.2026 | -1.25 | 0.0387 | 1.22 | 0.1651 | -1.02 | 0.8101 | -1.39 | 0.0397 | -1.13 | 0.2909 |
| *EGLN3* | 112399 | AK123350 | egl nine homolog 3 (C. elegans) | 1.13 | 0.1824 | 1.17 | 0.2131 | -1.06 | 0.6841 | 1.10 | 0.3225 | 1.32 | 0.0949 | 1.25 | 0.0957 |
| *EGLN3* | 112399 | NM_022073 | egl nine homolog 3 (C. elegans) | 1.17 | 0.1895 | -1.02 | 0.8464 | -1.01 | 0.9059 | -1.03 | 0.7605 | 1.14 | 0.0988 | 1.13 | 0.1497 |
| *FLJ36951* | 253314 | XM_171094 | similar to eukaryotic translation initiation factor 4e 1a | 1.21 | 0.5588 | 2.17 | 0.0247 | -1.68 | 0.1317 | 1.29 | 0.3574 | 2.62 | 0.0448 | 1.56 | 0.2823 |
| *FLJ36951* | 253314 | XM_927854 | similar to eukaryotic translation initiation factor 4e 1a | 1.33 | 0.2629 | 1.10 | 0.6328 | -1.03 | 0.8633 | 1.07 | 0.7267 | 1.47 | 0.0311 | 1.43 | 0.0797 |
| *FLJ36951* | 253314 | XM_932348 | similar to eukaryotic translation initiation factor 4e 1a | 1.56 | 0.1545 | 1.74 | 0.0465 | -1.34 | 0.2911 | 1.30 | 0.3225 | 2.72 | 0.0139 | 2.03 | 0.0642 |

| | | | | | | | | | | | | | |
|---|---|---|---|---|---|---|---|---|---|---|---|---|---|
| *DKFZp781N1041* | 387712 | XM_370577 | similar to RIKEN cDNA 6430537H07 gene | 1.25 | 0.2657 | -1.31 | 0.1892 | 1.21 | 0.3742 | -1.08 | 0.6946 | -1.05 | 0.7669 | 1.15 | 0.4935 |

**Supplementary Table S5.** Expression fold changes of 176 HIF-related genes represented by 375 probe set IDs analyzed in human U937 cells exposed to different gravitational conditions during a suborbital ballistic rocket flight (TEXUS-49). The analysis was made with the NimbleGen expression microarray based on the hg18 annotation.

| | | | | **µg vs BL-TX hyp-g** | | **BL-TX hyp-g vs HW 1g GC** | | **µg vs HW 1g GC** | |
|---|---|---|---|---|---|---|---|---|---|
| **Gene Symbol** | **Entrez Gene ID** | **Probe set ID** | **Description** | **Fold Change** | **p-value** | **Fold Change** | **p-value** | **Fold Change** | **p-value** |
| *ADM* | 133 | NM_001124 | adrenomedullin | 1.43 | 0.1949 | 1.07 | 0.6203 | 1.53 | 0.1034 |
| *ADORA2A* | 135 | BC013780 | adenosine A2a receptor | -1.41 | 0.3167 | -1.44 | 0.3685 | -2.03 | 0.1069 |
| *ADORA2A* | 135 | NM_000675 | adenosine A2a receptor | 1.05 | 0.8061 | -1.33 | 0.1953 | -1.26 | 0.2288 |
| *ADRA1B* | 147 | NM_000679 | adrenergic, alpha-1B-, receptor | 1.84 | 0.0885 | -1.88 | 0.1500 | -1.02 | 0.9419 |
| *AKT1* | 207 | BC084538 | v-akt murine thymoma viral oncogene homolog 1 | -1.19 | 0.1811 | 1.23 | 0.1489 | 1.03 | 0.7966 |
| *AKT1* | 207 | BX647722 | v-akt murine thymoma viral oncogene homolog 1 | 1.70 | 0.1578 | -1.31 | 0.2707 | 1.29 | 0.3372 |
| *AKT1* | 207 | NM_001014431 | v-akt murine thymoma viral oncogene homolog 1 | -1.11 | 0.3300 | 1.10 | 0.4325 | -1.01 | 0.9040 |
| *AKT2* | 208 | NM_001626 | v-akt murine thymoma viral oncogene homolog 2 | -1.35 | 0.3026 | -1.20 | 0.4833 | -1.62 | 0.0895 |
| *ALDOA* | 226 | CR592372 | aldolase A, fructose-bisphosphate | 1.06 | 0.1023 | -1.07 | 0.0559 | -1.01 | 0.8368 |
| *ALDOA* | 226 | NM_000034 | aldolase A, fructose-bisphosphate | -1.02 | 0.4955 | -1.04 | 0.2344 | -1.06 | 0.0502 |
| *ALDOA* | 226 | NM_184041 | aldolase A, fructose-bisphosphate | 1.02 | 0.5277 | -1.00 | 0.9843 | 1.01 | 0.5904 |
| *ALDOC* | 230 | BC106925 | aldolase C, fructose-bisphosphate | -1.30 | 0.3143 | -1.13 | 0.5555 | -1.47 | 0.0980 |
| *ALDOC* | 230 | NM_005165 | aldolase C, fructose-bisphosphate | -1.32 | 0.2574 | 1.21 | 0.4058 | -1.09 | 0.7259 |
| *ANGPT1* | 284 | BC029406 | angiopoietin 1 | -1.44 | 0.0345 | -1.02 | 0.9488 | -1.46 | 0.1090 |
| *ANGPT1* | 284 | NM_001146 | angiopoietin 1 | 1.00 | 0.9727 | 1.05 | 0.2889 | 1.05 | 0.4474 |
| *ANGPT2* | 285 | AF187858 | angiopoietin 2 | 1.39 | 0.0130 | -1.52 | 0.2336 | -1.09 | 0.7298 |
| *ANGPT2* | 285 | AF218015 | angiopoietin 2 | 1.10 | 0.5773 | -1.03 | 0.8690 | 1.07 | 0.6512 |
| *ANGPT2* | 285 | NM_001147 | angiopoietin 2 | 1.00 | 0.9852 | 1.01 | 0.9302 | 1.01 | 0.9344 |
| *ARG1* | 383 | BC020653 | arginase, liver | 1.07 | 0.5715 | -1.13 | 0.2970 | -1.05 | 0.5997 |
| *ARG1* | 383 | NM_000045 | arginase, liver | 1.08 | 0.6857 | -1.17 | 0.4386 | -1.09 | 0.6395 |
| *ARNT* | 405 | AB209877 | aryl hydrocarbon receptor nuclear translocator | -1.53 | 0.1571 | 1.11 | 0.6624 | -1.37 | 0.3298 |
| *ARNT* | 405 | BC060838 | aryl hydrocarbon receptor nuclear translocator | -1.75 | 0.1459 | 1.88 | 0.0451 | 1.07 | 0.8814 |
| *ARNT* | 405 | NM_001668 | aryl hydrocarbon receptor nuclear translocator | -1.81 | 0.0891 | 1.63 | 0.0658 | -1.11 | 0.8025 |
| *BCL2* | 596 | BC027258 | B-cell CLL/lymphoma 2 | -1.64 | 0.1676 | 1.63 | 0.1292 | -1.01 | 0.9879 |
| *BCL2* | 596 | NM_000633 | B-cell CLL/lymphoma 2 | -1.90 | 0.1412 | 3.88 | 0.0057 | 2.04 | 0.2525 |
| *BCL2* | 596 | NM_000657 | B-cell CLL/lymphoma 2 | 1.75 | 0.1024 | -1.45 | 0.0792 | 1.21 | 0.4610 |

| | | | | | | | | | |
|---|---|---|---|---|---|---|---|---|---|
| *BLR1* | 643 | NM_001716 | Burkitt lymphoma receptor 1, GTP binding protein (chemokine (C-X-C motif) receptor 5) | 3.86 | 0.0825 | -2.71 | 0.1564 | 1.42 | 0.4852 |
| *CA9* | 768 | NM_001216 | carbonic anhydrase IX | 3.12 | 0.1189 | -2.27 | 0.1005 | 1.37 | 0.5098 |
| *CAMK2A* | 815 | AF145710 | calcium/calmodulin-dependent protein kinase (CaM kinase) II alpha | 1.75 | 0.2483 | -1.45 | 0.3989 | 1.20 | 0.6126 |
| *CAMK2A* | 815 | AF145711 | calcium/calmodulin-dependent protein kinase (CaM kinase) II alpha | 2.36 | 0.0895 | -1.90 | 0.1880 | 1.24 | 0.5847 |
| *CAMK2A* | 815 | NM_015981 | calcium/calmodulin-dependent protein kinase (CaM kinase) II alpha | 1.82 | 0.0827 | -2.37 | 0.0529 | -1.30 | 0.4063 |
| *CAMK2B* | 816 | NM_001220 | calcium/calmodulin-dependent protein kinase (CaM kinase) II beta | 6.23 | 0.1142 | -1.81 | 0.3716 | 3.45 | 0.1387 |
| *CAMK2B* | 816 | NM_172078 | calcium/calmodulin-dependent protein kinase (CaM kinase) II beta | 4.25 | 0.1675 | -1.56 | 0.5172 | 2.74 | 0.2051 |
| *CAMK2B* | 816 | NM_172080 | calcium/calmodulin-dependent protein kinase (CaM kinase) II beta | 6.13 | 0.1390 | -2.37 | 0.2964 | 2.59 | 0.2429 |
| *CAMK2B* | 816 | NM_172081 | calcium/calmodulin-dependent protein kinase (CaM kinase) II beta | 8.43 | 0.0959 | -3.14 | 0.2312 | 2.69 | 0.2026 |
| *CAMK2B* | 816 | NM_172082 | calcium/calmodulin-dependent protein kinase (CaM kinase) II beta | 7.21 | 0.1533 | -2.07 | 0.3023 | 3.48 | 0.1950 |
| *CAMK2B* | 816 | NM_172084 | calcium/calmodulin-dependent protein kinase (CaM kinase) II beta | 3.70 | 0.1397 | -2.06 | 0.0981 | 1.80 | 0.3246 |
| *CAMK2D* | 817 | NM_172115 | calcium/calmodulin-dependent protein kinase (CaM kinase) II delta | -1.57 | 0.2100 | 1.66 | 0.1105 | 1.06 | 0.8807 |
| *CAMK2D* | 817 | AB209288 | calcium/calmodulin-dependent protein kinase (CaM kinase) II delta | -1.10 | 0.0291 | 1.07 | 0.4607 | -1.03 | 0.7303 |
| *CAMK2D* | 817 | NM_001221 | calcium/calmodulin-dependent protein kinase (CaM kinase) II delta | -1.90 | 0.1017 | 2.50 | 0.0139 | 1.31 | 0.5389 |
| *CAMK2D* | 817 | NM_172127 | calcium/calmodulin-dependent protein kinase (CaM kinase) II delta | -1.78 | 0.1799 | 4.60 | 0.0046 | 2.58 | 0.1478 |
| *CAMK2G* | 818 | NM_001222 | calcium/calmodulin-dependent protein kinase (CaM kinase) II gamma | -1.28 | 0.0565 | -1.05 | 0.6140 | -1.34 | 0.0100 |
| *CAMP* | 820 | BC055089 | cathelicidin antimicrobial peptide | 1.64 | 0.0892 | -1.27 | 0.1654 | 1.29 | 0.3013 |
| *CAMP* | 820 | NM_004345 | cathelicidin antimicrobial peptide | 1.80 | 0.1168 | -2.03 | 0.0614 | -1.13 | 0.6650 |
| *CD36* | 948 | BC008406 | CD36 molecule (thrombospondin receptor) | -1.37 | 0.0507 | 1.58 | 0.0082 | 1.15 | 0.2964 |
| *CD36* | 948 | NM_000072 | CD36 molecule (thrombospondin receptor) | -2.11 | 0.0199 | 2.33 | 0.0003 | 1.11 | 0.7990 |
| *CD36* | 948 | NM_001001548 | CD36 molecule (thrombospondin receptor) | -1.67 | 0.0758 | 1.96 | 0.0312 | 1.17 | 0.3875 |
| *CDKN1A* | 1026 | NM_000389 | cyclin-dependent kinase inhibitor 1A (p21, Cip1) | -1.01 | 0.8519 | 1.01 | 0.9309 | -1.01 | 0.9496 |
| *CDKN1A* | 1026 | NM_078467 | cyclin-dependent kinase inhibitor 1A (p21, Cip1) | 1.02 | 0.6913 | -1.05 | 0.6080 | -1.03 | 0.6923 |
| *CDKN1B* | 1027 | NM_004064 | cyclin-dependent kinase inhibitor 1B (p27, Kip1) | -2.35 | 0.0083 | 2.23 | 0.0006 | -1.05 | 0.8952 |

| | | | | | | | | | |
|---|---|---|---|---|---|---|---|---|---|
| *CCR1* | 1230 | BC051306 | chemokine (C-C motif) receptor 1 | -1.78 | 0.0392 | 2.35 | 0.0001 | 1.32 | 0.4458 |
| *CCR1* | 1230 | NM_001295 | chemokine (C-C motif) receptor 1 | -1.41 | 0.0818 | 1.55 | 0.0384 | 1.10 | 0.6812 |
| *CCR5* | 1234 | BC038398 | chemokine (C-C motif) receptor 5 | 1.40 | 0.2362 | -1.39 | 0.2173 | 1.01 | 0.9697 |
| *CCR5* | 1234 | NM_000579 | chemokine (C-C motif) receptor 5 | 1.35 | 0.3647 | -1.06 | 0.7782 | 1.28 | 0.3827 |
| *CCR7* | 1236 | NM_001838 | chemokine (C-C motif) receptor 7 | 1.73 | 0.2080 | -1.26 | 0.5868 | 1.38 | 0.4080 |
| *CREBBP* | 1387 | NM_004380 | CREB binding protein (Rubinstein-Taybi syndrome) | -1.91 | 0.1097 | 4.69 | 0.0038 | 2.46 | 0.1153 |
| *CREBBP* | 1387 | U85962 | CREB binding protein (Rubinstein-Taybi syndrome) | -1.49 | 0.2553 | 1.53 | 0.2221 | 1.03 | 0.9388 |
| *MAPK14* | 1432 | BC000092 | mitogen-activated protein kinase 14 | -1.40 | 0.0671 | 1.40 | 0.0056 | -1.00 | 0.9858 |
| *MAPK14* | 1432 | NM_001315 | mitogen-activated protein kinase 14 | -1.67 | 0.0318 | 1.57 | 0.0022 | -1.06 | 0.8102 |
| *MAPK14* | 1432 | NM_139013 | mitogen-activated protein kinase 14 | -1.50 | 0.0596 | 1.41 | 0.0284 | -1.07 | 0.7564 |
| *CTLA4* | 1493 | BC069566 | cytotoxic T-lymphocyte-associated protein 4 | 1.03 | 0.8638 | -1.34 | 0.0692 | -1.30 | 0.0514 |
| *CTLA4* | 1493 | BC074842 | cytotoxic T-lymphocyte-associated protein 4 | 1.29 | 0.3802 | -1.05 | 0.8126 | 1.23 | 0.3701 |
| *CTLA4* | 1493 | BC074893 | cytotoxic T-lymphocyte-associated protein 4 | 1.03 | 0.9259 | 1.10 | 0.6445 | 1.13 | 0.6395 |
| *CTLA4* | 1493 | NM_001037631 | cytotoxic T-lymphocyte-associated protein 4 | 2.44 | 0.2634 | -3.13 | 0.2735 | -1.29 | 0.7089 |
| *CTLA4* | 1493 | NM_005214 | cytotoxic T-lymphocyte-associated protein 4 | 3.32 | 0.2038 | -3.51 | 0.1914 | -1.06 | 0.9304 |
| *CTSD* | 1509 | NM_001909 | cathepsin D (lysosomal aspartyl peptidase) | 1.02 | 0.7466 | -1.10 | 0.1252 | -1.08 | 0.0488 |
| *CYBB* | 1536 | BC032720 | cytochrome b-245, beta polypeptide (chronic granulomatous disease) | -1.97 | 0.0596 | 1.82 | 0.0086 | -1.08 | 0.8501 |
| *CYBB* | 1536 | NM_000397 | cytochrome b-245, beta polypeptide (chronic granulomatous disease) | -1.96 | 0.0771 | 1.79 | 0.0101 | -1.09 | 0.8414 |
| *DEFB1* | 1672 | BC033298 | defensin, beta 1 | -1.40 | 0.1198 | -1.40 | 0.0660 | -1.96 | 0.0026 |
| *DEFB1* | 1672 | NM_005218 | defensin, beta 1 | 2.30 | 0.1222 | -2.86 | 0.2303 | -1.24 | 0.6903 |
| *EDG1* | 1901 | NM_001400 | endothelial differentiation, sphingolipid G-protein-coupled receptor, 1 | 1.72 | 0.3280 | -1.61 | 0.4006 | 1.07 | 0.8794 |
| *EDN1* | 1906 | CR605456 | endothelin 1 | 1.05 | 0.4277 | -1.01 | 0.9131 | 1.04 | 0.5859 |
| *EDN1* | 1906 | NM_001955 | endothelin 1 | -1.05 | 0.6028 | -1.46 | 0.1153 | -1.53 | 0.0426 |
| *EGF* | 1950 | BC093731 | epidermal growth factor (beta-urogastrone) | 1.25 | 0.1117 | -1.28 | 0.1467 | -1.03 | 0.8541 |
| *EGF* | 1950 | NM_001963 | epidermal growth factor (beta-urogastrone) | 1.06 | 0.7720 | 1.00 | 0.9864 | 1.07 | 0.7461 |
| *EGFR* | 1956 | BC094761 | epidermal growth factor receptor (erythroblastic leukemia viral (v-erb-b) oncogene hom | 3.37 | 0.1872 | -2.62 | 0.0903 | 1.28 | 0.6634 |
| *EGFR* | 1956 | K03193 | epidermal growth factor receptor (erythroblastic leukemia viral (v-erb-b) oncogene hom | 1.74 | 0.1685 | -1.73 | 0.2068 | 1.00 | 0.9906 |
| *EGFR* | 1956 | NM_005228 | epidermal growth factor receptor (erythroblastic leukemia viral (v-erb-b) oncogene hom | 1.07 | 0.6787 | -1.29 | 0.1514 | -1.21 | 0.2223 |
| *EGFR* | 1956 | NM_201282 | epidermal growth factor receptor (erythroblastic leukemia viral (v-erb-b) oncogene hom | 1.15 | 0.5976 | -1.69 | 0.0231 | -1.46 | 0.1041 |

| | | | | | | | | | |
|---|---|---|---|---|---|---|---|---|---|
| *EGFR* | 1956 | NM_201283 | epidermal growth factor receptor (erythroblastic leukemia viral (v-erb-b) oncogene hom | 4.04 | 0.0789 | -3.11 | 0.2448 | 1.30 | 0.6442 |
| *EGFR* | 1956 | NM_201284 | epidermal growth factor receptor (erythroblastic leukemia viral (v-erb-b) oncogene hom | 1.51 | 0.1358 | -1.23 | 0.2669 | 1.23 | 0.3806 |
| *EIF4E* | 1977 | BC012611 | eukaryotic translation initiation factor 4E | -2.11 | 0.0161 | 2.00 | 0.0001 | -1.06 | 0.8745 |
| *EIF4E* | 1977 | BC035166 | eukaryotic translation initiation factor 4E | 1.24 | 0.4388 | -1.07 | 0.7718 | 1.17 | 0.5615 |
| *EIF4E* | 1977 | NM_001968 | eukaryotic translation initiation factor 4E | -2.07 | 0.0137 | 2.15 | 0.0001 | 1.04 | 0.9198 |
| *EIF4EBP1* | 1978 | BC058073 | eukaryotic translation initiation factor 4E binding protein 1 | 1.03 | 0.3281 | -1.03 | 0.5313 | 1.00 | 0.9654 |
| *EIF4EBP1* | 1978 | NM_004095 | eukaryotic translation initiation factor 4E binding protein 1 | 1.04 | 0.2187 | -1.05 | 0.1690 | -1.01 | 0.7900 |
| *ENG* | 2022 | BC014271 | endoglin (Osler-Rendu-Weber syndrome 1) | -1.10 | 0.3934 | -1.14 | 0.3146 | -1.25 | 0.0560 |
| *ENG* | 2022 | NM_000118 | endoglin (Osler-Rendu-Weber syndrome 1) | -1.09 | 0.5051 | -1.09 | 0.5411 | -1.18 | 0.1883 |
| *ENO1* | 2023 | BC050642 | enolase 1, (alpha) | -1.01 | 0.7349 | 1.03 | 0.3931 | 1.02 | 0.5191 |
| *ENO1* | 2023 | BC073991 | enolase 1, (alpha) | -1.01 | 0.6366 | 1.01 | 0.5181 | -1.01 | 0.8360 |
| *ENO1* | 2023 | NM_001428 | enolase 1, (alpha) | -1.02 | 0.6153 | 1.00 | 0.9342 | -1.02 | 0.6520 |
| *ENO2* | 2026 | NM_001975 | enolase 2 (gamma, neuronal) | -1.15 | 0.4581 | 1.04 | 0.8147 | -1.10 | 0.5669 |
| *ENO3* | 2027 | BC017249 | enolase 3 (beta, muscle) | -1.05 | 0.7402 | -1.05 | 0.8014 | -1.10 | 0.5619 |
| *ENO3* | 2027 | NM_001976 | enolase 3 (beta, muscle) | -1.08 | 0.6540 | -1.01 | 0.9636 | -1.09 | 0.6859 |
| *ENO3* | 2027 | NM_053013 | enolase 3 (beta, muscle) | -1.04 | 0.8178 | -1.07 | 0.7124 | -1.11 | 0.5472 |
| *EP300* | 2033 | NM_001429 | E1A binding protein p300 | -1.51 | 0.0782 | 1.51 | 0.0543 | -1.00 | 0.9936 |
| *EPAS1* | 2034 | NM_001430 | endothelial PAS domain protein 1 | -1.60 | 0.0683 | 2.05 | 0.0079 | 1.28 | 0.4576 |
| *EPO* | 2056 | BC093628 | erythropoietin | 3.60 | 0.0896 | -1.93 | 0.2031 | 1.87 | 0.2320 |
| *EPO* | 2056 | NM_000799 | erythropoietin | 2.49 | 0.0260 | -1.83 | 0.0230 | 1.36 | 0.2797 |
| *ERBB2* | 2064 | AF177761 | v-erb-b2 erythroblastic leukemia viral oncogene homolog 2, neuro/glioblastoma derived | 3.01 | 0.0985 | -1.78 | 0.2898 | 1.69 | 0.2770 |
| *ERBB2* | 2064 | AK131568 | v-erb-b2 erythroblastic leukemia viral oncogene homolog 2, neuro/glioblastoma derived | 2.25 | 0.0642 | -1.61 | 0.1225 | 1.40 | 0.3099 |
| *ERBB2* | 2064 | NM_001005862 | v-erb-b2 erythroblastic leukemia viral oncogene homolog 2, neuro/glioblastoma derived | 1.36 | 0.2392 | 1.16 | 0.3930 | 1.58 | 0.0937 |
| *FLT1* | 2321 | BC039007 | fms-related tyrosine kinase 1 (vascular endothelial growth factor/vascular permeabilit | -1.94 | 0.0767 | 3.30 | 0.0052 | 1.70 | 0.2047 |
| *FLT1* | 2321 | NM_002019 | fms-related tyrosine kinase 1 (vascular endothelial growth factor/vascular permeabilit | 1.06 | 0.4494 | -1.05 | 0.5351 | 1.01 | 0.9073 |
| *FN1* | 2335 | AB191261 | fibronectin 1 | 1.01 | 0.9723 | -1.41 | 0.3318 | -1.40 | 0.2898 |

| | | | | | | | | | |
|---|---|---|---|---|---|---|---|---|---|
| *FN1* | 2335 | BX538018 | fibronectin 1 | -1.09 | 0.6871 | -1.31 | 0.2164 | -1.42 | 0.1056 |
| *FN1* | 2335 | CR749317 | fibronectin 1 | 1.08 | 0.5974 | -1.03 | 0.7967 | 1.04 | 0.7416 |
| *FN1* | 2335 | NM_002026 | fibronectin 1 | -1.18 | 0.3942 | -1.04 | 0.8979 | -1.22 | 0.4103 |
| *FN1* | 2335 | NM_054034 | fibronectin 1 | -1.06 | 0.6108 | -1.21 | 0.3515 | -1.28 | 0.2044 |
| *FRAP1* | 2475 | NM_004958 | FK506 binding protein 12-rapamycin associated protein 1 | -1.33 | 0.0519 | 1.36 | 0.0179 | 1.03 | 0.8719 |
| *GAPDH* | 2597 | BC001601 | glyceraldehyde-3-phosphate dehydrogenase | 1.03 | 0.4748 | 1.05 | 0.1872 | 1.08 | 0.0694 |
| *GAPDH* | 2597 | BC009081 | glyceraldehyde-3-phosphate dehydrogenase | 1.05 | 0.3451 | 1.05 | 0.4575 | 1.10 | 0.1206 |
| *GAPDH* | 2597 | NM_002046 | glyceraldehyde-3-phosphate dehydrogenase | -1.00 | 0.9516 | 1.08 | 0.0954 | 1.08 | 0.0738 |
| *GPI* | 2821 | BC004982 | glucose phosphate isomerase | -1.02 | 0.6728 | -1.00 | 0.9626 | -1.02 | 0.6383 |
| *GPI* | 2821 | NM_000175 | glucose phosphate isomerase | -1.06 | 0.4306 | -1.01 | 0.9507 | -1.07 | 0.3862 |
| *MKNK2* | 2872 | AF237775 | MAP kinase interacting serine/threonine kinase 2 | -1.07 | 0.7128 | 1.03 | 0.8862 | -1.04 | 0.7920 |
| *MKNK2* | 2872 | NM_017572 | MAP kinase interacting serine/threonine kinase 2 | -1.10 | 0.6397 | 1.00 | 0.9871 | -1.09 | 0.6199 |
| *MKNK2* | 2872 | NM_199054 | MAP kinase interacting serine/threonine kinase 2 | -1.02 | 0.8505 | 1.17 | 0.1181 | 1.15 | 0.1581 |
| *CXCL2* | 2920 | BC015753 | chemokine (C-X-C motif) ligand 2 | 1.51 | 0.0897 | -1.08 | 0.4705 | 1.40 | 0.1115 |
| *CXCL2* | 2920 | NM_002089 | chemokine (C-X-C motif) ligand 2 | -1.22 | 0.4470 | 1.27 | 0.3324 | 1.05 | 0.8474 |
| *HDAC2* | 3066 | NM_001527 | histone deacetylase 2 | -1.35 | 0.0472 | 1.54 | 0.0013 | 1.14 | 0.4208 |
| *HIF1A* | 3091 | NM_001530 | hypoxia-inducible factor 1, alpha subunit (basic helix-loop-helix transcription factor | -1.99 | 0.0630 | 2.28 | 0.0044 | 1.14 | 0.7872 |
| *HIF1A* | 3091 | NM_181054 | hypoxia-inducible factor 1, alpha subunit (basic helix-loop-helix transcription factor | -2.02 | 0.0642 | 2.49 | 0.0013 | 1.23 | 0.6765 |
| *HK1* | 3098 | NM_000188 | hexokinase 1 | -1.46 | 0.1643 | -1.10 | 0.5756 | -1.61 | 0.0494 |
| *HK2* | 3099 | BC021116 | hexokinase 2 | -1.58 | 0.1550 | 1.31 | 0.2793 | -1.21 | 0.5156 |
| *HK2* | 3099 | NM_000189 | hexokinase 2 | -1.86 | 0.0399 | 2.19 | 0.0001 | 1.18 | 0.6742 |
| *HK3* | 3101 | NM_002115 | hexokinase 3 (white cell) | -1.30 | 0.3826 | -1.12 | 0.7366 | -1.46 | 0.2607 |
| *HMOX1* | 3162 | BC001491 | heme oxygenase (decycling) 1 | -1.36 | 0.1515 | -1.01 | 0.9726 | -1.37 | 0.2655 |
| *HMOX1* | 3162 | NM_002133 | heme oxygenase (decycling) 1 | -1.36 | 0.2212 | -1.12 | 0.6770 | -1.51 | 0.1727 |
| *ICAM1* | 3383 | NM_000201 | intercellular adhesion molecule 1 (CD54), human rhinovirus receptor | 1.00 | 0.9534 | -1.00 | 0.9879 | 1.00 | 0.9855 |
| *IFNG* | 3458 | NM_000619 | interferon, gamma | -1.19 | 0.3501 | 1.06 | 0.7843 | -1.12 | 0.3547 |
| *IFNGR1* | 3459 | NM_000416 | interferon gamma receptor 1 | -2.10 | 0.0155 | 2.26 | 0.0000 | 1.08 | 0.8488 |
| *IFNGR2* | 3460 | NM_005534 | interferon gamma receptor 2 (interferon gamma transducer 1) | -1.29 | 0.0687 | 1.39 | 0.0084 | 1.08 | 0.6172 |
| *IGF1* | 3479 | M11568 | insulin-like growth factor 1 (somatomedin C) | 2.74 | 0.2295 | -2.18 | 0.2316 | 1.25 | 0.6772 |
| *IGF1* | 3479 | M29644 | insulin-like growth factor 1 (somatomedin C) | 1.75 | 0.0825 | -1.82 | 0.1015 | -1.04 | 0.9040 |
| *IGF1* | 3479 | M37484 | insulin-like growth factor 1 (somatomedin C) | 2.56 | 0.1323 | -1.52 | 0.4416 | 1.69 | 0.2905 |
| *IGF1* | 3479 | NM_000618 | insulin-like growth factor 1 (somatomedin C) | -1.23 | 0.0539 | 1.00 | 0.9671 | -1.22 | 0.0283 |
| *IGF1R* | 3480 | NM_000875 | insulin-like growth factor 1 receptor | -1.33 | 0.3047 | 1.17 | 0.5196 | -1.14 | 0.5914 |

| | | | | | | | | | |
|---|---|---|---|---|---|---|---|---|---|
| *IGF2* | 3481 | BC053318 | insulin-like growth factor 2 (somatomedin A) | 2.10 | 0.2741 | -1.06 | 0.8777 | 1.99 | 0.2508 |
| *IGF2* | 3481 | NM_000612 | insulin-like growth factor 2 (somatomedin A) | 3.59 | 0.1887 | -1.57 | 0.2147 | 2.28 | 0.2551 |
| *IGFBP1* | 3484 | NM_000596 | insulin-like growth factor binding protein 1 | 1.06 | 0.6414 | -1.46 | 0.4366 | -1.38 | 0.4207 |
| *IGFBP1* | 3484 | NM_001013029 | insulin-like growth factor binding protein 1 | 1.30 | 0.1376 | -1.34 | 0.2030 | -1.03 | 0.8642 |
| *IGFBP2* | 3485 | NM_000597 | insulin-like growth factor binding protein 2, 36kDa | -1.27 | 0.1459 | 1.15 | 0.2040 | -1.11 | 0.4315 |
| *IGFBP3* | 3486 | BC000013 | insulin-like growth factor binding protein 3 | 1.28 | 0.3060 | -1.08 | 0.6052 | 1.19 | 0.4271 |
| *IGFBP3* | 3486 | NM_000598 | insulin-like growth factor binding protein 3 | 1.39 | 0.2040 | -1.31 | 0.0475 | 1.06 | 0.7601 |
| *IL1B* | 3553 | NM_000576 | interleukin 1, beta | -1.43 | 0.0457 | 1.28 | 0.0754 | -1.12 | 0.5714 |
| *IL6* | 3569 | NM_000600 | interleukin 6 (interferon, beta 2) | 1.84 | 0.2540 | -1.70 | 0.1250 | 1.08 | 0.8511 |
| *IL6R* | 3570 | BC089410 | interleukin 6 receptor | -1.26 | 0.2663 | 1.22 | 0.2861 | -1.03 | 0.8402 |
| *IL6R* | 3570 | NM_000565 | interleukin 6 receptor | -1.57 | 0.1549 | 1.42 | 0.1157 | -1.11 | 0.7363 |
| *IL8* | 3576 | NM_000584 | interleukin 8 | -1.98 | 0.0402 | 2.17 | 0.0005 | 1.10 | 0.8284 |
| *IL12A* | 3592 | BC104982 | interleukin 12A (natural killer cell stimulatory factor 1, cytotoxic lymphocyte matura | -1.35 | 0.3592 | 1.73 | 0.0602 | 1.29 | 0.4594 |
| *IL12A* | 3592 | NM_000882 | interleukin 12A (natural killer cell stimulatory factor 1, cytotoxic lymphocyte matura | -1.15 | 0.3370 | 1.06 | 0.6569 | -1.09 | 0.5773 |
| *TNFRSF9* | 3604 | NM_001561 | tumor necrosis factor receptor superfamily, member 9 | -1.00 | 0.9909 | -1.50 | 0.0860 | -1.50 | 0.0650 |
| *INS* | 3630 | BC005255 | insulin | 1.13 | 0.7000 | -1.29 | 0.4240 | -1.14 | 0.6867 |
| *INS* | 3630 | NM_000207 | insulin | 6.45 | 0.1320 | -2.05 | 0.0489 | 3.15 | 0.1742 |
| *INSR* | 3643 | NM_000208 | insulin receptor | -1.54 | 0.2439 | 1.14 | 0.6413 | -1.34 | 0.3929 |
| *INSR* | 3643 | X02160 | insulin receptor | -1.60 | 0.0725 | 1.64 | 0.0110 | 1.02 | 0.9402 |
| *ITGAM* | 3684 | BC096346 | integrin, alpha M (complement component 3 receptor 3 subunit) | -1.14 | 0.3263 | -1.02 | 0.8850 | -1.17 | 0.2321 |
| *ITGAM* | 3684 | J03925 | integrin, alpha M (complement component 3 receptor 3 subunit) | -1.41 | 0.0913 | 1.99 | 0.0021 | 1.41 | 0.1932 |
| *ITGAM* | 3684 | NM_000632 | integrin, alpha M (complement component 3 receptor 3 subunit) | -1.18 | 0.3668 | 1.02 | 0.9082 | -1.15 | 0.4582 |
| *ITGB2* | 3689 | AK097864 | integrin, beta 2 (complement component 3 receptor 3 and 4 subunit) | 2.12 | 0.0238 | -2.08 | 0.0067 | 1.02 | 0.9239 |
| *ITGB2* | 3689 | NM_000211 | integrin, beta 2 (complement component 3 receptor 3 and 4 subunit) | -1.10 | 0.3811 | 1.12 | 0.1930 | 1.02 | 0.8217 |
| *KDR* | 3791 | NM_002253 | kinase insert domain receptor (a type III receptor tyrosine kinase) | 1.31 | 0.2868 | -1.41 | 0.3319 | -1.07 | 0.8169 |
| *KRT14* | 3861 | NM_000526 | keratin 14 (epidermolysis bullosa simplex, Dowling-Meara, Koebner) | 4.01 | 0.1353 | -1.87 | 0.2014 | 2.14 | 0.2456 |
| *KRT18* | 3875 | BC000180 | keratin 18 | 3.52 | 0.1505 | -3.93 | 0.2534 | -1.12 | 0.8655 |
| *KRT18* | 3875 | BC004253 | keratin 18 | 3.51 | 0.1144 | -3.41 | 0.2878 | 1.03 | 0.9612 |
| *KRT18* | 3875 | NM_000224 | keratin 18 | 3.50 | 0.1258 | -3.99 | 0.2709 | -1.14 | 0.8457 |

| | | | | | | | | | |
|---|---|---|---|---|---|---|---|---|---|
| *KRT18* | 3875 | NM_199187 | keratin 18 | 3.66 | 0.1072 | -3.18 | 0.2828 | 1.15 | 0.8162 |
| *KRT19* | 3880 | BC007628 | keratin 19 | 2.55 | 0.0656 | -2.05 | 0.2812 | 1.24 | 0.6323 |
| *KRT19* | 3880 | NM_002276 | keratin 19 | 3.17 | 0.0561 | -2.32 | 0.1424 | 1.37 | 0.4596 |
| *LAG3* | 3902 | BC052589 | lymphocyte-activation gene 3 | 3.38 | 0.1585 | -1.84 | 0.3812 | 1.84 | 0.3450 |
| *LAG3* | 3902 | NM_002286 | lymphocyte-activation gene 3 | 4.91 | 0.1080 | -2.33 | 0.2711 | 2.11 | 0.2608 |
| *LDHA* | 3939 | NM_005566 | lactate dehydrogenase A | -1.45 | 0.0101 | 1.37 | 0.0300 | -1.06 | 0.6665 |
| *LEP* | 3952 | BC069323 | leptin (obesity homolog, mouse) | 1.86 | 0.2130 | -1.48 | 0.3945 | 1.26 | 0.5825 |
| *LEP* | 3952 | D49487 | leptin (obesity homolog, mouse) | 1.82 | 0.1530 | -1.87 | 0.3031 | -1.03 | 0.9498 |
| *LEP* | 3952 | NM_000230 | leptin (obesity homolog, mouse) | 1.41 | 0.0980 | -1.74 | 0.1090 | -1.24 | 0.3996 |
| *LRP1* | 4035 | BC045107 | low density lipoprotein-related protein 1 (alpha-2-macroglobulin receptor) | 1.46 | 0.1368 | -2.04 | 0.0019 | -1.40 | 0.0932 |
| *LRP1* | 4035 | BC052593 | low density lipoprotein-related protein 1 (alpha-2-macroglobulin receptor) | 2.38 | 0.1195 | -1.96 | 0.0839 | 1.22 | 0.6050 |
| *LRP1* | 4035 | NM_002332 | low density lipoprotein-related protein 1 (alpha-2-macroglobulin receptor) | 2.74 | 0.0746 | -2.09 | 0.0395 | 1.31 | 0.4658 |
| *LTBR* | 4055 | NM_002342 | lymphotoxin beta receptor (TNFR superfamily, member 3) | -1.03 | 0.7233 | -1.08 | 0.3121 | -1.12 | 0.1434 |
| *MMP2* | 4313 | BC002576 | matrix metallopeptidase 2 (gelatinase A, 72kDa gelatinase, 72kDa type IV collagenase) | -1.32 | 0.2175 | 1.10 | 0.6141 | -1.20 | 0.4554 |
| *MMP2* | 4313 | NM_004530 | matrix metallopeptidase 2 (gelatinase A, 72kDa gelatinase, 72kDa type IV collagenase) | -1.39 | 0.1049 | 1.23 | 0.2229 | -1.13 | 0.5998 |
| *MMP9* | 4318 | NM_004994 | matrix metallopeptidase 9 (gelatinase B, 92kDa gelatinase, 92kDa type IV collagenase) | 1.23 | 0.3265 | -1.07 | 0.7400 | 1.15 | 0.4512 |
| *MMP14* | 4323 | BC064803 | matrix metallopeptidase 14 (membrane-inserted) | 2.00 | 0.0780 | -1.64 | 0.0268 | 1.22 | 0.4626 |
| *MMP14* | 4323 | NM_004995 | matrix metallopeptidase 14 (membrane-inserted) | 2.03 | 0.0699 | -1.73 | 0.0104 | 1.17 | 0.5545 |
| *NFKB1* | 4790 | NM_003998 | nuclear factor of kappa light polypeptide gene enhancer in B-cells 1 (p105) | -1.56 | 0.0391 | 1.46 | 0.0020 | -1.07 | 0.7691 |
| *NFKB1* | 4790 | BC051765 | nuclear factor of kappa light polypeptide gene enhancer in B-cells 1 (p105) | -1.62 | 0.0471 | 1.47 | 0.0043 | -1.10 | 0.6976 |
| *NFKB1* | 4790 | M58603 | nuclear factor of kappa light polypeptide gene enhancer in B-cells 1 (p105) | -1.61 | 0.0557 | 1.49 | 0.0016 | -1.08 | 0.7673 |
| *NOS2A* | 4843 | NM_000625 | nitric oxide synthase 2A (inducible, hepatocytes) | 2.27 | 0.1319 | -1.34 | 0.1569 | 1.69 | 0.2238 |
| *NOS3* | 4846 | BC069465 | nitric oxide synthase 3 (endothelial cell) | 1.19 | 0.3465 | -1.15 | 0.4137 | 1.04 | 0.8344 |
| *NOS3* | 4846 | NM_000603 | nitric oxide synthase 3 (endothelial cell) | 1.18 | 0.2947 | -1.23 | 0.2274 | -1.05 | 0.7718 |
| *NPPA* | 4878 | BC005893 | natriuretic peptide precursor A | 1.86 | 0.0897 | -1.88 | 0.0853 | -1.01 | 0.9729 |
| *NPPA* | 4878 | NM_006172 | natriuretic peptide precursor A | 1.81 | 0.1393 | -1.61 | 0.2726 | 1.12 | 0.7538 |
| *SERPINE1* | 5054 | BC010860 | serpin peptidase inhibitor, clade E (nexin, plasminogen activator inhibitor type 1), m | -1.49 | 0.1378 | 1.21 | 0.4634 | -1.22 | 0.4975 |

| | | | | | | | | | |
|---|---|---|---|---|---|---|---|---|---|
| *SERPINE1* | 5054 | NM_000602 | serpin peptidase inhibitor, clade E (nexin, plasminogen activator inhibitor type 1), m | -1.40 | 0.0591 | 1.50 | 0.0011 | 1.07 | 0.6995 |
| *PDGFB* | 5155 | BC029822 | platelet-derived growth factor beta polypeptide (simian sarcoma viral (v-sis) oncogene | 1.52 | 0.1189 | -1.36 | 0.3134 | 1.12 | 0.6663 |
| *PDGFB* | 5155 | BC077725 | platelet-derived growth factor beta polypeptide (simian sarcoma viral (v-sis) oncogene | 2.46 | 0.0607 | -1.39 | 0.3890 | 1.77 | 0.1613 |
| *PDGFB* | 5155 | NM_002608 | platelet-derived growth factor beta polypeptide (simian sarcoma viral (v-sis) oncogene | 1.90 | 0.1122 | -1.28 | 0.2705 | 1.48 | 0.2415 |
| *PDGFB* | 5155 | X83705 | platelet-derived growth factor beta polypeptide (simian sarcoma viral (v-sis) oncogene | 1.56 | 0.2063 | -1.19 | 0.2542 | 1.31 | 0.3661 |
| *PDHA1* | 5160 | CR614489 | pyruvate dehydrogenase (lipoamide) alpha 1 | -1.19 | 0.0952 | 1.19 | 0.0305 | 1.00 | 0.9937 |
| *PDHA1* | 5160 | NM_000284 | pyruvate dehydrogenase (lipoamide) alpha 1 | -1.19 | 0.0646 | 1.16 | 0.0444 | -1.02 | 0.8173 |
| *PDHA2* | 5161 | NM_005390 | pyruvate dehydrogenase (lipoamide) alpha 2 | 3.86 | 0.1873 | -4.72 | 0.1455 | -1.22 | 0.7517 |
| *PDHB* | 5162 | NM_000925 | pyruvate dehydrogenase (lipoamide) beta | -1.58 | 0.0493 | 1.04 | 0.6541 | -1.52 | 0.0660 |
| *PDK1* | 5163 | BC039158 | pyruvate dehydrogenase kinase, isozyme 1 | -1.56 | 0.0159 | 1.48 | 0.0008 | -1.05 | 0.7545 |
| *PDK1* | 5163 | DQ234350 | pyruvate dehydrogenase kinase, isozyme 1 | -1.29 | 0.0220 | 1.37 | 0.0002 | 1.06 | 0.6045 |
| *PDK1* | 5163 | NM_002610 | pyruvate dehydrogenase kinase, isozyme 1 | -1.56 | 0.0184 | 1.62 | 0.0004 | 1.04 | 0.8349 |
| *PFKFB3* | 5209 | AF056320 | 6-phosphofructo-2-kinase/fructose-2,6-biphosphatase 3 | -1.15 | 0.1187 | 1.35 | 0.0004 | 1.18 | 0.0837 |
| *PFKFB3* | 5209 | AK131307 | 6-phosphofructo-2-kinase/fructose-2,6-biphosphatase 3 | -1.21 | 0.0726 | 1.38 | 0.0012 | 1.14 | 0.2844 |
| *PFKFB3* | 5209 | NM_004566 | 6-phosphofructo-2-kinase/fructose-2,6-biphosphatase 3 | -1.42 | 0.1680 | 1.57 | 0.0315 | 1.10 | 0.7134 |
| *PFKL* | 5211 | NM_001002021 | phosphofructokinase, liver | -1.02 | 0.8517 | -1.07 | 0.4940 | -1.09 | 0.2835 |
| *PFKL* | 5211 | BX537446 | phosphofructokinase, liver | 1.04 | 0.7122 | -1.10 | 0.3140 | -1.06 | 0.3226 |
| *PGK1* | 5230 | BC023234 | phosphoglycerate kinase 1 | -1.14 | 0.0638 | 1.17 | 0.0006 | 1.03 | 0.6513 |
| *PGK1* | 5230 | BC103752 | phosphoglycerate kinase 1 | -1.12 | 0.0762 | 1.14 | 0.0128 | 1.01 | 0.8636 |
| *PGK1* | 5230 | NM_000291 | phosphoglycerate kinase 1 | -1.10 | 0.0645 | 1.16 | 0.0120 | 1.06 | 0.3270 |
| *ABCB1* | 5243 | AF016535 | ATP-binding cassette, sub-family B (MDR/TAP), member 1 | 1.45 | 0.2028 | -1.69 | 0.0083 | -1.17 | 0.4901 |
| *ABCB1* | 5243 | NM_000927 | ATP-binding cassette, sub-family B (MDR/TAP), member 1 | -1.09 | 0.4238 | -1.57 | 0.0222 | -1.71 | 0.0021 |
| *PIK3CA* | 5290 | NM_006218 | phosphoinositide-3-kinase, catalytic, alpha polypeptide | -1.90 | 0.1379 | 2.72 | 0.0105 | 1.43 | 0.5275 |
| *PIK3CB* | 5291 | NM_006219 | phosphoinositide-3-kinase, catalytic, beta polypeptide | -1.93 | 0.0783 | 1.91 | 0.0168 | -1.01 | 0.9858 |
| *PIK3CB* | 5291 | CR749357 | phosphoinositide-3-kinase, catalytic, beta polypeptide | -1.97 | 0.0256 | 2.77 | 0.0027 | 1.41 | 0.2559 |

| | | | | | | | | | |
|---|---|---|---|---|---|---|---|---|---|
| *PIK3CD* | 5293 | U57843 | phosphoinositide-3-kinase, catalytic, delta polypeptide | 1.01 | 0.9384 | -1.19 | 0.1568 | -1.18 | 0.0636 |
| *PIK3CD* | 5293 | NM_005026 | phosphoinositide-3-kinase, catalytic, delta polypeptide | -1.20 | 0.0800 | -1.00 | 0.9756 | -1.20 | 0.1241 |
| *PIK3R1* | 5295 | BC030815 | phosphoinositide-3-kinase, regulatory subunit 1 (p85 alpha) | -1.81 | 0.1445 | 1.12 | 0.6583 | -1.61 | 0.2160 |
| *PIK3R1* | 5295 | BC094795 | phosphoinositide-3-kinase, regulatory subunit 1 (p85 alpha) | -1.73 | 0.2124 | 2.00 | 0.0887 | 1.15 | 0.7963 |
| *PIK3R1* | 5295 | NM_181504 | phosphoinositide-3-kinase, regulatory subunit 1 (p85 alpha) | -1.75 | 0.1192 | 1.44 | 0.1550 | -1.22 | 0.6005 |
| *PIK3R2* | 5296 | NM_005027 | phosphoinositide-3-kinase, regulatory subunit 2 (p85 beta) | -1.11 | 0.5099 | 1.29 | 0.1162 | 1.16 | 0.4206 |
| *PKM2* | 5315 | NM_002654 | pyruvate kinase, muscle | -1.02 | 0.5475 | -1.00 | 0.9841 | -1.03 | 0.5202 |
| *PLAUR* | 5329 | NM_001005376 | plasminogen activator, urokinase receptor | -1.20 | 0.0530 | 1.06 | 0.3086 | -1.13 | 0.1909 |
| *PLAUR* | 5329 | NM_001005377 | plasminogen activator, urokinase receptor | -1.25 | 0.1610 | 1.01 | 0.8963 | -1.23 | 0.1332 |
| *PLAUR* | 5329 | NM_002659 | plasminogen activator, urokinase receptor | -1.16 | 0.0727 | 1.00 | 0.9993 | -1.16 | 0.0432 |
| *PLCG1* | 5335 | NM_002660 | phospholipase C, gamma 1 | -1.44 | 0.0949 | -1.08 | 0.7319 | -1.56 | 0.1012 |
| *PLCG2* | 5336 | BC007565 | phospholipase C, gamma 2 (phosphatidylinositol-specific) | -1.16 | 0.1367 | 1.22 | 0.0138 | 1.05 | 0.6870 |
| *PLCG2* | 5336 | NM_002661 | phospholipase C, gamma 2 (phosphatidylinositol-specific) | -1.15 | 0.2443 | 1.13 | 0.1779 | -1.02 | 0.8704 |
| *PRKCA* | 5578 | BC109273 | protein kinase C, alpha | 1.61 | 0.1224 | -1.67 | 0.1692 | -1.04 | 0.8963 |
| *PRKCA* | 5578 | NM_002737 | protein kinase C, alpha | 1.85 | 0.0946 | -2.15 | 0.1615 | -1.16 | 0.6932 |
| *PRKCB1* | 5579 | NM_212535 | protein kinase C, beta 1 | -1.77 | 0.0166 | 1.57 | 0.0021 | -1.13 | 0.5976 |
| *PRKCB1* | 5579 | BC036472 | protein kinase C, beta 1 | -1.48 | 0.0457 | 1.30 | 0.0024 | -1.14 | 0.4714 |
| *PRKCB1* | 5579 | NM_002738 | protein kinase C, beta 1 | -2.01 | 0.0263 | 2.40 | 0.0075 | 1.19 | 0.5247 |
| *PRKCG* | 5582 | BC047876 | protein kinase C, gamma | 3.72 | 0.1731 | -1.64 | 0.5505 | 2.27 | 0.2723 |
| *PRKCG* | 5582 | NM_002739 | protein kinase C, gamma | 4.69 | 0.0718 | -2.14 | 0.2930 | 2.19 | 0.1819 |
| *MAPK1* | 5594 | NM_138957 | mitogen-activated protein kinase 1 | -1.72 | 0.0659 | 1.52 | 0.0501 | -1.13 | 0.7170 |
| *MAPK1* | 5594 | Z11695 | mitogen-activated protein kinase 1 | -1.86 | 0.0423 | 1.90 | 0.0043 | 1.02 | 0.9502 |
| *MAPK1* | 5594 | BC099905 | mitogen-activated protein kinase 1 | -2.07 | 0.0141 | 2.87 | 0.0000 | 1.38 | 0.4239 |
| *MAPK1* | 5594 | NM_002745 | mitogen-activated protein kinase 1 | -1.74 | 0.0409 | 3.65 | 0.0000 | 2.10 | 0.0628 |
| *MAPK3* | 5595 | NM_002746 | mitogen-activated protein kinase 3 | 1.09 | 0.1978 | -1.08 | 0.2932 | 1.00 | 0.9388 |
| *MAPK3* | 5595 | AY033607 | mitogen-activated protein kinase 3 | -1.14 | 0.5712 | -1.12 | 0.6197 | -1.28 | 0.2751 |
| *MAPK3* | 5595 | NM_001040056 | mitogen-activated protein kinase 3 | 1.02 | 0.8119 | -1.04 | 0.5941 | -1.02 | 0.8220 |
| *MAP2K1* | 5604 | NM_002755 | mitogen-activated protein kinase kinase 1 | -1.48 | 0.0695 | 1.68 | 0.0064 | 1.14 | 0.6025 |
| *MAP2K2* | 5605 | NM_030662 | mitogen-activated protein kinase kinase 2 | 1.05 | 0.3736 | -1.06 | 0.2654 | -1.01 | 0.8482 |
| *RELA* | 5970 | BC011603 | v-rel reticuloendotheliosis viral oncogene homolog A, nuclear factor of kappa light po | -1.23 | 0.3378 | -1.18 | 0.3729 | -1.46 | 0.0662 |
| *RELA* | 5970 | BC014095 | v-rel reticuloendotheliosis viral oncogene homolog A, nuclear factor of kappa light po | -1.15 | 0.4102 | -1.17 | 0.3473 | -1.34 | 0.0831 |

| | | | | | | | | | |
|---|---|---|---|---|---|---|---|---|---|
| *RELA* | 5970 | BC110830 | v-rel reticuloendotheliosis viral oncogene homolog A, nuclear factor of kappa light po | -1.24 | 0.2445 | -1.08 | 0.7330 | -1.33 | 0.2306 |
| *RELA* | 5970 | NM_021975 | v-rel reticuloendotheliosis viral oncogene homolog A, nuclear factor of kappa light po | -1.20 | 0.4070 | -1.17 | 0.4016 | -1.40 | 0.0892 |
| *RORC* | 6097 | BC031554 | RAR-related orphan receptor C | 1.13 | 0.3642 | -1.11 | 0.5271 | 1.02 | 0.8476 |
| *RORC* | 6097 | BC110571 | RAR-related orphan receptor C | 1.02 | 0.9228 | -1.29 | 0.3105 | -1.27 | 0.2847 |
| *RORC* | 6097 | NM_001001523 | RAR-related orphan receptor C | 1.06 | 0.7904 | 1.13 | 0.5695 | 1.20 | 0.4785 |
| *RPS6* | 6194 | NM_001010 | ribosomal protein S6 | 1.12 | 0.5066 | 1.18 | 0.4840 | 1.32 | 0.1430 |
| *RPS6KB1* | 6198 | BC053365 | ribosomal protein S6 kinase, 70kDa, polypeptide 1 | -1.64 | 0.1190 | 1.53 | 0.0512 | -1.07 | 0.8416 |
| *RPS6KB1* | 6198 | BC036033 | ribosomal protein S6 kinase, 70kDa, polypeptide 1 | -1.96 | 0.0633 | 1.83 | 0.0073 | -1.07 | 0.8602 |
| *RPS6KB1* | 6198 | NM_003161 | ribosomal protein S6 kinase, 70kDa, polypeptide 1 | -1.37 | 0.0077 | 1.79 | 0.0000 | 1.30 | 0.0454 |
| *RPS6KB2* | 6199 | BC000094 | ribosomal protein S6 kinase, 70kDa, polypeptide 2 | -1.09 | 0.5157 | -1.01 | 0.9501 | -1.10 | 0.4160 |
| *RPS6KB2* | 6199 | BC006106 | ribosomal protein S6 kinase, 70kDa, polypeptide 2 | -1.06 | 0.6860 | 1.01 | 0.9711 | -1.05 | 0.6819 |
| *RPS6KB2* | 6199 | NM_001007071 | ribosomal protein S6 kinase, 70kDa, polypeptide 2 | -1.06 | 0.6380 | -1.03 | 0.8221 | -1.09 | 0.4382 |
| *CCL2* | 6347 | NM_002982 | chemokine (C-C motif) ligand 2 | -1.44 | 0.0579 | 1.48 | 0.0365 | 1.03 | 0.8868 |
| *CXCL6* | 6372 | NM_002993 | chemokine (C-X-C motif) ligand 6 (granulocyte chemotactic protein 2) | -1.01 | 0.9159 | 1.03 | 0.8703 | 1.02 | 0.9094 |
| *SELL* | 6402 | BC020758 | selectin L (lymphocyte adhesion molecule 1) | -1.80 | 0.0908 | 2.43 | 0.0064 | 1.35 | 0.4707 |
| *SELL* | 6402 | NM_000655 | selectin L (lymphocyte adhesion molecule 1) | -1.61 | 0.0532 | 1.89 | 0.0012 | 1.18 | 0.5814 |
| *SELL* | 6402 | AJ246000 | selectin L (lymphocyte adhesion molecule 1) | -1.77 | 0.1066 | 2.04 | 0.0327 | 1.15 | 0.7248 |
| *SLC2A1* | 6513 | NM_006516 | solute carrier family 2 (facilitated glucose transporter), member 1 | -1.34 | 0.2891 | -1.17 | 0.5071 | -1.57 | 0.1082 |
| *SLC2A3* | 6515 | BC039196 | solute carrier family 2 (facilitated glucose transporter), member 3 | -1.51 | 0.0630 | 1.50 | 0.0099 | -1.01 | 0.9694 |
| *SLC2A3* | 6515 | NM_006931 | solute carrier family 2 (facilitated glucose transporter), member 3 | -1.40 | 0.0365 | 1.50 | 0.0007 | 1.07 | 0.7093 |
| *SLC11A1* | 6556 | BC041787 | solute carrier family 11 (proton-coupled divalent metal ion transporters), member 1 | 2.47 | 0.1280 | -1.23 | 0.6553 | 2.02 | 0.1458 |
| *SLC11A1* | 6556 | NM_000578 | solute carrier family 11 (proton-coupled divalent metal ion transporters), member 1 | 2.22 | 0.1128 | -1.42 | 0.1969 | 1.56 | 0.2633 |
| *SLC11A1* | 6556 | NM_001032220 | solute carrier family 11 (proton-coupled divalent metal ion transporters), member 1 | 2.05 | 0.1352 | -1.09 | 0.6820 | 1.88 | 0.1294 |
| *STAT3* | 6774 | BC000627 | signal transducer and activator of transcription 3 (acute-phase response factor) | -1.09 | 0.0755 | 1.31 | 0.0004 | 1.20 | 0.0056 |
| *STAT3* | 6774 | BC014482 | signal transducer and activator of transcription 3 (acute-phase response factor) | -1.07 | 0.1427 | 1.30 | 0.0008 | 1.21 | 0.0012 |

| | | | | | | | | | |
|---|---|---|---|---|---|---|---|---|---|
| *STAT3* | 6774 | NM_003150 | signal transducer and activator of transcription 3 (acute-phase response factor) | -1.67 | 0.1091 | 1.62 | 0.0404 | -1.03 | 0.9368 |
| *STAT3* | 6774 | NM_213662 | signal transducer and activator of transcription 3 (acute-phase response factor) | -1.67 | 0.1005 | 1.67 | 0.0366 | 1.00 | 0.9999 |
| *STAT4* | 6775 | NM_003151 | signal transducer and activator of transcription 4 | 1.23 | 0.0235 | -1.64 | 0.1542 | -1.33 | 0.2557 |
| *TCEB1* | 6921 | BC093065 | transcription elongation factor B (SIII), polypeptide 1 (15kDa, elongin C) | -1.38 | 0.2365 | 1.29 | 0.4157 | -1.07 | 0.6993 |
| *TCEB1* | 6921 | BC100028 | transcription elongation factor B (SIII), polypeptide 1 (15kDa, elongin C) | -1.40 | 0.2048 | 1.28 | 0.4324 | -1.09 | 0.6256 |
| *TCEB1* | 6921 | BC100283 | transcription elongation factor B (SIII), polypeptide 1 (15kDa, elongin C) | -1.32 | 0.3778 | 1.28 | 0.5054 | -1.03 | 0.8769 |
| *TCEB1* | 6921 | NM_005648 | transcription elongation factor B (SIII), polypeptide 1 (15kDa, elongin C) | -1.34 | 0.2935 | 1.19 | 0.6107 | -1.13 | 0.5461 |
| *TCEB2* | 6923 | BC013306 | transcription elongation factor B (SIII), polypeptide 2 (18kDa, elongin B) | 1.19 | 0.1920 | -1.05 | 0.6149 | 1.13 | 0.2826 |
| *TCEB2* | 6923 | BC065000 | transcription elongation factor B (SIII), polypeptide 2 (18kDa, elongin B) | 1.04 | 0.6348 | -1.08 | 0.1161 | -1.04 | 0.5314 |
| *TCEB2* | 6923 | NM_007108 | transcription elongation factor B (SIII), polypeptide 2 (18kDa, elongin B) | -1.07 | 0.5593 | -1.02 | 0.7810 | -1.09 | 0.4363 |
| *TCEB2* | 6923 | NM_207013 | transcription elongation factor B (SIII), polypeptide 2 (18kDa, elongin B) | -1.03 | 0.7214 | -1.05 | 0.3642 | -1.07 | 0.3240 |
| *TCF7* | 6932 | BC048769 | transcription factor 7 (T-cell specific, HMG-box) | -1.26 | 0.1897 | 1.17 | 0.4467 | -1.07 | 0.6812 |
| *TCF7* | 6932 | NM_003202 | transcription factor 7 (T-cell specific, HMG-box) | -1.02 | 0.8709 | 1.11 | 0.4446 | 1.09 | 0.5039 |
| *TCF7* | 6932 | NM_201633 | transcription factor 7 (T-cell specific, HMG-box) | 1.67 | 0.1336 | -1.43 | 0.3279 | 1.17 | 0.6438 |
| *TEK* | 7010 | NM_000459 | TEK tyrosine kinase, endothelial (venous malformations, multiple cutaneous and mucosal | -1.08 | 0.1633 | -1.23 | 0.1832 | -1.33 | 0.0396 |
| *TF* | 7018 | AK126941 | transferrin | 1.17 | 0.2817 | -1.40 | 0.1470 | -1.20 | 0.3531 |
| *TF* | 7018 | NM_001063 | transferrin | 2.59 | 0.1642 | -1.57 | 0.4255 | 1.65 | 0.3330 |
| *TFF3* | 7033 | BC017859 | trefoil factor 3 (intestinal) | 4.00 | 0.1445 | -1.25 | 0.4870 | 3.21 | 0.1383 |
| *TFF3* | 7033 | NM_003226 | trefoil factor 3 (intestinal) | 5.03 | 0.1387 | -1.97 | 0.1643 | 2.55 | 0.2121 |
| *TFRC* | 7037 | BC001188 | transferrin receptor (p90, CD71) | -1.98 | 0.0548 | 2.27 | 0.0007 | 1.14 | 0.7746 |
| *TFRC* | 7037 | NM_003234 | transferrin receptor (p90, CD71) | -2.07 | 0.0428 | 2.29 | 0.0005 | 1.11 | 0.8240 |
| *TGFA* | 7039 | AF149096 | transforming growth factor, alpha | -1.13 | 0.3324 | 1.07 | 0.6057 | -1.06 | 0.5710 |
| *TGFA* | 7039 | AF149098 | transforming growth factor, alpha | -1.12 | 0.4613 | 1.19 | 0.3705 | 1.07 | 0.6880 |
| *TGFA* | 7039 | BC005308 | transforming growth factor, alpha | -1.04 | 0.7025 | 1.57 | 0.0027 | 1.50 | 0.0041 |
| *TGFA* | 7039 | NM_003236 | transforming growth factor, alpha | -1.43 | 0.1833 | 3.14 | 0.0029 | 2.20 | 0.0157 |

| | | | | | | | | | |
|---|---|---|---|---|---|---|---|---|---|
| *TGFB1* | 7040 | BC000125 | transforming growth factor, beta 1 (Camurati-Engelmann disease) | 1.02 | 0.7315 | -1.06 | 0.3919 | -1.05 | 0.4635 |
| *TGFB1* | 7040 | NM_000660 | transforming growth factor, beta 1 (Camurati-Engelmann disease) | -1.01 | 0.8357 | 1.02 | 0.7733 | 1.00 | 0.9462 |
| *TGFB3* | 7043 | BC018503 | transforming growth factor, beta 3 | -1.10 | 0.4774 | 1.45 | 0.0204 | 1.32 | 0.0708 |
| *TGFB3* | 7043 | NM_003239 | transforming growth factor, beta 3 | -1.50 | 0.0223 | 1.50 | 0.0021 | -1.00 | 0.9888 |
| *THBS1* | 7057 | NM_003246 | thrombospondin 1 | 1.04 | 0.8452 | 1.31 | 0.0410 | 1.35 | 0.1233 |
| *TIMP1* | 7076 | BC000866 | TIMP metallopeptidase inhibitor 1 | -1.01 | 0.9314 | 1.14 | 0.1804 | 1.13 | 0.1788 |
| *TIMP1* | 7076 | NM_003254 | TIMP metallopeptidase inhibitor 1 | -1.05 | 0.4277 | 1.18 | 0.0911 | 1.12 | 0.1886 |
| *TLR4* | 7099 | NM_003266 | toll-like receptor 4 | -1.98 | 0.0839 | 1.97 | 0.0233 | -1.01 | 0.9910 |
| *TLR4* | 7099 | NM_138554 | toll-like receptor 4 | -1.60 | 0.1461 | 1.87 | 0.0194 | 1.17 | 0.6716 |
| *TLR4* | 7099 | NM_138557 | toll-like receptor 4 | -1.89 | 0.1620 | 1.89 | 0.0754 | 1.00 | 0.9944 |
| *TNF* | 7124 | NM_000594 | tumor necrosis factor (TNF superfamily, member 2) | 1.17 | 0.0986 | -1.43 | 0.0630 | -1.22 | 0.1462 |
| *TPI1* | 7167 | BC007086 | triosephosphate isomerase 1 | 1.01 | 0.8098 | 1.01 | 0.7052 | 1.02 | 0.5877 |
| *TPI1* | 7167 | BC017917 | triosephosphate isomerase 1 | 1.02 | 0.5229 | 1.01 | 0.5823 | 1.03 | 0.3180 |
| *TPI1* | 7167 | BC070129 | triosephosphate isomerase 1 | -1.01 | 0.8885 | 1.10 | 0.2055 | 1.09 | 0.0830 |
| *TPI1* | 7167 | NM_000365 | triosephosphate isomerase 1 | 1.00 | 0.9027 | 1.02 | 0.6912 | 1.02 | 0.6306 |
| *TNFRSF4* | 7293 | NM_003327 | tumor necrosis factor receptor superfamily, member 4 | 3.37 | 0.2294 | -1.23 | 0.2254 | 2.73 | 0.2297 |
| *VEGF* | 7422 | AF323587 | vascular endothelial growth factor | 1.14 | 0.2088 | -1.18 | 0.5878 | -1.03 | 0.8854 |
| *VEGF* | 7422 | AY263145 | vascular endothelial growth factor | -1.30 | 0.2222 | 1.33 | 0.1066 | 1.02 | 0.8941 |
| *VEGF* | 7422 | M27281 | vascular endothelial growth factor | -1.20 | 0.1624 | 1.31 | 0.0397 | 1.09 | 0.4574 |
| *VEGF* | 7422 | NM_001025366 | vascular endothelial growth factor | -1.18 | 0.2346 | 1.45 | 0.0050 | 1.22 | 0.1805 |
| *VEGF* | 7422 | S85192 | vascular endothelial growth factor | -1.47 | 0.1457 | 1.51 | 0.0689 | 1.02 | 0.9316 |
| *VHL* | 7428 | BC058831 | von Hippel-Lindau tumor suppressor | -1.61 | 0.0856 | 1.57 | 0.0335 | -1.03 | 0.9198 |
| *VHL* | 7428 | L15409 | von Hippel-Lindau tumor suppressor | -1.70 | 0.1762 | 1.92 | 0.0550 | 1.13 | 0.7751 |
| *VHL* | 7428 | NM_000551 | von Hippel-Lindau tumor suppressor | -1.60 | 0.0936 | 1.45 | 0.0802 | -1.10 | 0.7249 |
| *VIM* | 7431 | AK093924 | vimentin | -1.12 | 0.5931 | -1.34 | 0.4869 | -1.50 | 0.2765 |
| *VIM* | 7431 | BC030573 | vimentin | -1.32 | 0.1236 | 1.22 | 0.0912 | -1.09 | 0.6041 |
| *VIM* | 7431 | BC066956 | vimentin | -1.34 | 0.0971 | 1.16 | 0.1901 | -1.15 | 0.3297 |
| *VIM* | 7431 | NM_003380 | vimentin | -1.43 | 0.0966 | 1.33 | 0.0605 | -1.08 | 0.7138 |
| *CXCR4* | 7852 | NM_001008540 | chemokine (C-X-C motif) receptor 4 | -1.19 | 0.0350 | 1.40 | 0.0005 | 1.17 | 0.1040 |
| *EOMES* | 8320 | NM_005442 | eomesodermin homolog (Xenopus laevis) | 2.17 | 0.0817 | -1.95 | 0.2977 | 1.11 | 0.8075 |
| *CUL2* | 8453 | NM_003591 | cullin 2 | -1.88 | 0.0770 | 1.62 | 0.0371 | -1.16 | 0.7219 |
| *PIK3R3* | 8503 | AF028785 | phosphoinositide-3-kinase, regulatory subunit 3 (p55, gamma) | -1.90 | 0.1696 | 2.25 | 0.0419 | 1.19 | 0.7480 |
| *PIK3R3* | 8503 | NM_003629 | phosphoinositide-3-kinase, regulatory subunit 3 (p55, gamma) | -1.85 | 0.1051 | 2.59 | 0.0055 | 1.40 | 0.4885 |
| *MKNK1* | 8569 | AB000409 | MAP kinase interacting serine/threonine kinase 1 | -1.36 | 0.2366 | 1.23 | 0.3638 | -1.10 | 0.7126 |

| | | | | | | | | | |
|---|---|---|---|---|---|---|---|---|---|
| *MKNK1* | 8569 | AK096423 | MAP kinase interacting serine/threonine kinase 1 | -1.26 | 0.2628 | 1.14 | 0.4884 | -1.10 | 0.6424 |
| *MKNK1* | 8569 | NM_003684 | MAP kinase interacting serine/threonine kinase 1 | -1.23 | 0.2770 | 1.16 | 0.3972 | -1.06 | 0.7557 |
| *MKNK1* | 8569 | NM_198973 | MAP kinase interacting serine/threonine kinase 1 | -1.37 | 0.2128 | 1.21 | 0.3865 | -1.14 | 0.6020 |
| *TNFRSF18* | 8784 | NM_004195 | tumor necrosis factor receptor superfamily, member 18 | 2.94 | 0.1252 | -1.74 | 0.0208 | 1.69 | 0.2878 |
| *TNFRSF18* | 8784 | NM_148901 | tumor necrosis factor receptor superfamily, member 18 | 3.91 | 0.2270 | -1.52 | 0.0226 | 2.57 | 0.2692 |
| *NRP1* | 8829 | AF268691 | neuropilin 1 | -1.29 | 0.1054 | 1.09 | 0.2621 | -1.18 | 0.2841 |
| *NRP1* | 8829 | AF280547 | neuropilin 1 | 1.20 | 0.2619 | -1.73 | 0.0534 | -1.45 | 0.1005 |
| *NRP1* | 8829 | NM_001024628 | neuropilin 1 | -1.31 | 0.1622 | 1.21 | 0.1515 | -1.08 | 0.6859 |
| *NRP1* | 8829 | NM_001024629 | neuropilin 1 | -1.23 | 0.2473 | 1.15 | 0.2721 | -1.07 | 0.6715 |
| *NRP1* | 8829 | NM_003873 | neuropilin 1 | -1.74 | 0.0802 | 1.92 | 0.0111 | 1.10 | 0.7752 |
| *EIF4E2* | 9470 | NM_004846 | eukaryotic translation initiation factor 4E member 2 | -1.05 | 0.7364 | 1.41 | 0.0729 | 1.34 | 0.0091 |
| *RBX1* | 9978 | NM_014248 | ring-box 1 | -1.70 | 0.0290 | 1.21 | 0.2500 | -1.40 | 0.0817 |
| *AKT3* | 10000 | NM_005465 | v-akt murine thymoma viral oncogene homolog 3 (protein kinase B, gamma) | -1.93 | 0.1343 | 2.57 | 0.0271 | 1.33 | 0.5833 |
| *AKT3* | 10000 | NM_181690 | v-akt murine thymoma viral oncogene homolog 3 (protein kinase B, gamma) | -2.04 | 0.1318 | 2.28 | 0.0572 | 1.12 | 0.8431 |
| *TBX21* | 30009 | NM_013351 | T-box 21 | 2.74 | 0.0924 | -1.92 | 0.1645 | 1.43 | 0.4050 |
| *AK3* | 50808 | BC013771 | adenylate kinase 3 | -1.79 | 0.1222 | 1.81 | 0.0362 | 1.01 | 0.9817 |
| *AK3* | 50808 | NM_016282 | adenylate kinase 3 | -1.91 | 0.0528 | 3.01 | 0.0003 | 1.58 | 0.3176 |
| *FOXP3* | 50943 | NM_014009 | forkhead box P3 | 2.45 | 0.0717 | -1.87 | 0.0423 | 1.31 | 0.4424 |
| *ANGPT4* | 51378 | BC111976 | angiopoietin 4 | 1.27 | 0.0948 | -1.28 | 0.1763 | -1.01 | 0.9536 |
| *ANGPT4* | 51378 | BC111978 | angiopoietin 4 | 1.00 | 0.9880 | -1.24 | 0.3289 | -1.24 | 0.1983 |
| *ANGPT4* | 51378 | NM_015985 | angiopoietin 4 | 1.43 | 0.0512 | -1.39 | 0.0839 | 1.03 | 0.8539 |
| *EGLN1* | 54583 | BC005369 | egl nine homolog 1 (C. elegans) | -1.45 | 0.0941 | 1.50 | 0.0380 | 1.04 | 0.8704 |
| *EGLN1* | 54583 | NM_022051 | egl nine homolog 1 (C. elegans) | -1.66 | 0.1747 | 1.57 | 0.1582 | -1.06 | 0.8868 |
| *HIF1AN* | 55662 | AK025680 | hypoxia-inducible factor 1, alpha subunit inhibitor | -1.56 | 0.1871 | -1.00 | 0.9944 | -1.56 | 0.1677 |
| *HIF1AN* | 55662 | BC007719 | hypoxia-inducible factor 1, alpha subunit inhibitor | -1.47 | 0.1531 | -1.00 | 0.9970 | -1.47 | 0.1783 |
| *HIF1AN* | 55662 | NM_017902 | hypoxia-inducible factor 1, alpha subunit inhibitor | -1.22 | 0.2476 | -1.07 | 0.6816 | -1.30 | 0.1725 |
| *HAMP* | 57817 | NM_021175 | hepcidin antimicrobial peptide | 3.25 | 0.0767 | -1.95 | 0.1779 | 1.67 | 0.2668 |
| *HKDC1* | 80201 | AK026414 | hexokinase domain containing 1 | 1.47 | 0.1634 | -1.57 | 0.0534 | -1.07 | 0.7807 |
| *HKDC1* | 80201 | BC110504 | hexokinase domain containing 1 | 1.75 | 0.0378 | -1.62 | 0.1691 | 1.09 | 0.7592 |
| *HKDC1* | 80201 | NM_025130 | hexokinase domain containing 1 | 1.87 | 0.1029 | -1.90 | 0.1135 | -1.02 | 0.9533 |
| *PROK1* | 84432 | NM_032414 | prokineticin 1 | 2.25 | 0.1851 | -1.80 | 0.1839 | 1.25 | 0.6059 |

| | | | | | | | | | |
|---|---|---|---|---|---|---|---|---|---|
| *RETNLB* | 84666 | BC069318 | resistin like beta | 1.87 | 0.2629 | -2.27 | 0.2871 | -1.21 | 0.7219 |
| *RETNLB* | 84666 | NM_032579 | resistin like beta | 1.72 | 0.2424 | -2.01 | 0.3197 | -1.17 | 0.7577 |
| *EGLN2* | 112398 | NM_017555 | egl nine homolog 2 (C. elegans) | -1.12 | 0.3876 | 1.07 | 0.6528 | -1.04 | 0.7830 |
| *EGLN2* | 112398 | NM_053046 | egl nine homolog 2 (C. elegans) | -1.02 | 0.7879 | 1.00 | 0.9624 | -1.02 | 0.7897 |
| *EGLN3* | 112399 | AK123350 | egl nine homolog 3 (C. elegans) | 1.31 | 0.2688 | -1.40 | 0.0278 | -1.07 | 0.7287 |
| *EGLN3* | 112399 | NM_022073 | egl nine homolog 3 (C. elegans) | -1.12 | 0.2563 | -1.16 | 0.4483 | -1.30 | 0.1341 |
| *FLJ36951* | 253314 | XM_171094 | similar to eukaryotic translation initiation factor 4e 1a | 1.52 | 0.4884 | 1.05 | 0.9408 | 1.59 | 0.3160 |
| *FLJ36951* | 253314 | XM_927854 | similar to eukaryotic translation initiation factor 4e 1a | 1.72 | 0.0537 | -1.52 | 0.1337 | 1.13 | 0.6048 |
| *FLJ36951* | 253314 | XM_932348 | similar to eukaryotic translation initiation factor 4e 1a | 3.02 | 0.1274 | -1.82 | 0.3622 | 1.66 | 0.3142 |
| *DKFZp781 N1041* | 387712 | XM_370577 | similar to RIKEN cDNA 6430537H07 gene | -1.04 | 0.8982 | -1.21 | 0.5278 | -1.26 | 0.2926 |
